# Enantioselective nickel-catalyzed dicarbofunctionalization of 3,3,3-trifluoropropene

**DOI:** 10.1038/s41467-022-33159-2

**Published:** 2022-09-21

**Authors:** Yun-Ze Li, Na Rao, Lun An, Xiao-Long Wan, Yanxia Zhang, Xingang Zhang

**Affiliations:** 1 Key Laboratory of Organofluorine Chemistry, Center for Excellence in Molecular Synthesis, Shanghai Institute of Organic Chemistry, University of Chinese Academy of Sciences, Chinese Academy of Sciences, 345 Lingling Road, 200032 Shanghai, China; 2 Henan Institute of Advanced Technology, Zhengzhou University, 450001 Zhengzhou, P.R. China

**Keywords:** Synthetic chemistry methodology, Stereochemistry

## Abstract

Despite paramount applications of chiral trifluoromethylated compounds in medicinal chemistry and materials science, limited strategies have been developed for catalytic asymmetric synthesis of such valuable fluorinated structures. Here, we report a nickel catalyzed enantioselective dicarbofunctionalization of inexpensive industrial chemical 3,3,3-trifluoropropene (TFP) with readily available tertiary alkyl and aryl iodides. The reaction overcomes the β-F elimination side reaction of TFP, and proceeds efficiently under mild reaction conditions. The protocol possesses advantages, such as synthetic convenience, high enantioselectivity, and excellent functional group tolerance, providing rapid and straightforward access to chiral trifluoromethylated compounds of medicinal interest.

## Abstract (web summary)

Strategies for the catalytic asymmetric synthesis of trifluoromethylated compounds remain scarce. Here, the authors report the nickel-catalyzed enantioselective dicarbofunctionalization of 3,3,3-trifluoropropene.

## Introduction

**Fig. 1:**
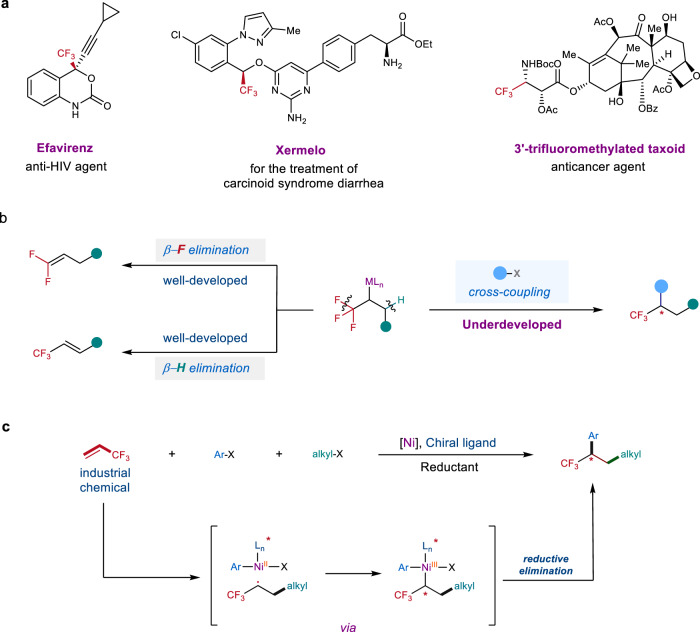
Nickel-catalyzed enantioselective dicarbofunctionalization of TFP and representative biologically active molecules bearing an α-CF_3_ stereogenic center. **a** Biologically active molecules bearing an α-CF_3_ stereogenic center. **b** Pathways for α-CF_3_ metal species. **c** Nickel-catalyzed enantioselective dicarbofunctionalization of TFP (this work).

Trifluoromethy group (CF_3_) represents one of the prominent functional groups in developing pharmaceuticals, agrochemicals, and advanced functional materials^1–4^. Particularly, the tactical introduction of CF_3_ into organic molecules can significantly improve their biological properties^5–7^. Consequently, numerous therapeutic drugs contain CF_3_ moiety. Over the past decades, impressive achievements on the site-selective trifluoromethylations have been made. However, many of developed methods mainly focus on the synthesis of trifluoromethylated arenes^8–10^, the construction of Csp^3^–CF_3_ bond at a stereogenic center remains a challenging topic (Fig. 1a). To date, only limited strategies for catalytic asymmetric trifluoromethylation have been reported^11–13^. Using Lewis acids^14,15^ or organocatalysis^11,13,16–19^ can enantioselectively construct the Csp^3^–CF_3_ bond at a stereogenic center, but shows efficiency with carbonyl compounds and their derivatives. Transition-metal catalyzed asymmetric trifluoromethylation via cross-coupling reactions is an alternative route to access chiral trifluoromethylated compounds^20,21^. However, the lack of an efficient catalytic system and limited substrate scope restrict its widespread synthetic applications. To circumvent these limitations, the use of low-cost and widely available prochiral CF_3_-containing chemical feedstocks as starting materials to asymmetrically synthesize chiral trifluoromethylated compounds shows great advantages, because they are ready for cost-efficiently diversified transformations.

As an inexpensive and bulk industrial chemical, 3,3,3-trifluoropropene (TFP) has important applications in the production of fluorinated materials^22^ and refrigerants^23^, and should be a cost-effective and versatile linchpin to access chiral trifluoromethylated compounds. However, only limited efficient strategies for transformations of TFP have been developed^24^. To date, the asymmetric synthesis of trifluoromethylated compounds from TFP remains severely underdeveloped. Only rare examples that rely on the asymmetric dihydroxylation^25^ and hydroformylation^26^ of TFP have been reported. The asymmetric formation of a C–C bond at α-CF_3_ stereogenic center from TFP remains challenging. Usually, the installation of a functional group, such as an electron-withdrawing group and π-system, onto TFP is needed to facilitate the asymmetric process^12,27,28^. Despite effectiveness of these approaches, they require additional steps to prepare the trifluoromethylated alkenes. As such, to overcome these limitations, it is highly desirable to develop an efficient method that can provide a straightforward and cost-efficient access to chiral trifluoromethylated compounds through stereoselective transformations of TFP.

In 2016, we developed a chelation-assisted strategy for nickel-catalyzed dicarbofunctionalization of alkenes, in which the newly formed chiral center could be stereoselectively controlled by a chiral nickel catalyst to provide an 18% ee^29^. Inspired by this work, we hypothesized that the nickel-catalyzed asymmetric dicarbofunctionalization of TFP would be a promising strategy to construct the valuable chiral trifluomethylated compounds^30–37^. Because such a strategy can simultaneously form two new carbon-carbon bonds and generate one new α-CF_3_ stereogenic center without tedious synthetic procedure, thereby providing an attractive opportunity to diversely construct complex chiral trifluoromethylated structures. However, this process is plagued by the high tendency of β-H^38–40^ and β-F^39–43^ eliminations from the resulting α-CF_3_ alkyl-metal intermediate (Fig. 1b). We envisioned that with the aid of a suitable chiral ligand, the α-CF_3_ stereogenic center would be enantioselectively controlled by reaction of a chiral nickel(II) species with the α-CF_3_ alkyl radical that was generated by fast radical addition of TFP catalyzed by nickel complex; subsequently, the fast reductive elimination of the resulting highly active α-CF_3_ alkylnickel(III) intermediate would suppress the β-H and β-F eliminations (Fig. 1c).

Herein, we disclose an enantioselective nickel-catalyzed dicarbofunctionalization of TFP with aryl and tertiary iodides. The approach features excellent functional group tolerance and synthetic convenience without the preparation of organometallic reagents, providing efficient and straightforward access to a variety of chiral trifluoromethylated compounds.

## Results

### Optimization of nickel-catalyzed enantioselective alkyl-arylation of TFP

To test our hypothesis, we chose two electrophiles to couple with TFP, as this strategy could omit the preparation of organometallic reagents, and would provide rapid and straightforward access to chiral trifluoromethylated compounds. Initially, *tert*-butyl iodide **2a** and ethyl 4-iodobenzoate **3a** were chosen as the model substrates for asymmetric dicarbofunctionalization of TFP (Table 1). In a reaction that was carried out with TFP (1.6 equiv), **2a** (1.5 equiv), and **1a** (1.0 equiv) in the presence of NiBr_2_·DME (10 mol%) and reductant Zn in dioxane at room temperature, a high yield (84%) of **4a** was obtained using an achiral ligand 4,4’-di*t*-Bu-bpy (**L1**) (entry 1). Encouraged by this result, a survey of chiral ligands was conducted (entries 2-8). We found that bis(oxazoline) (Biox) ligands showed a beneficial effect on the enantioselectivity (entries 2-6), and isobutyl-substituted chiral Biox (**L6**) was identified as the best ligand, providing **4a** in 69% yield and 90% ee (entry 6). The same enantioselectivity with a slightly lower yield (66%) could be obtained by using Cy-Biox (**L3**) (entry 3), but other Biox ligands led to lower yields and enantioselectivities (entries 2, 4, 5). Pyox ligand was proved to be less effective (entry 7), and even no reaction occurred using Box or diamine ligands (entry 8). Further optimization of the reaction conditions by using DME instead of 1,4-dioxane as the solvent could improve the yield of **4a** to 70% with 91% ee (entry 9). Other nickel catalysts were also examined. Slightly lower yields and comparable enantioselectivities were obtained by using NiCl_2_·DME or NiBr_2_·diglyme, but no reaction occurred with NiCl_2_ or NiBr_2_ (Supplementary Table 3). The absence of nickel catalyst or Biox ligand failed to provide desired product **4a** (entries 10, 11), demonstrating the essential role of [Ni/**L**] in promoting the reaction.

**Table 1:** Representative results for the optimization of the reaction conditions^a^


Entry	Ligand	Yield (%)^b^	ee (%)^c^
1	**L1**	84	–
2	**L2**	51	87
3	**L3**	66	90
4	**L4**	72	69
5	**L5**	61	63
6	**L6**	69	90
7	**L7**	52	13
8	**L8-L9**	nd	–
9^d^	**L6**	74 (70)	91
10^d,e^	**L6**	nd	–
11^d^	None	nd	–

*nd* not detected, *DMA* dimethylacetamide, *DME* dimethyl ether.

^a^Reaction conditions (unless otherwise specified): **1** (0.64 mmol, 0.54 mL, 1.2 M in DMA, 1.6 equiv), **2a** (0.6 mmol, 1.5 equiv), **3a** (0.4 mmol, 1.0 equiv), Zn (0.6 mmol, 1.5 equiv), 1,4-dioxane (3.2 mL).

^b^The yield was determined by ^19^F NMR using benzotrifluoride as an internal standard, and number in parentheses is the isolated yield.

^c^Determined by chiral HPLC.

^d^DME instead of 1,4-dioxane was used.

^e^Without nickel catalyst.

### Scope of the nickel-catalyzed enantioselective alkyl-arylation of TFP

**Fig. 2:**
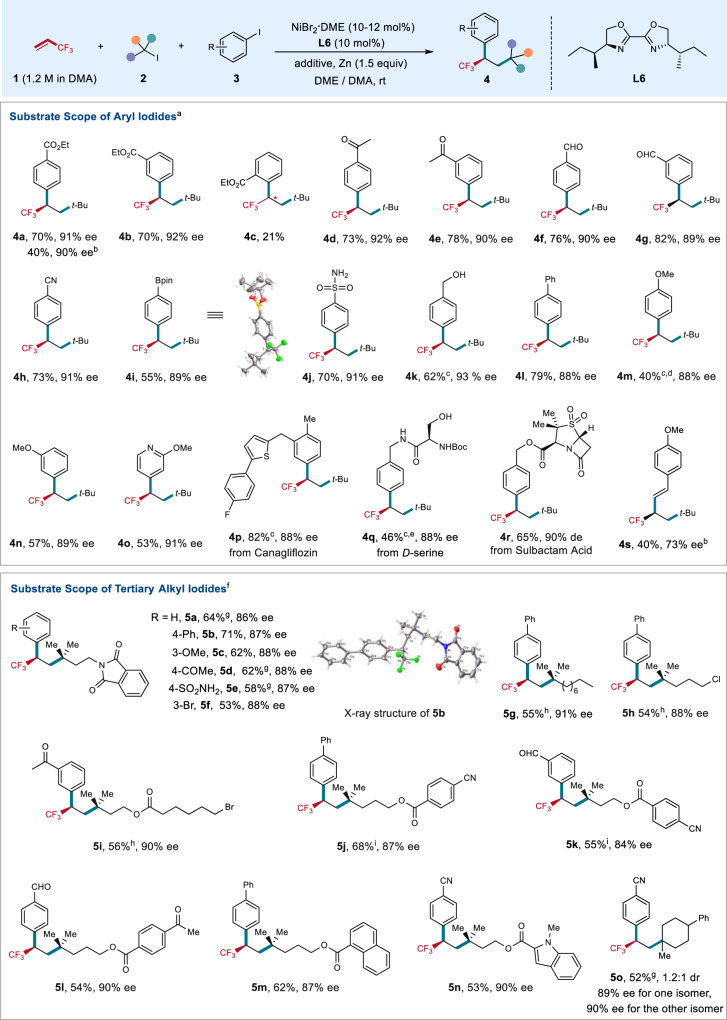
Scope of the nickel-catalyzed enantioselective alkyl-arylation of TFP. ^a^Conditions **A** (unless otherwise specified): NiBr_2_·DME (10 mol%), **L6** (10 mol%), **1** (1.2 M in DMA, 1.6 equiv), **2** (1.5 equiv), **3** (0.4 mmol, 1.0 equiv), DME (3.2 mL). All reported yields are isolated yields. ^b^Aryl bromide or vinyl bromide was used. ^c^0.5 equiv of NaI was used. ^d^10 mol% DMAP, **2** (0.4 mmol, 1.0 equiv) and **3** (0.6 mmol, 1.5 equiv) were used. ^e^NiBr_2_·DME (13 mol%) and **L6** (13 mol%) were used. ^f^Conditions **B** (unless otherwise specified): NiBr_2_·DME (12 mol%), **L6** (10 mol%), **1** (1.2 M in DMA, 1.6 equiv), **2** (1.5 equiv), **3** (0.2 mmol, 1.0 equiv), FeCl_3_ (0.25 equiv), DME (1.6 mL). All reported yields are isolated yields. ^g^FeBr_2_ (0.25 equiv) instead of FeCl_3_ (0.25 equiv) was used. ^h^0.5 equiv of NaI instead of FeCl_3_ (0.25 equiv) was used. ^i^No additive was used. DMAP, *N*,*N*-dimethylpyridin-4-amine.

With the viable reaction conditions in hand, the substrate scope of aryl iodides **3** toward dicarbofunctionalization of TFP with *tert*-butyl iodide **2a** was examined. As shown in Fig. 2, a variety of aryl iodides bearing substituents with different electronic nature were applicable to the reaction, providing the corresponding products with good yields and high enantioselectivities, ranging from 88 to 92% ee. Generally, electron-deficient aryl iodides (**4a**–**4h**, **4n**) provided higher yields than electron-rich substrate (**4m**). Aryl bromide was also applicable to the reaction, with a lower yield and comparable ee obtained (**4a**). However, aryl chlorides were not suitable substrates. The reaction exhibited excellent functional group tolerance. Synthetically versatile handles, such as base and nucleophile sensitive ester, enolizable ketone, aldehyde, and cyanide, were compatible with the reaction (**4a**–**4h**). The successful formation of **4i** with intact boronate further demonstrated the synthetic advance of the current nickel-catalyzed process. Notably, substrates bearing acidic proton, such as sulfonamide (**4j**) and alcohol (**4k**), are competent coupling partners, with good yields and high enantioselectivities obtained. Pyridine- and thiophene-containing aryl iodides underwent the coupling smoothly without loss of enanotioselectivity (**4o**, **4p**). The substrate scope of aryl iodides can also be extended to complex molecules. *D*-Serine derived substrate with unprotected hydroxyl group furnished corresponding product **4q** in synthetically useful yield and high enantioselectivity. Sulbactam-derived aryl iodide bearing two additional chiral centers did not influence the stereoselectivity, and afforded **4r** in good yield and high diastereoselectivity (90% de), thus providing a potential opportunity for applications in medicinal chemistry. However, the reaction was sensitive to the sterically hindered substrates. For instance, *ortho*-substituted aryl iodide led to poor yield (**4c**). The reaction can also be extended to vinyl bromide, providing corresponding product **4s** with a synthetically useful yield and moderate ee. In some cases (**4k**, **4m**, **4p**, **4q**), the addition of NaI to the reaction could provide slightly higher yields, possibly because NaI can facilitate the reduction of the nickel catalyst or form a nickelate species to facilitate the catalytic cycle^44,45^.

In addition to *tert*-butyl iodide **2a**, tertiary alkyl iodides **2** bearing different side chains were also examined (Fig. 2). We found that low yields were obtained under standard reactions due to the relatively low conversion of aryl iodides. Increasing the loading amount of NiBr_2_·DME from 10 to 12 mol% with FeCl_3_ or FeBr_2_^46–48^ as the additive benefited the conversion of aryl iodides and provided the chiral trifluoromethylated compounds in good yields and high ee (Supplementary Tables 4–10). The possible role of the ion salts is likely to facilitate the reduction of nickel(II) species, thus benefiting the catalytic cycle. However, the exact role of these iron salts in the reaction remains elusive at this stage. Tertiary alkyl iodide substituted with a phthalimide underwent the reaction efficiently, allowing the dicarbofunctionalization of TFP with a variety of aryl iodides with high ee and excellent functional group tolerance (**5a**–**5e**). Remarkably, 1-bromo-3-iodobenzene was also amenable to the reaction with selective formation of C–C bond at aryl iodide position (**5f**). Given that aryl bromides have widespread synthetic applications in organic synthesis, the survival of aryl bromide in the current process further demonstrated the synthetic utility of this protocol. The side chains in tertiary alkyl iodides with different chain lengths did not affect the reaction efficiency and enantioselectivities. For instance, good yields and high ee were provided with substrate bearing a long aliphatic side chain of eight carbons (**5g**), as well as that bearing a shorter carbon chain of three carbons (**5h**). In these cases, NaI instead of iron salts, was needed to facilitate the reaction. In addition, tertiary alkyl iodides **3** substituted with different important functional groups, such as ester, alkyl bromide, cyanide, ketone, and indole moieties, furnished the corresponding products efficiently (**5i**–**5n**). Last, the cyclic substrate was also applicable to the reaction, showing high ee (89–90%) and 1.2:1 dr (**5o**). The absolute configuration of the chiral trifluoromethylated products **4** and **5** was determined to be *R* by X-ray crystal structure analysis of compounds **4i** and **5b**. However, the primary and secondary alkyl halides as well as the substituted TFPs were not applicable to the reaction.

**Fig. 3:**
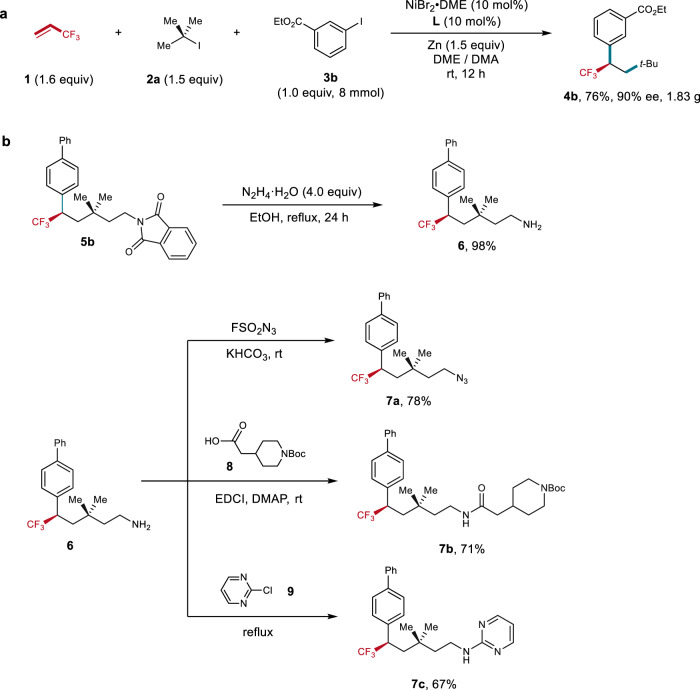
Synthetic applications. **a** Gram-scale synthesis of **4b**. **b** Transformations of compound **5b**. EDCI, (1-ethyl-3(3-dimethylpropylamine) carbodiimide.

To demonstrate the synthetic practicability of this protocol, gram-scale synthesis of **4b** was conducted (Fig. 3a), with even a higher yield (76%) and comparable ee obtained (90%). The resulting products can serve as a useful building block for various transformations. As illustrated in Fig. 3b, deprotection of the phthalimide group on compound **5b** with N_2_H_4_·H_2_O provided amine **6** in almost quantity yield. Compound **6** can be easily converted into alkyl azide **7a**^49^, a useful functional group for click chemistry. Condensation of **6** with carboxylic acid **8** led to amide **7b** efficiently. The reaction of 2-chloropyrimidine with **6** also proceeded smoothly, providing pyrimidine containing compound **7c** in good yield.

## Discussion

**Fig. 4:**
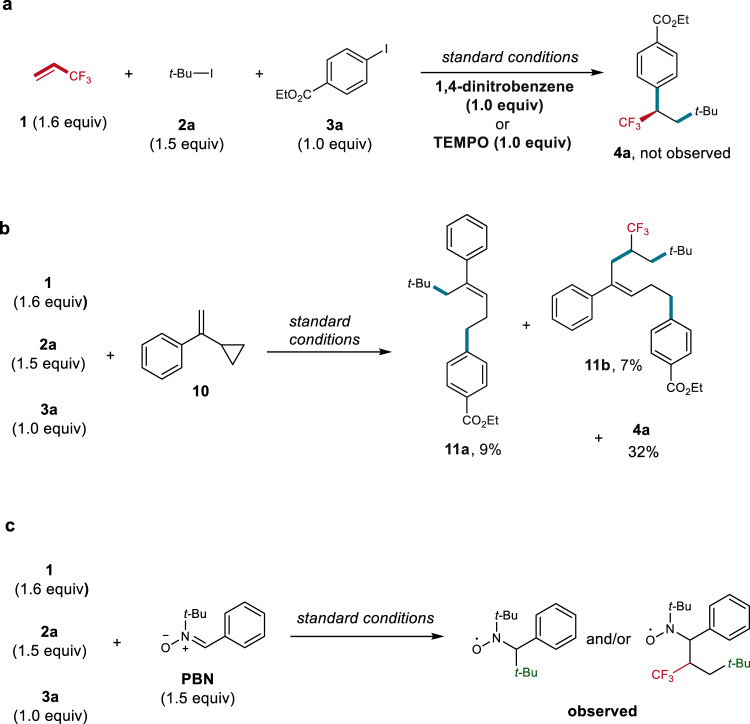
Radical trapping experiments. **a** Radical inhibition experiments. **b** Radical clock experiment. **c** EPR experiment.

**Fig. 5:**
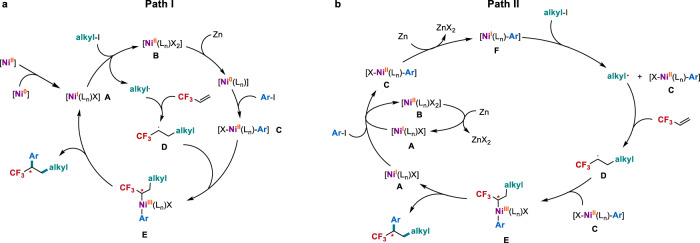
Proposed reaction mechanisms. **a** Possible mechanism I (path I). **b** Possible mechanism II (path II).

To gain mechanistic insight into the reaction, we conducted several radical trapping experiments (Fig. 4). The addition of an electron transfer inhibitor 1,4-dinitrobenzene or a radical scavenger TEMPO (2,2,6,6-tetramethylpiperidinooxy) to the reaction mixture of **1**, **2a**, and **3a** under standard reaction conditions totally shut down the reaction (Fig. 4a). Radical clock experiment by using α-cyclopropylstyrene^50^ **10** as a probe provided a mixture of ring-opening products **11a** and **11b** (Fig. 4b). These results clearly suggest that the alkyl radicals are involved in the reaction. This deduction was further supported by electron paramagnetic resonance (EPR) studies (Fig. 4c and Supplementary Figs. 5–7) with phenyl *tert*-butyl nitrone (PBN) as the spin trapping agent, in which the formation of spin adducts of the trapped *tert*-butyl radical and/or α-CF_3_ alkyl radical were detected. On the basis of these results and previous reports^51–56^, two plausible mechanisms were proposed (Fig. 5). Path I (Fig. 5a): The reaction begins with the formation of a nickel(I) species [Ni^I^L_n_X] (**A**)^54,55^. **A** subsequently reacts with tertiary alkyl iodide via a single electron transfer (SET) pathway to produce a tertiary alkyl radical and nickel(II) species [Ni^II^L_n_X_2_] (**B**). The resulting **B** is reduced by Zn to provide nickel(0) [Ni^0^(L_n_)], which undergoes oxidative addition with aryl iodide to generate arylnickel(II) complex [Ar-Ni^II^(L_n_)-X] (**C**). Meanwhile, the tertiary alkyl radical is trapped by TFP to form a new α-CF_3_ alkyl radical **D**. The combination of **D** with **C** produces the key intermediate nickel(III) complex **E**, in which the chiral ligand controls the stereoselectivity to form a highly reactive chiral **E**. Finally, **E** undergoes reductive elimination to deliver the chiral trifluoromethylated product and regenerate Ni(I) (**A**). Path II (Fig. 5b): A bimolecular oxidative addition of [Ni^I^L_n_X] (**A**) with aryl iodide initiates the reaction to provide arylnickel(II) complex **C**^56^, wherein complex **A** is generated by the reduction of nickel(II) complex **B** with zinc^56,57^. Subsequently, reduction of **C** with zinc provides arylnickel(I) complex [Ar-Ni^I^(L_n_)] (**F**), which reacts with tertiary alkyl iodide via a SET pathway to produce **C** and the tertiary alkyl radical. Radical addition of TFP delivers a new alkyl radical **D**. Combination of **D** with **C** provides the key intermediate **E**. Finally, reductive elimination of **E** affords the final product and regenerates **A**.

In conclusion, we have developed a highly enantioselective nickel-catalyzed alkyl-arylation of TFP with aryl and tertiary alkyl iodides. The combination of nickel catalyst with chiral Biox ligand renders the approach efficient to construct a variety of chiral trifluoromethylated compounds with high enantioselectivity. The protocol features synthetic convenience without laborious process to prepare organometallic reagents, as well as excellent functional group tolerance, even towards aryl bromide, boronate, and free alcohol, providing rapid and straightforward access to chiral trifluoromethylated compounds. In particular, the use of inexpensive industrial chemical TFP and successful suppression of its β-F elimination side reaction pave a new way to access chiral trifluoromethylated compounds of great interest in life and materials sciences. Preliminary mechanistic studies reveal that a radical tandem process is involved in the reaction.

## Methods

### General procedure for the enantioselective nickel-catalyzed alkyl-arylation of TFP

To a 25 mL of Schlenk tube were added Zn dust (1.5 equiv), tertiary alkyl iodide **2** (1.5 equiv), aryl iodide **3** (0.4 mmol, 1.0 equiv), **L6** (10 mol%), and NiBr_2_·DME (10 mol%) in a glovebox. The tube was then taken out of the glovebox and evacuated and backfilled with Ar (three times). Anhydrous DME (3.2 mL) and TFP solution (1.2 M in DMA, 0.54 mL, 1.6 equiv) were added under Ar. The Schlenk tube was screw capped and stirred (800 rpm) for 12 h at room temperature. The reaction mixture was then diluted with EtOAc and filtered through a pad of Celite. The filtrate was washed with Na_2_S_2_O_3_, water, and brine, the combined organic layers were dried over Na_2_SO_4_, filtered. The filtrate was concentrated. The residue was purified with silica gel chromatography to provide corresponding product **4**.

## Supplementary information

Supplementary Information

## Data Availability

The data that support the findings of this study are available within the paper and its supplementary information files. ^1^H, ^13^C, ^19^F NMR spectra, and mass spectrometry data are available in the Supplementary Information. The crystallographic data generated in this study have been deposited in the Cambridge Crystallographic Data Center database under accession code CCDC 2191969 (**4i**) and 2191970 (**5b**) (www.ccdc.cam.ac.uk/data_request/cif). Source data are provided with this paper.
