# Supplementary material for: Enantioselective nickel-catalyzed dicarbofunctionalization of 3,3,3-trifluoropropene
Source: Nat Commun. 2022 Sep 21;13:5539. doi: 10.1038/s41467-022-33159-2 (PMC9492779; doi:10.1038/s41467-022-33159-2)
Supplement: Supplementary file 1 — Supplementary Information [file 41467_2022_33159_MOESM1_ESM.pdf]

Supporting Information

Enantioselective Nickel-Catalyzed Dicarbofunctionalization of 3,3,3-Trifluoropropene

Yun-Ze Li[1], Na Rao[1], Lun An[1], Xiao-Long Wan[1], Yanxia Zhang[1], and Xingang Zhang*[1,2]

[1]Key Laboratory of Organofluorine Chemistry, Center for Excellence in Molecular Synthesis, Shanghai Institute of Organic Chemistry, University of Chinese Academy of Sciences, Chinese Academy of Sciences, 345 Lingling Road, Shanghai 200032, China

[2] Henan Institute of Advanced Technology, Zhengzhou University, Zhengzhou, 450001 P.R. China

*Corresponding author: xgzhang@mail.sioc.ac.cn

Table of Contents

I. Supplementary Notes……………..……………………………..……………………………………...……S3

1. General information and materials………………………………………………………………...………S3

II. Supplementary Methods ……………………………………………………………. ………………..……S4

2. Optimizations of the nickel-catalyzed dicarbofunctionalization of 3,3,3-trifluoropropene ……………….………S4

3. General procedure for the preparation of tertiary alkyl iodides and aryl iodides.……………………………..……S13

   3.1 General procedure for the preparation of tertiary alkyl iodides **2** ……………………...………………S13

   3.2 General procedure for the preparation of aryl iodides **3**…………..…………..…………………………S16

4. General procedure for the nickel-catalyzed enantioselective dicarbofunctionalization of TFP……….……..….S17

5. Characterization data for compounds **4** and **5**…………………………………………………………..…..S18

6.Transformations of compound **5b**………………………………………………………………..…………S39

III. Supplementary Discussion………………………………………………………………………………...S42

7. Mechanistic studies………………………………………………………………………………………...S42

   7.1 Radical inhibition experiments………………………………………………………………..……..S42

   7.2 Radical clock experiment………………………………………………………………………...….S42

   7.3 EPR experiments…………………………………………………..………………………...S44

8. Crystal data and structure refinement for compounds **4i** and **5b**………………………………………...……S47

IV. Supplementary Figures………………………………..…………………………...……………………S49

9. Copies of ^{1}H, ^{13}C, ^{19}F NMR and HPLC Spectra…………………………………...……………………S49

V. Supplementary References…………………………………………………………………………..…S174

I. Supplementary Notes

1. General information and materials

General Information: ^{1}H NMR and ^{13}C NMR spectra were recorded on Bruker AM 400, Agilent MR 400 and Agilent MR 500 spectrometer. ^{19}F NMR was recorded on the Agilent MR 400 spectrometer ($CFCl_3$ as an external standard and low field is positive). All ^{1}H NMR, ^{13}C NMR and ^{19}F NMR spectra were recorded at room temperature. The chemical shifts (δ) are given in parts per million (ppm) relative to $CDCl_3$ (7.26 ppm for ^{1}H) or TMS (0 ppm for ^{1}H) and $CDCl_3$ (77.0 ppm for ^{13}C), and coupling constants (J) are reported in Hertz (Hz). The following abbreviations were used to explain the multiplicities: s = singlet, d = doublet, t = triplet, dd = doublet of doublets, m = multiplet, br = broad. NMR yield was determined by ^{19}F NMR using benzotrifluoride as an internal standard before working up the reaction mixture. High performance liquid chromatography was performed on Waters 2487-600E, Waters ACQUITY UPC2, and Agilent Series HPLC, using PC-3, PC-4, AY-3, IC, IG, ADH, OJH, ODH, ASH chiral columns eluted with a mixture of acetonitrile/water or hexane/isopropyl alcohol. Optical rotation was measured on Rudolph-Autopol I and Autopol VI.

Materials: All reagents were used as received from commercial sources unless specified otherwise, or prepared according to literatures as described below. Anhydrous 1,4-dioxane (99.5%, extra dry, with molecular sieves, water ≤ 50 ppm (by K.F.)), 1,2-dimethoxyethane (DME, 99%, extra dry, with molecular sieves, water ≤ 50 ppm (by K.F.)) and *N*,*N*-dimethylacetamide (DMA, 99.8%, extra dry, with molecular sieves, water ≤ 50 ppm (by K.F.)) were purchased from Energy Chemical. $NiBr_2\cdot DME$ was purchased from Strem Chemicals, Inc.

II. Supplementary Methods

2. Optimizations of the nickel-catalyzed dicarbofunctionalization of 3,3,3-trifluoropropene

Supplementary Table 1. Ligand effect on the reaction of TFP with **2a** and **3a**[a]

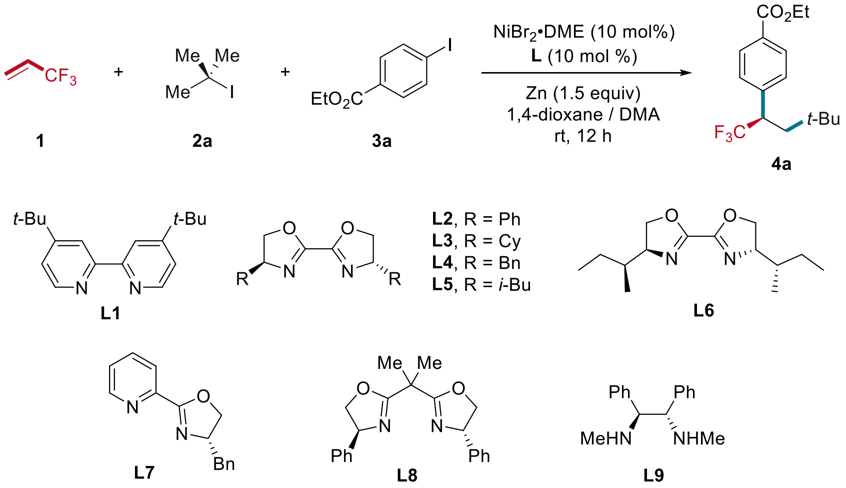

Entry	Ligand	**4a**, Yield (%)[b]	**4a**, ee (%)[c]
1	**L1**	84	-
2	**L2**	51	87
3	**L3**	66	90
4	**L4**	72	69
5	**L5**	61	63
6	**L6**	69	90
7	**L7**	52	13
8	**L8**	n.d.	-
9	**L9**	n.d.	-

[a]Reaction conditions (unless otherwise specified): **1** (0.64 mmol, 0.54 mL, 1.2 M in DMA, 1.6 equiv), **2a** (0.6 mmol, 1.5 equiv), **3a** (0.4 mmol, 1.0 equiv), 1,4-dioxane (3.2 mL). [b]Determined by ^{19}F NMR using benzotrifluoride as an internal standard. [c]Determined by chiral HPLC. n.d., not detected.

Supplementary Table 2. Solvent effect on the reaction of TFP with **2a** and **3a**[a]

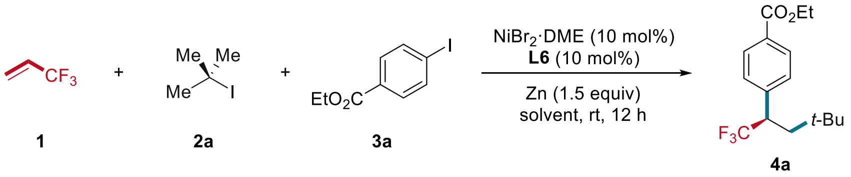

Entry	Solvent	**4a**, Yield (%)[b]	**4a**, ee (%)[c]
1	1,4-dioxane/DMA = 6:1	69	90
2	DME/DMA = 6:1	74 (70)	91
3	THF/DMA = 6:1	65	90
4	Diglyme/DMA = 6:1	76	90
5	Acetone/DMA = 6:1	70	86
6	EtOAc/DMA = 6:1	65	90
7	DME/DMA = 8:1	66	91
8	DME/DMA = 4:1	64	90
9	DME/DMA = 2:1	57	89.5
10	DME	6	-
11	DMA	32	86

[a]Reaction conditions (unless otherwise specified): **1** (0.64 mmol, 0.54 mL, 1.2 M in DMA, 1.6 equiv), **2a** (0.6 mmol, 1.5 equiv), **3a** (0.4 mmol, 1.0 equiv), solvent (3.2 mL). [b]Determined by ^{19}F NMR using benzotrifluoride as an internal standard, and number in parentheses is the isolated yield. [c]Determined by chiral HPLC.

Supplementary Table 3. Screening of the nickel sources for the reaction of TFP with **2a** and **3a**[a]

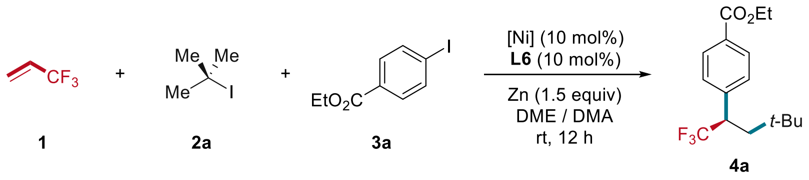

Entry	[Ni]	**4a**, Yield (%)[b]	**4a**, ee (%)[c]
1	$NiBr_2$·DME	74	91
2	$NiCl_2$·DME	70	91
3	$Ni(COD)_2$	70	91.5
4	$NiBr_2$·Diglyme	63	91
5	$NiCl_2$	n.d.	-
6	$NiBr_2$	n.d.	-
7	NiI_2	5	-
8	None	n.d.	-

[a]Reaction conditions (unless otherwise specified): **1** (0.64 mmol, 0.54 mL, 1.2 M in DMA, 1.6 equiv), **2a** (0.6 mmol, 1.5 equiv), **3a** (0.4 mmol, 1.0 equiv), DME (3.2 mL). [b]Determined by ^{19}F NMR using benzotrifluoride as an internal standard. [c]Determined by chiral HPLC.

Supplementary Table 4. Ligand effect on the reaction of TFP with **2b** and **3k**[a]

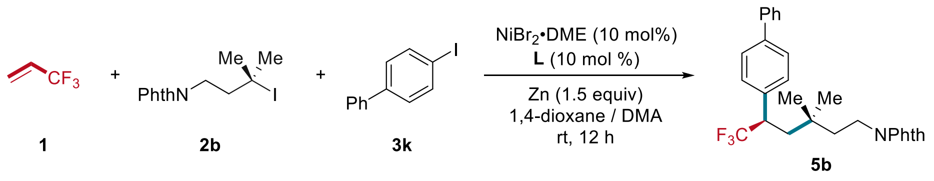

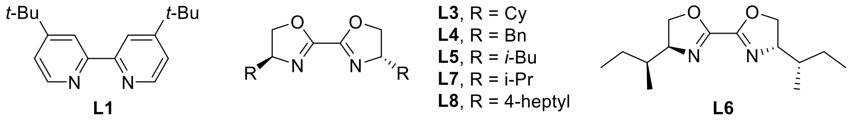

Entry	Ligand	**5b**, Yield (%)[b]	**5b**, ee (%)[c]
1	**L1**	44	-
2	**L3**	27	80
3	**L4**	41	53
4	**L5**	42	65
5	**L6**	36	87
6	**L7**	29	81
7	**L8**	11	93
8	None	trace	-

[a]Reaction conditions (unless otherwise specified): **1** (1.2 M in DMA; 0.27 mL, 0.32 mmol, 1.6 equiv), **2b** (0.3 mmol, 1.5 equiv), **3k** (0.2 mmol, 1.0 equiv), 1,4-dioxane (1.6 mL). [b]Determined by ^{19}F NMR using benzotrifluoride as an internal standard. [c]Determined by chiral HPLC.

Supplementary Table 5. Optimization of the loading amount of **2b**[a]

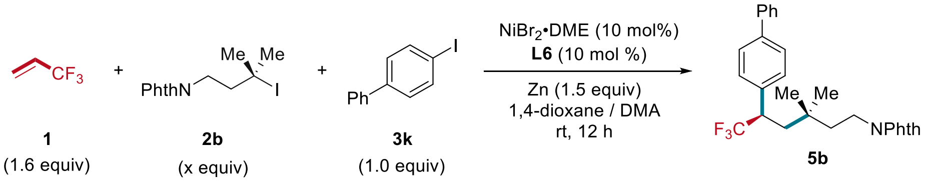

Entry	x	**5b**, Yield (%)[b]	**5b**, ee (%)[c]
1	1.2	29	-
2	1.5	36	-
3	2.0	27	-
4	2.5	31	-

[a]Reaction conditions (unless otherwise specified): **1** (1.2 M in DMA; 0.27 mL, 0.32 mmol, 1.6 equiv), **3k** (0.2 mmol, 1.0 equiv), 1,4-dioxane (1.6 mL). [b]Determined by ^{19}F NMR using benzotrifluoride as an internal standard. [c]Determined by chiral HPLC.

Supplementary Table 6. Optimization of the loading amount of nickel catalyst and ligand **L6**[a]

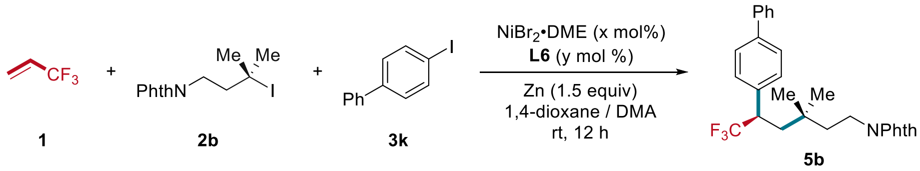

Entry	x	y	**5b**, Yield (%)[b]	**5b**, ee (%)[c]
1	10	10	36	87
2	12	10	46	89
3	14	10	41	89
4	20	10	33	-
5	15	7.5	35	-

[a]Reaction conditions (unless otherwise specified): **1** (1.2 M in DMA; 0.27 mL, 0.32 mmol, 1.6 equiv), **2b** (0.3 mmol, 1.5 equiv), **3k** (0.2 mmol, 1.0 equiv), 1,4-dioxane (1.6 mL). [b]Determined by ^{19}F NMR using benzotrifluoride as an internal standard. [c]Determined by chiral HPLC.

Supplementary Table 7. Screening of the nickel sources for the reaction of TFP with **2b** and **3k**[a]

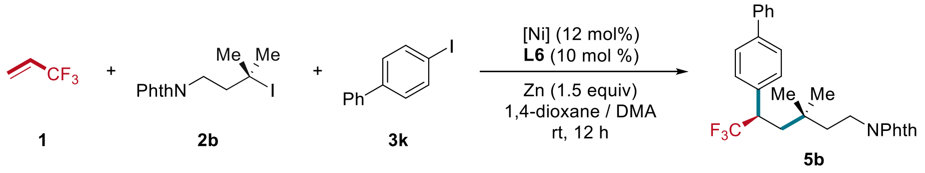

Entry	[Ni]	**5b**, Yield (%)[b]	**5b**, ee (%)[c]
1	$NiBr_2$·DME	46	89
2	$NiCl_2$·DME	37	-
3	NiI_2	39	-
4	$NiBr_2$·Diglyme	33	-
5	$NiBr_2(PPh_3)_2$	0	-
6	$NiCl_2(PCy_3)_2$	6	-

[a]Reaction conditions (unless otherwise specified): **1** (1.2 M in DMA; 0.27 mL, 0.32 mmol, 1.6 equiv), **2b** (0.3 mmol, 1.5 equiv), **3k** (0.2 mmol, 1.0 equiv), 1,4-dioxane (1.6 mL). [b]Determined by ^{19}F NMR using benzotrifluoride as an internal standard. [c]Determined by chiral HPLC.

Supplementary Table 8. Optimization of the solvent for the reaction of TFP with **2b** and **3k**[a]

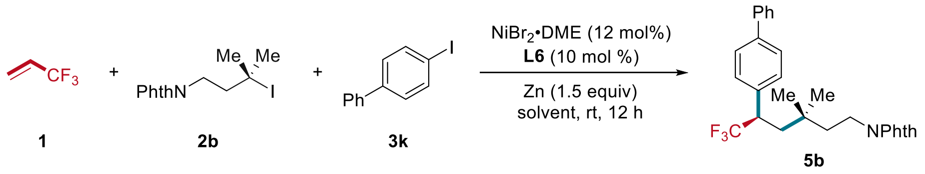

Entry	Solvent	**5b**, Yield (%)[b]	**5b**, ee (%)[c]
1	1,4-dioxane/DMA = 6:1	46	89
2	DME/DMA = 6:1	52	87
3	THF/DMA = 6:1	29	--
4	Acetone/DMA = 6:1	30	--
5	1,4-dioxane/DMA = 3:1	42	--
6	1,4-dioxane/DMA = 1:1	16	--
7	DMA	trace	--

[a]Reaction conditions (unless otherwise specified): **1** (1.2 M in DMA; 0.27 mL, 0.32 mmol, 1.6 equiv), **2b** (0.3 mmol, 1.5 equiv), **3k** (0.2 mmol, 1.0 equiv), solvent (1.6 mL). [b]Determined by ^{19}F NMR using benzotrifluoride as an internal standard. [c]Determined by chiral HPLC.

Supplementary Table 9. Additive effect on the reaction of TFP with **2b** and **3k**[a]

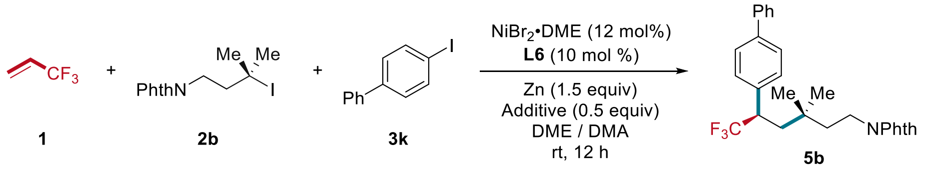

Entry	Additive	**5b**, Yield (%)[b]	**5b**, ee (%)[c]
1	None	52	87
2	TMSCl	26	--
3	TBAI	47	--
4	NaI	58	--
5	$FeCl_3$	67	87
6	LiI	48	--
7	ZnI_2	41	--
8	$MgBr_2$	42	--
9	3 Å MS (150 mg)	31	--
10	4 Å MS (150 mg)	39	--

[a]Reaction conditions (unless otherwise specified): **1** (1.2 M in DMA; 0.27 mL, 0.32 mmol, 1.6 equiv), **2b** (0.3 mmol, 1.5 equiv), **3k** (0.2 mmol, 1.0 equiv), DME (1.6 mL). [b]Determined by ^{19}F NMR using benzotrifluoride as an internal standard. [c]Determined by chiral HPLC.

Supplementary Table 10. Optimization of the loading amount of additive[a]

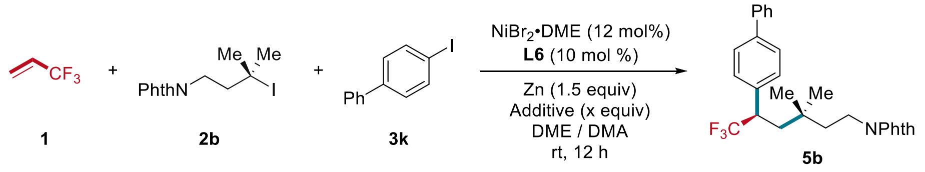

Entry	Additive	**5b**, Yield (%)[b]	**5b**, ee (%)[c]
1	NaI (0.25 equiv)	54	--
2	NaI (0.5 equiv)	58	--
3	NaI (1.0 equiv)	58	--
4	$FeCl_3$ (0.25 equiv)	70	87
5	$FeCl_3$ (0.5 equiv)	67	87
6	$FeCl_3$ (1.0 equiv)	41	--

[a]Reaction conditions (unless otherwise specified): **1** (1.2 M in DMA; 0.27 mL, 0.32 mmol, 1.6 equiv), **2b** (0.3 mmol, 1.5 equiv), **3k** (0.2 mmol, 1.0 equiv), DME (1.6 mL). [b]Determined by ^{19}F NMR using benzotrifluoride as an internal standard. [c]Determined by chiral HPLC.

3. General procedure for the preparation of tertiary alkyl and aryl iodides.

3.1 General procedure for the preparation of tertiary alkyl iodides 2[1]

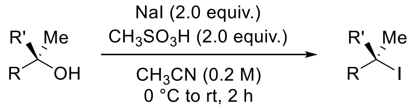

To a mixture of NaI (2.0 equiv) and corresponding tertiary alcohol (1.0 equiv) in MeCN (0.2 M) was added methylsulfonic acid (2.0 equiv) dropwise at 0 °C. The reaction mixture was allowed to warm to room temperature and stirred for 2 hours. The reaction mixture was then diluted with diethyl ether, washed with water, saturated aqueous $NaHCO_3$, aqueous $Na_2S_2O_3$, and brine. The organic layers was dried over Na_2SO_4, filtered, and concentrated. The crude product was purified by flash chromatography on silica gel or recrystallization from petroleum ether/EtOAc to afford the corresponding tertiary alkyl iodide **2**.

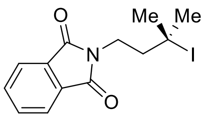

2b

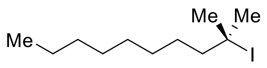

2c

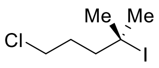

2d

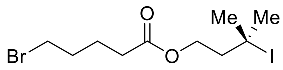

2e

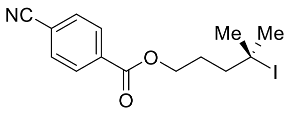

2f

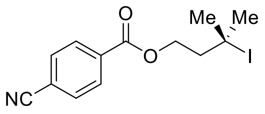

2g

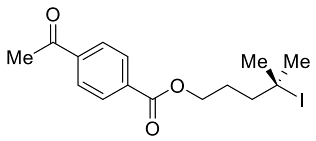

2h

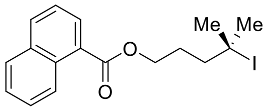

2i

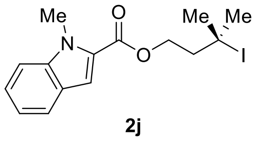

2j

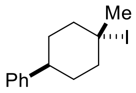

2k

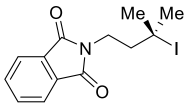

2-(3-iodo-3-methylbutyl)isoindoline-1,3-dione (2b). This compound was synthesized from the iodination of 2-(3-hydroxy-3-methylbutyl)isoindoline-1,3-dione in 31% yield as a white solid (recrystallization from PE/EA). Compound **2b** is known.[2] **^{1}H NMR** (400 MHz, $CDCl_3$) δ 7.86 (dd, J = 5.5, 3.0 Hz, 2 H), 7.72 (dd, J = 5.4, 3.0 Hz, 2 H), 3.97 – 3.87 (m, 2 H), 2.02 (m, 8 H). **^{13}C NMR** (126 MHz, $CDCl_3$) δ 168.1, 133.9, 132.0, 123.2, 47.5, 45.2, 38.0, 37.7.

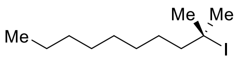

2-iodo-2-methyldecane (2c). This compound was synthesized from the iodination of 2-methyldecan-2-ol in 30% yield as a yellow oil. Compound **2c** is known.[3] **^{1}H NMR** (400 MHz, $CDCl_3$) δ 1.92 (s, 6 H), 1.64 – 1.58 (m, 2 H), 1.55 – 1.45 (m, 2 H), 1.37 – 1.22 (m, 10 H), 0.89 (t, J = 6.8 Hz, 3 H).

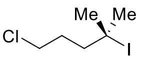

1-chloro-4-iodo-4-methylpentane (2d). This compound was synthesized from the iodination of 5-chloro-2-methylpentan-2-ol in 42% yield as a brown oil. Compound **2d** is known.[4] **^{1}H NMR** (400 MHz, $CDCl_3$) δ 3.60 (t, J = 6.4 Hz, 2 H), 2.09 – 1.99 (m, 2 H), 1.94 (s, 6 H), 1.80 – 1.69 (m, 2 H). **^{13}C NMR** (101 MHz, $CDCl_3$) δ 50.2, 47.6, 44.5, 38.1, 31.9.

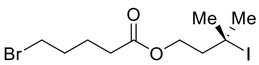

3-Iodo-3-methylbutyl 5-bromopentanoate (2e). This compound was synthesized from the iodination of 3-hydroxy-3-methylbutyl 5-bromopentanoate in 57% yield as a yellow oil. **^{1}H NMR** (400 MHz, $CDCl_3$) δ 4.31 (t, J = 7.0 Hz, 2 H), 3.41 (t, J = 6.7 Hz, 2 H), 2.33 (t, J = 7.0 Hz, 2 H), 2.02 (t, J = 7.0 Hz, 2 H), 1.97 (s, 6 H), 1.93 – 1.83 (m, 2 H), 1.72 – 1.61 (m, 2 H), 1.53 – 1.44 (m, 2 H). **^{13}C NMR** (101 MHz, $CDCl_3$) δ 173.2, 64.4, 47.9, 46.0, 38.4, 34.0, 33.4, 32.3, 27.5, 23.9. MS (DART): m/z (%) 408.0 (100) $[M+NH_4]^+$. HRMS (DART) m/z: $[M+H]^+$ Calcd. for $C_{11}H_{21}BrIO_2$: 390.9764; Found: 390.9763. IR (neat) ν_{max} 2959, 1735, 1455, 1180, 1123 cm^{-1}.

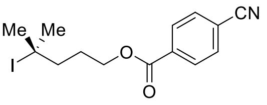

4-Iodo-4-methylpentyl 4-cyanobenzoate (2f). This compound was synthesized from the iodination of 4-hydroxy-4-methylpentyl 4-cyanobenzoate in 53% yield as a white solid (m.p. 60.1-61.8 °C). **^{1}H NMR** (400 MHz, $CDCl_3$) δ 8.15 (d, J = 8.2 Hz, 2 H), 7.76 (d, J = 8.2 Hz, 2 H), 4.41 (t, J = 6.6 Hz, 2 H), 2.09 – 2.01 (m, 2 H), 1.96 (s, 6 H), 1.78 – 1.71 (m, 2 H). **^{13}C NMR** (126 MHz, $CDCl_3$) δ 164.9, 134.0, 132.2, 130.0, 117.9, 116.3, 65.2, 50.3, 46.6, 38.0, 25.6. MS (FT): m/z (%) 248 (100), 358 (24) $[M+H]^+$. HRMS (DART) m/z: $[M+H]^+$ Calcd. for $C_{14}H_{17}NO_2I$: 358.0298; Found: 358.0296. IR (thin film) ν_{max} 2971, 2229, 1724, 1451, 1367, 1310, 1287, 1125, 1110, 813 cm^{-1}.

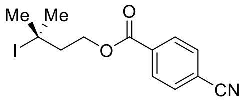

3-Iodo-3-methylbutyl 4-cyanobenzoate (2g). This compound was synthesized from the iodination of 3-hydroxy-3-methylbutyl 4-cyanobenzoate in 59% yield as a white solid (m.p. 62.0-63.0 °C). **^{1}H NMR** (400 MHz, $CDCl_3$) δ 8.14 (d, J = 8.3 Hz, 2 H), 7.75 (d, J = 8.3 Hz, 2 H), 4.61 (t, J = 6.8 Hz, 2 H), 2.18 (t, J = 6.8 Hz, 2 H), 2.03 (s, 6 H).**^{13}C NMR** (126 MHz, $CDCl_3$) δ 164.7, 133.8, 132.2, 130.0, 117.9, 116.4, 65.8, 47.9, 45.3, 38.4. MS (FI): m/z (%) 216 [M-HI]$^+$, 130, 69 (100). HRMS (FI) m/z: [M-HI]$^+$ Calcd. for $C_{13}H_{13}NO_2$: 215.0941; Found: 215.0940. IR (thin film) ν_{max} 2978, 2228, 1721, 1470, 1404, 1324, 1273, 767 cm^{-1}.

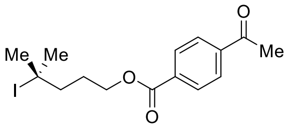

4-Iodo-4-methylpentyl 4-acetylbenzoate (2h). This compound was synthesized from the iodination of 4-hydroxy-4-methylpentyl 4-acetylbenzoate in 55% yield as a yellow solid (m.p. 37.8-39.5 °C). **^{1}H NMR** (400 MHz, $CDCl_3$) δ 8.14 (d, J = 8.3 Hz, 2 H), 8.02 (d, J = 8.2 Hz, 2 H), 4.41 (t, J = 6.5 Hz, 2 H), 2.65 (s, 3 H), 2.09 – 2.00 (m, 2 H), 1.97 (s, 6 H), 1.82 – 1.72 (m, 2 H). **^{13}C NMR** (101 MHz, $CDCl_3$) δ 197.5, 165.7, 140.3, 134.0, 129.8, 128.2, 64.9, 50.4, 46.7, 38.0, 28.2, 26.9. MS (FT): m/z (%) 247 (100) [M-HI]$^+$, 375 (18) [M+H]$^+$. HRMS (DART) m/z: [M+H]$^+$ Calcd. for $C_{15}H_{20}O_3I$: 375.0452; Found: 375.0449. IR (thin film) ν_{max} 3408, 2971, 1720, 1686, 1405, 1281, 1114, 769 cm^{-1}.

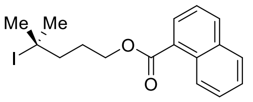

4-Iodo-4-methylpentyl 1-naphthoate (2i). This compound was synthesized from the iodination of 4-hydroxy-4-methylpentyl 1-naphthoate in 32% yield according to the literature method as a yellow solid (m.p. 46.6-48.5 °C). **^{1}H NMR** (400 MHz, $CDCl_3$) δ 8.92 (d, J = 8.6 Hz, 1 H), 8.20 (d, J = 7.3 Hz, 1 H), 8.04 (d, J = 8.1 Hz, 1 H), 7.90 (d, J = 8.3 Hz, 1 H), 7.66 – 7.59 (m, 1 H), 7.58 – 7.48 (m, 2 H), 4.47 (t, J = 6.4 Hz, 2 H), 2.15 – 2.04 (m, 2 H), 1.98 (s, 6 H), 1.86 – 1.77 (m, 2 H). **^{13}C NMR** (101 MHz, $CDCl_3$) δ 167.5, 133.8, 133.6, 131.3, 130.1, 128.5, 127.7, 127.1, 126.2, 125.8, 124.5, 64.5, 50.6, 46.9, 38.0, 28.3. MS (FT): m/z (%) 400 (100) [M+NH_4]$^+$. HRMS (DART) m/z: [M+NH_4]$^+$ Calcd. for $C_{17}H_{15}NO_2I$: 400.0768; Found: 400.0763. IR (thin film) ν_{max} 2982, 2893, 1713, 1507, 1461, 1386, 1287, 1164, 782 cm^{-1}.

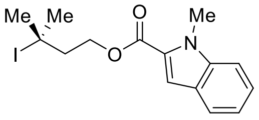

3-Iodo-3-methylbutyl 1-methyl-1*H*-indole-2-carboxylate (2j). This compound was synthesized from the iodination of 3-hydroxy-3-methylbutyl 1-methyl-1*H*-indole-2-carboxylate in 28% yield as a white solid (m.p. 59.8-60.9 °C). **^{1}H NMR** (400 MHz, $CDCl_3$) δ 7.68 (d, *J* = 7.9 Hz, 1 H), 7.43 – 7.32 (q, *J* = 8.4 Hz, 2 H), 7.28 (s, 1 H), 7.16 (t, *J* = 7.2 Hz, 1 H), 4.56 (t, *J* = 6.7 Hz, 2 H), 4.09 (s, 3 H), 2.19 (t, *J* = 6.8 Hz, 2 H). 2.05 (s, 6 H). **^{13}C NMR** (101 MHz, $CDCl_3$) δ 161.9, 139.6, 127.5, 125.7, 125.0, 122.5, 120.6, 110.2, 110.2, 64.6, 48.1, 46.3, 38.5, 31.5. MS (FT): m/z (%) 372 (100) $[M+H]^+$. HRMS (DART) m/z: $[M+H]^+$ Calcd. for $C_{15}H_{19}NO_2I$: 372.0455; Found: 372.0448. IR (thin film) ν_{max} 2979, 1700, 1526, 1466, 1359, 1259, 1123, 751 cm^{-1}.

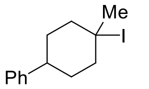

4-Iodo-4-methylcyclohexyl)benzene (2k). This compound was synthesized from the iodination of 1-methyl-4-phenylcyclohexan-1-ol in 86% yield as a yellow solid (m.p. 88.9-90.2 °C). **^{1}H NMR** (400 MHz, $CDCl_3$) δ 7.36 – 7.25 (m, 4 H), 7.24 – 7.18 (m, 1 H), 2.51 (tt, *J* = 12.4, 3.9 Hz, 1 H), 2.29 – 2.21 (m, 2 H), 2.18 (s, 3 H), 2.08 – 1.96 (m, 2 H), 1.92 – 1.82 (m, 2 H), 1.13 – 1.01 (m, 2 H). **^{13}C NMR** (126 MHz, $CDCl_3$) δ 146.5, 128.4, 126.9, 126.2, 58.5, 45.8, 43.5, 39.4, 32.7. MS (EI): m/z (%) 173 (100) $[M-I]^+$, 300 (4) $[M]^+$. HRMS (EI) m/z: $[M]^+$ Calcd. for $C_{13}H_{17}I$: 300.0369; Found: 300.0376. IR (thin film) ν_{max} 3056, 2968, 2910, 1491, 1442, 1298, 1224, 1107, 757 cm^{-1}.

3.2 General procedure for the preparation of aryl iodides 3.

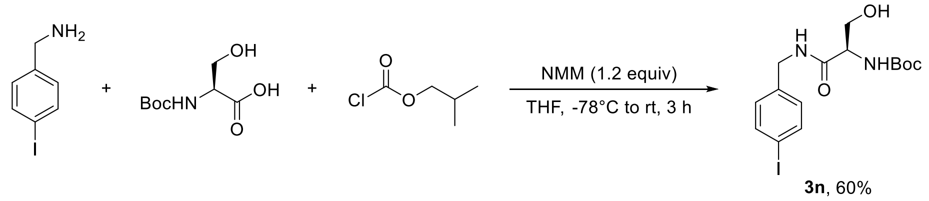

3n, 60%

***tert*-Butyl (*R*)-(3-hydroxy-1-((4-iodobenzyl)amino)-1-oxopropan-2-yl)carbamate** (**3n**). Under Ar atmosphere, (*tert*-butoxycarbonyl)-*L*-serine (2.05 g, 10 mmol, 1.0 equiv) and anhydrous THF (80 mL) were added to a 250 mL of Schlenk flask. After cooling down to -78 °C, 4-methylmorpholine (1.22 g, 12 mmol, 1.2 equiv) was added and the mixture was stirred at same temperature for 2 minutes. Isobutyl carbonochloridate was added, and the mixture was stirred at -78 °C for another 5 minutes. (4-Iodophenyl)methanamine was then added dropwise, and the resulting solution was slowly warmed to room temperature and stirred for additional 3 h. Upon completion, the reaction mixture was filtered

through a pad of Celite and washed with EtOAc. After removal of solvents, the crude product was recrystallized from EtOAc to give **3n** (2.54 g, 60%) as a white crystal (m.p. 125.8-127.4 °C). The spectral data was in accordance with the literature.[5]

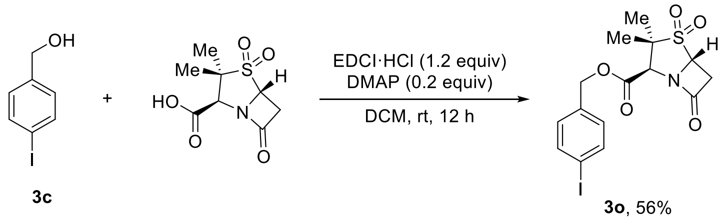

3c **3o**, 56%

4-Iodobenzyl (2*S*, 5*R*)-3,3-dimethyl-7-oxo-4-thia-1-azabicyclo[3.2.0]heptane-2-carboxylate 4,4-dioxide (**3o**). To a 50 mL of Schlenk flask were added (4-iodophenyl)methanol **3c** (0.936 g, 4 mmol, 2.0 equiv), 4-(dimethylamino)pyridine (48.9 mg, 0.4 mmol, 0.2 equiv), *N*-(3-dimethylaminopropyl)-*N'*-ethylcarbodiimide hydrochloride (EDCI·HCl) (0.767 g, 2.4 mmol, 1.2 equiv), (2*S*,5*R*)-3,3-dimethyl-7-oxo-4-thia-1-azabicyclo[3.2.0]heptane-2-carboxylic acid 4,4-dioxide (0.467 g, 2 mmol, 1.0 equiv), and anhydrous CH_2Cl_2 (20 mL) under Ar atmosphere. After the mixture was stirred at room temperature for 12 h, the reaction mixture was diluted with CH_2Cl_2 and washed with water and brine. The organic layer was dried over anhydrous Na_2SO_4, filtered, and concentrated. The product **3o** (0.50 g, 56% yield) was purified with silica gel chromatography (Petroleum ether/EtOAc = 10:1 to 4:1) as a white solid (m.p. 49.0-50.9 °C). **^{1}H NMR** (400 MHz, $CDCl_3$) δ 7.73 (d, J = 8.1 Hz, 2 H), 7.12 (d, J = 8.1 Hz, 2 H), 5.21 (d, J = 12.1 Hz, 1 H), 5.11 (d, J = 12.1 Hz, 1 H), 4.60 (dd, J = 4.2, 2.2 Hz, 1 H), 4.41 (s, 1 H), 3.63 – 3.36 (m, 2 H), 1.56 (s, 3 H), 1.30 (s, 3 H). **^{13}C NMR** (101 MHz, $CDCl_3$) δ 170.7, 166.7, 138.0, 133.9, 130.6, 94.9, 67.4, 63.1, 62.7, 61.0, 38.3, 20.2, 18.6. MS (ESI): m/z (%) 472 $[M+Na]^+$. HRMS (DART) m/z: $[M+H]^+$ Calcd. for $C_{15}H_{16}NO_5NaSI$: 471.9686; Found: 471.9678. IR (thin film) ν_{max} 2977, 2933, 1791, 1759, 1486, 1356, 1166 cm^{-1}.

4. General procedure for the nickel-catalyzed enantioselective dicarbofunctionalization of TFP

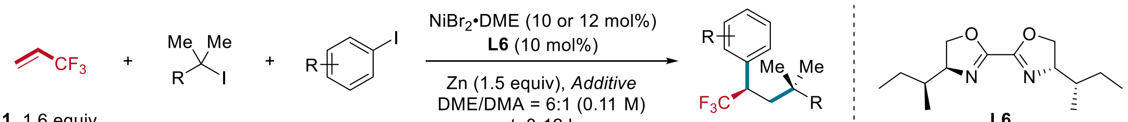

1, 1.6 equiv 1.2 M in DMA; **2**, 1.5 equiv; **3**, 1.0 equiv; **4** or **5**; **L6**

General Procedure A:

To a 25 mL of Schlenk tube were added Zn dust (1.5 equiv), tertiary alkyl iodide **2** (1.5 equiv), aryl iodide **3** (0.4 mmol, 1.0 equiv), **L6** (10 mol%) and $NiBr_2$·DME (10 mol%) in a glovebox. The tube

was then taken out of the glovebox and evacuated and backfilled with Ar (3 times). Anhydrous DME (3.2 mL) and TFP solution (1.2 M in DMA, 0.54 mL, 1.6 equiv) were added under Ar. The Schlenk tube was screw capped and stirred (800 rpm) for 12 h at room temperature. The reaction mixture was then diluted with EtOAc and filtered through a pad of Celite. The filtrate was washed with $Na_2S_2O_3$, water and brine, the combined organic layers were dried over Na_2SO_4, filtered and concentrated. The residue was purified with silica gel chromatography to give the corresponding product **4**.

General Procedure B:

To a 25 mL of Schlenk tube were added Zn dust (1.5 equiv), tertiary alkyl iodide **2** (1.5 equiv), aryl iodide **3** (0.2 mmol, 1.0 equiv), **L6** (10 mol%), $NiBr_2$·DME (10 or 12mol%) and additive ($FeCl_3$ (0.25 equiv), $FeBr_2$ (0.25 equiv) or NaI (0.5 equiv)) in a glovebox. The tube was then taken out of the glovebox, evacuated and backfilled with Ar (3 times). Anhydrous DME (1.6 mL) and TFP solution (1.2 M in DMA, 0.27 mL, 1.6 equiv) were added under Ar. The Schlenk tube was screw capped and stirred (800 rpm) for 12 h at room temperature. The reaction mixture was then diluted with EtOAc and filtered through a pad of Celite. The filtrate was washed with $Na_2S_2O_3$, water and brine, the combined organic layers were dried over Na_2SO_4, filtered and concentrated. The residue was purified with silica gel chromatography to give the corresponding product **5**.

5. Characterization data for compounds 4 and 5

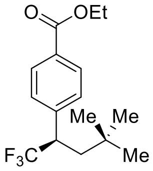

Ethyl (*R*)-4-(1,1,1-trifluoro-4,4-dimethylpentan-2-yl)benzoate (4a). General Procedure A. The product (84.5 mg, 70% yield) was purified with silica gel chromatography (Petroleum ether/EtOAc = 30:1) as a white solid (m.p. 44.5-45.0 ^{o}C). $[\alpha]_D^{20}$ = -22.60 (*c* = 0.97, $CHCl_3$) for a sample with 91% ee. **^{1}H NMR** (400 MHz, $CDCl_3$) δ 8.03 (d, *J* = 8.4 Hz, 2 H), 7.42 (d, *J* = 8.1 Hz, 2 H), 4.38 (q, *J* = 7.1 Hz, 2 H), 3.40 (qt, *J* = 9.6, 5.8 Hz, 1 H), 1.94 (d, *J* = 6.0 Hz, 1 H), 1.39 (t, *J* = 7.1 Hz, 3 H), 0.79 (s, 9 H). **^{13}C NMR** (101 MHz, $CDCl_3$) δ 166.2, 141.8 (q, C-F, $^3J_{C-F}$ = 2.1 Hz), 130.3, 129.8, 129.4, 126.9 (q, C-F, $^1J_{C-F}$ = 279.6 Hz), 61.0, 47.1 (q, C-F, $^2J_{C-F}$ = 26.4 Hz), 42.1, 30.8, 29.6, 14.3. **^{19}F NMR** (376 MHz, $CDCl_3$) δ -70.11 (d, *J* = 9.9 Hz, 3 F). MS (EI): m/z (%) 57 (100), 246, 257, 302 $[M]^+$. HRMS (EI) m/z: $[M]^+$ Calcd. for $C_{16}H_{21}O_2F_3$:

302.1488; Found: 302.1483. IR (thin film): ν_{max} 2991, 2959, 1717, 1510, 1451, 1369, 1282, 1106, 716 cm^{-1}. Enantiomeric purity (91% ee) was measured by chiral HPLC on IG column (Hexane/^{i}PrOH = 98:2, 0.7 mL/min, UV detection at 214 nm); retention time = 7.51 min (major), retention time = 7.22 min (minor).

Using ethyl 4-bromobenzoate instead of ethyl 4-iodobenzoate gives corresponding product **4a** with 40% yield and 90% ee. Enantiomeric purity (90% ee) was measured by chiral HPLC on IG column (Hexane/^{i}PrOH = 98:2, 0.7 mL/min, UV detection at 214 nm); retention time = 7.52 min (major), retention time = 7.19 min (minor).

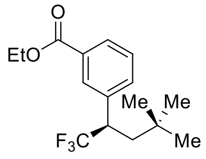

Ethyl (*R*)-3-(1,1,1-trifluoro-4,4-dimethylpentan-2-yl)benzoate (4b). General Procedure A. The product (84.0 mg, 70% yield) was purified with silica gel chromatography (Petroleum ether/EtOAc = 30:1) as a colorless liquid. $[\alpha]_D^{20}$ = -18.34 (c = 0.41, $CHCl_3$) for a sample with 92% ee. **^{1}H NMR** (400 MHz, $CDCl_3$) δ 8.04 – 7.97 (m, 1 H), 7.53 (d, J = 7.7 Hz, 2 H), 7.47 – 7.39 (m, 2 H), 4.39 (q, J = 7.1 Hz, 2 H), 3.40 (dtd, J = 16.7, 9.8, 5.2 Hz, 1 H), 2.01 – 1.90 (m, 2 H), 1.41 (t, J = 7.2 Hz, 3 H), 0.80 (s, 9 H). **^{13}C NMR** (101 MHz, $CDCl_3$) δ 166.2, 137.3 (q, C-F, $^3J_{C-F}$ = 2.0 Hz), 133.6, 130.9, 130.6, 129.2, 128.6, 127.0 (q, C-F, $^1J_{C-F}$ = 279.8 Hz), 61.1, 46.9 (q, C-F, $^2J_{C-F}$ = 26.3 Hz), 42.0 (q, C-F, $^3J_{C-F}$ = 1.4 Hz), 30.8, 29.7, 14.3. **^{19}F NMR** (376 MHz, $CDCl_3$) δ -70.32 (d, J = 9.7 Hz, 3 F). MS (EI): m/z (%) 57 (100), 246, 257, 302 $[M]^+$. HRMS (EI) m/z: $[M]^+$ Calcd. for $C_{16}H_{21}O_2F_3$: 302.1488; Found: 302.1483. IR (thin film) ν_{max} 2960, 2870, 1720, 1477, 1398, 1284, 1260, 1108, 712 cm^{-1}. Enantiomeric purity (92% ee) was measured by chiral HPLC on ODH column (Hexane/^{i}PrOH = 98:2, 0.7 mL/min, UV detection at 214 nm); retention time = 5.55 min (major), retention time = 6.11 min (minor).

Gram-scale synthesis of 4b

To a 100 mL of Schlenk tube were added Zn dust (784.6 mg, 12 mmol, 1.5 equiv), **L6** (201.9 mg, 0.8 mmol, 10 mol%). The tube was then transferred to a glovebox, $NiBr_2$·DME (246.9 mg, 0.8 mmol, 10 mol%) was added. The tube was taken out of the glovebox and evacuated and backfilled with Ar (3 times). **2a** (2.21 g, 12 mmol, 1.5 equiv), **3b** (2.14 g, 8 mmol, 1.0 equiv), anhydrous DME (64 mL) and TFP solution (10.7 mL, 1.2 M in DMA, 1.6 equiv) were added under Ar at 0 °C. The Schlenk tube was screw capped and stirred (800 rpm) for 8 h at room temperature. The reaction mixture was then diluted

with EtOAc and filtered through a pad of Celite. The filtrate was washed with $Na_2S_2O_3$, water and brine, the combined organic layers were dried over Na_2SO_4, filtered and concentrated. The residue was purified with silica gel chromatography to give **4b** as a colorless oil (1.83 g, 76%, 90% ee).

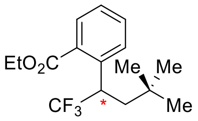

Ethyl 2-(1,1,1-trifluoro-4,4-dimethylpentan-2-yl)benzoate (4c). General Procedure A. The product (25.2 mg, 21% yield) was purified with silica gel chromatography (Petroleum ether/DCM = 5:1) as a colorless liquid. $[\alpha]_D^{20}$ = -0.65 (*c* = 0.99, $CHCl_3$). **^{1}H NMR** (400 MHz, $CDCl_3$) δ 7.90 (dd, *J* = 7.8, 1.6 Hz, 1 H), 7.61 – 7.56 (m, 1 H), 7.52 (td, *J* = 7.7, 1.6 Hz, 1 H), 7.39 – 7.33 (m, 1 H), 5.10 – 4.96 (m, 1 H), 4.39 (qd, *J* = 7.1, 1.4 Hz, 2 H), 2.03 – 1.92 (m, 2 H), 1.40 (t, *J* = 7.1 Hz, 3 H), 0.81 (s, 9 H). **^{13}C NMR** (101 MHz, $CDCl_3$) δ 167.7, 131.7, 131.7, 130.7, 129.2, 127.5, 127.3 (q, C-F, $^1J_{C\text{-}F}$ = 279.8 Hz), 61.3, 42.7, 39.7 (q, C-F, $^2J_{C\text{-}F}$ = 26.2 Hz), 31.0, 29.6, 14.2. **^{19}F NMR** (376 MHz, $CDCl_3$) δ -69.80 (d, *J* = 10.9 Hz). MS (FI): m/z (%) 206, 282 (100), 302 $[M]^+$. HRMS (FI) m/z: $[M]^+$ Calcd. for $C_{16}H_{21}O_2F_3$: 302.1488; Found: 302.1486. IR (thin film) ν_{max} 2956, 2869, 1718, 1466, 1398, 1298, 1247, 1176, 1108, 719 cm^{-1}.

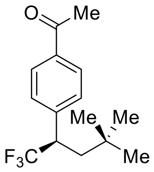

(*R*)-1-(4-(1,1,1-Trifluoro-4,4-dimethylpentan-2-yl)phenyl)ethan-1-one (4d). General Procedure A. The product (79.1 mg, 73% yield) was purified with silica gel chromatography (Petroleum ether/EtOAc = 20:1) as a white solid (m.p. 48.0-48.9 ^oC). $[\alpha]_D^{20}$ = -30.51 (*c* = 0.47, $CHCl_3$) for a sample with 92% ee. **^{1}H NMR** (400 MHz, $CDCl_3$) δ 7.94 (d, *J* = 8.4 Hz, 2 H), 7.44 (d, *J* = 8.0 Hz, 2 H), 3.40 (dtd, *J* = 11.5, 9.6, 5.5 Hz, 1 H), 2.61 (s, 3 H), 2.02 – 1.88 (m, 2 H), 0.80 (s, 9 H). **^{13}C NMR** (101 MHz, $CDCl_3$) δ 197.5, 142.1 (q, C-F, $^3J_{C\text{-}F}$ = 2.2 Hz), 136.8, 129.7, 128.5, 126.9 (q, C-F, $^1J_{C\text{-}F}$ = 279.9 Hz), 47.1 (q, C-F, $^2J_{C\text{-}F}$ = 26.4 Hz), 42.1 (q, C-F, $^3J_{C\text{-}F}$ = 1.7 Hz), 30.8, 29.7, 26.6. **^{19}F NMR** (376 MHz, $CDCl_3$) δ -70.05 (d, *J* = 9.7 Hz, 3 F). MS (EI): m/z (%) 257 (100), 272 $[M]^+$. HRMS (EI) m/z: $[M]^+$ Calcd. for $C_{15}H_{19}OF_3$: 272.1383; Found: 272.1379. IR (thin film) ν_{max} 2963, 2870, 1610, 1479, 1468, 1371, 1258, 1174, 1099, 827, 705 cm^{-1}. Enantiomeric purity (92% ee) was measured by chiral HPLC on ASH column (Hexane/^{i}PrOH = 98:2, 0.7 mL/min, UV detection at 214 nm); retention time = 7.14 min (major), retention time = 6.71 min (minor).

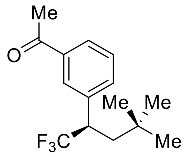

(*R*)-1-(3-(1,1,1-Trifluoro-4,4-dimethylpentan-2-yl)phenyl)ethan-1-one (4e). General Procedure A. The product (85.0 mg, 78% yield) was purified with silica gel chromatography (Petroleum ether/EtOAc = 20:1) as a colorless liquid. $[\alpha]_D^{20}$ = -23.82 (*c* = 0.34, $CHCl_3$) for a sample with 90% ee. **^{1}H NMR** (400 MHz, $CDCl_3$) δ 7.98 – 7.86 (m, 2 H), 7.56 (d, *J* = 7.7 Hz, 1 H), 7.47 (t, *J* = 7.7 Hz, 1 H), 3.41 (m, 1 H), 2.63 (s, 3 H), 1.95 (d, *J* = 6.0 Hz, 2 H), 0.80 (s, 9 H). **^{13}C NMR** (101 MHz, $CDCl_3$) δ 197.7, 137.6 (q, C-F, $^3J_{C-F}$ = 2.1 Hz), 137.4, 133.9, 129.1, 128.9, 128.2, 127.0 (q, C-F, $^1J_{C-F}$ = 279.8 Hz), 47.0 (q, C-F, $^2J_{C-F}$ = 26.3 Hz), 42.0 (q, C-F, $^3J_{C-F}$ = 1.8 Hz), 30.8, 29.7, 26.6. **^{19}F NMR** (376 MHz, $CDCl_3$) δ -70.31 (d, *J* = 9.9 Hz, 3 F). MS (EI): m/z (%) 257 (100), 272 $[M]^+$. HRMS (EI) m/z: $[M]^+$ Calcd. for $C_{15}H_{19}OF_3$: 272.1383; Found: 272.1389. IR (thin film) ν_{max} 2960, 2870, 1690, 1477, 1399, 1369, 1295, 1108, 701 cm^{-1}. Enantiomeric purity (90% ee) was measured by chiral HPLC on AY3 column (Hexane/^{i}PrOH = 98:2, 0.7 mL/min, UV detection at 214 nm); retention time = 4.72 min (major), retention time = 4.45 min (minor).

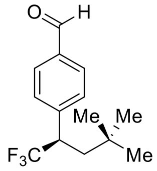

(*R*)-4-(1,1,1-Trifluoro-4,4-dimethylpentan-2-yl)benzaldehyde (4f). General Procedure A. The product (78.5 mg, 76% yield) was purified with silica gel chromatography (Petroleum ether/EtOAc = 50:1) as a white solid (m.p. 34.9-35.6 ^oC). $[\alpha]_D^{20}$ = -28.41 (*c* = 0.88, $CHCl_3$) for a sample with 90% ee. **^{1}H NMR** (400 MHz, $CDCl_3$) δ 10.02 (s, 1 H), 7.88 (d, *J* = 8.2 Hz, 2 H), 7.53 (d, *J* = 7.9 Hz, 2 H), 3.45 (qdd, *J* = 9.7, 6.9, 5.1 Hz, 1 H), 2.00 – 1.90 (m, 2 H), 0.80 (s, 9 H). **^{13}C NMR** (101 MHz, $CDCl_3$) δ 191.6, 143.6 (q, C-F, $^3J_{C-F}$ = 2.1 Hz), 136.1, 130.1, 129.8, 126.8 (q, C-F, $^1J_{C-F}$ = 279.7 Hz), 47.2 (q, C-F, $^2J_{C-F}$ = 26.4 Hz), 42.1 (q, C-F, $^3J_{C-F}$ = 1.9 Hz), 30.8, 29.6. **^{19}F NMR** (376 MHz, $CDCl_3$) δ -69.95 (d, *J* = 9.6 Hz, 3 F). MS (EI): m/z (%) 159, 202 (100), 215, 258 $[M]^+$. HRMS (EI) m/z: $[M]^+$ Calcd. for $C_{14}H_{17}OF_3$: 258.1226; Found: 258.1225. IR (thin film) ν_{max} 2960, 2869, 1698, 1610, 1477, 1370, 1296, 1259, 1153, 1107 cm^{-1}. Enantiomeric purity (90% ee) was measured by chiral HPLC on ASH column (Hexane/^{i}PrOH = 98:2, 0.7 mL/min, UV detection at 214 nm); retention time = 6.72 min (major), retention time = 6.35 min (minor).

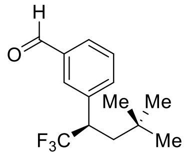

(*R*)-3-(1,1,1-Trifluoro-4,4-dimethylpentan-2-yl)benzaldehyde (4g). General Procedure A. The product (84.7 mg, 82% yield) was purified with silica gel

chromatography (Petroleum ether/EtOAc = 50:1) as a colorless liquid. $[\alpha]_D^{20}$ = -18.46 (c = 0.57, $CHCl_3$) for a sample with 89% ee. **^{1}H NMR** (400 MHz, $CDCl_3$) δ 10.04 (s, 1 H), 7.89 – 7.81 (m, 2 H), 7.62 (d, J = 7.6 Hz, 1 H), 7.54 (t, J = 7.6 Hz, 1 H), 3.44 (qdd, J = 9.8, 7.0, 4.9 Hz, 1 H), 2.06 – 1.88 (m, 2 H), 0.80 (s, 9 H). **^{13}C NMR** (101 MHz, $CDCl_3$) δ 191.9, 138.1 (q, C-F, $^3J_{C\text{-}F}$ = 2.1 Hz), 136.7, 135.4, 130.2, 129.7, 129.3, 126.9 (q, C-F, $^1J_{C\text{-}F}$ = 279.8 Hz), 46.8 (q, C-F, $^2J_{C\text{-}F}$ = 26.5 Hz), 42.0 (q, C-F, $^3J_{C\text{-}F}$ = 1.8 Hz), 30.8, 29.7. **^{19}F NMR** (376 MHz, $CDCl_3$) δ -70.32 (d, J = 9.8 Hz, 3 F). MS (EI): m/z (%) 202 (100), 258 $[M]^+$. HRMS (EI) m/z: $[M]^+$ Calcd. for $C_{14}H_{17}OF_3$: 258.1226; Found: 258.1224. IR (thin film) ν_{max} 2960, 2870, 2685, 2559, 1698, 1590, 1476, 1284, 1260, 1108, 712 cm^{-1}. The enantiomeric purity of **4g** (89% ee) was determined by **4g'**, which was derived from **4g** through reduction as showing below.

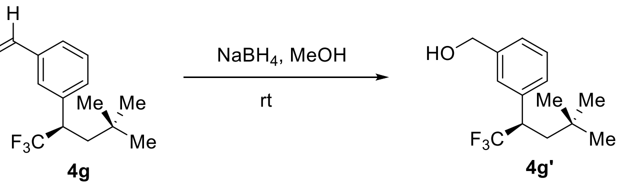

4g **4g'**

(*R*)-(3-(1,1,1-Trifluoro-4,4-dimethylpentan-2-yl)phenyl)methanol (4g'). To a solution of (*R*)-(4-(1,1,1-trifluoro-4,4-dimethylpentan-2-yl)phenyl)methanol **4g** (126.1 mg, 0.5 mmol) in MeOH (2 mL) was added $NaBH_4$ (22.7mg, 0.6 mmol). The mixture was stirred at rt for 2 h and then quenched with H_2O. The solvent was evaporated, the crude product was redissolved in EtOAc and washed with H_2O. The organic layer was dried (Na_2SO_4), filtered, and concentrated. The residue was purified with silica gel chromatography (Petroleum ether/EtOAc = 6:1) to give **4g′** as a colorless oil. (109.4 mg, 84%), $[\alpha]_D^{20}$ = -24.67 (c = 0.95, $CHCl_3$) for a sample with 89% ee. **^{1}H NMR** (400 MHz, $CDCl_3$) δ 7.37 – 7.15 (m, 4 H), 4.64 (s, 2 H), 3.32 (m, 1 H), 2.27 (br, 1 H), 2.01 – 1.83 (m, 2 H), 0.79 (s, 9 H). **^{13}C NMR** (101 MHz, $CDCl_3$) δ 141.2, 137.1 (q, C-F, $^3J_{C\text{-}F}$ = 1.8 Hz), 128.7, 128.6, 127.8, 127.2 (q, $^1J_{C\text{-}F}$ = 279.6 Hz),126.4, 64.9 (d, J = 4.5 Hz), 47.0 (q, $^2J_{C\text{-}F}$ = 26.1 Hz), 42.0 (q, C-F, $^3J_{C\text{-}F}$ = 2.0 Hz), 30.8, 29.6. **^{19}F NMR** (376 MHz, $CDCl_3$) δ -70.23 (d, J = 10.2 Hz, 3 F). MS (FT): m/z (%) 278 (100) $[M+NH_4]^+$. HRMS (DART) m/z: $[M+NH_4]^+$ Calcd. for $C_{14}H_{23}NOF_3$: 278.1726; Found: 278.1725. IR (thin film) ν_{max} 3320, 2959, 2869, 1476, 1370, 1261, 1177, 1107, 707 cm^{-1}. Enantiomeric purity (89% ee) was measured by chiral HPLC on OJH column (Hexane/^{i}PrOH = 98:2, 0.7 mL/min, UV detection at 214 nm); retention time = 13.83 min (major), retention time = 16.91 min (minor).

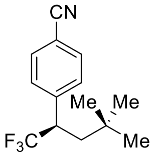

(*R*)-4-(1,1,1-Trifluoro-4,4-dimethylpentan-2-yl)benzonitrile (4h). General Procedure A. The product (74.5 mg, 73% yield) was purified with silica gel chromatography (Petroleum ether/EtOAc = 30:1) as a white solid (m.p. 40.4-40.8 °C). $[\alpha]_D^{20}$ = -31.48 (*c* = 0.50, $CHCl_3$) for a sample with 91% ee. **^{1}H NMR** (400 MHz, $CDCl_3$) δ 7.66 (d, *J* = 8.4 Hz, 2 H), 7.47 (d, *J* = 8.1 Hz, 2 H), 3.40 (pd, *J* = 9.5, 2.6 Hz, 1 H), 2.01 – 1.85 (m, 2 H), 0.79 (s, 9 H). **^{13}C NMR** (101 MHz, $CDCl_3$) δ 142.2 (q, C-F, $^3J_{C-F}$ = 1.9 Hz), 132.3, 130.2, 126.6 (q, C-F, $^1J_{C-F}$ = 279.9 Hz), 118.3, 112.1, 47.1 (q, C-F, $^2J_{C-F}$ = 26.7 Hz), 42.0 (q, C-F, $^3J_{C-F}$ = 1.8 Hz), 30.8, 29.6. **^{19}F NMR** (376 MHz, $CDCl_3$) δ -70.04 (d, *J* = 9.6 Hz, 3 F). MS (EI): m/z (%) 57 (100), 184, 240, 255 $[M]^+$. HRMS (EI) m/z: $[M]^+$ Calcd for $C_{14}H_{16}NF_3$: 255.1229; Found: 255.1229. IR (thin film) ν_{max} 2964, 2871, 2230, 1508, 1476, 1399, 1287, 1175, 1105, 831 cm^{-1}. The enantiomeric purity of **4h** (91% ee) was determined by **4h'**, which was derived from **4h** through oxidation as showing below.

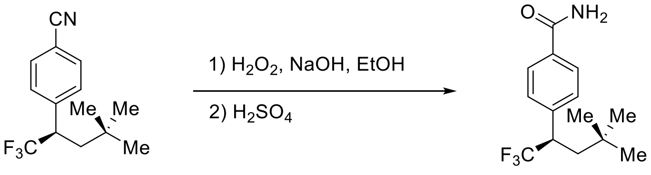

4h **4h'**

(*R*)-4-(1,1,1-Trifluoro-4,4-dimethylpentan-2-yl)benzamide (**4h'**). To a solution of (*R*)-4-(1,1,1-trifluoro-4,4-dimethylpentan-2-yl)benzonitrile **4h** (102.1 mg, 0.4 mmol) in a mixture of EtOH (0.3 mL) and aqueous NaOH (25 wt%, 0.05 mL) was added aqueous H_2O_2 (30 wt%, 0.2 mL). After stirring at rt for 6 h, the reaction was quenched with 2 drops of 50% H_2SO_4. EtOH was evaporated, and the resulting residue was redissolved in EtOAc and washed with H_2O. The combined organic layers were dried (Na_2SO_4), filtered, and concentrated to give a thick oil. The resulting thick white suspension was diluted with hexane (10 mL) and DCM (1 mL), and filtered. The solid was washed with hexane (5 mL) and dried in vacuo affording the product as a white solid (76.5 mg, 75%, m.p. 120.7-121.2 °C), $[\alpha]_D^{20}$ = -26.99 (*c* = 0.69, $CHCl_3$) for a sample with 91% ee. **^{1}H NMR** (400 MHz, $CDCl_3$) δ 7.81 (d, *J* = 8.0 Hz, 2 H), 7.41 (d, *J* = 7.8 Hz, 2 H), 6.39 (br, 2 H), 3.38 (qd, *J* = 10.1, 4.3 Hz, 1 H), 2.04 – 1.81 (m, 2 H), 0.79 (s, 9 H). **^{13}C NMR** (101 MHz, $CDCl_3$) δ 169.3, 140.9, 133.1, 129.7, 127.6, 126.9 (q, C-F, $^1J_{C-F}$ = 279.7 Hz), 46.9 (q, C-F, $^2J_{C-F}$ = 26.3 Hz), 42.0, 30.8, 29.6. **^{19}F NMR** (376 MHz, $CDCl_3$) δ -70.11 (d, *J* = 9.5 Hz, 3 F). MS (ESI): m/z (%) 274 (26) $[M]^+$. HRMS (ESI) m/z: $[M+H]^+$ Calcd. for $C_{14}H_{19}NOF_3$: 274.1413; Found: 274.1406. IR (thin film) ν_{max} 3382, 3199, 2961, 2870, 1656, 1615,

1570, 1262, 1175, 1107 cm^{-1}. Enantiomeric purity (91% ee) was measured by chiral HPLC on ASH column (Hexane/^{i}PrOH/DEA = 96:4:0.1, 0.7 mL/min, UV detection at 230 nm); retention time = 55.67 min (major), retention time = 64.53 min (minor).

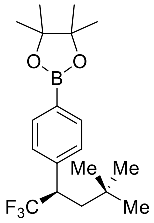

(*R*)-4,4,5,5-Tetramethyl-2-(4-(1,1,1-trifluoro-4,4-dimethylpentan-2-yl)phenyl)-1,3,2-dioxaborolane (4i). General Procedure A. The product (78.7 mg, 55% yield) was purified with silica gel chromatography (Petroleum ether/EtOAc = 50:1) as a white solid (m.p. 137.5-138.5 °C). $[\alpha]_D^{20}$ = -17.00 (*c* = 0.50, $CHCl_3$) for a sample with 89% ee. **^{1}H NMR** (400 MHz, $CDCl_3$) δ 7.79 (d, *J* = 7.3 Hz, 2 H), 7.34 (d, *J* = 7.7 Hz, 2 H), 3.33 (pd, *J* = 9.7, 3.0 Hz, 1 H), 2.01 – 1.84 (m, 2 H), 1.34 (s, 12 H), 0.78 (s, 9 H). **^{13}C NMR** (101 MHz, $CDCl_3$) δ 139.9 (q, C-F, $^3J_{C-F}$ = 2.2 Hz), 134.9, 128.8, 127.6 (q, C-F, $^1J_{C-F}$ = 279.9 Hz), 83.8, 47.2 (q, C-F, $^2J_{C-F}$ = 26.3 Hz), 42.0, 30.8, 29.7, 24.9, 24.9. **^{19}F NMR** (376 MHz, $CDCl_3$) δ -70.16 (d, *J* = 9.8 Hz, 3 F). MS (ESI): m/z (%) 219, 357 (100) $[M+H]^+$. HRMS (EI) m/z: $[M]^+$ Calcd. for $C_{19}H_{28}{}^{10}BO_2F_3$: 355.2165; Found: 355.2160. IR (thin film) ν_{max} 2961, 2869, 1614, 1400, 1362, 1258, 1145, 1093, 690 cm^{-1}. Enantiomeric purity (89% ee) was measured by chiral HPLC on IG column (Hexane/^{i}PrOH = 99:1, 0.7 mL/min, UV detection at 214 nm); retention time = 4.97 min (major), retention time = 5.36 min (minor).

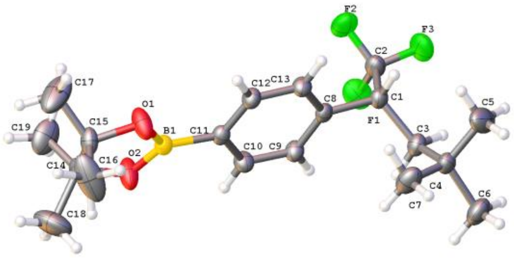

Supplementary Figure 1. X-ray crystal structure of compound **4i**

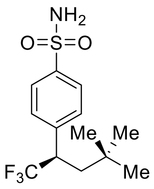

(*R*)-4-(1,1,1-Trifluoro-4,4-dimethylpentan-2-yl)benzenesulfonamide (4j). General Procedure A. The product (85.9 mg, 70% yield) was purified with silica gel chromatography (Petroleum ether/EtOAc = 2:1) as a white solid (m.p. 142.1-143.2 °C). $[\alpha]_D^{20}$ = -19.36 (*c* = 0.56, $CHCl_3$) for a sample with 91% ee. **^{1}H NMR** (400 MHz, $CDCl_3$) δ 7.92 (d, *J* = 8.0 Hz, 2 H), 7.50 (d, *J* = 8.0 Hz, 2 H), 5.14 (s, 2 H), 3.41 (pd, *J* = 9.5, 2.7 Hz, 1H), 2.03 – 1.85 (m, 2 H), 0.80 (s, 9 H). **^{13}C NMR** (101 MHz, $CDCl_3$) δ 142.1 (q, C-F, $^3J_{C-F}$ = 1.9 Hz), 141.6, 130.2, 126.7, 126.7 (q, C-F, $^1J_{C-F}$ = 280.1 Hz), 46.9 (q, C-F, $^2J_{C-F}$ = 26.6 Hz), 42.1 (q, C-F, $^3J_{C-F}$ = 1.8

Hz), 30.8, 29.7. **^{19}F NMR** (376 MHz, $CDCl_3$) δ -70.02 (d, J = 9.6 Hz, 3 F). MS (ESI): m/z (%) 332 (100) $[M+Na]^+$. HRMS (ESI) m/z: $[M+Na]^+$ Calcd. for $C_{13}H_{18}NO_2F_3NaS$: 332.0903; Found: 332.0901. IR (thin film) ν_{max} 3375, 3275, 2963, 2870, 1550, 1478, 1400, 1331, 1163, 672 cm^{-1}. Enantiomeric purity (91% ee) was measured by chiral HPLC on ADH column (Hexane/^{i}PrOH = 9:1, 0.7 mL/min, UV detection at 214 nm); retention time = 11.88 min (major), retention time = 10.76 min (minor).

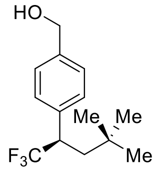

(*R*)-(4-(1,1,1-Trifluoro-4,4-dimethylpentan-2-yl)phenyl)methanol (4k). General Procedure A. 0.5 equiv of NaI was used. The product (64.4 mg, 62% yield) was purified with silica gel chromatography (Petroleum ether/EtOAc = 6:1) as a colorless liquid. $[\alpha]_D^{20}$ = -18.48 (c = 0.67, $CHCl_3$) for a sample with 93% ee. **^{1}H NMR** (400 MHz, $CDCl_3$) δ 7.33 (m, 4 H), 4.68 (s, 2 H), 3.33 (dtt, J = 14.0, 5.8, 2.9 Hz, 1 H), 2.00 – 1.86 (m, 2 H), 1.79 (br, 1 H), 0.79 (s, 9 H). **^{13}C NMR** (101 MHz, $CDCl_3$) δ 140.6, 136.2 (q, C-F, $^3J_{C\text{-}F}$ = 2.3 Hz), 129.6, 127.2 (q, C-F, $^1J_{C\text{-}F}$ = 279.6 Hz), 127.1, 64.9, 46.8 (q, C-F, $^2J_{C\text{-}F}$ = 26.3 Hz), 42.0 (q, C-F, $^3J_{C\text{-}F}$ = 2.3 Hz), 30.8, 29.7. **^{19}F NMR** (376 MHz, $CDCl_3$) δ -70.37 (d, J = 10.2 Hz). MS (ESI): m/z (%) 243 (100) $[M\text{-}OH]^+$. HRMS (EI) m/z: $[M]^+$ Calcd. for $C_{14}H_{19}OF_3$: 260.1383; Found: 260.1381. IR (thin film) ν_{max} 3346, 2959, 2869, 1476, 1370, 1246, 1174, 1105 cm^{-1}. Enantiomeric purity (93% ee) was measured by chiral HPLC on PC4 column (Hexane/^{i}PrOH = 9:1, 0.7 mL/min, UV detection at 224 nm); retention time = 22.70 min (major), retention time = 26.64 min (minor).

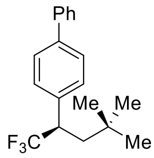

(*R*)-4-(1,1,1-Trifluoro-4,4-dimethylpentan-2-yl)-1,1'-biphenyl (4l). General Procedure A. The product (97.5 mg, 79% yield) was purified with silica gel chromatography (Petroleum ether) as a white solid (m.p. 101.2-101.9 °C). $[\alpha]_D^{20}$ = -21.64 (c = 0.55, $CHCl_3$) for a sample with 88% ee. **^{1}H NMR** (400 MHz, $CDCl_3$) δ 7.65 – 7.51 (m, 4 H), 7.49 – 7.29 (m, 5 H), 3.36 (dtd, J = 17.6, 10.0, 4.2 Hz, 1 H), 2.03 – 1.86 (m, 2 H) , 0.82 (s, 9 H). **^{13}C NMR** (101 MHz, $CDCl_3$) δ 140.8, 140.5, 135.8 (q, C-F, $^3J_{C\text{-}F}$ = 2.1 Hz), 129.8, 128.8, 127.4, 127.2, 127.0, 127.3 (q, C-F, $^1J_{C\text{-}F}$ = 279.6 Hz), 46.7 (q, C-F, $^2J_{C\text{-}F}$ = 26.2 Hz), 42.1 (q, C-F, $^3J_{C\text{-}F}$ = 1.9 Hz), 30.8, 29.7. **^{19}F NMR** (376 MHz, $CDCl_3$) δ -70.01 (d, J = 9.6 Hz, 3 F). MS (EI): m/z (%) 306 (100) $[M]^+$. HRMS (EI) m/z: $[M]^+$ Calcd. for $C_{19}H_{21}F_3$: 306.1590; Found: 306.1595. IR (thin film) ν_{max} 2962, 2870, 1489, 1452, 1370, 1208, 1100, 763 cm^{-1}. Enantiomeric purity (88% ee) was measured by chiral HPLC

on PC-3 column ($MeCN/H_2O$ = 70:30, 0.7 mL/min, UV detection at 214 nm); retention time = 12.75 min (major), retention time = 11.07 min (minor).

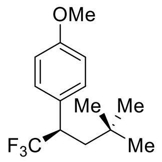

(*R*)-1-Methoxy-4-(1,1,1-trifluoro-4,4-dimethylpentan-2-yl)benzene (4m). General Procedure A. 10 mol% DMAP, **2a** (0.4 mmol, 1.0 equiv), **3m** (0.6 mmol, 1.5 equiv), and 0.5 equiv of NaI were used. The product (40.4 mg, 40% yield) was purified with silica gel chromatography (Petroleum ether/DCM = 50:1) as a colorless oil. $[\alpha]_D^{20}$ = -12.37 (*c* = 1.18, $CHCl_3$) for a sample with 88% ee. **^{1}H NMR** (400 MHz, $CDCl_3$) δ 7.23 (d, *J* = 8.5 Hz, 2 H), 6.86 (d, *J* = 8.6 Hz, 2 H), 3.79 (s, 3 H), 3.26 (tq, *J* = 9.8, 5.4, 4.8 Hz, 1 H), 1.94 – 1.81 (m, 2 H), 0.79 (s, 9 H). **^{13}C NMR** (101 MHz, $CDCl_3$) δ 159.2, 130.4, 128.7 (q, C-F, $^3J_{C-F}$ = 2.0 Hz), 127.4 (q, C-F, $^1J_{C-F}$ = 279.7 Hz), 113.9, 55.1, 46.2 (q, C-F, $^2J_{C-F}$ = 26.1 Hz), 42.0 (q, C-F, $^3J_{C-F}$ = 2.0 Hz), 30.7, 29.7. **^{19}F NMR** (376 MHz, $CDCl_3$) δ -70.70 (d, *J* = 9.5 Hz). MS (FI): m/z (%) 260 (100) $[M]^+$. HRMS (FI) m/z: $[M]^+$ Calcd. for $C_{14}H_{19}OF_3$: 260.1388; Found: 260.1386. IR (thin film) ν_{max} 2958, 1615, 1518, 1467, 1248, 1104, 825 cm^{-1}. Enantiomeric purity (88% ee) was measured by chiral HPLC on OJH column (Hexane/^{i}PrOH = 6:4, 0.7 mL/min, UV detection at 214 nm); retention time = 5.63 min (major), retention time = 6.14 min (minor).

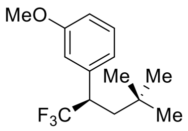

(*R*)-1-Methoxy-3-(1,1,1-trifluoro-4,4-dimethylpentan-2-yl)benzene (4n). General Procedure A. The product (59.3 mg, 57% yield) was purified with silica gel chromatography (Petroleum ether/EtOAc = 50:1) as a colorless liquid. $[\alpha]_D^{20}$ = -19.53 (*c* = 0.38, $CHCl_3$) for a sample with 89% ee. **^{1}H NMR** (400 MHz, $CDCl_3$) δ 7.32 – 7.16 (m, 1 H), 6.95 – 6.79 (m, 3 H), 3.81 (s, 3 H), 3.29 (dtd, *J* = 19.7, 9.9, 4.0 Hz, 1 H), 1.98 – 1.82 (m, 2 H), 0.81 (s, 9 H). **^{13}C NMR** (101 MHz, $CDCl_3$) δ 159.6, 138.3 (q, C-F, $^3J_{C-F}$ = 2.3 Hz), 129.4, 127.2 (q, C-F, $^1J_{C-F}$ = 279.7 Hz), 121.9, 115.5, 112.9, 55.2, 47.0 (q, C-F, $^2J_{C-F}$ = 26.0 Hz, 3 F), 42.1 (q, C-F, $^3J_{C-F}$ = 1.9 Hz), 30.8, 29.7. **^{19}F NMR** (376 MHz, $CDCl_3$) δ -70.26 (d, *J* = 10.0 Hz). MS (EI): m/z (%) 122 (100), 260 $[M]^+$. HRMS (EI) m/z: $[M]^+$ Calcd. for $C_{14}H_{19}OF_3$: 260.1383; Found: 260.1385. IR (thin film) ν_{max} 2958, 2869, 1604, 1588, 1495, 1457, 1370, 1260, 1108, 707 cm^{-1}. Enantiomeric purity (89% ee) was measured by chiral HPLC on OJH column (Hexane/^{i}PrOH = 98:2, 0.7 mL/min, UV detection at 214 nm); retention time = 5.18 min (major), retention time = 5.46 min (minor).

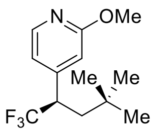

(*R*)-2-Methoxy-4-(1,1,1-trifluoro-4,4-dimethylpentan-2-yl)pyridine (4o). General Procedure A. The product (55.1 mg, 53% yield) was purified with silica gel chromatography (Petroleum ether/EtOAc = 50:1) as a colorless liquid. $[\alpha]_D^{20}$ = -9.23 (*c* = 0.39, $CHCl_3$) for a sample with 91% ee. **^{1}H NMR** (400 MHz, $CDCl_3$) δ 8.14 (d, *J* = 5.3 Hz, 1 H), 6.86 (d, *J* = 5.3 Hz, 1 H), 6.73 (s, 1 H), 3.95 (s, 3 H), 3.33 – 3.19 (m, 1 H), 1.97 – 1.77 (m, 2 H), 0.82 (s, 9 H).**^{13}C NMR** (101 MHz, $CDCl_3$) δ 164.5, 148.6 (q, C-F, $^3J_{C\text{-}F}$ = 2.1 Hz), 147.1, 126.6 (q, C-F, $^1J_{C\text{-}F}$ = 279.9 Hz), 117.6, 111.9, 53.5, 46.5 (q, C-F, $^2J_{C\text{-}F}$ = 26.6 Hz), 41.7 (q, C-F, $^3J_{C\text{-}F}$ = 1.8 Hz), 30.8, 29.6. **^{19}F NMR** (376 MHz, $CDCl_3$) δ -70.01 (d, *J* = 9.6 Hz, 3 F). MS (ESI): m/z (%) 262 (100) $[M+H]^+$. HRMS (ESI) m/z: $[M+H]^+$ Calcd. for $C_{13}H_{19}NOF_3$: 262.1413; Found: 262.1411. Enantiomeric purity (91% ee) was measured by chiral HPLC on ASH column (Hexane/^{i}PrOH = 98:2, 0.7 mL/min, UV detection at 214 nm); retention time = 4.86 min (major), retention time = 5.24 min (minor). ν_{max} 2958, 2870, 1614, 1562, 1484, 1403, 1316, 1261, 1109, 708 cm^{-1}.

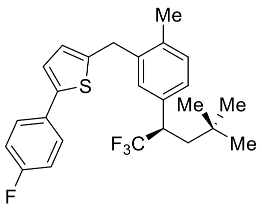

(*R*)-2-(4-Fluorophenyl)-5-(2-methyl-5-(1,1,1-trifluoro-4,4-dimethylpentan-2-yl)benzyl)thiophene (4p). General Procedure A. 0.5 equiv of NaI was used. The product (142.9 mg, 82% yield) was purified with silica gel chromatography (Petroleum ether/EtOAc = 100:1) as a colorless oil. $[\alpha]_D^{20}$ = -7.44 (*c* = 0.78, $CHCl_3$) for a sample with 88% ee. **^{1}H NMR** (400 MHz, $CDCl_3$) δ 7.53 – 7.39 (m, 2 H), 7.18 (s, 1 H), 7.15 (s, 2 H), 7.09 – 6.92 (m, 3 H), 6.60 (m, 1 H), 4.12 (s, 2 H), 3.38 – 3.20 (m, 1 H), 2.28 (s, 3 H), 1.97 – 1.83 (m, 2 H), 0.81 (s, 9 H). **^{13}C NMR** (101 MHz, $CDCl_3$) δ 162.1 (d, C-F, $^1J_{C\text{-}F}$ = 246.8 Hz), 143.4, 141.5, 138.2, 136.2, 134.6 (q, C-F, $^3J_{C\text{-}F}$ = 1.8 Hz), 130.9 (d, C-F, $^4J_{C\text{-}F}$ = 3.3 Hz), 130.8, 130.7, 127.9, 127.3 (q, C-F, $^1J_{C\text{-}F}$ = 275.5 Hz), 127.1 (d, C-F, $^3J_{C\text{-}F}$ = 8.1 Hz), 125.8, 122.6, 115.7 (d, C-F, $^2J_{C\text{-}F}$ = 21.8 Hz), 46.7 (q, C-F, $^2J_{C\text{-}F}$ = 26.0 Hz), 42.0, 34.2, 30.8, 29.8, 19.1. **^{19}F NMR** (376 MHz, $CDCl_3$) δ -70.35 (d, *J* = 10.1 Hz, 3 F), -115.20 (m, 1 F). MS (ESI): m/z (%) 435 (100) $[M]^+$, 452 $[M+NH_4]^+$. HRMS (DART) m/z: $[M+H]^+$ Calcd. for $C_{25}H_{27}F_4S$: 435.1764; Found: 435.1762. IR (thin film) ν_{max} 2957, 2867, 1509, 1469, 1398, 1234, 1174, 1106, 833 cm^{-1}. Enantiomeric purity (88% ee) was measured by chiral HPLC on OJH column (Hexane/^{i}PrOH = 85:15, 0.7 mL/min, UV detection at 214 nm); retention time = 7.76 min (major), retention time = 6.97 min (minor).

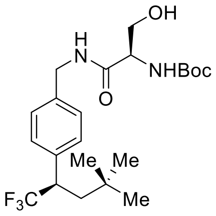

***tert*-Butyl ((*R*)-3-hydroxy-1-oxo-1-((4-((*R*)-1,1,1-trifluoro-4,4-dimethylpentan-2-yl)benzyl)amino)propan-2-yl)carbamate (4q)**. General Procedure A. 0.5 equiv of NaI, 13 mol% $NiBr_2$·DME and 13 mol% **L6** were used. The product (40.9 mg, 46% yield) was purified with silica gel chromatography (Petroleum ether/EtOAc = 2:1) as a colorless liquid. $[\alpha]_D^{20}$ = -39.26 (c = 1.03, $CHCl_3$) for a sample with 88% ee. **^{1}H NMR** (400 MHz, $CDCl_3$) δ 7.27 (d, J = 8.0 Hz, 2 H), 7.22 (d, J = 8.0 Hz, 2 H), 7.18 (br, 1 H), 5.69 (d, J = 7.1 Hz, 1 H), 4.56 – 4.33 (m, 2 H), 4.21 (s, 1 H), 4.15 – 4.02 (m, 1 H), 3.69 (s, 1 H), 3.47 (s, 1 H), 3.36 – 3.22 (m, 1 H), 1.93 – 1.85 (m, 2 H), 1.40 (s, 9 H), 0.79 (s, 9 H). **^{13}C NMR** (101 MHz, $CDCl_3$) δ 171.4, 156.3, 137.5, 136.1, 129.7, 127.6, 127.1 (q, C-F, $^1J_{C\text{-}F}$ = 279.7 Hz), 80.6, 62.7, 55.0, 46.6 (q, C-F, $^2J_{C\text{-}F}$ = 26.3 Hz), 42.9, 42.0, 30.8, 29.7, 28.2. **^{19}F NMR** (376 MHz, $CDCl_3$) δ -70.36 (d, J = 9.5 Hz, 3 F). MS (ESI): m/z (%) 469 (100) $[M+Na]^+$, 447 (57) $[M+H]^+$. HRMS (FT) m/z: $[M+H]^+$ Calcd. for $C_{22}H_{34}N_2O_4F_3$: 447.2465; Found: 447.2473. IR (thin film) ν_{max} 3301, 2961, 2870, 1701, 1648, 1541, 1475, 1368, 1261, 1105 cm^{-1}. The enantiomeric purity of **4q** (88% ee) was determined by **4q'**, which was derived from **4q** as showing below.

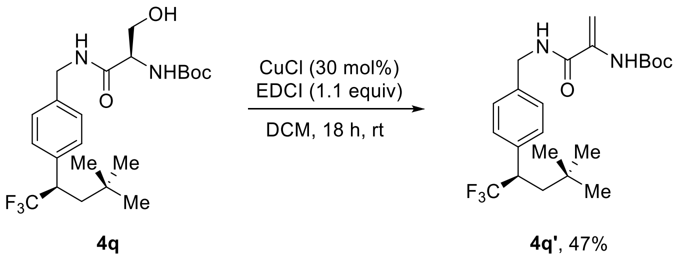

***tert*-Butyl (*R*)-(3-oxo-3-((4-(1,1,1-trifluoro-4,4-dimethylpentan-2-yl)benzyl)amino)prop-1-en-2-yl)carbamate (4q').** To a 25 mL Schlenk tube was added *tert*-butyl ((*R*)-3-hydroxy-1-oxo-1-((4-((*R*)-1,1,1-trifluoro-4,4-dimethylpentan-2-yl)benzyl)amino)propan-2-yl)carbamate (**4q**) (178.6 mg, 0.4 mmol), EDCI·HCl (84.3 mg, 0.44 mmol) and CuCl (11.9 mg, 0.12 mmol). The tube was then purged with Ar for three times. Anhydrous DCM (4 mL) were added under Ar. The tube was sealed with Teflon cap. After stirring at 800 rpm for 18 h at room temperature, the reaction mixture was diluted with DCM, washed with water and brine. The organic layer was dried over Na_2SO_4, filtered and concentrated. The resulting mixture was purified with silica gel chromatography (Petroleum ether/EtOAc = 8:1) to give product **4q'** as a colorless oil (80.7 mg, 47%) with 88% ee. $[\alpha]_D^{20}$ = -7.71 (c = 1.11, $CHCl_3$) for a sample with 88% ee. **^{1}H NMR** (400 MHz, $CDCl_3$) δ 7.35 (br, 1 H), 7.30 (d, J

= 8.2 Hz, 2 H), 7.24 (d, J = 8.1 Hz, 2 H), 6.57 (t, J = 6.0 Hz, 1 H), 6.04 – 5.96 (m, 1 H), 5.07 (t, J = 1.8 Hz, 1 H), 4.50 (d, J = 5.9 Hz, 2 H), 3.39 – 3.23 (m, 1 H), 1.94 – 1.85 (m, 2 H), 1.47 (s, 9 H), 0.79 (s, 9 H). **^{13}C NMR** (126 MHz, $CDCl_3$) δ 164.0, 152.7, 137.3, 136.3 (q, C-F, $^3J_{C\text{-}F}$ = 1.3 Hz), 134.8, 129.8, 127.8, 127.1 (q, C-F, $^1J_{C\text{-}F}$ = 279.6 Hz), 97.6, 80.6, 46.6 (q, C-F, $^2J_{C\text{-}F}$ = 26.1 Hz), 43.5, 42.0, 30.8, 29.6, 28.2. **^{19}F NMR** (376 MHz, $CDCl_3$) δ -70.32 (d, J = 9.5 Hz). MS (ESI): m/z (%) 373 (100) $[M\text{-}C_4H_7]^+$, 429 (5) $[M+H]^+$. Enantiomeric purity (88% ee) was measured by chiral HPLC on ODH column (Hexane/^{i}PrOH = 95:5, 0.7 mL/min, UV detection at 214 nm); retention time = 20.00 min (major), retention time = 15.68 min (minor).

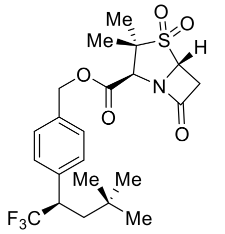

4-((*R*)-1,1,1-Trifluoro-4,4-dimethylpentan-2-yl)benzyl (2*S*,5*R*)-3,3-dimethyl-7-oxo-4-thia-1-azabicyclo[3.2.0]heptane-2-carboxylate 4,4-dioxide (4r). General Procedure A. The product (122.9 mg, 65% yield) was purified with silica gel chromatography (Petroleum ether/EtOAc = 6:1) as a white solid (m.p. 49.0-50.9 ^{o}C). $[\alpha]_D^{20}$ = 126.85 (c = 0.82, $CHCl_3$) for a sample with 90% de. **^{1}H NMR** (400 MHz, $CDCl_3$) δ 7.36 (m, 4 H), 5.30 (d, J = 12.1 Hz, 1 H), 5.15 (d, J = 12.0 Hz, 1 H), 4.64 – 4.55 (m, 1 H), 4.41 (s, 1 H), 3.55 – 3.25 (m, 3 H), 2.00 – 1.84 (m, 2 H), 1.53 (s, 3 H), 1.19 (s, 3 H), 0.78 (s, 9 H). **^{13}C NMR** (101 MHz, $CDCl_3$) δ 170.7, 166.7, 137.7 (q, C-F, $^3J_{C\text{-}F}$ = 2.1 Hz), 134.1, 129.8, 129.0, 127.0 (q, C-F, $^1J_{C\text{-}F}$ = 279.8 Hz), 67.5, 63.0, 62.6, 61.0, 46.7 (q, C-F, $^2J_{C\text{-}F}$ = 26.3 Hz), 41.9, 38.2, 30.7, 29.6, 19.8, 18.4. **^{19}F NMR** (376 MHz, $CDCl_3$) δ -70.26 (d, J = 9.5 Hz). MS (ESI): m/z (%) 498 (100) $[M+Na]^+$. HRMS (ESI) m/z: $[M+Na]^+$ Calcd. for $C_{22}H_{28}NO_5F_3NaS$: 498.1533; Found: 498.1530. IR (thin film) ν_{max} 2960, 2870, 1800, 1757, 1467, 1399, 1262, 1186, 1105 cm^{-1}. Enantiomeric purity (90% de) was measured by chiral HPLC on ADH column (Hexane/^{i}PrOH = 9:1, 0.7 mL/min, UV detection at 214 nm); retention time = 19.59 min (major), retention time = 18.64 min (minor).

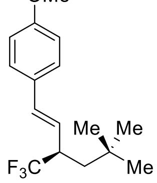

(*R*,*E*)-1-(5,5-Dimethyl-3-(trifluoromethyl)hex-1-en-1-yl)-4-methoxybenzene (4s). General Procedure A. The product (45.5 mg, 40% yield) was purified with silica gel chromatography (Petroleum ether/DCM = 10:1) as a colorless oil. $[\alpha]_D^{20}$ = -69.14 (c = 0.88, $CHCl_3$) for a sample with 73% ee. **^{1}H NMR** (400 MHz, $CDCl_3$) δ 7.31 (d, J = 8.7 Hz, 2 H), 6.86 (d, J = 8.7 Hz, 2 H), 6.51 (d, J = 15.8 Hz, 1 H), 5.82 (dd, J = 15.9, 9.2 Hz, 1 H), 3.81

(s, 3 H), 2.92 (qd, J = 9.5, 2.2 Hz, 1 H), 1.72 (dd, J = 14.1, 2.2 Hz, 1 H), 1.52 (dd, J = 14.1, 9.4 Hz, 1 H), 0.94 (s, 9 H). **^{13}C NMR** (101 MHz, $CDCl_3$) δ 159.5, 134.5, 129.3, 127.6, 127.2 (q, C-F, $^1J_{C-F}$ = 279.7 Hz), 123.2 (q, C-F, $^3J_{C-F}$ = 2.3 Hz), 114.0, 55.3, 44.8 (q, C-F, $^2J_{C-F}$ = 26.1 Hz), 41.3, 30.7, 29.8. **^{19}F NMR** (376 MHz, $CDCl_3$) δ -71.27 (d, J = 8.9 Hz). MS (FI): m/z (%) 286 (100) $[M]^+$. HRMS (FI) m/z: $[M]^+$ Calcd. for $C_{16}H_{21}OF_3$: 286.1543; Found: 286.1539. IR (thin film) ν_{max} 2957, 2867, 1608, 1513, 1466, 1254, 1173, 1107 cm^{-1}. Enantiomeric purity (73% ee) was measured by chiral HPLC on PC-3 column (MeCN/H_2O = 55:45, 0.7 mL/min, UV detection at 214 nm); retention time = 21.39 min (major), retention time = 19.80 min (minor).

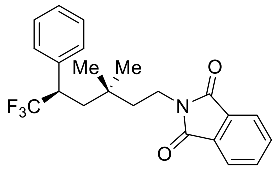

(*R*)-2-(6,6,6-Trifluoro-3,3-dimethyl-5-phenylhexyl)isoindoline-1,3-dione (5a). General Procedure B. $FeBr_2$ (0.25 equiv) instead of $FeCl_3$ (0.25 equiv) was used. The product (50.0 mg, 64% yield) was purified with silica gel chromatography (Petroleum ether/EtOAc = 15:1) as a colorless oil. $[\alpha]_D^{20}$ = -35.50 (c = 1.11, $CHCl_3$) for a sample with 86% ee. **^{1}H NMR** (400 MHz, $CDCl_3$) δ 7.88 – 7.80 (m, 2 H), 7.75 – 7.66 (m, 2 H), 7.39 – 7.27 (m, 5 H), 3.69 – 3.59 (m, 2 H), 3.46 (qdd, J = 9.9, 7.1, 4.8 Hz, 1 H), 2.08 – 1.96 (m, 2 H), 1.65 – 1.56 (m, 1 H), 1.55 – 1.45 (m, 1 H), 0.85 (s, 3 H), 0.82 (s, 3 H). **^{13}C NMR** (101 MHz, $CDCl_3$) δ 168.2, 136.4 (q, C-F, $^3J_{C-F}$ = 1.9 Hz), 133.9, 132.2, 129.4, 128.6, 128.0, 127.1 (q, C-F, $^1J_{C-F}$ = 279.9 Hz), 123.1, 46.4 (q, C-F, $^2J_{C-F}$ = 26.3 Hz), 40.0 (q, C-F, $^3J_{C-F}$ = 1.8 Hz), 33.9, 32.8, 27.3, 27.0. **^{19}F NMR** (376 MHz, $CDCl_3$) δ -70.26 (d, J = 10.2 Hz, 3 F). MS (ESI): m/z (%) 390 (100) $[M+H]^+$. HRMS (DART) m/z: $[M+H]^+$ Calcd. for $C_{22}H_{23}NO_2F_3$: 390.1675; Found: 390.1675. IR (thin film) ν_{max} 2960, 2873, 1712, 1468, 1401, 1256, 1161, 1106, 720 cm^{-1}. Enantiomeric purity (86% ee) was measured by chiral HPLC on IG column (Hexane/^{i}PrOH = 96:4, 0.7 mL/min, UV detection at 214 nm); retention time = 13.63 min (major), retention time = 14.96 min (minor).

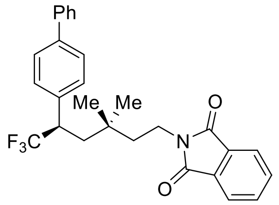

(*R*)-2-(5-([1,1'-Biphenyl]-4-yl)-6,6,6-trifluoro-3,3-dimethylhexyl)isoindoline-1,3-dione (5b). General Procedure B. The product (66.1 mg, 71% yield) was purified with silica gel chromatography (Petroleum ether/EtOAc = 15:1) as a white solid (m.p. 120.0-121.0 °C). $[\alpha]_D^{20}$ = -22.93 (c = 0.41, $CHCl_3$) for a sample with 87% ee. **^{1}H NMR** (400 MHz, $CDCl_3$) δ 7.82 (dt, J = 7.8, 3.7 Hz, 2

H), 7.68 (dd, J = 5.5, 3.0 Hz, 2 H), 7.62 – 7.48 (m, 4 H), 7.47 – 7.36 (m, 4 H), 7.33 (t, J = 7.4 Hz, 1 H), 3.73 – 3.58 (m, 2 H), 3.51 (tq, J = 15.6, 9.9, 7.8 Hz, 1 H), 2.12 – 1.98 (m, 2 H), 1.72 – 1.56 (m, 1 H), 1.55 – 1.45 (m, 1 H), 0.89 (s, 3 H), 0.85 (s, 3 H). **^{13}C NMR** (101 MHz, $CDCl_3$) δ 168.2, 140.9, 140.4, 135.4 (q, C-F, $^3J_{C\text{-}F}$ = 1.9 Hz), 133.8, 132.1, 129.7, 128.7, 127.4, 127.3, 127.2 (q, C-F, $^1J_{C\text{-}F}$ = 275.4 Hz), 127.0, 125.8, 123.1, 46.1 (q, C-F, $^2J_{C\text{-}F}$ = 26.3 Hz), 40.1, 39.9, 33.9, 32.9, 27.3, 27.1. **^{19}F NMR** (376 MHz, $CDCl_3$) δ -70.13 (d, J = 9.6 Hz, 3 F). MS (ESI): m/z (%) 483 (100) $[M+NH_4]^+$. HRMS (DART) m/z: $[M+H]^+$ Calcd. for $C_{28}H_{27}NO_2F_3$: 466.1988; Found: 466.1985. IR (thin film) ν_{max} 2953, 2924, 2871, 1717, 1400, 1374, 1245, 1100, 736 cm^{-1}. Enantiomeric purity (87% ee) was measured by chiral HPLC on IG column (Hexane/^{i}PrOH = 95:5, 0.7 mL/min, UV detection at 214 nm); retention time = 22.31 min (major), retention time = 21.14 min (minor).

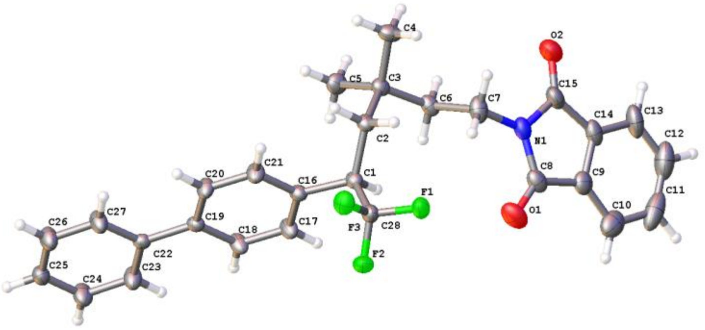

Supplementary Figure 2. X-ray crystal structure of compound **5b**

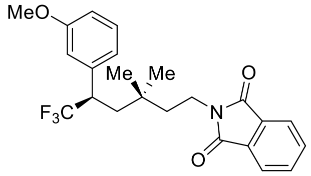

(*R*)-2-(6,6,6-Trifluoro-5-(3-methoxyphenyl)-3,3-dimethylhexyl)isoindoline-1,3-dione (5c). General Procedure B. The product (52.1 mg, 62% yield) was purified with silica gel chromatography (Petroleum ether/EtOAc = 10:1) as a colorless oil. $[\alpha]_D^{20}$ = -37.21 (c = 1.27, $CHCl_3$) for a sample with 88% ee. **^{1}H NMR** (400 MHz, $CDCl_3$) δ 7.88 – 7.80 (m, 2 H), 7.75 – 7.66 (m, 2 H), 7.30 – 7.21 (m, 1 H), 6.94 (d, J = 7.4 Hz, 1 H), 6.89 (s, 1 H), 6.84 (d, J = 8.2 Hz, 1 H), 3.81 (s, 3 H), 3.72 – 3.57 (m, 2 H), 3.50 – 3.35 (m, 1 H), 2.06 – 1.92 (m, 2 H), 1.66 – 1.54 (m, 2 H), 0.87 (s, 3 H), 0.83 (s, 3 H). **^{13}C NMR** (101 MHz, $CDCl_3$) δ 168.2, 159.7, 137.9 (q, C-F, $^3J_{C\text{-}F}$ = 2.3 Hz), 133.9, 132.2, 129.6, 127.1 (q, C-F, $^1J_{C\text{-}F}$ = 279.7 Hz), 123.1, 121.8, 115.4, 113.2, 55.2, 46.5 (q, C-F, $^2J_{C\text{-}F}$ = 26.1 Hz), 40.1, 40.0, 34.0, 32.8, 27.3, 27.0. **^{19}F NMR** (376 MHz, $CDCl_3$) δ -70.17 (d, J = 9.5 Hz, 3 F). MS (ESI): m/z (%) 442 (100) $[M+Na]^+$. HRMS (ESI) m/z: $[M+Na]^+$ Calcd. for $C_{23}H_{24}NO_3F_3Na$: 442.1601; Found:

442.1600. IR (thin film) ν_{max} 2960, 2872, 1713, 1603, 1457, 1401, 1259, 1158, 1106, 720 cm^{-1}. Enantiomeric purity (88% ee) was measured by chiral HPLC on IC column (Hexane/^{i}PrOH = 98:2, 0.7 mL/min, UV detection at 214 nm); retention time = 15.66 min (major), retention time = 14.89 min (minor).

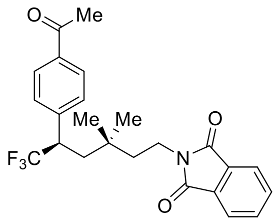

(*R*)-2-(5-(4-Acetylphenyl)-6,6,6-trifluoro-3,3-dimethylhexyl)isoindoline-1,3-dione (5d). General Procedure B. $FeBr_2$ (0.25 equiv) instead of $FeCl_3$ (0.25 equiv) was used. The product (53.4 mg, 62% yield) was purified with silica gel chromatography (Petroleum ether/EtOAc = 8:1) as a colorless oil. $[\alpha]_D^{20}$ = -53.56 (c = 0.37, $CHCl_3$) for a sample with 88% ee. **^{1}H NMR** (400 MHz, $CDCl_3$) δ 7.94 (d, J = 8.0 Hz, 2 H), 7.88 – 7.78 (m, 2 H), 7.76 – 7.66 (m, 2 H), 7.48 (d, J = 8.0 Hz, 2 H), 3.72 – 3.61 (m, 2 H), 3.61 – 3.48 (m, 1 H), 2.60 (s, 3 H), 2.11 – 1.97 (m, 2 H), 1.61 – 1.54 (m, 1 H), 1.54 – 1.45 (m, 1 H), 0.86 (s, 3 H), 0.81 (s, 3 H). **^{13}C NMR** (101 MHz, $CDCl_3$) δ 197.6, 168.2, 141.7, 136.9, 133.9, 132.1, 129.7, 128.7, 126.8 (q, C-F, $^1J_{C-F}$ = 280.1 Hz), 123.2, 46.5 (q, C-F, $^2J_{C-F}$ = 26.4 Hz), 40.0, 39.8, 33.9, 32.9, 27.4 27.2, 26.6. **^{19}F NMR** (376 MHz, $CDCl_3$) δ -69.94 (d, J = 9.7 Hz, 3 F). MS (ESI): m/z (%) 454 (100) $[M+Na]^+$. HRMS (ESI) m/z: $[M+Na]^+$ Calcd. for $C_{24}H_{24}NO_3F_3Na$: 454.1601; Found: 454.1592. IR (thin film) ν_{max} 2960, 2873, 1714, 1686, 1402, 1373, 1256, 1161, 1107, 720 cm^{-1}. Enantiomeric purity (88% ee) was measured by chiral HPLC on OJH column (Hexane/^{i}PrOH = 95:5, 0.7 mL/min, UV detection at 214 nm); retention time = 41.13 min (major), retention time = 46.65 min (minor).

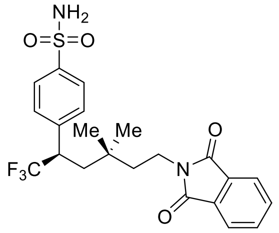

(*R*)-4-(6-(1,3-Dioxoisoindolin-2-yl)-1,1,1-trifluoro-4,4-dimethylhexan-2-yl)benzenesulfonamide (5e). General Procedure B. $FeBr_2$ (0.25 equiv) instead of $FeCl_3$ (0.25 equiv) was used. The product (54.3 mg, 58% yield) was purified with silica gel chromatography (Petroleum ether/EtOAc = 3:1) as a white solid. $[\alpha]_D^{20}$ = -48.88 (c = 0.61, $CHCl_3$) for a sample with 87% ee. **^{1}H NMR** (400 MHz, $CDCl_3$) δ 7.94 (d, J = 8.3 Hz, 2 H), 7.87 – 7.77 (m, 2 H), 7.76 – 7.66 (m, 2 H), 7.54 (d, J = 8.3 Hz, 2 H), 5.12 (s, 2 H), 3.65 – 3.48 (m, 3 H), 2.11 – 1.94 (m, 2 H), 1.60 – 1.36 (m, 2 H), 0.90 (s, 3 H), 0.85 (s, 3 H). **^{13}C NMR** (101 MHz, $CDCl_3$) δ 168.2, 141.8, 141.5, 134.0, 132.0, 130.2, 126.9, 126.6 (q, C-F, $^1J_{C-F}$ = 280.3 Hz), 123.2, 46.4 (q, C-F, $^2J_{C-F}$ = 26.8 Hz), 39.9, 39.5, 33.8, 32.8, 27.6, 27.4. **^{19}F NMR** (376 MHz, $CDCl_3$) δ -69.90

(d, J = 9.5 Hz, 3 F). MS (ESI): m/z (%) 491 (100) $[M+Na]^+$. HRMS (ESI) m/z: $[M+Na]^+$ Calcd. for $C_{22}H_{23}N_2O_4F_3NaS$: 491.1223; Found: 491.1221. IR (thin film) ν_{max} 3351, 3267, 2960, 2873, 1709, 1404, 1339, 1255, 1165, 1107, 721 cm^{-1}. Enantiomeric purity (87% ee) was measured by chiral HPLC on IG column (Hexane/^{i}PrOH = 6:4, 0.7 mL/min, UV detection at 214 nm); retention time = 12.59 min (major), retention time = 11.80 min (minor).

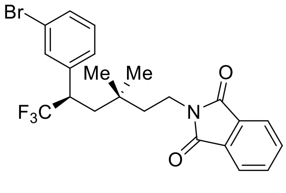

(*R*)-2-(5-(3-Bromophenyl)-6,6,6-trifluoro-3,3-dimethylhexyl)isoindoline-1,3-dione (5f). General Procedure B. The product (49.6 mg, 53% yield) was purified with silica gel chromatography (Petroleum ether/EtOAc = 15:1). An analytical pure sample was purified with reversed-phase HPLC, the pure product was obtained as a white solid (m.p. 117.8-118.8 °C). $[\alpha]_D^{20}$ = -35.50 (c = 1.11, $CHCl_3$) for a sample with 88% ee. **^{1}H NMR** (400 MHz, $CDCl_3$) δ 7.87 – 7.81 (m, 2 H), 7.75 – 7.68 (m, 2 H), 7.51 (t, J = 2.1 Hz, 1 H), 7.45 (ddd, J = 7.8, 2.0, 1.0 Hz, 1 H), 7.30 (d, J = 8.5 Hz, 1 H), 7.23 (t, J = 7.8 Hz, 1 H), 3.73 – 3.57 (m, 2 H), 3.44 (pd, J = 9.8, 2.1 Hz, 1 H), 2.09 – 1.89 (m, 2 H), 1.65 – 1.56 (m, 1 H), 1.56 – 1.46 (m, 1 H), 0.87 (s, 3 H), 0.82 (s, 3 H). **^{13}C NMR** (101 MHz, $CDCl_3$) δ 168.2, 138.7 (q, C-F, $^3J_{C-F}$ = 2.3 Hz), 133.9, 132.3, 132.2, 131.3, 130.2, 128.1, 126.8 (q, C-F, $^1J_{C-F}$ = 279.8 Hz), 123.2, 122.7, 46.1 (q, C-F, $^2J_{C-F}$ = 26.8 Hz), 40.0, 39.9, 33.9, 32.9, 27.4, 27.1. **^{19}F NMR** (376 MHz, $CDCl_3$) δ -70.18 (d, J = 9.5 Hz, 3 F). MS (FT): m/z (%) 468, 470 (100) $[M+H]^+$. HRMS (DART) m/z: $[M+H]^+$ Calcd. for $C_{22}H_{22}NO_2F_3Br$: 468.0781; Found: 468.0775. IR (thin film) ν_{max} 2959, 2872, 1713, 1468, 1401, 1254, 1160, 1108, 714 cm^{-1}. Enantiomeric purity (88% ee) was measured by chiral HPLC on ADH column (Hexane/^{i}PrOH = 97:3, 0.7 mL/min, UV detection at 214 nm); retention time = 14.60 min (major), retention time = 13.21 min (minor).

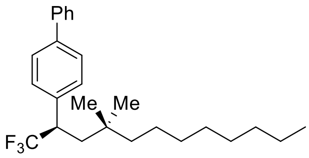

(*R*)-4-(1,1,1-Trifluoro-4,4-dimethyldodecan-2-yl)-1,1'-biphenyl (5g). General Procedure B. NaI (0.5 equiv) instead of $FeCl_3$ (0.25 equiv) was used. The product (44.6 mg, 55% yield) was purified with silica gel chromatography (Petroleum ether) as a white solid (m.p. 43.1-44.3 °C). $[\alpha]_D^{20}$ = -20.06 (c = 1.22, $CHCl_3$) for a sample with 91% ee. **^{1}H NMR** (400 MHz, $CDCl_3$) δ 7.64 – 7.50 (m, 4 H), 7.49 – 7.31 (m, 5 H), 3.34 (qt, J = 9.8, 4.8 Hz, 1 H), 2.04 – 1.83 (m, 2 H), 1.29 – 0.99 (m, 14 H), 0.85 (t, J = 6.9

Hz, 3 H), 0.78 (s, 3 H), 0.77 (s, 3 H). **^{13}C NMR** (101 MHz, $CDCl_3$) δ 140.8, 140.5, 135.9 (q, C-F, $^{3}J_{C-F}$ = 2.3 Hz), 129.8, 128.7, 127.5 (q, C-F, $^{1}J_{C-F}$ = 279.8 Hz), 127.4, 127.2, 127.0, 46.3 (q, C-F, $^{2}J_{C-F}$ = 26.3 Hz), 42.1, 39.9, 33.3, 31.9, 30.4, 29.6, 29.3, 27.7, 27.5, 23.8, 22.6, 14.1. **^{19}F NMR** (376 MHz, $CDCl_3$) δ -70.15 (d, J = 9.5 Hz, 3 F). MS (FT): m/z (%) 404 (67) [M]$^+$. HRMS (DART) m/z: [M]$^+$ Calcd. for $C_{26}H_{35}F_3$: 404.2685; Found: 404.2684. IR (thin film) v_{max} 3346, 2950, 2931, 2870, 2847, 1489, 1466, 1314, 1246, 1172, 1122, 736 cm^{-1}. Enantiomeric purity (91% ee) was measured by chiral HPLC on PC-3 column (MeCN/H_2O = 65:35, 0.7 mL/min, UV detection at 214 nm); retention time = 40.51 min (major), retention time = 37.57 min (minor).

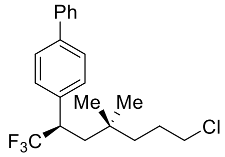

(*R*)-4-(7-Chloro-1,1,1-trifluoro-4,4-dimethylheptan-2-yl)-1,1'-biphenyl (5h). General Procedure B. NaI (0.5 equiv) instead of $FeCl_3$ (0.25 equiv) was used. The product (39.6 mg, 54% yield) was purified with silica gel chromatography (Petroleum ether) as a white solid. $[\alpha]_D^{20}$ = -20.24 (c = 0.51, $CHCl_3$) for a sample with 88% ee. **^{1}H NMR** (400 MHz, $CDCl_3$) δ 7.64 – 7.52 (m, 4 H), 7.49 – 7.31 (m, 5 H), 3.43 – 3.21 (m, 3 H), 2.05 – 1.87 (m, 2 H), 1.75 – 1.62 (m, 1 H), 1.61 – 1.56 (m, 1 H), 1.34 – 1.24 (m, 1 H), 1.23 – 1.11 (m, 1 H), 0.82 (s, 3 H), 0.81 (s, 3 H). **^{13}C NMR** (101 MHz, $CDCl_3$) δ 141.0, 140.4, 135.5, 129.7, 128.8, 127.5, 127.3, 127.2 (q, C-F, $^{1}J_{C-F}$ = 280.1 Hz), 127.1, 46.3 (q, C-F, $^{2}J_{C-F}$ = 26.6 Hz), 45.5, 39.8, 39.1, 33.1, 27.6, 27.4, 27.4.**^{19}F NMR** (376 MHz, $CDCl_3$) δ -70.17 (d, J = 9.5 Hz, 3 F). MS (FT): m/z (%) 368 (100) [M]$^+$, 391 (51) [M+Na]$^+$. HRMS (DART) m/z: [M]$^+$ Calcd. for $C_{21}H_{24}F_3Cl$: 368.1513; Found: 368.1513. IR (thin film) v_{max} 2958, 2875, 1489, 1442, 1372, 1257, 1162, 1106, 766 cm^{-1}. Enantiomeric purity (88% ee) was measured by chiral HPLC on ADH column (Hexane/^{i}PrOH = 97:3, 0.7 mL/min, UV detection at 214 nm); retention time = 5.67 min (major), retention time = 5.35 min (minor).

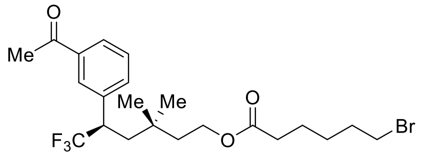

(*R*)-5-(3-Acetylphenyl)-6,6,6-trifluoro-3,3-dimethylhexyl 6-bromohexanoate (5i). General Procedure B. NaI (0.5 equiv) instead of $FeCl_3$ (0.25 equiv) was used. The product (53.9 mg, 56% yield) was purified with silica gel chromatography (Petroleum ether/EtOAc = 8:1) as a colorless oil. $[\alpha]_D^{20}$ = -11.88 (c = 0.35, $CHCl_3$) for a sample with 90% ee. **^{1}H NMR** (400 MHz, $CDCl_3$) δ 7.96 – 7.88 (m, 2 H), 7.56 (d, J = 7.7 Hz, 1 H), 7.48 (t, J = 7.6 Hz, 1 H), 4.13 – 3.92 (m, 2 H), 3.51 – 3.34 (m, 3

H), 2.63 (s, 3 H), 2.27 (t, J = 7.4 Hz, 2 H), 2.07 – 1.94 (m, 2 H), 1.87 (p, J = 6.8 Hz, 2 H), 1.67 – 1.57 (m, 2 H), 1.55 – 1.38 (m, 4 H), 0.82 (s, 3 H), 0.79 (s, 3 H). **^{13}C NMR** (101 MHz, $CDCl_3$) δ 197.5, 173.4, 137.5, 137.3, 133.8, 129.1, 129.0, 128.3, 126.9 (q, C-F, $^{1}J_{C-F}$ = 280.0 Hz), 68.0, 61.1, 46.5 (q, C-F, $^{2}J_{C-F}$ = 26.3 Hz), 40.6, 40.1, 34.1, 33.4, 32.8, 32.4, 27.6, 27.6, 27.3, 26.7, 25.6, 24.0. **^{19}F NMR** (376 MHz, $CDCl_3$) δ -70.23 (d, J = 9.5 Hz, 3 F). MS (FT): m/z (%) 479 (98) $[M+H]^+$, 481 (100) $[M+H+2]^+$, 496 (88) $[M+NH_4]^+$, 498 (86) $[M+NH_4+2]^+$. HRMS (DART) m/z: $[M+H]^+$ Calcd. for $C_{22}H_{31}O_3F_3Br$: 479.1403; Found: 479.1397. IR (thin film) ν_{max} 2961, 2870, 1732, 1687, 1466, 1442, 1361, 1255, 1164, 1108, 701 cm^{-1}. Enantiomeric purity (90% ee) was measured by chiral HPLC on IG column (Hexane/^{i}PrOH = 95:5, 0.7 mL/min, UV detection at 214 nm); retention time = 20.09 min (major), retention time = 22.03 min (minor).

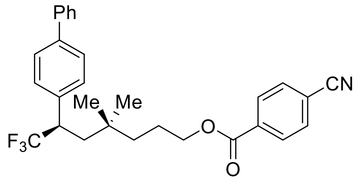

(*R*)-6-([1,1'-Biphenyl]-4-yl)-7,7,7-trifluoro-4,4-dimethylheptyl 4-cyanobenzoate (5j). General Procedure B. No additive was used. The product (65.2 mg, 68% yield) was purified with silica gel chromatography (Petroleum ether/EtOAc = 10:1) as a colorless oil. $[\alpha]_D^{20}$ = -22.36 (c = 0.73, $CHCl_3$) for a sample with 87% ee. **^{1}H NMR** (400 MHz, $CDCl_3$) δ 8.06 (d, J = 8.4 Hz, 2 H), 7.69 (d, J = 8.4 Hz, 2 H), 7.59 – 7.52 (m, 4 H), 7.46 – 7.32 (m, 5 H), 4.23 – 4,06 (m, 2 H), 3.45 – 3.28 (m, 1 H), 2.06 – 1.91 (m, 2 H), 1.76 – 1.62 (m, 1 H), 1.57 – 1.47 (m, 1 H), 1.34 – 1.13 (m, 2 H), 0.86 (s, 3 H), 0.83 (s, 3 H). **^{13}C NMR** (101 MHz, $CDCl_3$) δ 164.8, 140.9, 140.3, 135.5 (q, C-F, $^{3}J_{C-F}$ = 1.8 Hz), 134.1, 132.1, 123.0, 129.7, 128.8, 127.5, 127.2, 127.2 (q, C-F, $^{1}J_{C-F}$ = 279.9 Hz), 127.0, 118.0, 116.3, 66.2, 46.3 (q, C-F, $^{2}J_{C-F}$ = 26.3 Hz), 40.0, 38.0, 33.1, 27.4, 27.3, 23.3. **^{19}F NMR** (376 MHz, $CDCl_3$) δ -70.18 (d, J = 10.2 Hz, 3 F). MS (FT): m/z (%) 479 (100) $[M]^+$ HRMS (DART) m/z: $[M]^+$ Calcd for $C_{29}H_{28}NO_2F_3$: 479.2067; Found: 479.2059. IR (thin film) ν_{max} 2960, 2853, 2228, 1721, 1474, 1311, 1179, 1106, 766 cm^{-1}. Enantiomeric purity (87% ee) was measured by chiral HPLC on ADH column (Hexane/^{i}PrOH = 95:5, 0.7 mL/min, UV detection at 214 nm); retention time = 15.52 min (major), retention time = 13.37 min (minor).

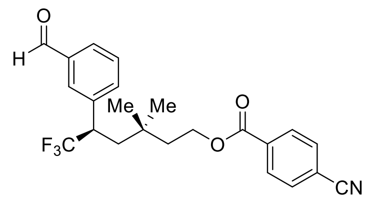

(*R*)-6,6,6-Trifluoro-5-(3-formylphenyl)-3,3-dimethylhexyl 4-cyanobenzoate (5k). General Procedure B. No additive was used. The product (45.8 mg, 55% yield) was purified with silica gel chromatography (Petroleum ether/EtOAc = 8:1) as a white solid (m.p. 79.1-80.6 °C). $[\alpha]_D^{20}$ = -15.74 (*c* = 0.47, $CHCl_3$) for a sample with 84% ee. **^{1}H NMR** (400 MHz, $CDCl_3$) δ 10.02 (s, 1 H), 8.08 (d, *J* = 8.5 Hz, 2 H), 7.88 (s, 1 H), 7.83 (d, *J* = 7.5 Hz, 1H), 7.75 (d, *J* = 8.1 Hz, 2 H), 7.63 (d, *J* = 7.7 Hz, 1 H), 7.55 (t, *J* = 7.6 Hz, 1 H), 4.40 – 4.31 (m, 1 H), 4.30 – 4.21 (m, 1H), 3.55 – 3.43 (m, 1 H), 2.13 – 2.01 (m, 2 H), 1.75 – 1.61 (m, 2 H), 0.90 (s, 3 H), 0.85 (s, 3 H). **^{13}C NMR** (101 MHz, $CDCl_3$) δ 191.7, 164.8, 137.7 (q, C-F, $^3J_{C-F}$ = 1.7 Hz), 136.8, 135.2, 133.9, 132.2, 130.0, 130.0, 130.0, 129.6, 126.7 (q, C-F, $^1J_{C-F}$ = 279.9 Hz), 117.9, 116.5, 62.4, 46.4 (q, C-F, $^2J_{C-F}$ = 26.3 Hz), 40.7, 40.1, 32.9, 27.6, 27.2. **^{19}F NMR** (376 MHz, $CDCl_3$) δ -70.23 (d, *J* = 9.5 Hz, 3 F). MS (FT): m/z (%) 440 (100) $[M+Na]^+$ HRMS (DART) m/z: $[M+Na]^+$ Calcd. for $C_{23}H_{22}NO_3F_3Na$: 440.1444; Found: 440.1441. IR (thin film) ν_{max} 3029, 2818, 2727, 1694, 1599, 1466, 1290, 1203, 1163, 783, 690 cm^{-1}. Enantiomeric purity (84% ee) was measured by chiral HPLC on ADH column (Hexane/^{i}PrOH = 95:5, 0.7 mL/min, UV detection at 214 nm); retention time = 36.84 min (major), retention time = 34.36 min (minor).

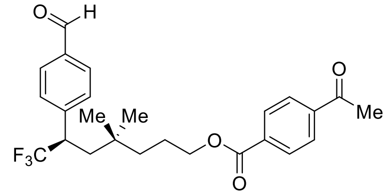

(*R*)-7,7,7-Trifluoro-6-(4-formylphenyl)-4,4-dimethylheptyl 4-acetylbenzoate (5l). General Procedure B. The product (48.7 mg, 54% yield) was purified with silica gel chromatography (Petroleum ether/EtOAc = 6:1) as a colorless oil. $[\alpha]_D^{20}$ = -27.20 (*c* = 0.82, $CHCl_3$) for a sample with 90% ee. **^{1}H NMR** (400 MHz, $CDCl_3$) δ 10.00 (s, 1 H), 8.09 (d, *J* = 8.2 Hz, 2 H), 8.02 (d, *J* = 8.3 Hz, 2 H), 7.86 (d, *J* = 8.0 Hz, 2 H), 7.52 (d, *J* = 7.8 Hz, 2 H), 4.19 (t, *J* = 6.5 Hz, 2 H), 3.53 – 3.34 (m, 1 H), 2.66 (s, 3 H), 2.04 – 1.94 (m, 2 H), 1.79 – 1.66 (m, 1 H), 1.65 – 1.51 (m, 1 H), 1.36 – 1.15 (m, 2 H), 0.83 (s, 3 H), 0.78 (s, 3 H). **^{13}C NMR** (101 MHz, $CDCl_3$) δ 197.5, 191.5, 165.6, 143.4 (q, C-F, $^3J_{C-F}$ = 2.3 Hz), 140.2, 136.1, 134.0, 130.1, 129.9, 129.7, 128.2, 126.7 (q, C-F, $^1J_{C-F}$ = 280.0 Hz), 65.7, 46.8 (q, C-F, $^2J_{C-F}$ = 26.3 Hz), 40.1, 38.2, 33.1, 27.2, 27.2, 26.9, 23.3. **^{19}F NMR** (376 MHz, $CDCl_3$) δ -69.86 (d, *J* = 9.5 Hz, 3 F). MS (FT): m/z (%) 449 (100) $[M+H]^+$, 462 (90) $[M+NH_4]^+$. HRMS (DART) m/z: $[M+H]^+$ Calcd. for $C_{25}H_{28}O_4F_3$: 449.1934; Found: 449.1929. IR (thin film) ν_{max}

2959, 2872, 1707, 1690, 1609, 1472, 1311, 1260, 1107, 770 cm^{-1}. Enantiomeric purity (90% ee) was measured by chiral HPLC on OJH column (Hexane/^{i}PrOH = 6:4, 0.7 mL/min, UV detection at 214 nm); retention time = 57.46 min (major), retention time = 29.73 min (minor).

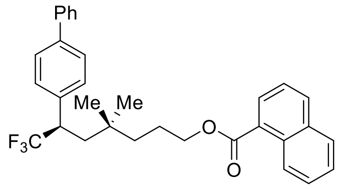

(*R*)-6-([1,1'-Biphenyl]-4-yl)-7,7,7-trifluoro-4,4-dimethylheptyl 1-naphthoate (5m). General Procedure B. The product (62.3 mg, 62% yield) was purified with silica gel chromatography (Petroleum ether/EtOAc = 15:1) as a colorless oil. $[\alpha]_D^{20}$ = -19.91 (*c* = 1.16, $CHCl_3$) for a sample with 87% ee. **^{1}H NMR** (400 MHz, $CDCl_3$) δ 8.93 – 8.82 (m, 1 H), 8.18 – 8.08 (m, 1 H), 8.01 (d, *J* = 8.4 Hz, 1 H), 7.88 (d, *J* = 7.9 Hz, 1 H), 7.64 – 7.44 (m, 7 H), 7.44 – 7.29 (m, 5 H), 4.32 – 4.16 (m, 2 H), 3.46 – 3.29 (m, 1 H), 2.07 – 1.92 (m, 2 H), 1.82 – 1.69 (m, 1 H), 1.68 – 1.56 (m, 1 H), 1.43 – 1.21 (m, 2 H), 0.85 (s, 3 H), 0.84 (s, 3 H). **^{13}C NMR** (101 MHz, $CDCl_3$) δ 167.5, 140.9, 140.4, 135.6 (q, C-F, $^3J_{C-F}$ = 1.9 Hz), 133.8, 133.3, 131.3, 130.0, 129.7, 128.7, 128.5, 127.7, 127.4, 127.4, 127.3, 127.2 (q, C-F, $^1J_{C-F}$ = 279.9 Hz), 127.0, 126.2, 125.8, 124.5, 65.5, 46.3 (q, C-F, $^2J_{C-F}$ = 26.1 Hz), 40.0, 38.2, 33.2, 27.5, 27.4, 23.5. **^{19}F NMR** (376 MHz, $CDCl_3$) δ -70.16 (d, *J* = 9.5 Hz, 3 F). MS (FT): m/z (%) 505 (60) $[M+H]^+$, 522 (100) $[M+ NH_4]^+$. HRMS (DART) m/z: $[M+H]^+$ Calcd. for $C_{32}H_{32}O_2F_3$: 505.2349; Found: 505.2344. IR (thin film) ν_{max} 3032, 2958, 2872, 1713, 1509, 1488, 1278, 1247, 1136, 1104, 784, 740 cm^{-1}. Enantiomeric purity (87% ee) was measured by chiral HPLC on ODH column (Hexane/^{i}PrOH = 95:5, 0.7 mL/min, UV detection at 214 nm); retention time = 11.96 min (major), retention time = 13.36 min (minor).

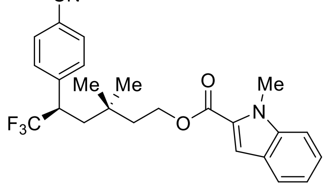

(*R*)-5-(4-Cyanophenyl)-6,6,6-trifluoro-3,3-dimethylhexyl 1-methyl-1*H*-indole-2-carboxylate (5n). General Procedure B. The product (44.6 mg, 53% yield) was purified with silica gel chromatography (Petroleum ether/EtOAc = 8:1) as a colorless oil. $[\alpha]_D^{20}$ = -33.34 (*c* = 0.76, $CHCl_3$) for a sample with 90% ee. **^{1}H NMR** (400 MHz, $CDCl_3$) δ 7.67 (t, *J* = 8.6 Hz, 3 H), 7.48 (d, *J* = 8.0 Hz, 2 H), 7.42 – 7.33 (m, 2 H), 7.22 (s, 1 H), 7.16 (t, *J* = 7.2 Hz, 1 H), 4.32 (dt, *J* = 11.2, 7.2 Hz, 1 H), 4.23 (dt, *J* = 11.2, 7.3 Hz, 1 H), 4.07 (s, 3 H), 3.54 – 3.40 (m, 1 H), 2.13 – 1.97 (m, 2 H), 1.74 – 1.59 (m, 2 H), 0.91 (s, 3 H), 0.84 (s, 3 H). **^{13}C NMR** (101 MHz, $CDCl_3$) δ 162.1, 141.9 (q, C-F, $^3J_{C-F}$ = 2.3 Hz),

139.7, 132.5, 130.2, 127.6, 126.5 (q, C-F, $^{1}J_{C-F}$ = 280.0 Hz), 125.8, 125.1, 122.6, 120.6, 118.2, 112.4, 110.3, 110.2, 61.0, 46.7 (q, C-F, $^{2}J_{C-F}$ = 26.8 Hz), 40.7 (q, C-F, $^{3}J_{C-F}$ = 1.8 Hz), 40.3, 32.9, 31.6, 27.5, 27.2. **^{19}F NMR** (376 MHz, $CDCl_3$) δ -69.91 (d, J = 9.5 Hz, 3 F). MS (ESI): m/z (%) 465 (100) $[M+Na]^+$. HRMS (ESI) m/z: $[M+Na]^+$ Calcd. for $C_{25}H_{25}N_2O_2F_3Na$: 465.1760; Found: 465.1768. IR (thin film) ν_{max} 2961, 2874, 2231, 1709, 1518, 1470, 1404, 1252, 1166, 1107, 749 cm^{-1}. Enantiomeric purity (90% ee) was measured by chiral HPLC on OJH column (Hexane/^{i}PrOH = 7:3, 0.7 mL/min, UV detection at 214 nm); retention time = 52.13 min (major), retention time = 72.32 min (minor).

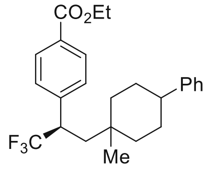

Ethyl (R)-4-(1,1,1-trifluoro-3-(1-methyl-4-phenylcyclohexyl)propan-2-yl)benzoate (5o). General Procedure B. $FeBr_2$ (0.25 equiv) instead of $FeCl_3$ (0.25 equiv) was used. The product (43.7 mg, 52% yield) was purified with silica gel chromatography (Petroleum ether/EtOAc = 50:1) as a a mixture of *cis* and *trans* isomer (colorless oil). **^{1}H NMR** (400 MHz, $CDCl_3$) δ 8.09 – 7.98 (m, 4 H), 7.45 (t, J = 8.4 Hz, 4 H), 7.32 – 7.24 (m, 4 H), 7.22 – 7.12 (m, 6 H), 4.43 – 4.32 (m, 4 H), 3.58 – 3.46 (m, 1 H), 3.45 – 3.32 (m, 1 H), 2.49 – 2.27 (m, 2 H), 2.22 – 2.09 (m, 2 H), 2.04 – 1.93 (m, 2 H), 1.78 – 1.49 (m, 8 H), 1.42 – 1.06 (m, 14 H), 0.87 (s, 3 H), 0.75 (s, 3 H). **^{13}C NMR** (126 MHz, $CDCl_3$) δ 166.2, 166.1, 147.1, 147.0, 142.0, 141.7, 130.3, 130.2, 129.8, 129.4, 129.4, 128.3, 128.3, 127.0 (q, C-F, $^{1}J_{C-F}$ = 279.8 Hz), 126.9 (q, C-F, $^{1}J_{C-F}$ = 279.8 Hz), 126.7, 126.7, 126.0, 125.9, 61.0, 46.5 (q, C-F, $^{2}J_{C-F}$ = 26.4 Hz), 46.0 (q, C-F, $^{2}J_{C-F}$ = 26.4 Hz), 44.3, 44.2, 43.9, 38.2, 38.1, 38.1, 38.0, 34.5, 32.9, 32.5, 29.8, 29.5, 29.4, 29.2, 29.1, 21.6, 14.3, 14.3. **^{19}F NMR** (376 MHz, $CDCl_3$) δ -69.89 (d, J = 10.2 Hz), -70.00 (d, J = 9.5 Hz). MS (FT): m/z (%) 436 (100) $[M+NH_4]^+$. HRMS (DART) m/z: $[M+H]^+$ Calcd. for $C_{25}H_{30}O_2F_3$: 419.2192; Found: 419.2193. IR (thin film) ν_{max} 3027, 2926, 2850, 1720, 1614, 1493, 1452, 1368, 1279, 1157, 1106, 712, 700 cm^{-1}. Enantiomeric purity (one isomer 90% ee, the other isomer 89% ee) was measured by chiral HPLC on ODH column (Hexane/^{i}PrOH = 98:2, 0.7 mL/min, UV detection at 214 nm); One isomer: retention time (isomer 1) = 8.50 min (major), retention time = 7.94 min (minor); The other isomer: retention time (isomer 2) = 9.41 min (major), retention time = 14.21 min (minor).

6. Transformations of compound 5b

6.1 Phthalimide deprotection

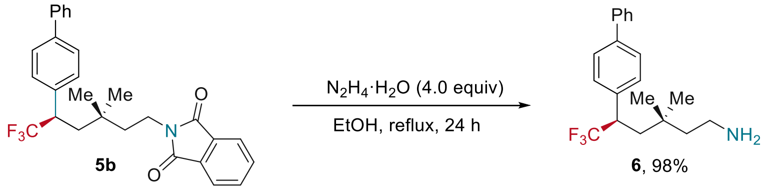

(*R*)-5-([1,1'-Biphenyl]-4-yl)-6,6,6-trifluoro-3,3-dimethylhexan-1-amine (6). To a suspension of **5b** (465.5 mg, 1 mmol, 1 equiv) in 10 mL ethanol was added hydrazine monohydrate (0.25 mL, 4 mmol, 4 equiv) at room temperature. The mixture was heated under reflux and monitored by TLC. After completion, the reaction was cooled to room temperature, and the resulting precipitates were filtered off and washed with ethanol. The filtrate was concentrated and dried under vacumm to give compound **6** as a yellow solid (328.1 mg, 98% yield, m.p. 113.0-115.0 °C) without further purification. **^{1}H NMR** (400 MHz, $CDCl_3$) δ 7.66 – 7.49 (m, 4 H), 7.47 – 7.27 (m, 5 H), 3.37 (pd, J = 9.8, 2.6 H, 1 H), 2.60 (td, J = 11.5, 5.5 Hz, 1 H), 2.50 (td, J = 11.5, 5.0 Hz, 1 H), 2.05 – 1.87 (m, 2 H), 1.40 – 1.18 (m, 2 H), 1.04 (br, 2 H), 0.79 (s, 6 H). **^{13}C NMR** (101 MHz, $CDCl_3$) δ 140.9, 140.3, 135.6 (q, C-F, $^3J_{C\text{-}F}$ = 2.3 Hz), 129.7, 128.8, 127.4, 127.2, 127.2 (q, C-F, $^1J_{C\text{-}F}$ = 279.9 Hz), 127.0, 46.2 (q, C-F, $^2J_{C\text{-}F}$ = 26.1 Hz), 45.7, 40.2, 37.4, 32.9, 27.7, 27.5. **^{19}F NMR** (376 MHz, $CDCl_3$) δ -70.12 (d, J = 9.9 Hz, 3 F). MS (ESI): m/z (%) 336 (100) $[M+H]^+$. HRMS (ESI) m/z: $[M+H]^+$ Calcd. for $C_{20}H_{25}NF_3$: 336.1934; Found: 336.1937. IR (thin film) ν_{max} 3363, 2958, 2872, 1653, 1489, 1393, 1255, 1152, 1104 cm^{-1}.

6.2 Synthesis of compound 7a

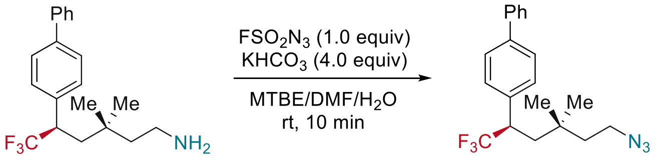

(*R*)-4-(6-Azido-1,1,1-trifluoro-4,4-dimethylhexan-2-yl)-1,1'-biphenyl (**7a**). Compound **6** (33.5 mg, 0.1 mmol, 1.0 equiv), FSO_2N_3 (0.17 M in DMF/MTBE 1:1, 0.6 mL, 0.1 mmol, 1.0 equiv), and aqueous $KHCO_3$ (3 M in H_2O, 0.14 mL, 0.4 mmol, 4.0 equiv) were added to a 15 mL vail. The mixture was stirred at room temperature for 10 min, then 30 mL of EtOAc was added and the mixture was washed sequentially with brine (60 mL × 6), water (60 mL × 2) and brine (60 mL), dried over Na_2SO_4 and concentrated. The product **7a** (28.2 mg, 78% yield) was purified with silica gel chromatography

(Petroleum ether/EtOAc = 100:1) as a colorless oil. **^{1}H NMR** (400 MHz, $CDCl_3$) δ 7.65 – 7.50 (m, 4 H), 7.48 – 7.30 (m, 5 H), 3.36 (pd, J = 9.9, 3.2 Hz, 1 H), 3.17 (ddd, J = 12.1, 9.3, 6.8 Hz, 1 H), 3.08 (ddd, J = 12.1, 9.2, 6.8 Hz, 1 H),2.04 – 1.91 (m, 2 H), 1.46 (ddd, J = 9.4, 6.6, 2.7 Hz, 2 H), 0.86 (s, 3 H), 0.84 (s, 3 H). **^{13}C NMR** (101 MHz, $CDCl_3$) δ 141.1, 140.4, 135.3 (q, C-F, $^{3}J_{C\text{-}F}$ = 2.3 Hz), 129.6, 128.8, 127.5, 127.4, 127.1 (q, C-F, $^{1}J_{C\text{-}F}$ = 279.5 Hz), 127.1, 47.3, 46.2 (q, C-F, $^{2}J_{C\text{-}F}$ = 26.6 Hz), 40.4 (q, C-F, $^{3}J_{C\text{-}F}$ = 1.8 Hz), 40.0, 32.8, 27.4, 27.3. **^{19}F NMR** (376 MHz, $CDCl_3$) δ -70.23 (d, J = 9.5 Hz, 3 F). MS (ESI): m/z (%) 379 (4) $[M+NH_4]^+$, 334 (100) $[M-N_3+NH_3]^+$. HRMS (ESI) m/z: $[M+NH_4]^+$ Calcd. for $C_{20}H_{26}N_4F_3$: 379.2104; Found: 379.2101. IR (thin film) ν_{max} 3032, 2960, 2874, 2096, 1486, 1372, 1255, 1160, 1104 cm^{-1}.

6.3 Synthesis of compound 7b

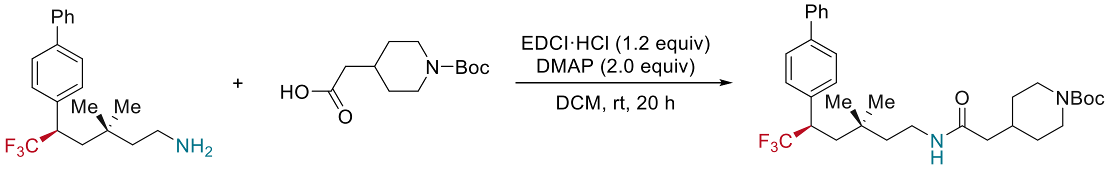

***tert*-Butyl (*R*)-4-(2-((5-([1,1'-biphenyl]-4-yl)-6,6,6-trifluoro-3,3-dimethylhexyl)amino)-2-oxoethyl)piperidine-1-carboxylate** (**7b**). To a mixture of 2-(1-(*tert*-butoxycarbonyl)piperidin-4-yl)acetic acid (0.12 mmol, 1.2 equiv) and 4-(dimethylamino)pyridine (0.2 mmol, 2 equiv) in 0.5 mL anhydrous CH_2Cl_2 were added *N*-(3-dimethylaminopropyl)-*N'*-ethylcarbodiimide hydrochloride (EDCI·HCl) (0.12 mmol, 1.2 equiv) and the amine **6** (0.1 mmol, 1 equiv) at room temperature. The mixture was stirred at room temperature for 20 h. The resulting solution was diluted with ethyl acetate and washed with water and brine. The organic layer was dried over anhydrous Na_2SO_4, filtered, and concentrated. The product **7b** (39.4 mg, 71% yield) was purified with silica gel chromatography (Petroleum ether/EtOAc = 3:2) as a colorless oil. **^{1}H NMR** (400 MHz, $CDCl_3$) δ 7.57 – 7.47 (m, 4 H), 7.40 – 7.31 (m, 4 H), 7.28 (t, J = 7.3 Hz, 1 H), 5.00 (s, 1 H), 3.96 (d, J = 13.5 Hz, 2 H), 3.32 (p, J = 9.6 Hz, 1 H), 3.32 – 3.19 (m, 1 H), 3.03 – 2.87 (m, 1 H), 2.66 – 2.50 (m, 2 H), 2.02 – 1.99 (m, 1 H), 1.98 – 1.76 (m, 4 H), 1.59 – 1.46 (m, 2 H), 1.38 (s, 9 H), 1.35 – 1.26 (m, 1 H), 1.24 – 1.09 (m, 1 H), 1.03 – 0.87 (m, 2 H), 0.79 (s, 3 H), 0.77 (s, 3 H). **^{13}C NMR** (101 MHz, $CDCl_3$) δ 171.3, 154.8, 140.9, 140.1, 135.5, 129.8, 128.8, 127.6, 127.2, 127.1 (q, C-F, $^{1}J_{C\text{-}F}$ = 280.0 Hz), 126.9, 79.3, 46.2 (q, C-F,

$^{2}J_{C\text{-}F}$ = 26.8 Hz), 43.6, 41.2, 39.8, 35.4, 33.3, 32.9, 31.8, 28.4, 27.5, 27.4. **^{19}F NMR** (376 MHz, $CDCl_3$) δ -70.18 (d, J = 10.2 Hz, 3 F). MS (FT): m/z (%) 505 (100) $[M\text{-}C_4H_8\text{+}H]^+$, 561 (17) $[M+H]^+$, 583 (48) $[M+Na]^+$. HRMS (ESI) m/z: $[M+Na]^+$ Calcd. for $C_{32}H_{43}N_2O_3F_3Na$: 583.3118; Found: 583.3117. IR (thin film) ν_{max} 3316, 2934, 2870, 1690, 1548, 1472, 1366, 1163, 1105 cm^{-1}.

6.4 Synthesis of compound 7c

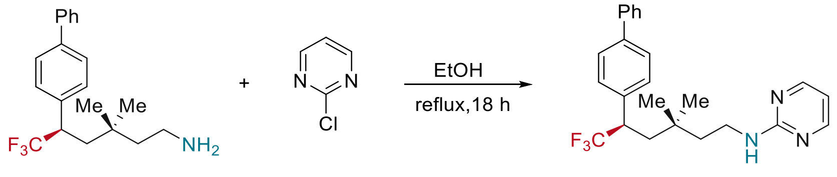

(*R*)-N-(5-([1,1'-Biphenyl]-4-yl)-6,6,6-trifluoro-3,3-dimethylhexyl)pyrimidin-2-amine (7c). A mixture of **6** (0.19 mmol, 1.9 equiv) and 2-chloropyrimidine (0.1 mmol, 1 equiv) in 1.0 mL anhydrous EtOH was heated under reflux for 18 h. After cooling to room temperature, the reaction mixture was concentrated under reduced pressure. The resulting residue was purified with silica gel chromatography (Petroleum ether/EtOAc = 3:1) to afford **7c** (27.8 mg, 67% yield) as a colorless oil. **^{1}H NMR** (400 MHz, $CDCl_3$) δ 8.24 (d, J = 4.9 Hz, 2 H), 7.62 – 7.49 (m, 4 H), 7.47 – 7.30 (m, 5 H), 6.48 (t, J = 4.8 Hz, 1 H), 5.07 – 4.92 (m, 1 H), 3.51 (pd, J = 9.6, 3.2 Hz, 1 H), 3.45 – 3.34 (m, 1 H), 3.29 – 3.14 (m, 1 H), 2.09 – 1.97 (m, 2 H), 1.55 (ddd, J = 13.4, 11.3, 5.3 Hz, 1 H), 1.40 (ddd, J = 13.3, 11.3, 5.1 Hz, 1 H), 0.87 (s, 3 H), 0.86 (s, 3 H). **^{13}C NMR** (126 MHz, $CDCl_3$) δ 162.1, 157.9, 140.9, 140.3, 135.5, 129.7, 128.7, 127.4, 127.3, 127.2 (q, C-F, $^{1}J_{C\text{-}F}$ = 279.9 Hz), 127.1, 110.4, 46.1 (q, C-F, $^{2}J_{C\text{-}F}$ = 26.6 Hz), 41.2, 40.1, 37.3, 32.9, 27.7, 27.6. **^{19}F NMR** (376 MHz, $CDCl_3$) δ -70.14 (d, J = 10.2 Hz, 3 F). MS (FT): m/z (%) 414 (100) $[M+H]^+$. HRMS (ESI) m/z: $[M+H]^+$ Calcd. for $C_{24}H_{27}N_3F_3$: 414.2152; Found: 414.2153.

III. Supplementary Discussion

7. Mechanistic studies

7.1 Radical inhibition experiments

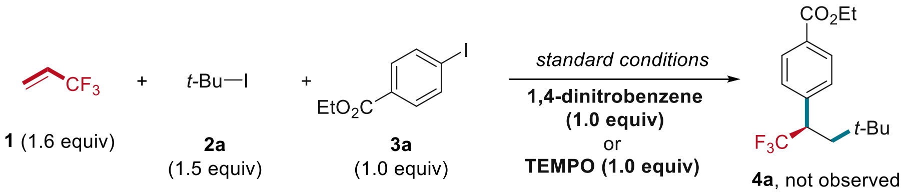

To a 25 mL of Schlenk tube were added Zn dust (39.2 mg, 0.6 mmol, 1.5 equiv), 1,4-dinitrobenzene (67.2 mg, 0.4 mmol, 1.0 equiv) or TEMPO (62.5 mg, 0.4 mmol, 1.0 equiv), **L6** (10.0 mg, 0.04 mmol, 10 mol%). The tube was then transferred to a glovebox, and $NiBr_2$·DME (12.4 mg, 0.04 mmol, 10 mol%) was added. The tube was then taken out of the glovebox and purge with Ar for three times, evacuated and backfilled with Ar (3 times). *tert*-Butyl iodide **2a** (110.4 mg, 0.6 mmol, 1.5 equiv), ethyl 4-iodobenzoate **3a** (110.4 mg, 0.4 mmol, 1.0 equiv), anhydrous DME and TFP solution (0.54 mL, 1.2 M in DMA, 1.6 equiv) were added under Ar. The Schlenk tube was screw capped and stirred (800 rpm) for 12 h at room temperature. The yield was determined by ^{19}F NMR using benzotrifluoride as an internal standard.

7.2 Radical clock experiment

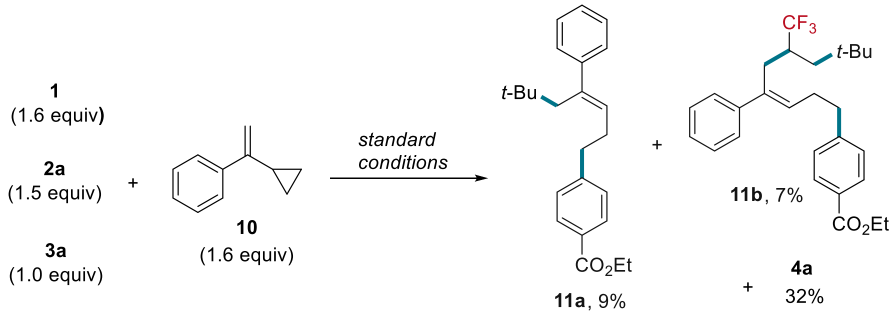

To a 25 mL of Schlenk tube were added Zn dust (39.2 mg, 0.6 mmol, 1.5 equiv), **L6** (10.0 mg, 0.04 mmol, 10 mol%). The tube was then transferred to a glovebox, $NiBr_2$·DME (12.4 mg, 0.04 mmol, 10 mol%) was added. The tube was then taken out of the glovebox, evacuated and backfilled with Ar (3 times). *tert*-Butyl iodide **2a** (110.4 mg, 0.6 mmol, 1.5 equiv), ethyl 4-iodobenzoate **3a** (110.4 mg, 0.4 mmol, 1.0 equiv), **10** (92.2 mg, 0.64 mmol, 1.6 equiv), anhydrous DME and TFP solution (0.54 mL, 1.2 M in DMA, 1.6 equiv) were added under Ar. The tube was sealed with a Teflon cap. After the reaction mixture was stirred at 800 rpm for 12 h at room temperature, 24 μL benzotrifluoride was

added. The yields of **4a** was determined by ^{19}F NMR using benzotrifluoride as an internal standard. The reaction mixture was filtered through a pad of Celite. The filtrate was extracted with EtOAc and washed with brine. The organic layer was dried over Na_2SO_4, filtered and concentrated. The residue was purified with silica gel chromatography to give the corresponding products as a mixture. The mixture was further purified with reverse-phase HPLC to give the corresponding products **4a**, **11a** and **11b**.

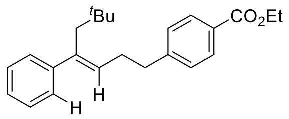

Ethyl (*E*)-4-(6,6-dimethyl-4-phenylhept-3-en-1-yl)benzoate (11a). Colorless oil (13.1 mg, 9% yield). **^{1}H NMR** (400 MHz, $CDCl_3$) δ 7.97 (d, J = 8.2 Hz, 2 H), 7.31 – 7.23 (m, 6 H), 7.23 – 7.16 (m, 1 H), 5.63 (t, J = 7.2 Hz, 1 H), 4.37 (q, J = 7.1 Hz, 2 H), 2.79 (t, J = 7.8 Hz, 2 H), 2.51 (q, J = 7.7 Hz, 2 H), 2.42 (s, 2 H), 1.39 (t, J = 7.1 Hz, 3 H), 0.74 (s, 9 H).**^{13}C NMR** (126 MHz, $CDCl_3$) δ 166.7, 147.4, 145.9, 140.0, 130.8, 129.7, 128.5, 128.2, 128.0, 126.7, 126.3, 60.8, 42.7, 36.0, 33.0, 31.2, 30.3, 14.4. MS (FI): m/z (%) 350 (17) $[M]^+$. HRMS (FI) m/z: $[M]^+$ Calcd. for $C_{24}H_{30}O_2$: 350.2240; Found: 350.2247. IR (thin film) ν_{max} 2954, 2927, 2866, 1720, 1611, 1465, 1365, 1275, 1106 cm^{-1}.

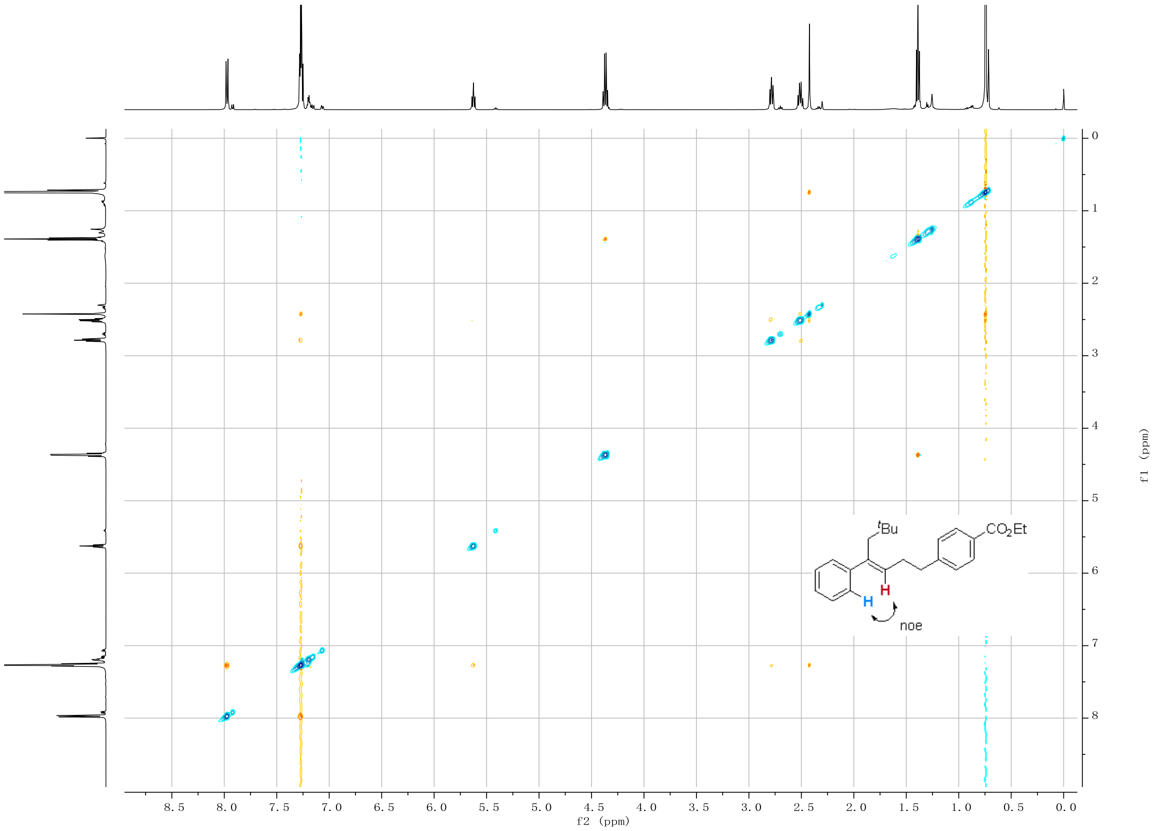

Supplementary Figure 3. NOE spectrum of compound **11a**

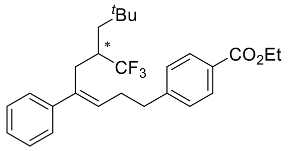

Ethyl (*E*)-4-(8,8-dimethyl-4-phenyl-6-(trifluoromethyl)non-3-en-1-yl)benzoate (11b). Colorless oil (13.5 mg, 7% yield). **^{1}H NMR** (600 MHz, $CDCl_3$) δ 8.00 – 7.97 (m, 2 H), 7.35 – 7.23 (m, 8 H), 5.69 (t, J = 7.3 Hz, 1 H), 4.37 (q, J = 7.1 Hz, 2 H), 2.86 – 2.76 (m, 3 H), 2.62 – 2.47 (m, 3 H), 2.06 – 1.96 (m, 1 H), 1.59 (dd, J = 14.7, 4.2 Hz, 1 H), 1.39 (t, J = 7.1 Hz, 3 H), 1.12 (dd, J = 14.9, 4.3 Hz, 1 H), 0.74 (s, 9 H). **^{13}C NMR** (126 MHz, $CDCl_3$) δ 166.7, 147.1, 141.8, 137.7, 130.1, 129.7, 128.6 (q, C-F, $^1J_{C\text{-}F}$ = 280.2 Hz), 128.5, 128.3, 128.3, 127.1, 126.8, 60.8, 42.1, 37.5 (q, C-F, $^2J_{C\text{-}F}$ = 24.3 Hz), 35.9, 31.3 (q, C-F, $^3J_{C\text{-}F}$ = 3.1 Hz), 30.5, 30.3, 29.4, 14.3. **^{19}F NMR** (376 MHz, $CDCl_3$) δ -69.79 (d, J = 8.9 Hz). MS (FI): m/z (%) 446 (8) [M]$^+$. HRMS (FI) m/z: [M]$^+$ Calcd. for $C_{27}H_{33}O_2F_3$: 446.2439; Found: 446.2432. IR (thin film) ν_{max} 2959, 2868, 1719, 1611, 1466, 1368, 1278, 1179, 1156, 1106 cm^{-1}.

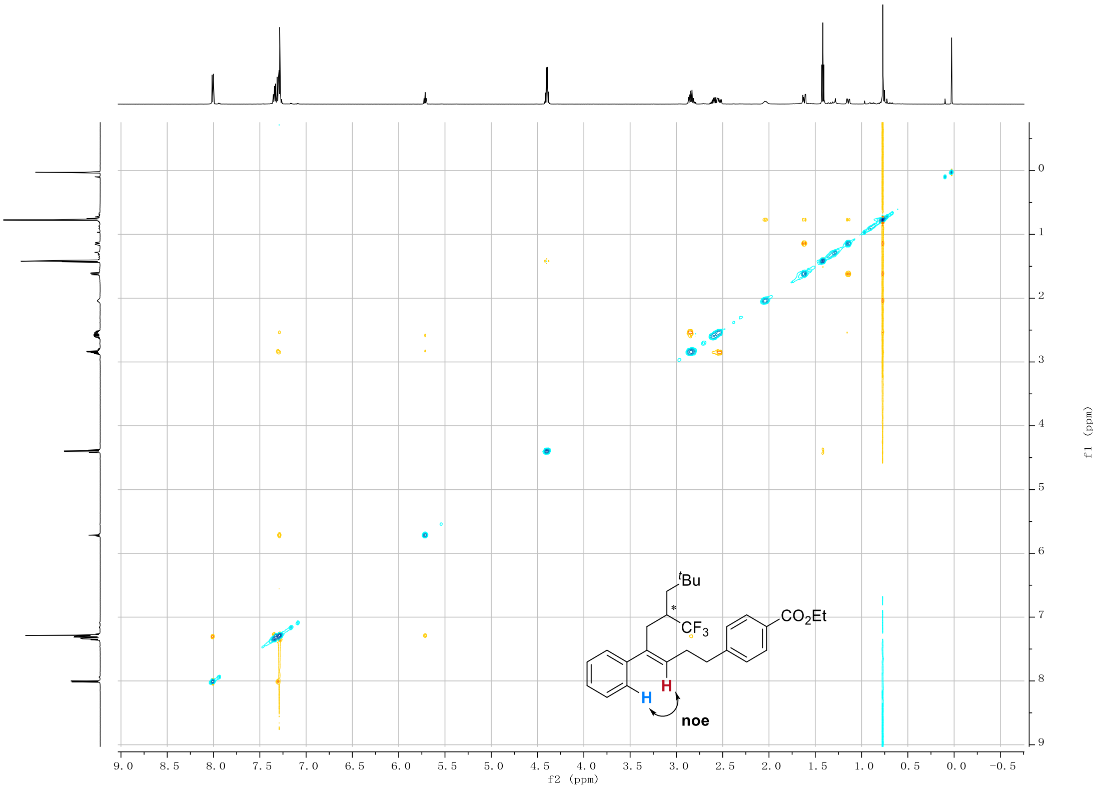

Supplementary Figure 4. NOE spectrum of compound **11b**

7.3 EPR experiments

7.3.1 Reaction of 1 with 2a and 3a in the presence of PBN under standard reaction conditions

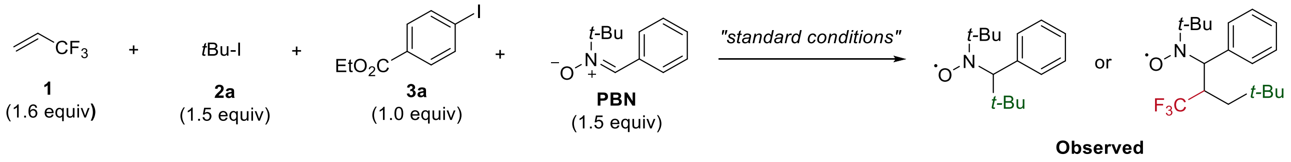

To a 25 mL of Schlenk tube were added Zn dust (39.2 mg, 0.6 mmol, 1.5 equiv), **L6** (10.0 mg, 0.04 mmol, 10 mol%) in the air. The tube was then moved to a glovebox. $NiBr_2$·DME (12.3 mg, 0.04 mmol, 10 mol%) and PBN (106.3 mg, 0.6 mmol, 1.5 equiv) were added to the tube in a glovebox. The tube was then taken out from the glovebox and purge with Ar for three times. *tert*-Butyl iodide **2a** (110.4 mg, 0.6 mmol, 1.5 equiv), ethyl 4-iodobenzoate **3a** (110.4 mg, 0.4 mmol, 1.0 equiv), anhydrous DME and TFP solution (0.54 mL, 1.2 M in DMA, 1.6 equiv) were added under Ar. The tube was sealed with a Teflon cap. After stirring at 800 rpm for 12 h at room temperature, the resulting mixture was analyzed by EPR. The EPR showed an e.p.r. spectrum of nitroxides.

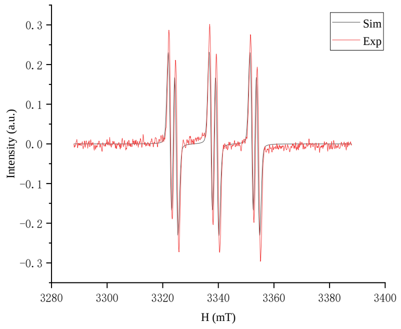

Supplementary Figure 5. The EPR spectrum of a mixture of PBN, **1**, **2a** and **3a** under standard reaction conditions.

7.3.2 Reaction of 1 with 2a and 3a in the presence of PBN without 2a.

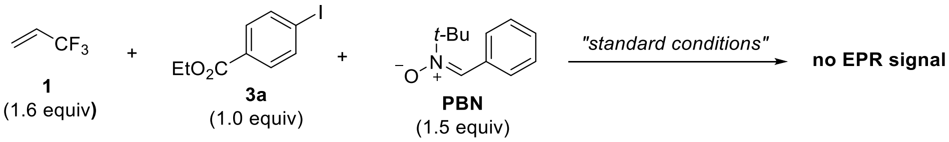

To a 25 mL of Schlenk were added **L6** (10.0 mg, 0.04 mmol, 10 mol%) in the air. The tube was then moved to a glovebox. $NiBr_2$·DME (12.3 mg, 0.04 mmol, 10 mol%) and PBN (106.3 mg, 0.6 mmol, 1.5 equiv) were added to the tube in a glovebox. The tube was then taken out from the glovebox and purge with Ar for three times. Ethyl 4-iodobenzoate **3a** (110.4 mg, 0.4 mmol, 1.0 equiv), anhydrous DME and TFP solution (0.54 mL, 1.2 M in DMA, 1.6 equiv) were added under Ar. The tube was sealed with a Teflon cap. After stirring at 800 rpm for 12 h at room temperature, the resulting mixture was analyzed by EPR. The EPR showed no e.p.r. signal of nitroxide.

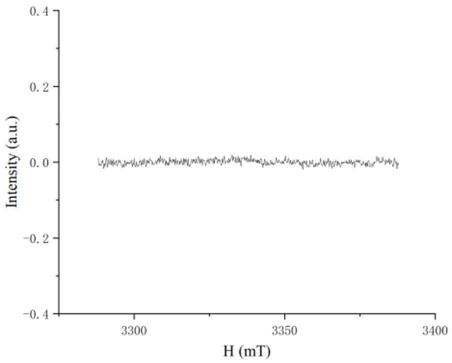

Supplementary Figure 6. The EPR spectrum of a mixture of PBN, **1** and **3a** under standard reaction conditions.

7.3.3 Reaction of 1 with 2a and 3a in the presence of PBN without Zn dust.

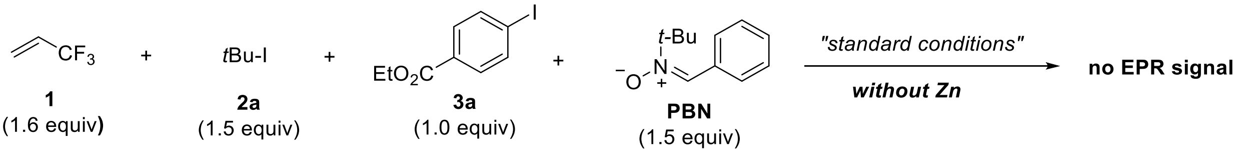

To a 25 mL of Schlenk were added **L6** (10.0 mg, 0.04 mmol, 10 mol%) in the air. The tube was moved to a glovebox. $NiBr_2$·DME (12.3 mg, 0.04 mmol, 10 mol%) and PBN (106.3 mg, 0.6 mmol, 1.5 equiv) were added to the tube in a glovebox. The tube was then taken out from the glovebox and purge with Ar for three times. *tert*-Butyl iodide **2a** (110.4 mg, 0.6 mmol, 1.5 equiv), ethyl 4-iodobenzoate **3a** (110.4 mg, 0.4 mmol, 1.0 equiv), anhydrous DME and TFP solution (0.54 mL, 1.2 M in DMA, 1.6 equiv) were added under Ar. The tube was sealed with Teflon cap. After stirring at 800 rpm for 12 h at room temperature, the resulting mixture was analyzed by EPR. The EPR showed no e.p.r. signal of nitroxide.

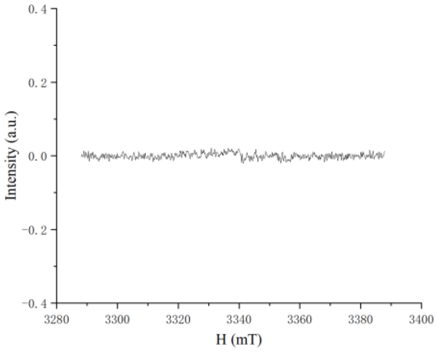

Supplementary Figure 7. The EPR spectrum of a mixture of PBN, **1**, **2a** and **3a** without Zn dust.

8. Crystal data and structure refinement for compounds 4i and 5b.

Compound 4i: (The crystal structure of compound **4i** has been deposited at the Cambridge Crystallographic Data Centre (**CCDC 2191969**). The data is available free of charge at www.ccdc.cam.ac.uk/conts/retrieving.html.)

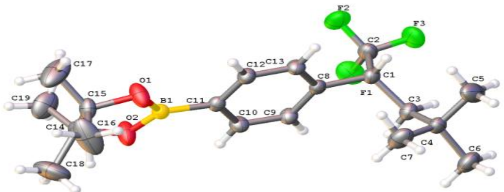

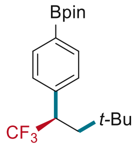

4i

Supplementary Table 11. Crystal data and structure refinement for compound **4i**.

Identification code	mj22213_0m	
Empirical formula	C19 H28 B F3 O2	
Formula weight	356.22	
Temperature	213.00 K	
Wavelength	1.34139 Å	
Crystal system	Orthorhombic	
Space group	$P2_12_12_1$	
Unit cell dimensions	a = 12.1099(2) Å	α= 90°.
	b = 12.4495(2) Å	β= 90°.
	c = 26.6762(5) Å	γ = 90°.
Volume	4021.76(12) $Å^3$	
Z	8	
Density (calculated)	1.177 Mg/m^3	
Absorption coefficient	0.491 mm^{-1}	
F(000)	1520	
Crystal size	0.07 x 0.07 x 0.05 mm^3	
Theta range for data collection	3.408 to 54.944°.	
Index ranges	-14<=h<=14, -15<=k<=11, -32<=l<=32	
Reflections collected	31897	
Independent reflections	7590 [R(int) = 0.0449]	
Completeness to theta = 53.594°	99.4 %	
Absorption correction	Semi-empirical from equivalents	
Max. and min. transmission	0.7508 and 0.6026	
Refinement method	Full-matrix least-squares on F^2	
Data / restraints / parameters	7590 / 0 / 465	
Goodness-of-fit on F^2	1.034	
Final R indices [I>2sigma(I)]	R1 = 0.0753, wR2 = 0.2116	
R indices (all data)	R1 = 0.0961, wR2 = 0.2347	
Absolute structure parameter	0.22(7)	
Extinction coefficient	n/a	
Largest diff. peak and hole	0.696 and -0.323 e.$Å^{-3}$	

Compound 5b: (The crystal structure of compound **5b** has been deposited at the Cambridge Crystallographic Data Centre (**CCDC 2191970**). The data is available free of charge at www.ccdc.cam.ac.uk/conts/retrieving.html.)

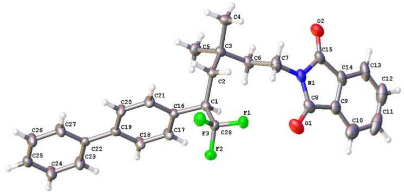

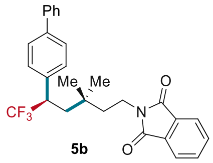

Supplementary Table 12. Crystal data and structure refinement for compound 5b

Identification code	mj22251_0m	
Empirical formula	C28 H26 F3 N O2	
Formula weight	465.50	
Temperature	213.00 K	
Wavelength	1.34139 Å	
Crystal system	Monoclinic	
Space group	P 1 21 1	
Unit cell dimensions	a = 7.88550(10) Å	α= 90°.
	b = 5.67580(10) Å	β= 95.6380(10)°.
	c = 26.4362(4) Å	γ = 90°.
Volume	1177.47(3) $Å^3$	
Z	2	
Density (calculated)	1.313 Mg/m^3	
Absorption coefficient	0.520 mm^{-1}	
F(000)	488	
Crystal size	0.07 x 0.07 x 0.05 mm^3	
Theta range for data collection	4.386 to 54.900°.	
Index ranges	-9<=h<=8, -6<=k<=6, -32<=l<=32	
Reflections collected	16216	
Independent reflections	4419 [R(int) = 0.0308]	
Completeness to theta = 53.594°	99.6 %	
Absorption correction	Semi-empirical from equivalents	
Max. and min. transmission	0.7508 and 0.6305	
Refinement method	Full-matrix least-squares on F^2	
Data / restraints / parameters	4419 / 2 / 309	
Goodness-of-fit on F^2	1.062	
Final R indices [I>2sigma(I)]	R1 = 0.0354, wR2 = 0.1182	
R indices (all data)	R1 = 0.0385, wR2 = 0.1242	
Absolute structure parameter	0.09(5)	
Extinction coefficient	n/a	
Largest diff. peak and hole	0.115 and -0.182 e.$Å^{-3}$	

IV. Supplementary Figures

9. Copies of ^{1}H, ^{13}C, ^{19}F NMR and HPLC Spectra

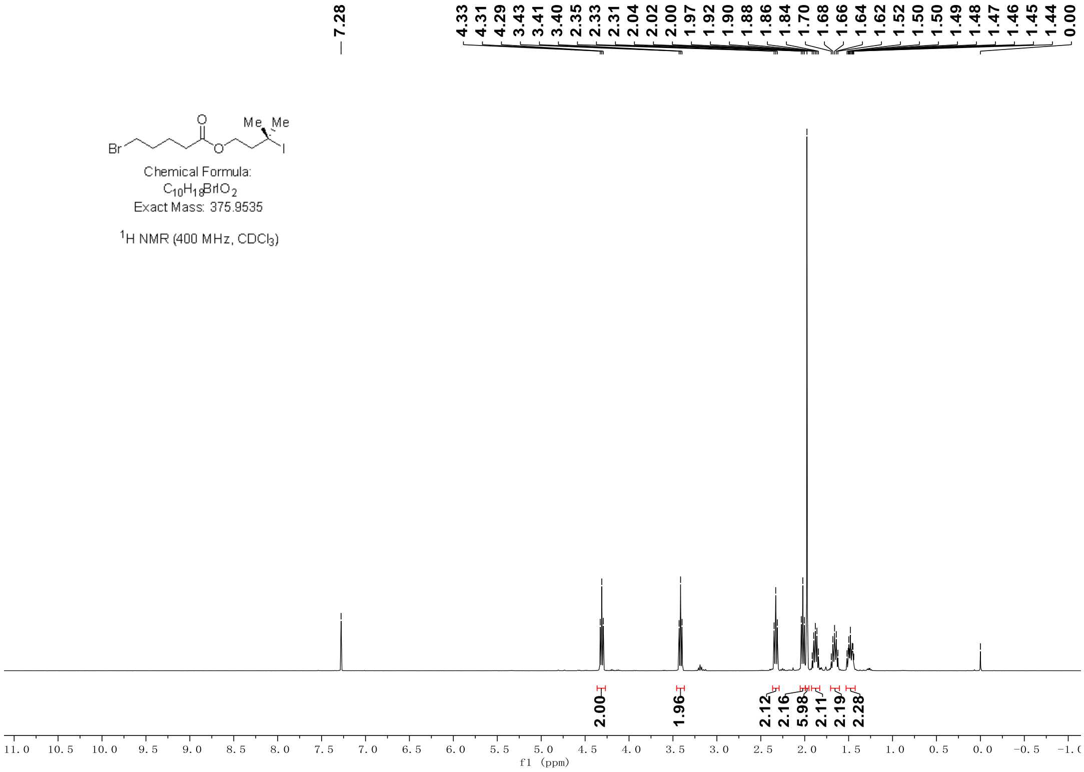

Supplementary Figure 8. ^{1}H NMR spectrum of compound 2e

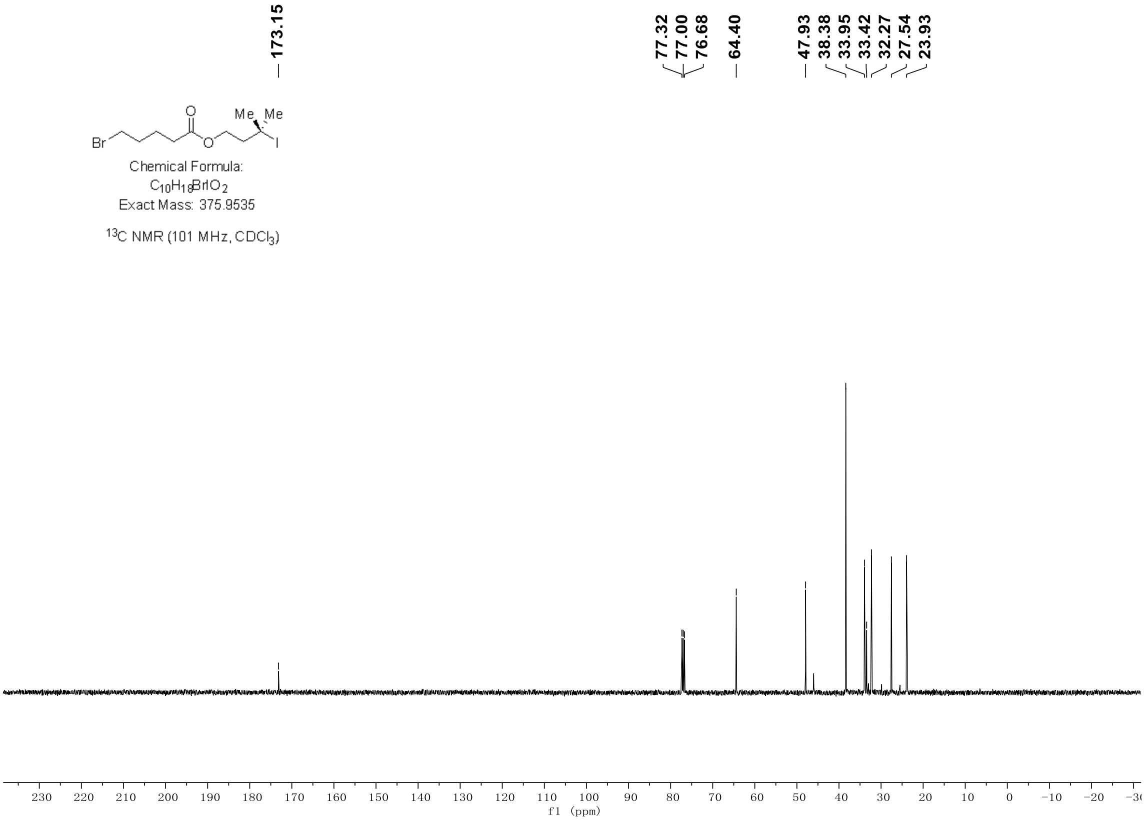

Supplementary Figure 9. ^{13}C NMR spectrum of compound 2e

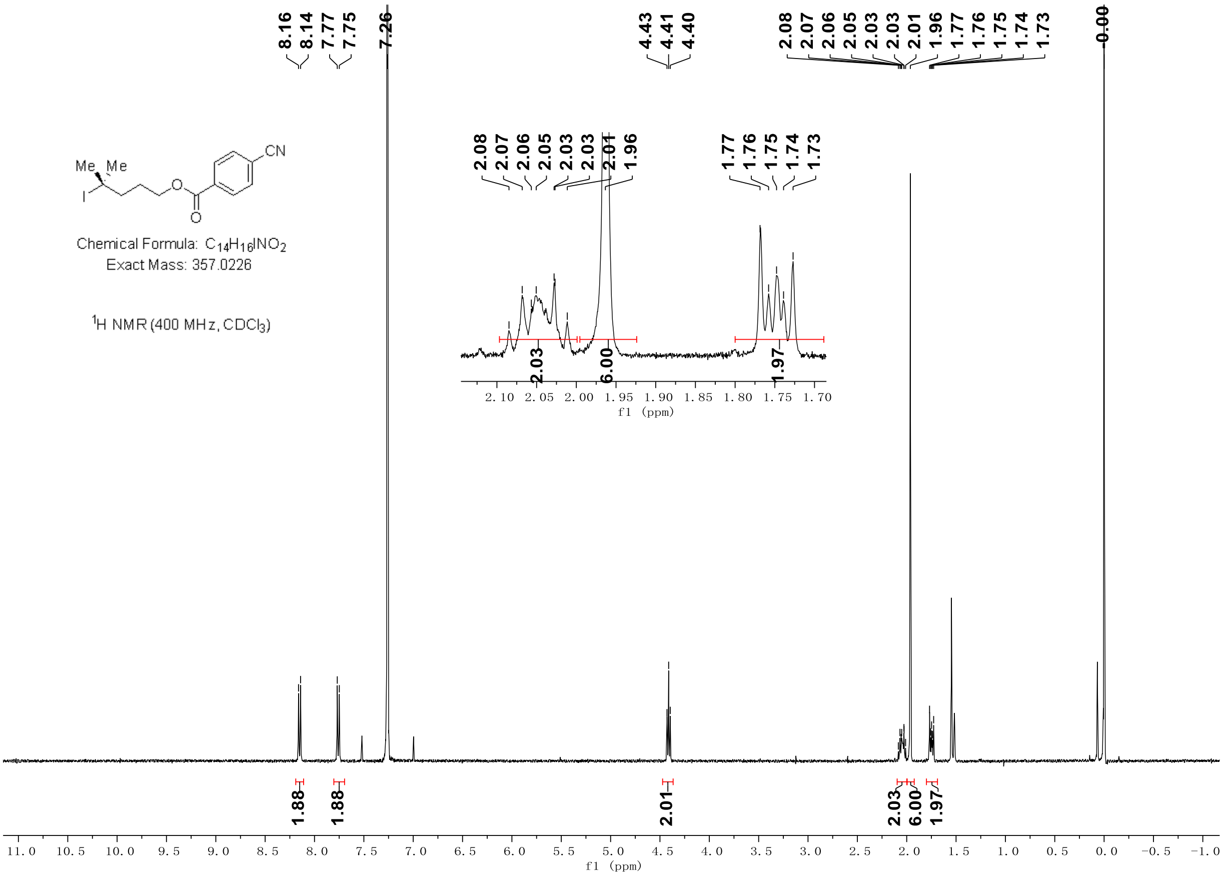

Supplementary Figure 10. ^{1}H NMR spectrum of compound 2f

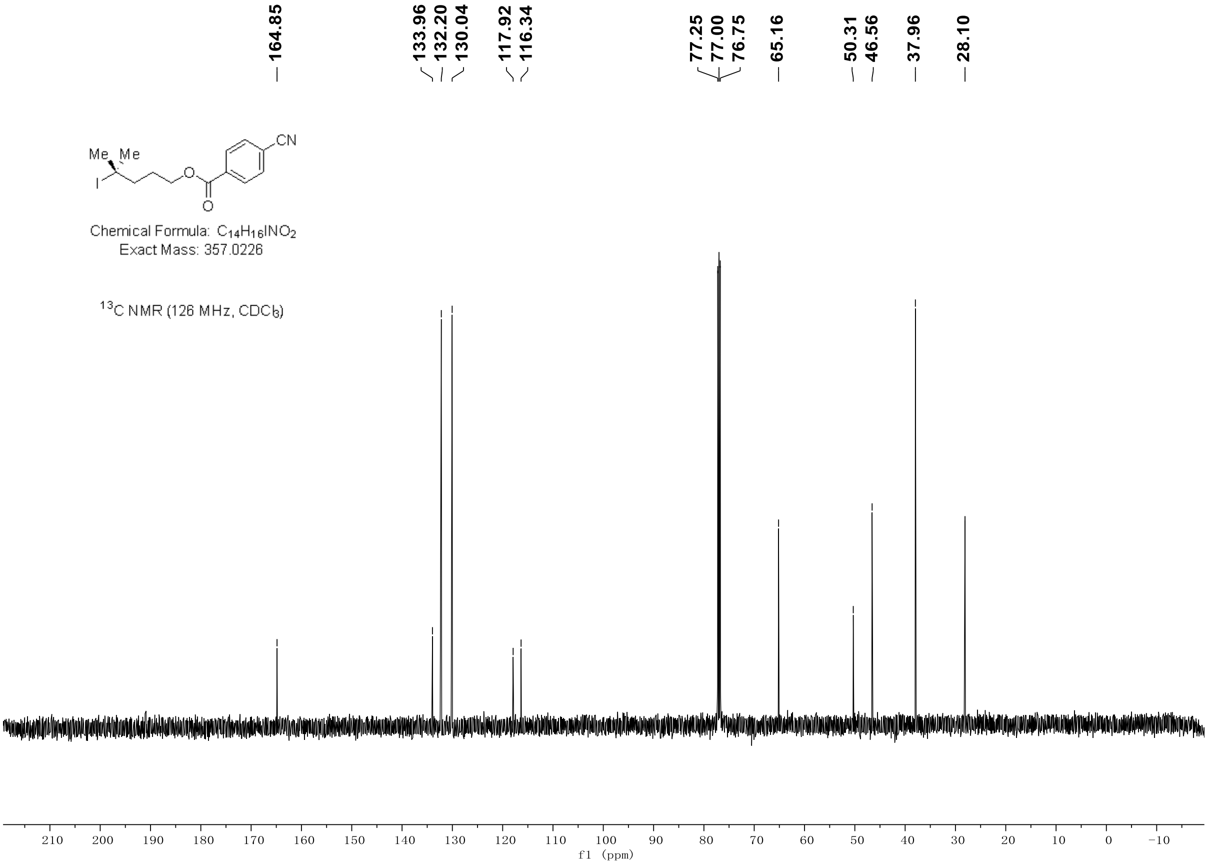

Supplementary Figure 11. ^{13}C NMR spectrum of compound 2f

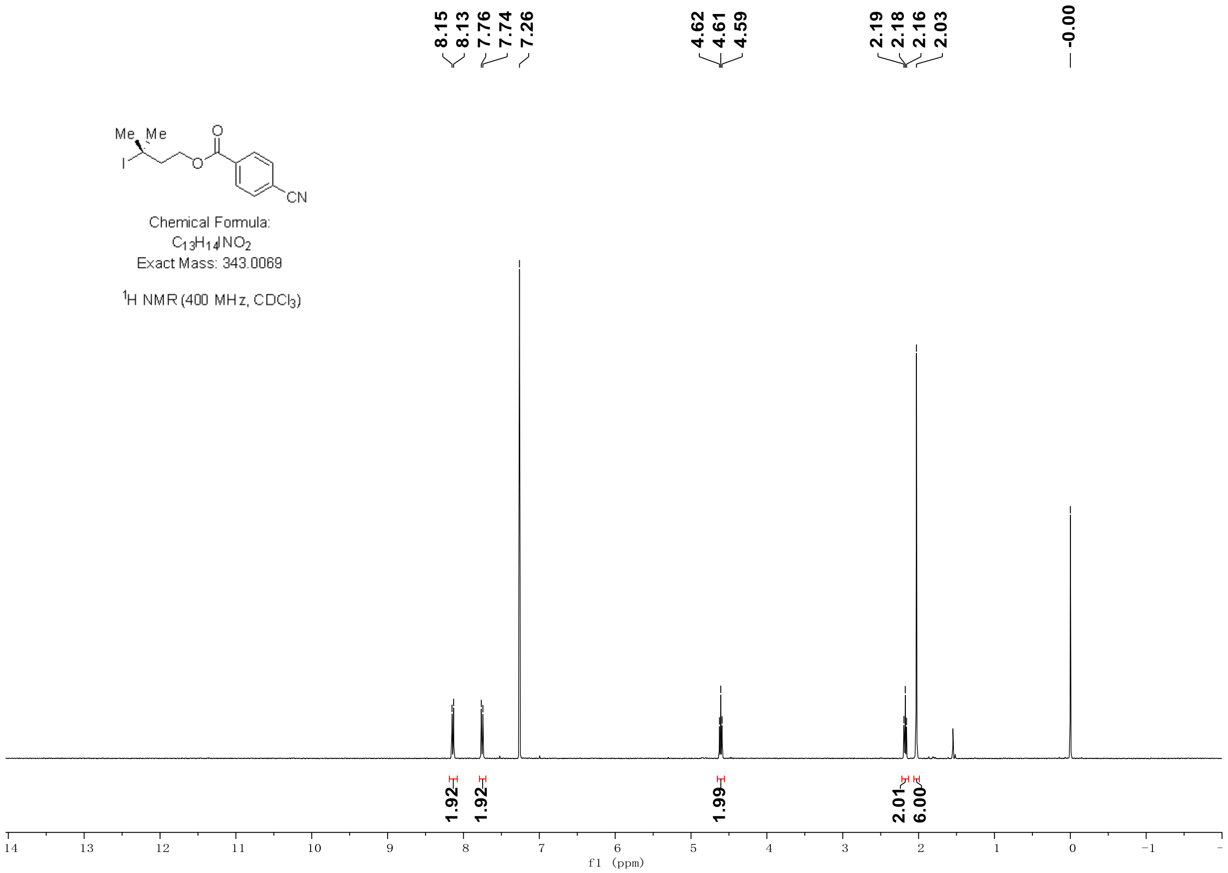

Supplementary Figure 12. ^{1}H NMR spectrum of compound 2g

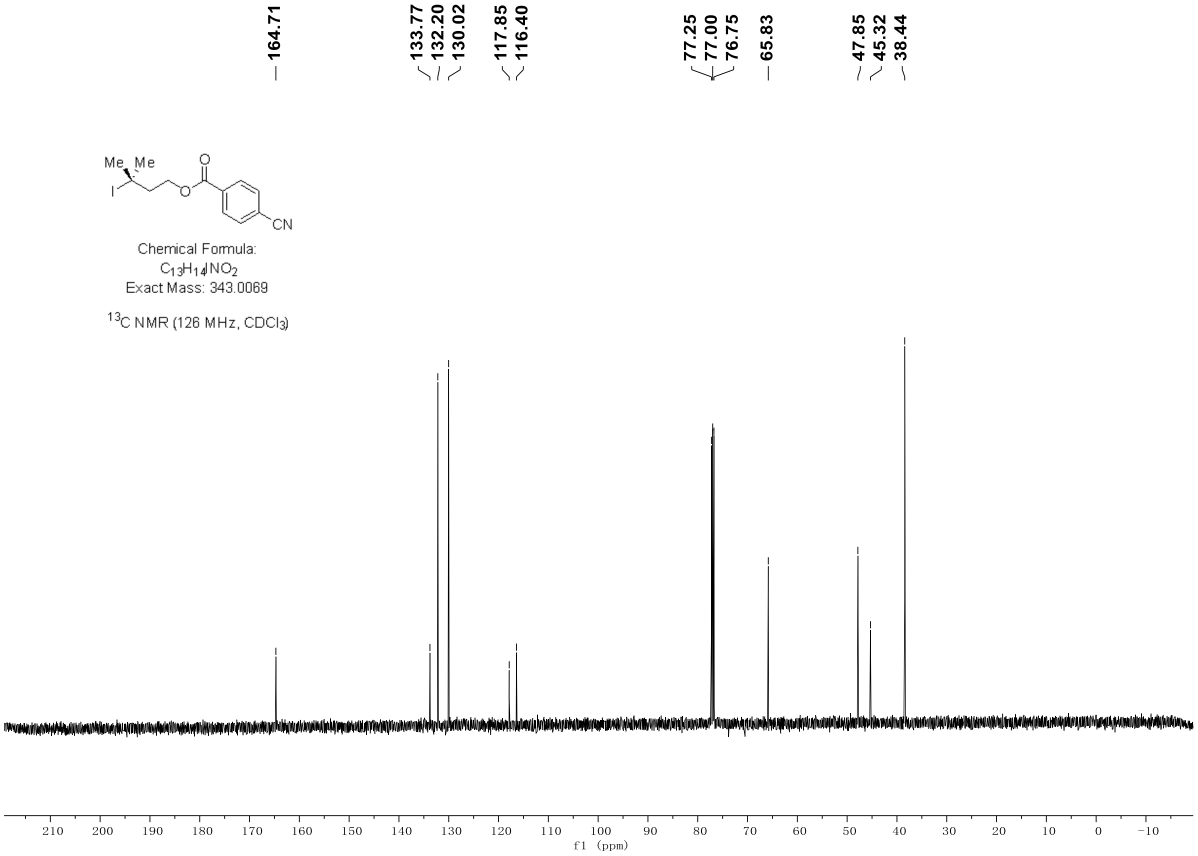

Supplementary Figure 13. ^{13}C NMR spectrum of compound 2g

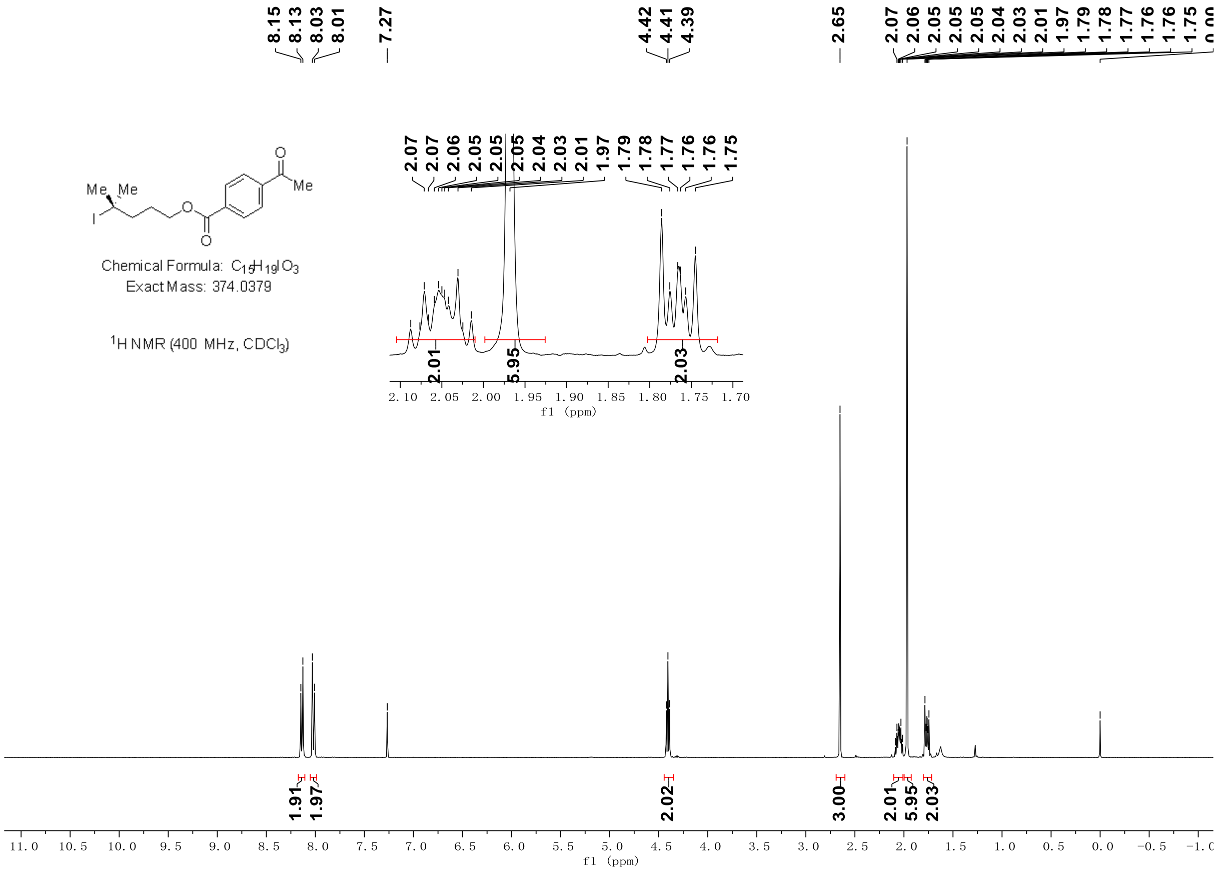

Supplementary Figure 14. ^{1}**H NMR spectrum of compound 2h**

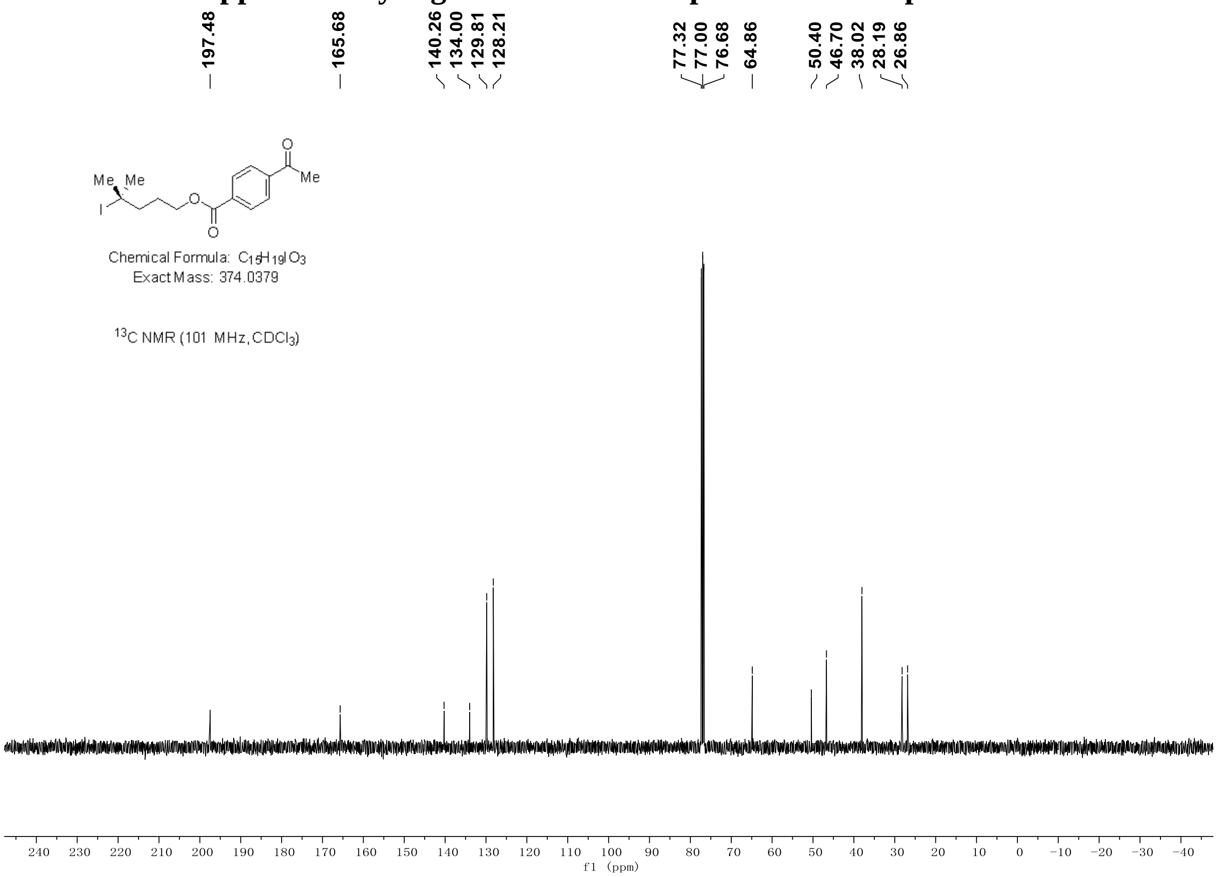

Supplementary Figure 15. ^{13}**C NMR spectrum of compound 2h**

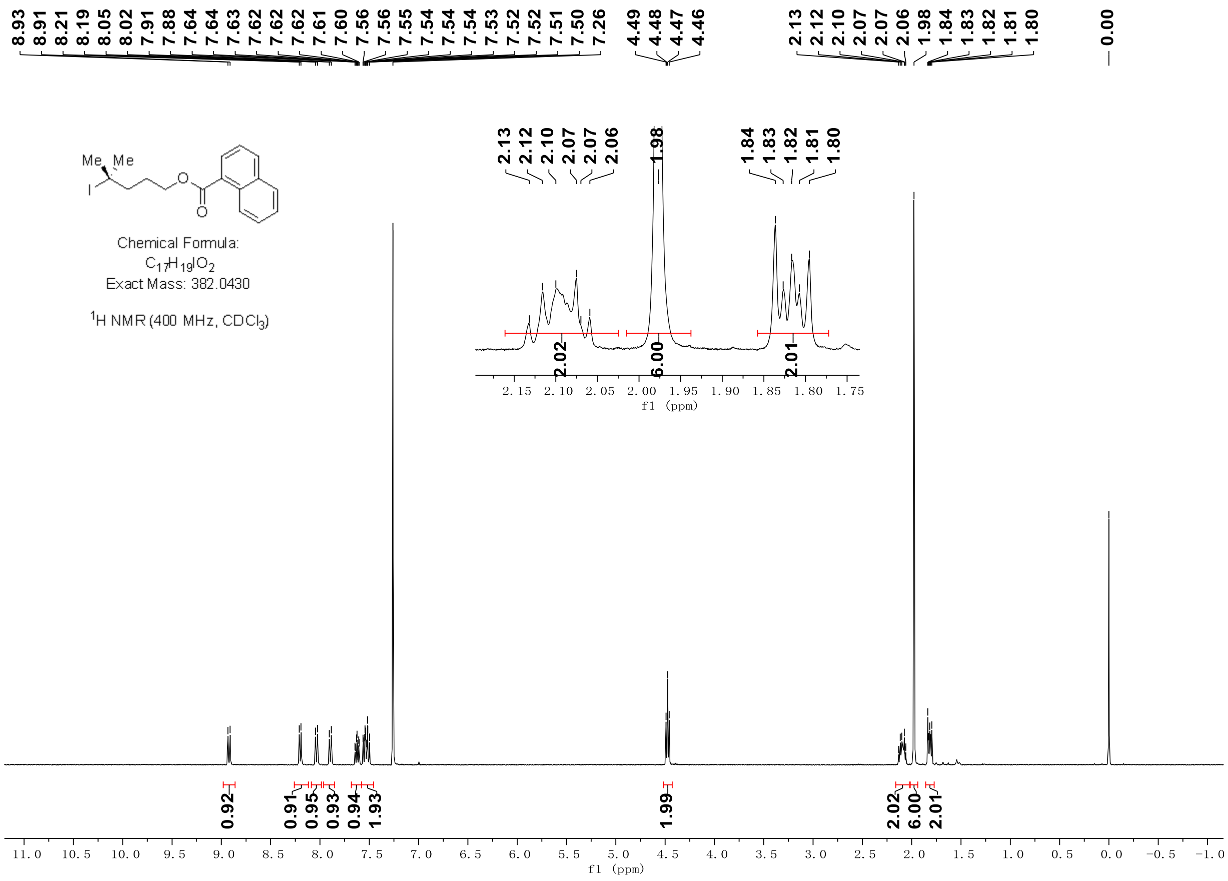

Supplementary Figure 16. ^{1}H NMR spectrum of compound 2i

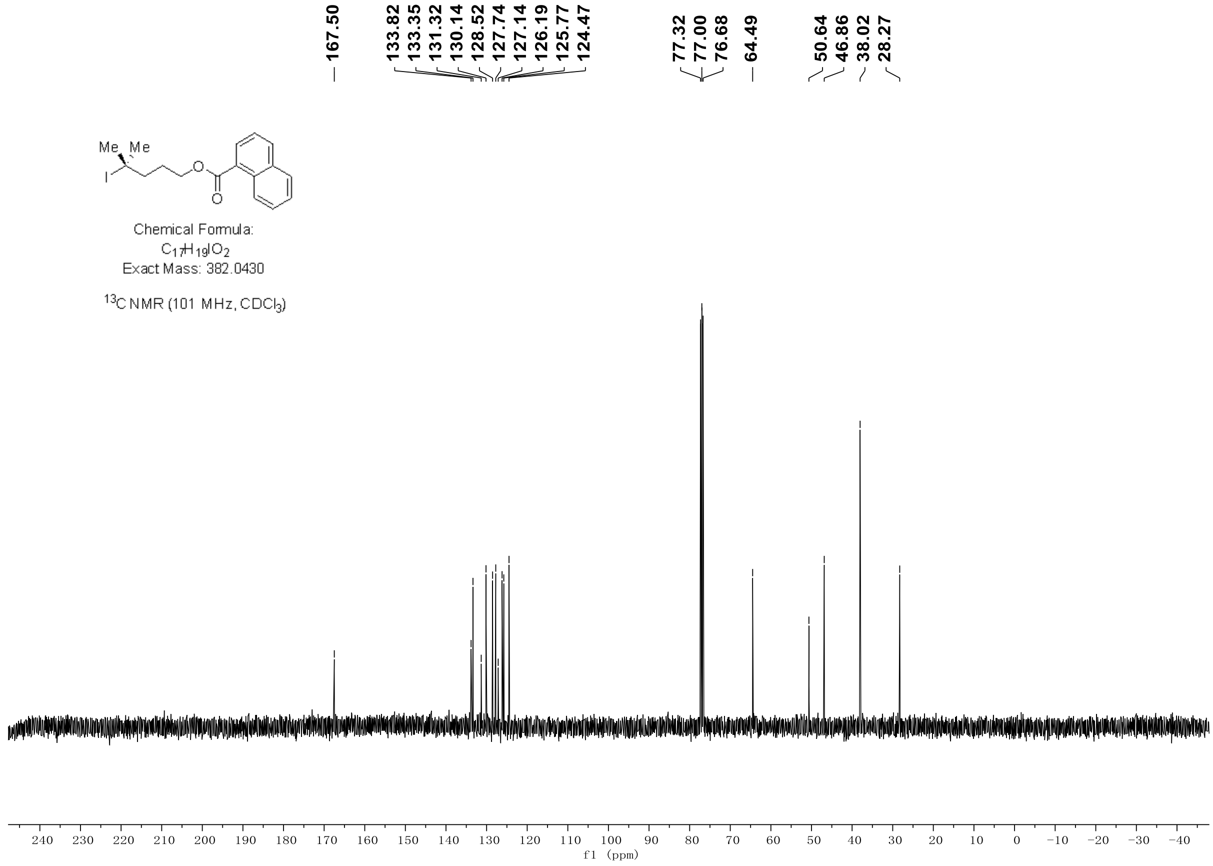

Supplementary Figure 17. ^{13}C NMR spectrum of compound 2i

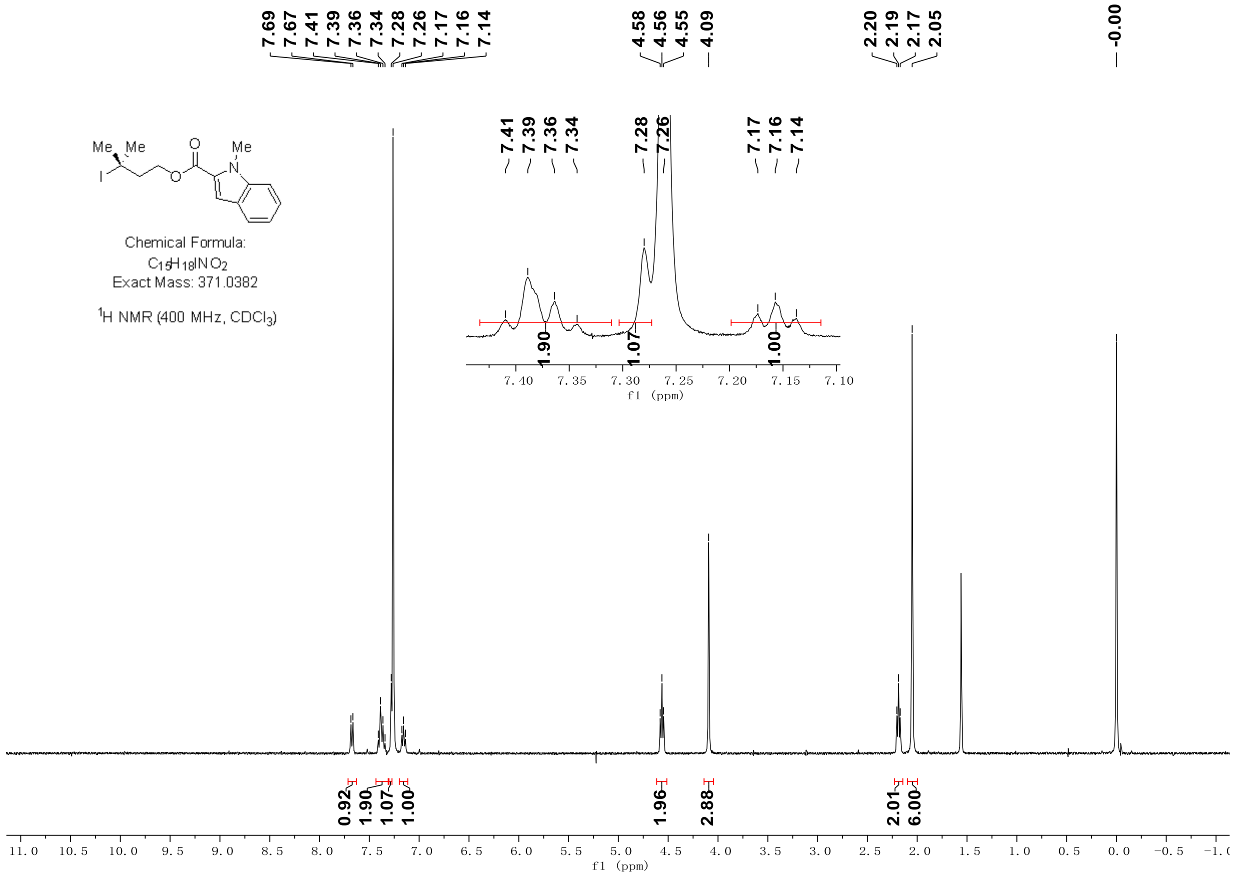

Supplementary Figure 18. ^{1}H NMR spectrum of compound 2j

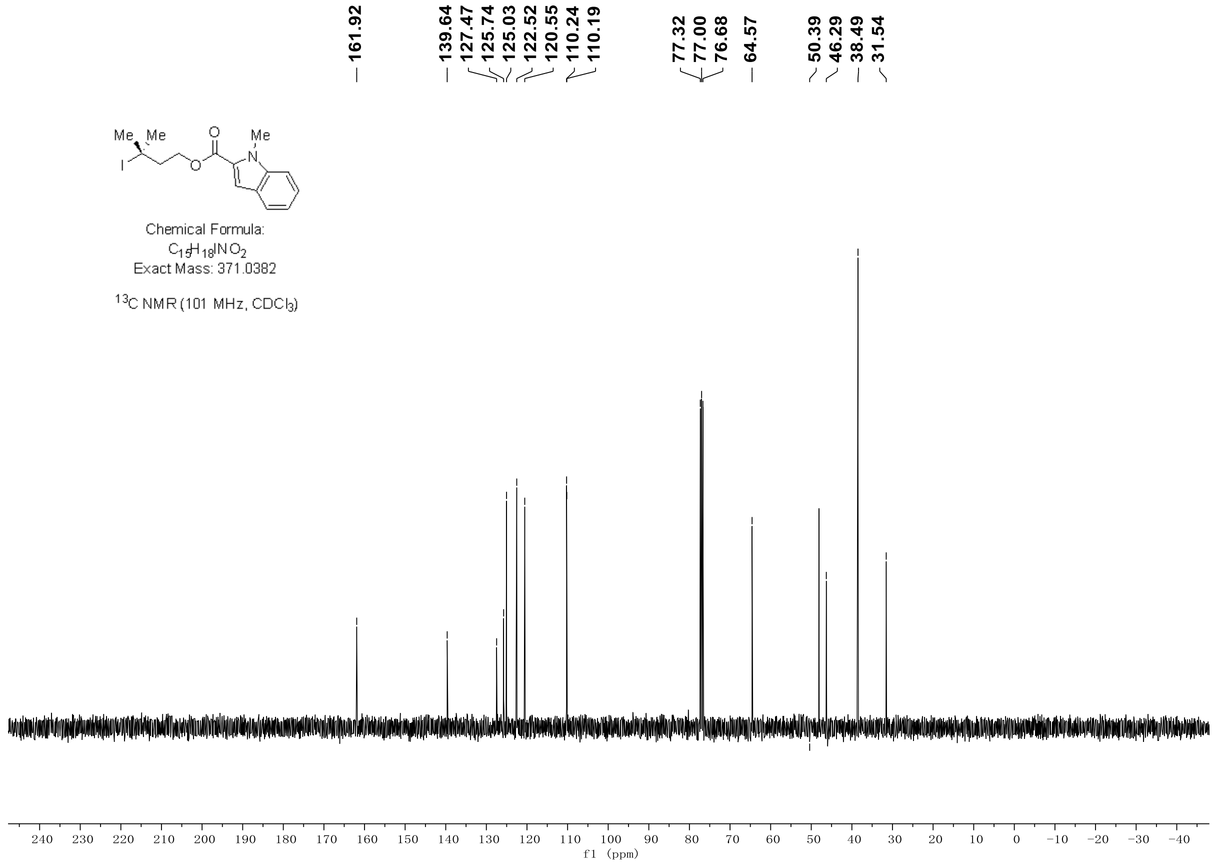

Supplementary Figure 19. ^{13}C NMR spectrum of compound 2j

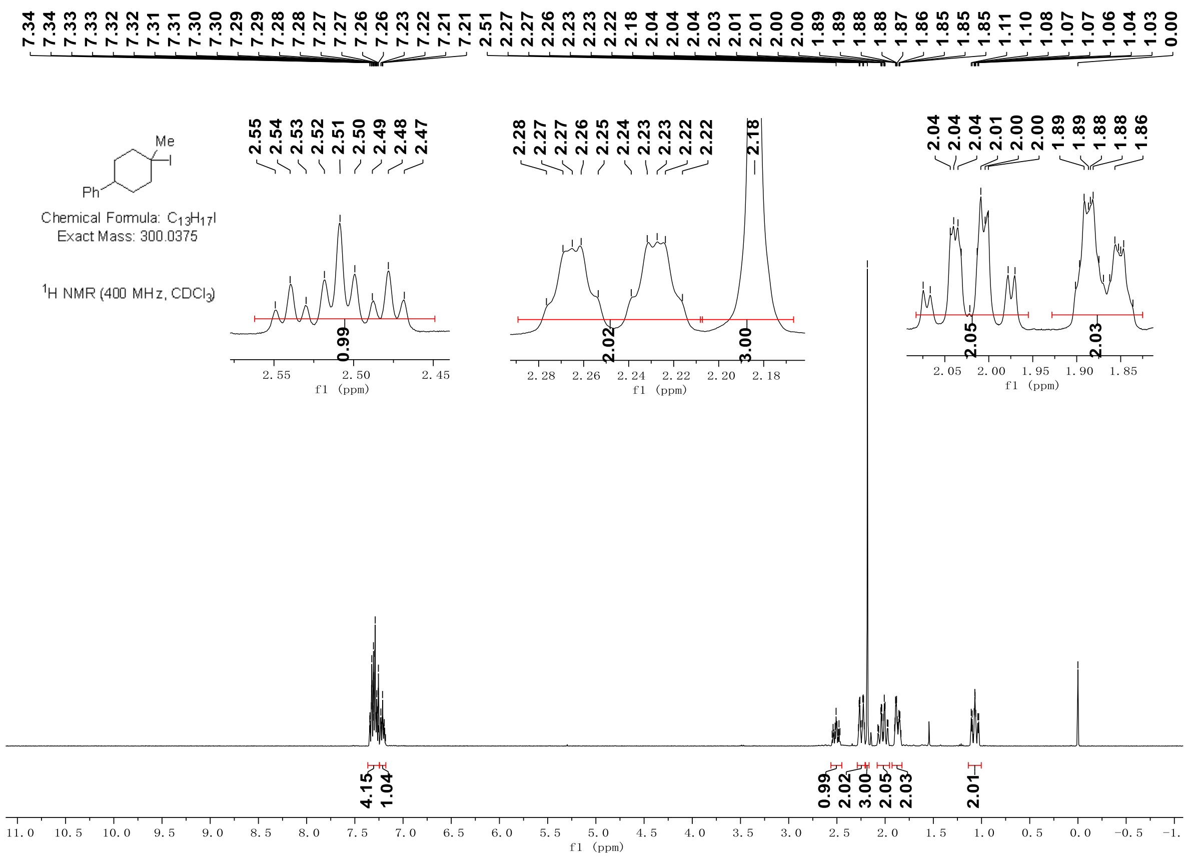

Supplementary Figure 20. ^{1}H NMR spectrum of compound 2k

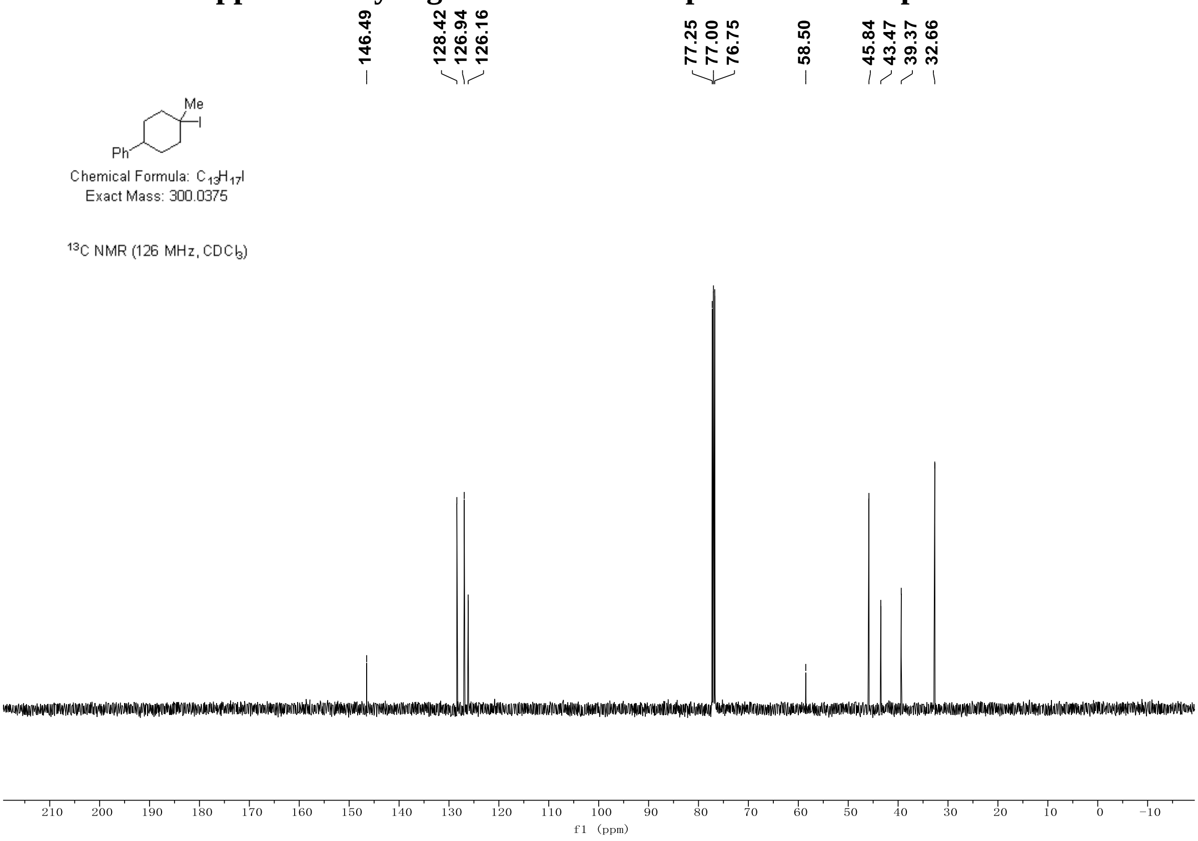

Supplementary Figure 21. ^{13}C NMR spectrum of compound 2k

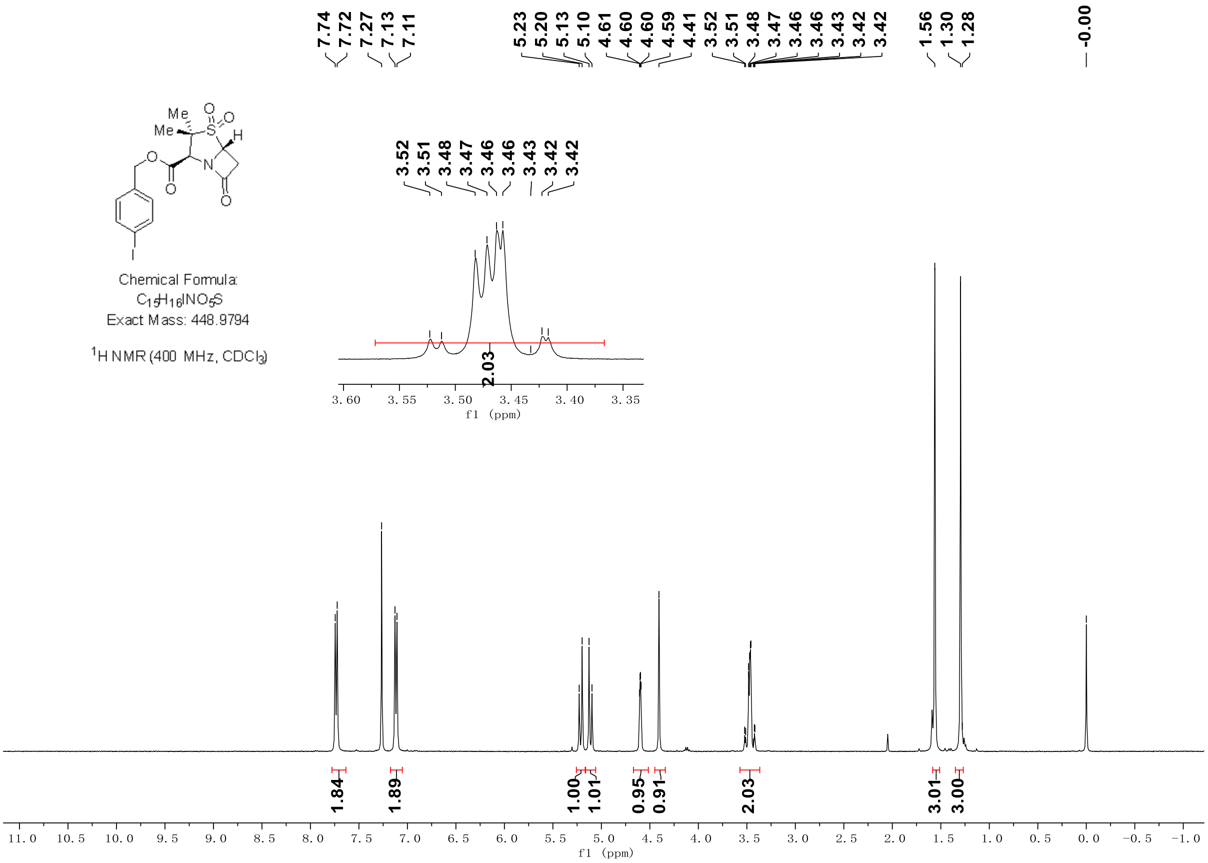

Supplementary Figure 22. ^{1}H NMR spectrum of compound 3q

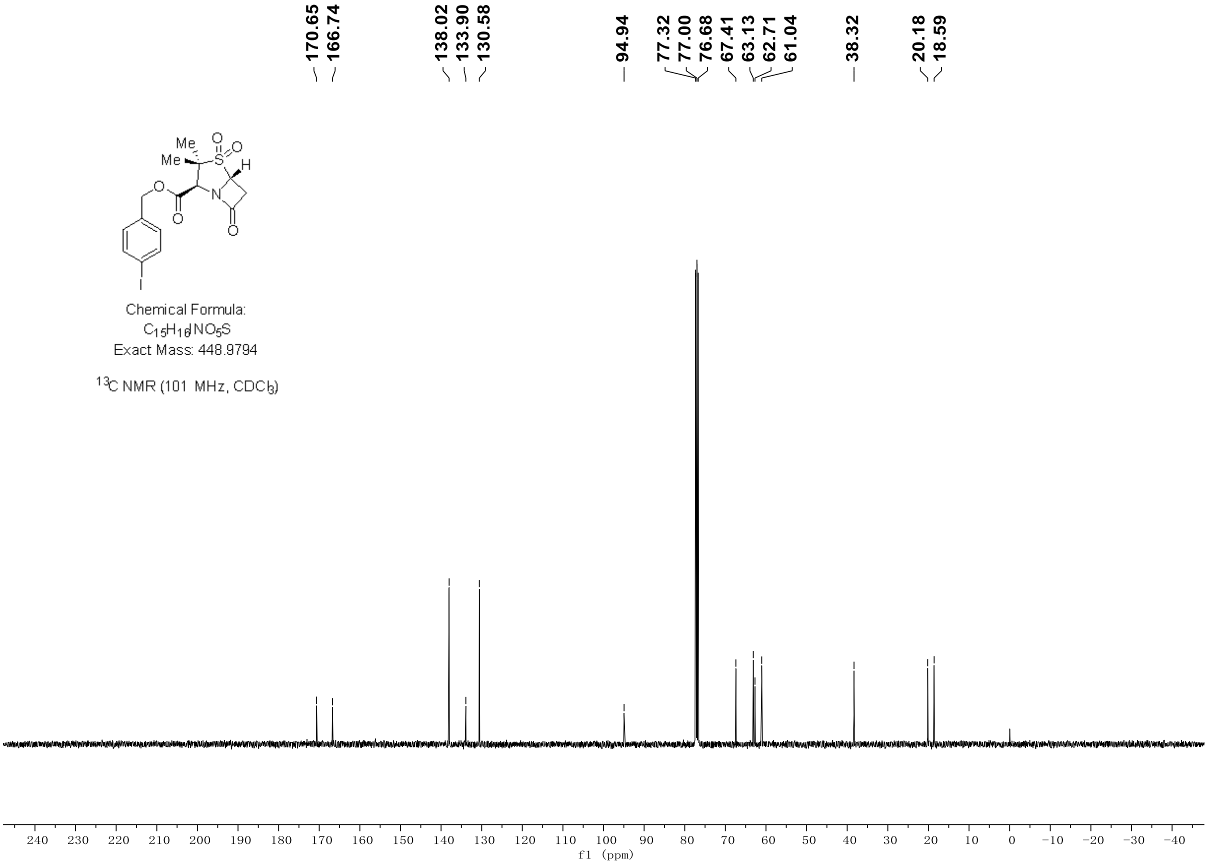

Supplementary Figure 23. ^{13}C NMR spectrum of compound 3q

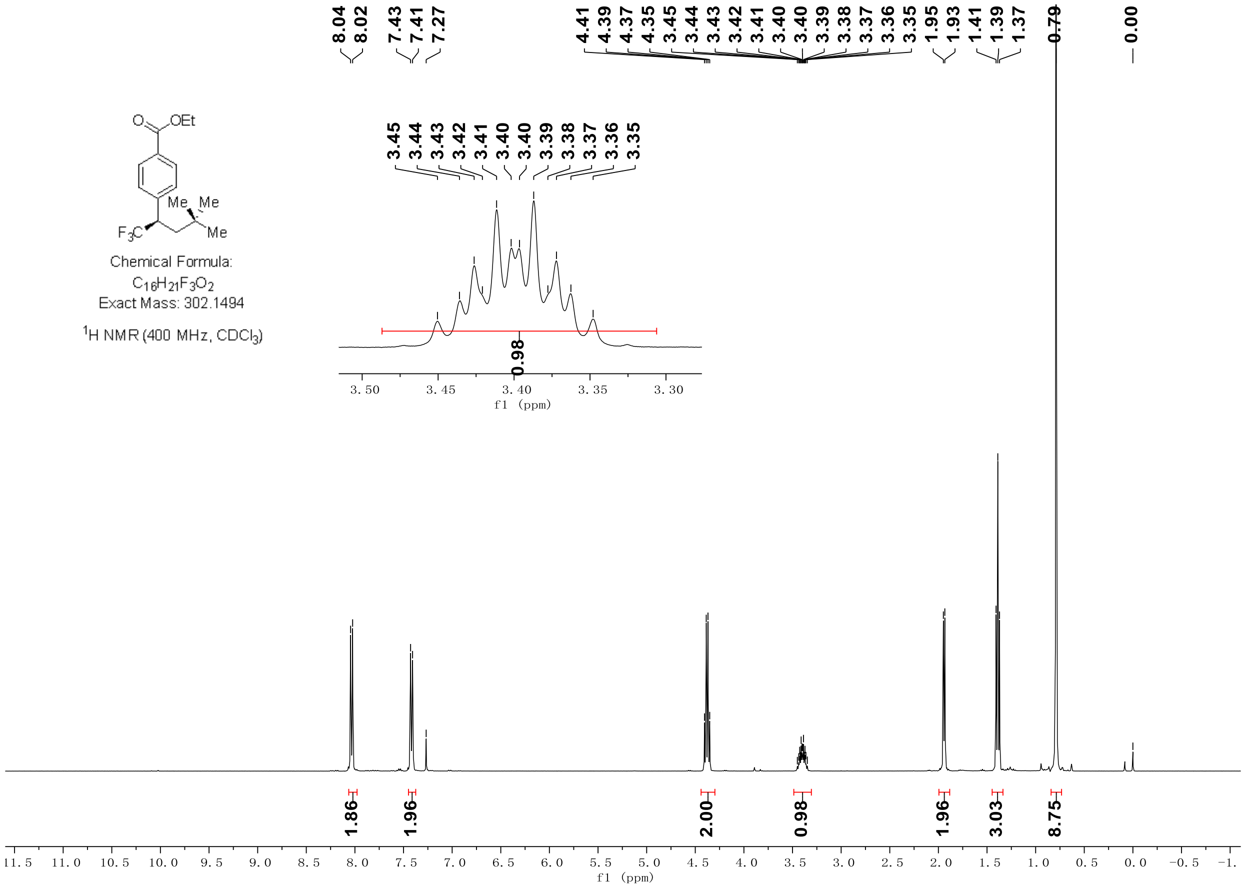

Supplementary Figure 24. ^{1}H NMR spectrum of compound 4a

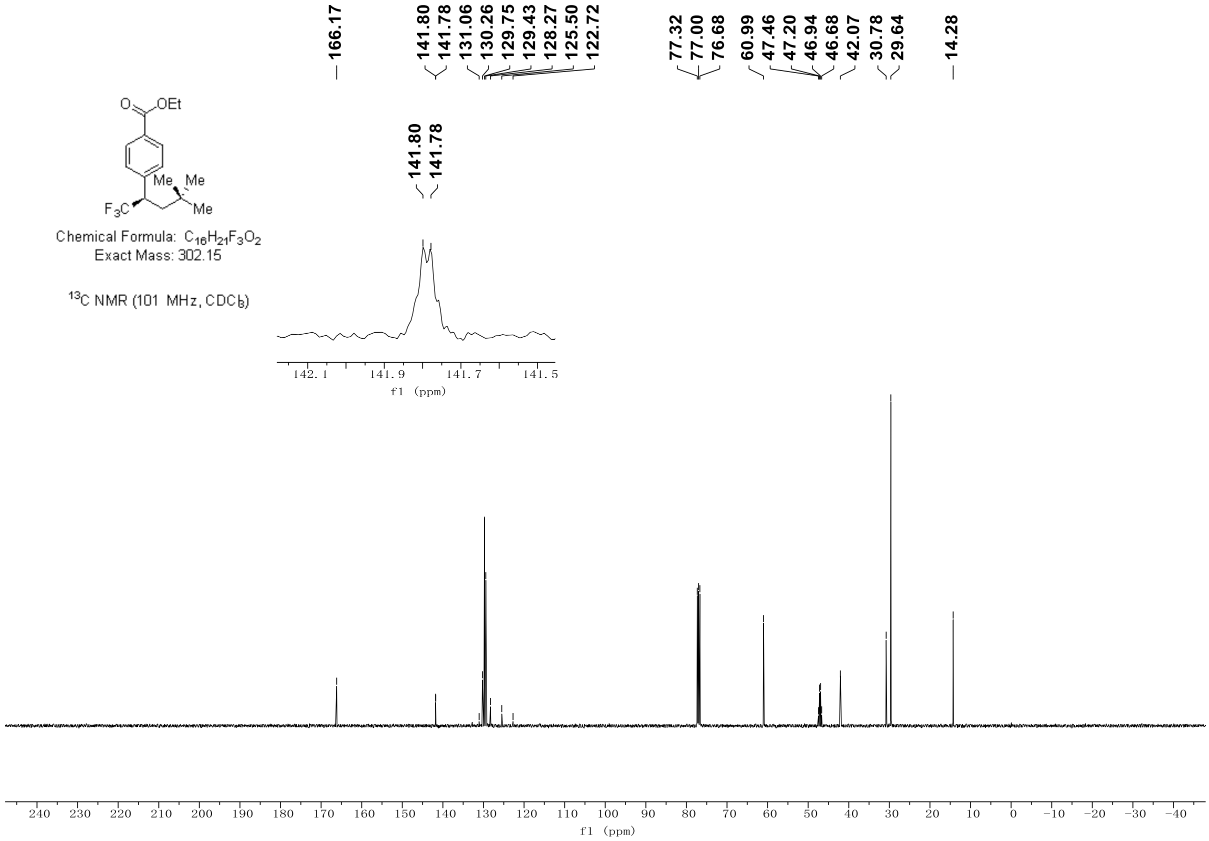

Supplementary Figure 25. ^{13}C NMR spectrum of compound 4a

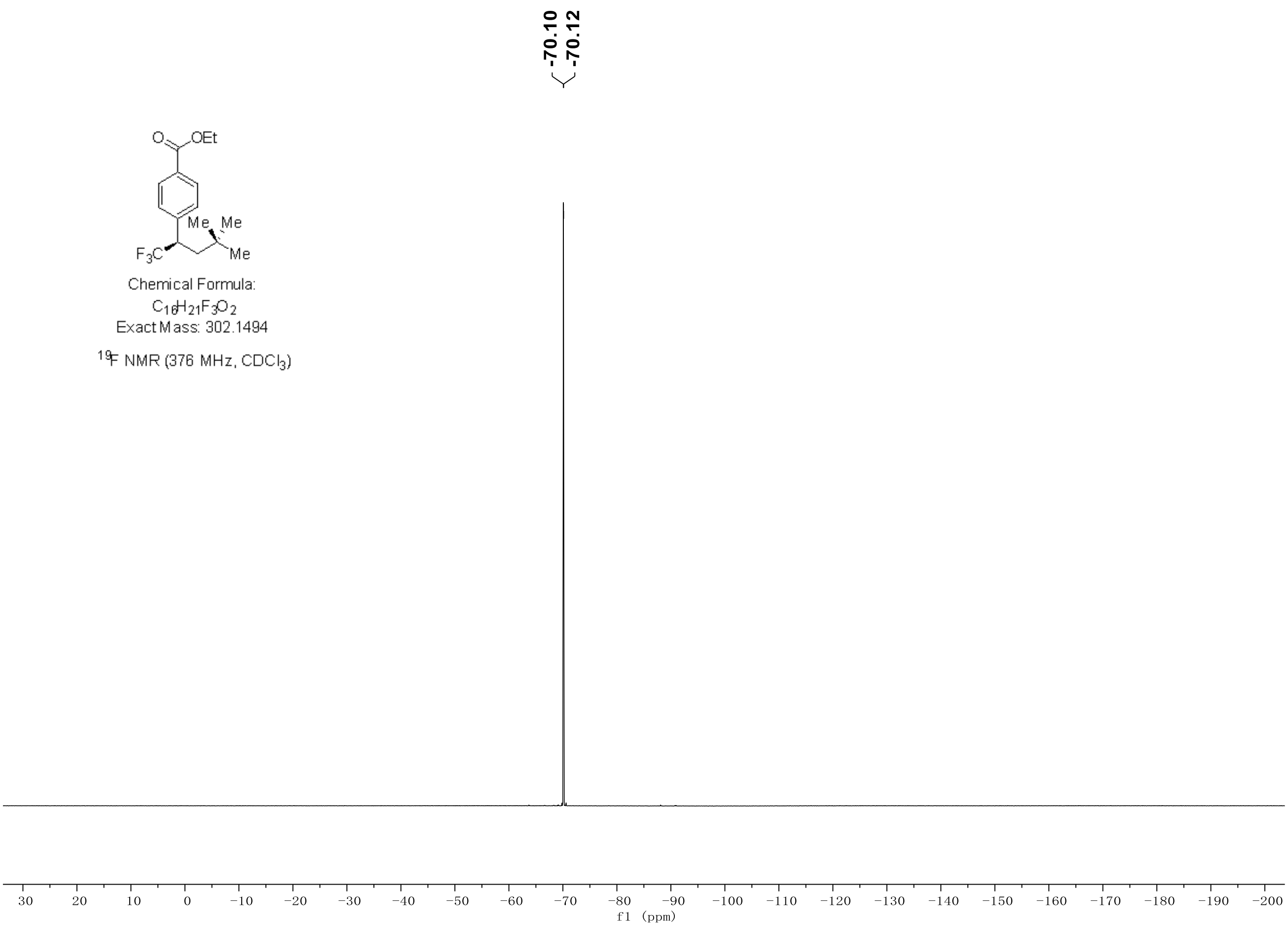

Supplementary Figure 26. ^{19}F NMR spectrum of compound 4a

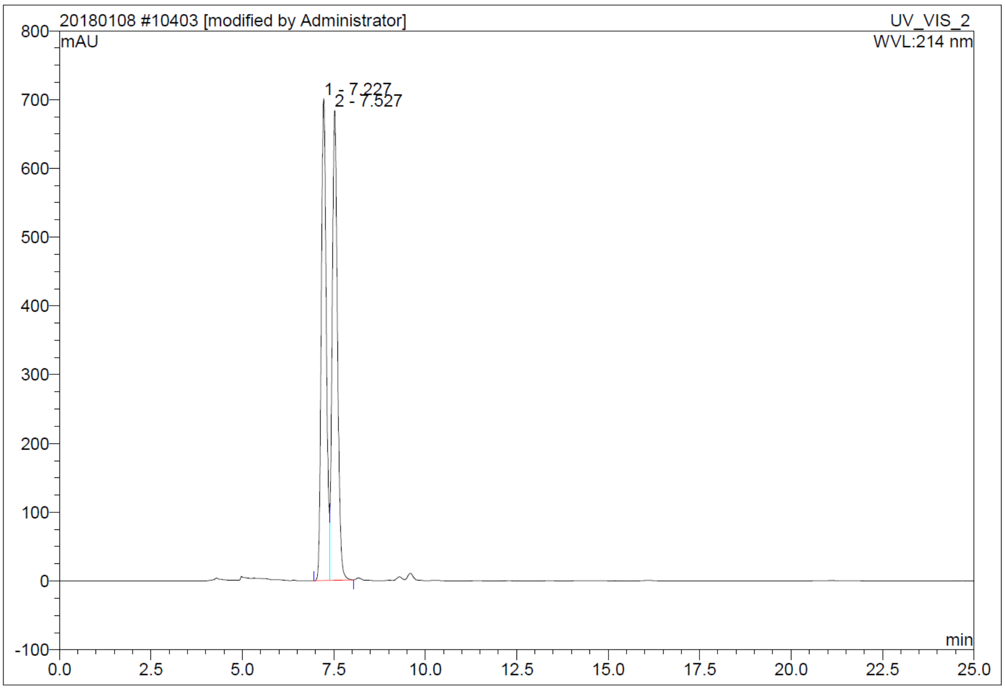

No.	Ret.Time min	Peak Name	Height mAU	Area mAU*min	Rel.Area %	Amount	Type
1	7.23	n.a.	700.978	110.713	48.91	n.a.	BM *
2	7.53	n.a.	683.645	115.664	51.09	n.a.	MB*
Total:			1384.623	226.377	100.00	0.000	

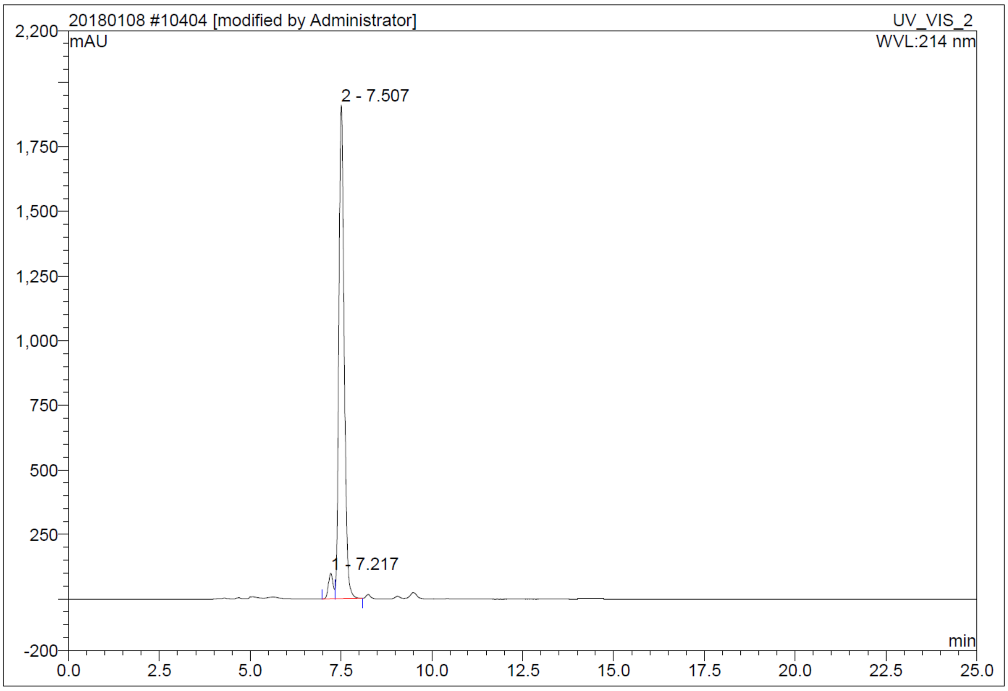

No.	Ret.Time min	Peak Name	Height mAU	Area mAU*min	Rel.Area %	Amount	Type
1	7.22	n.a.	98.639	14.603	4.31	n.a.	BM
2	7.51	n.a.	1911.048	323.897	95.69	n.a.	MB
Total:			2009.687	338.500	100.00	0.000	

Supplementary Figure 27. Chiral HPLC analysis of compound 4a

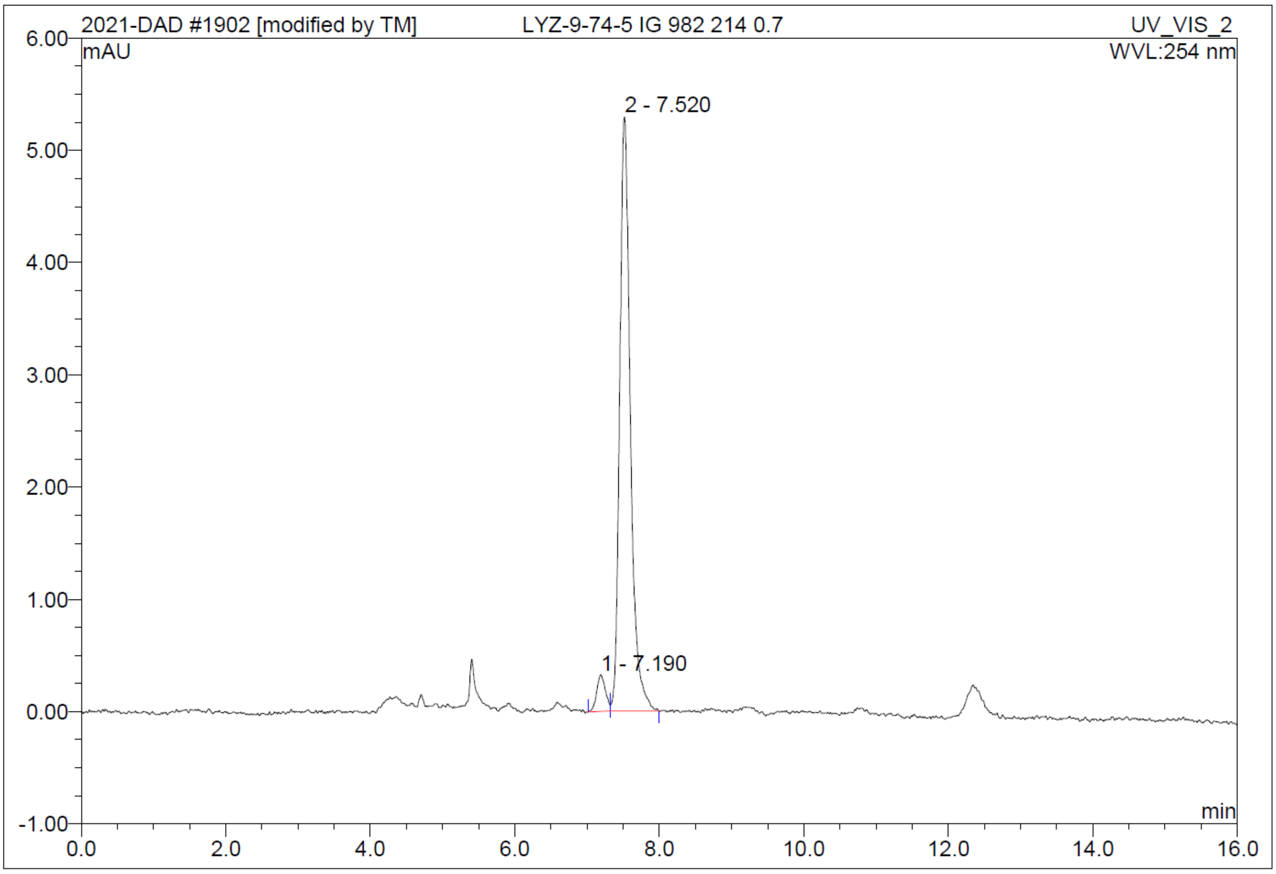

No.	Ret.Time min	Peak Name	Height mAU	Area mAU*min	Rel.Area %	Amount	Type
1	7.19	n.a.	0.326	0.046	4.81	n.a.	BM *
2	7.52	n.a.	5.293	0.903	95.19	n.a.	MB*
Total:			5.618	0.948	100.00	0.000	

Supplementary Figure 28. Chiral HPLC analysis of compound 4a using ethyl 4-bromobenzoate instead of ethyl 4-iodobenzoate.

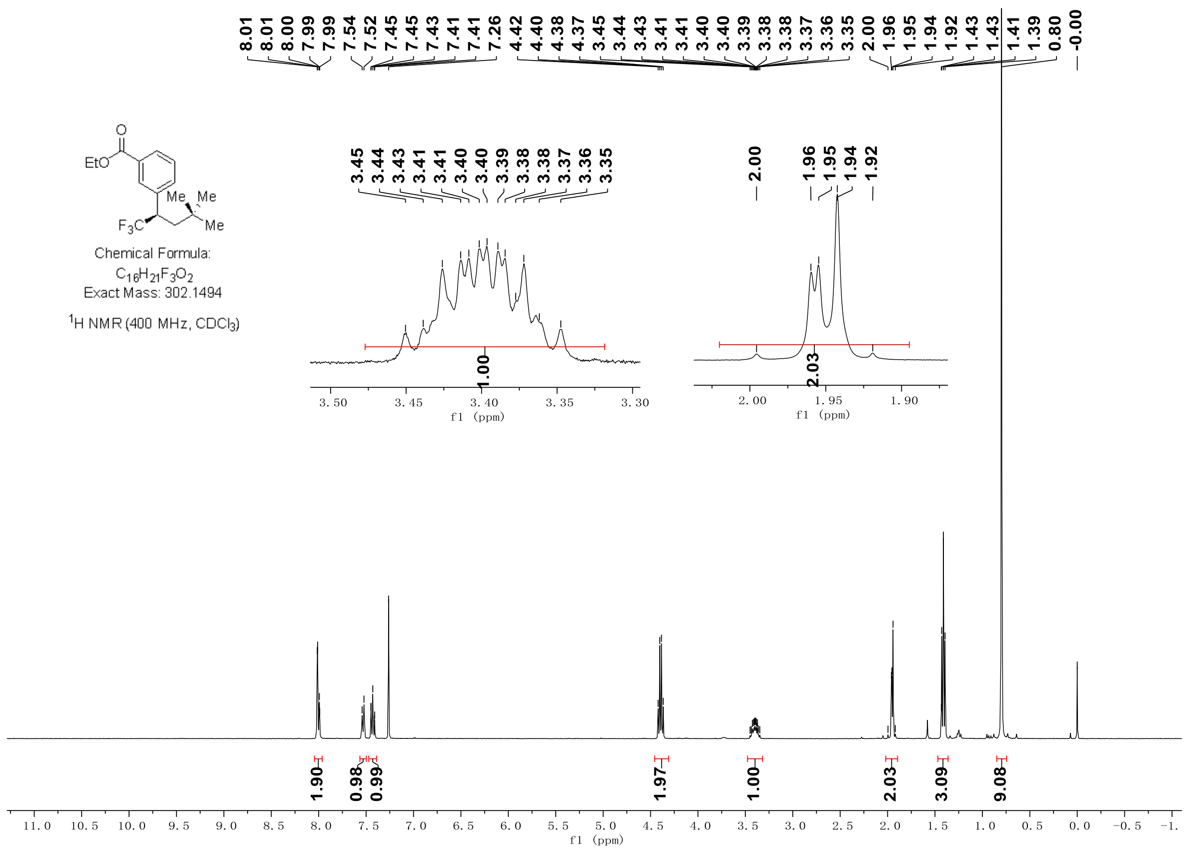

Supplementary Figure 29. ^{1}H NMR spectrum of compound 4b

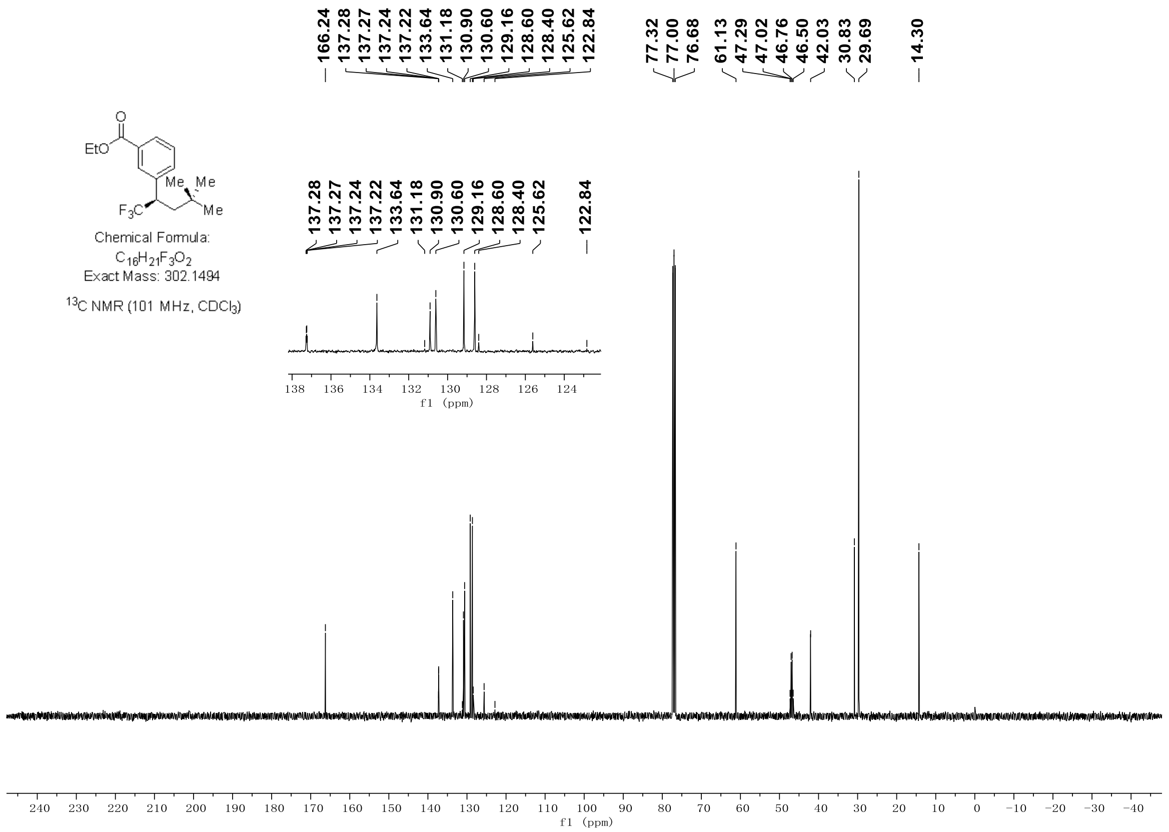

Supplementary Figure 30. ^{13}C NMR spectrum of compound 4b

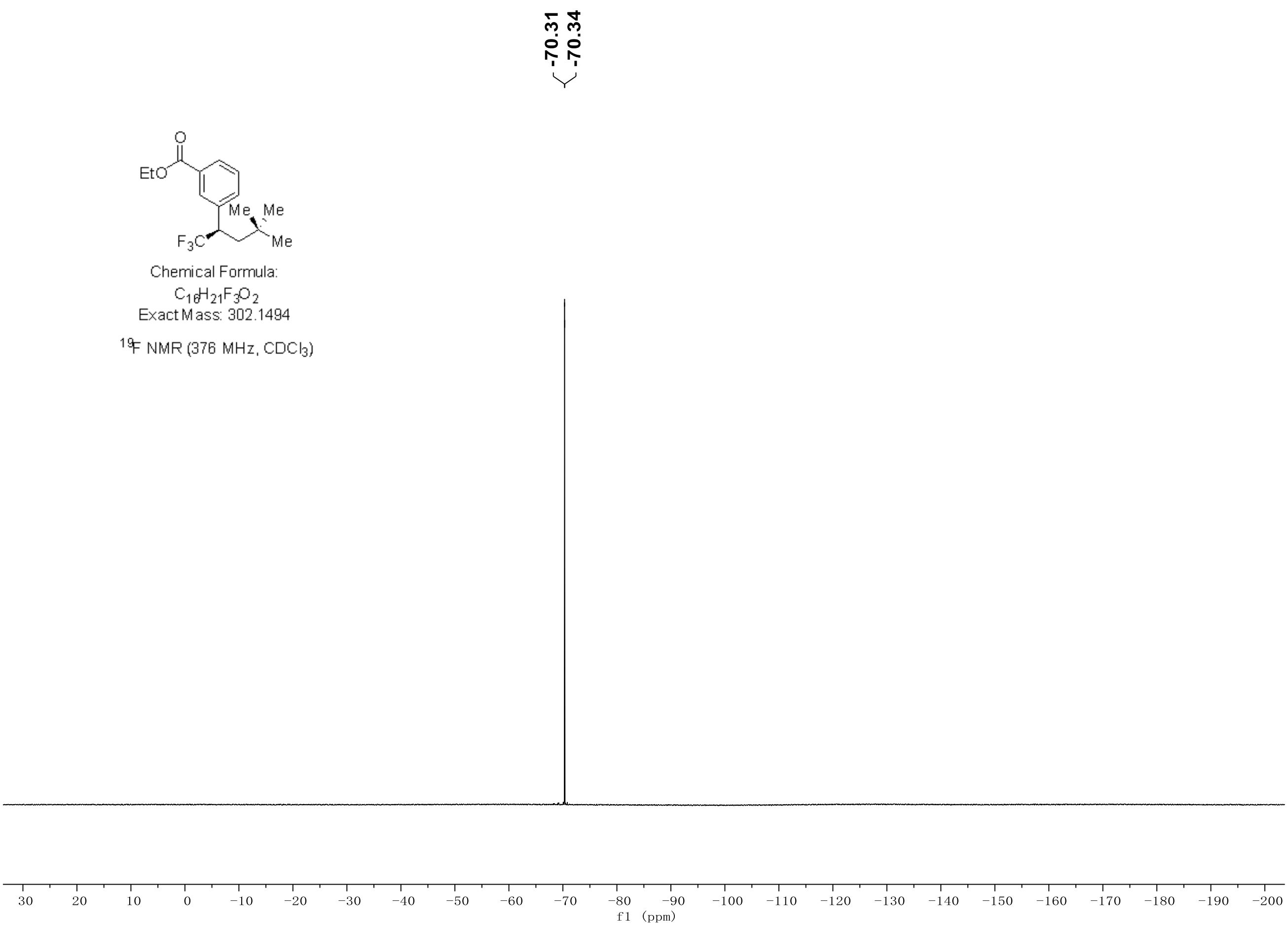

Supplementary Figure 31. ^{19}F NMR spectrum of compound 4b

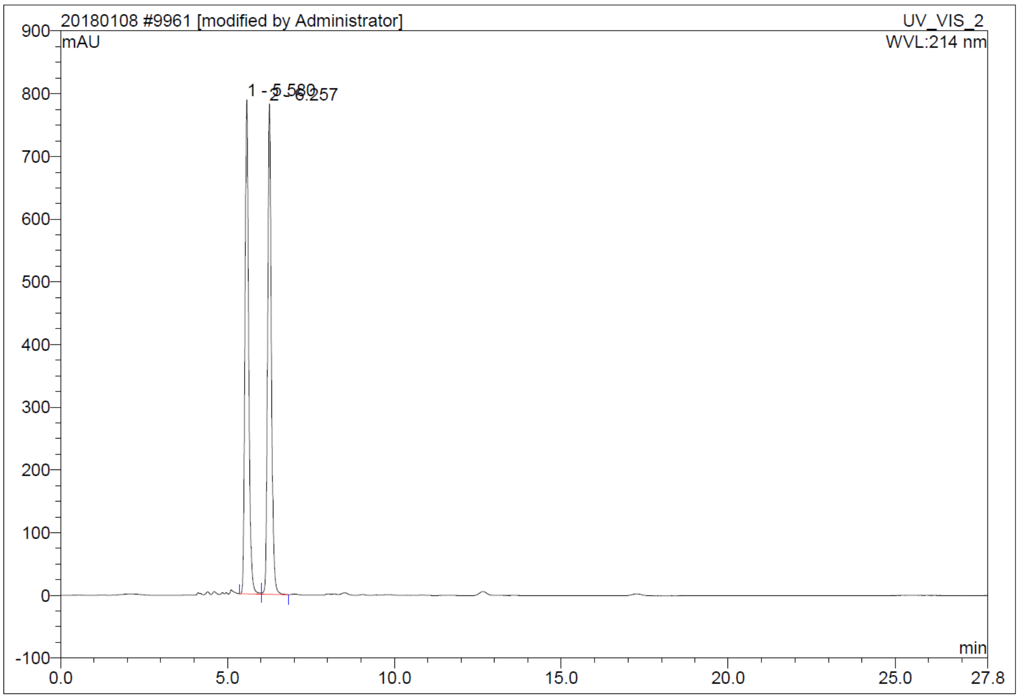

No.	Ret.Time min	Peak Name	Height mAU	Area mAU*min	Rel.Area %	Amount	Type
1	5.58	n.a.	788.392	101.530	49.88	n.a.	BM *
2	6.26	n.a.	782.721	102.012	50.12	n.a.	MB*
Total:			1571.113	203.542	100.00	0.000	

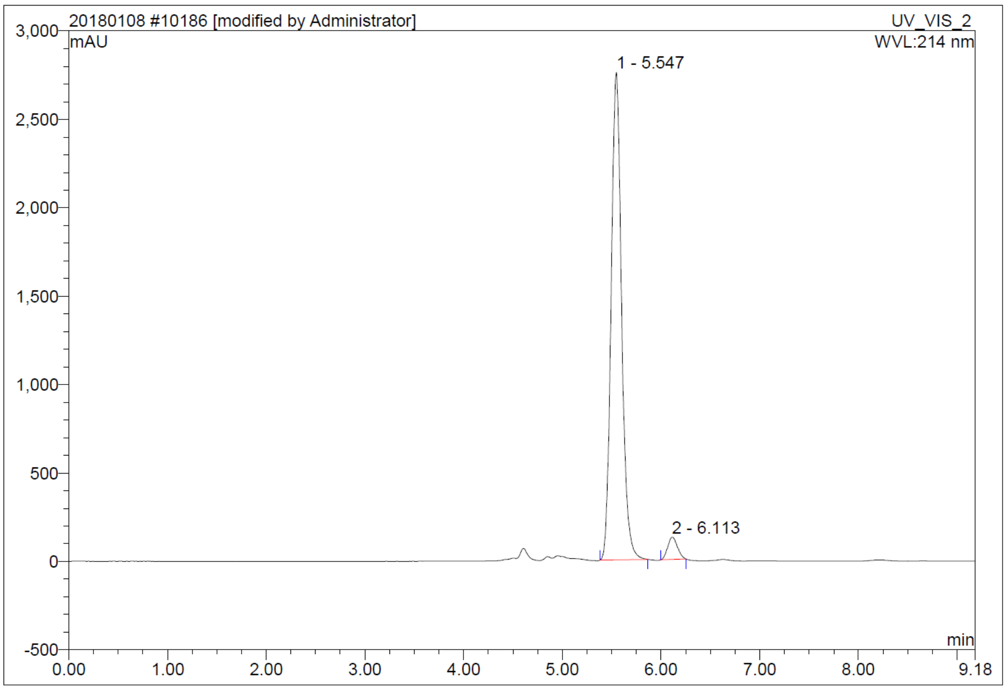

No.	Ret.Time min	Peak Name	Height mAU	Area mAU*min	Rel.Area %	Amount	Type
1	5.55	n.a.	2759.270	338.038	95.92	n.a.	BMB*
2	6.11	n.a.	125.435	14.365	4.08	n.a.	BMB*
Total:			2884.706	352.402	100.00	0.000	

Supplementary Figure 32. Chiral HPLC analysis of compound 4b

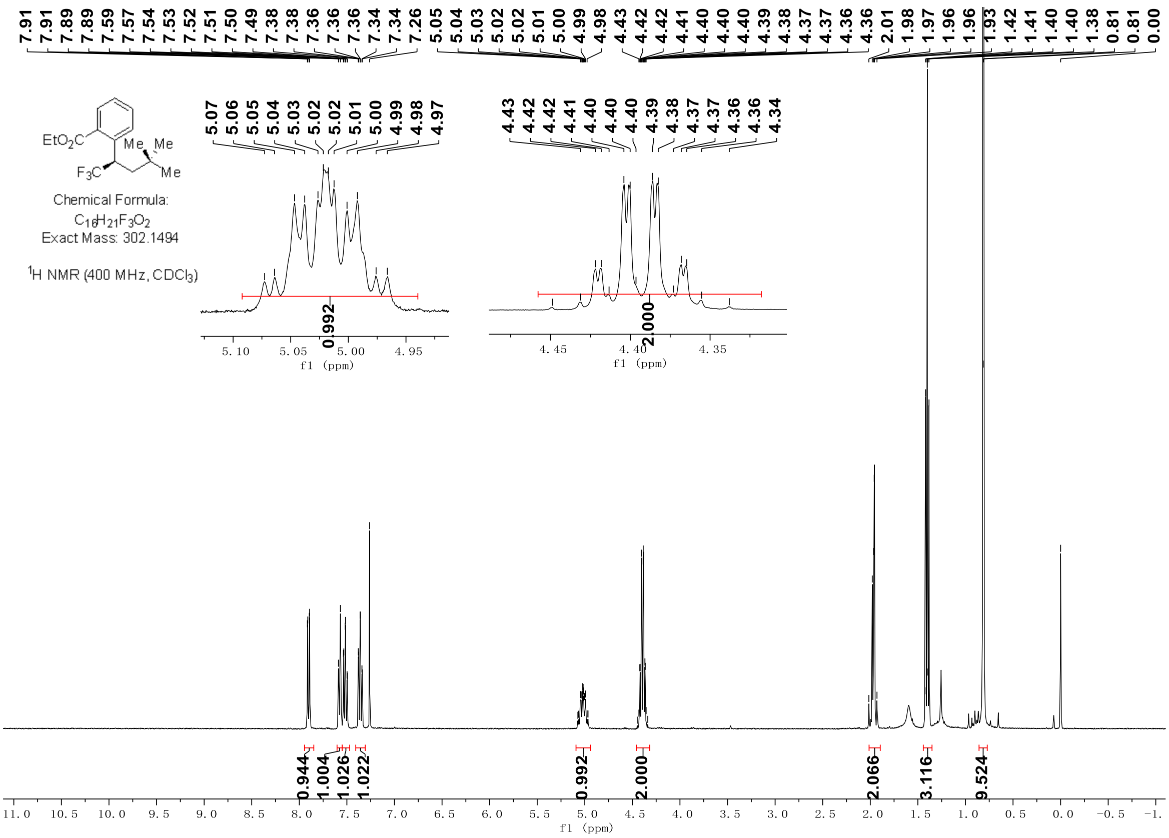

Supplementary Figure 33. ^{1}H NMR spectrum of compound 4c

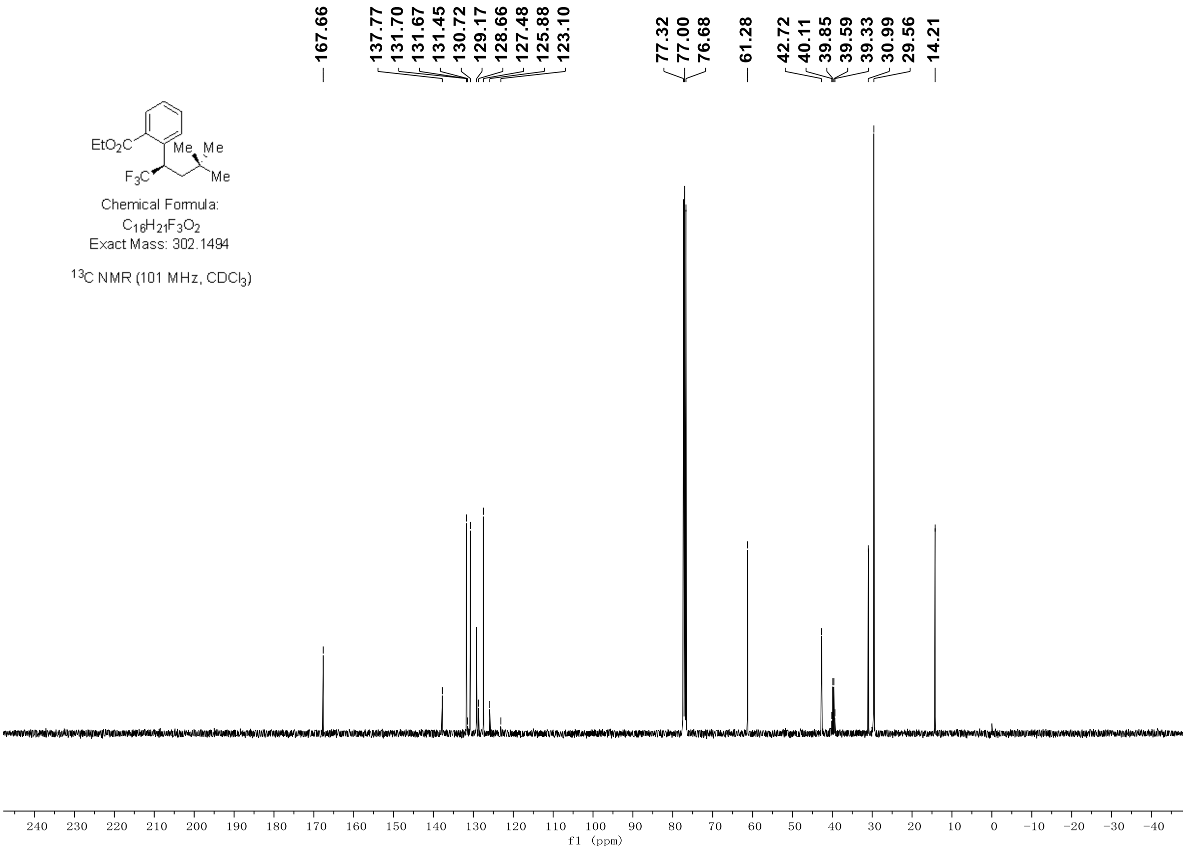

Supplementary Figure 34. ^{13}C NMR spectrum of compound 4c

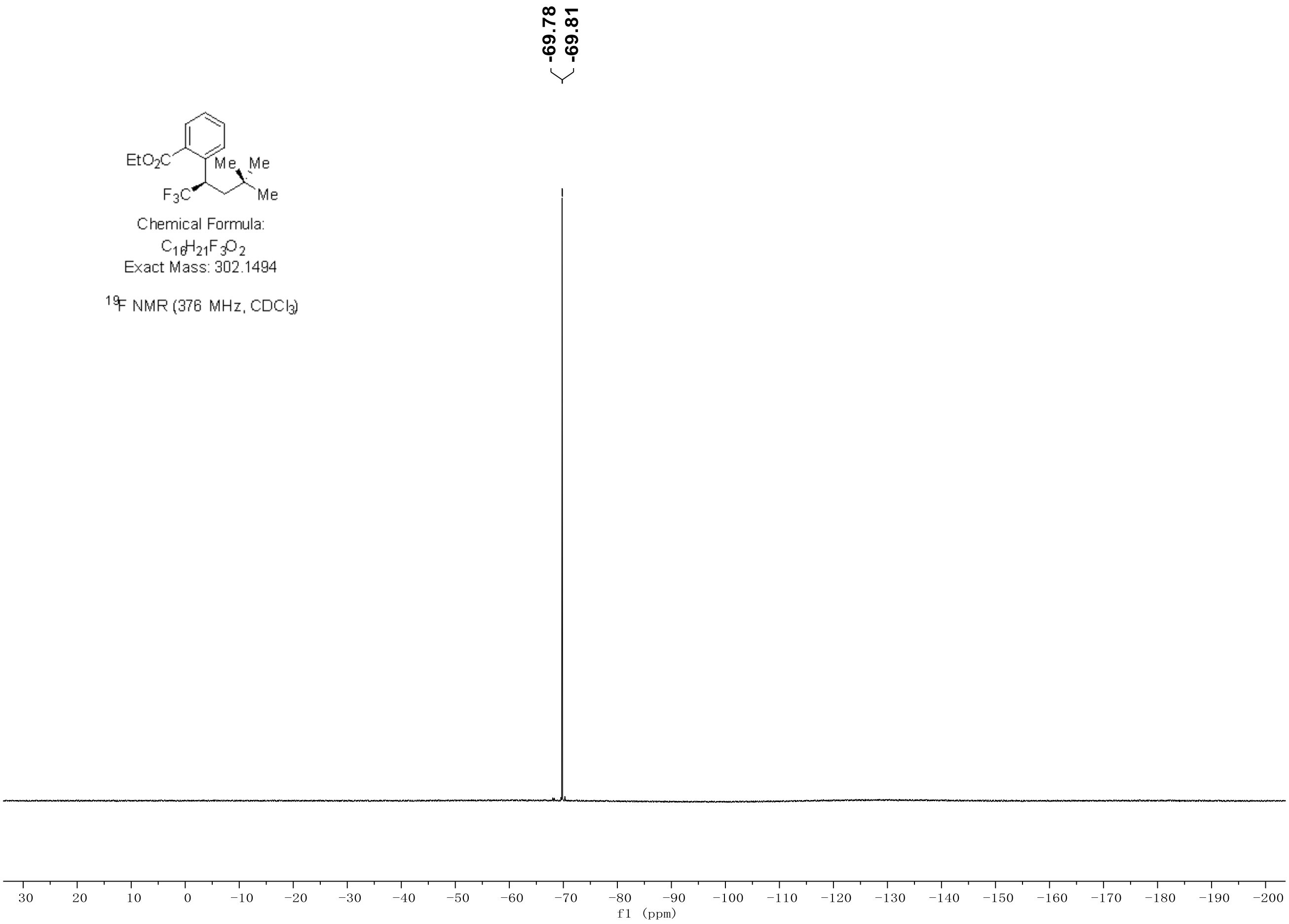

Supplementary Figure 35. ^{19}F NMR spectrum of compound 4c

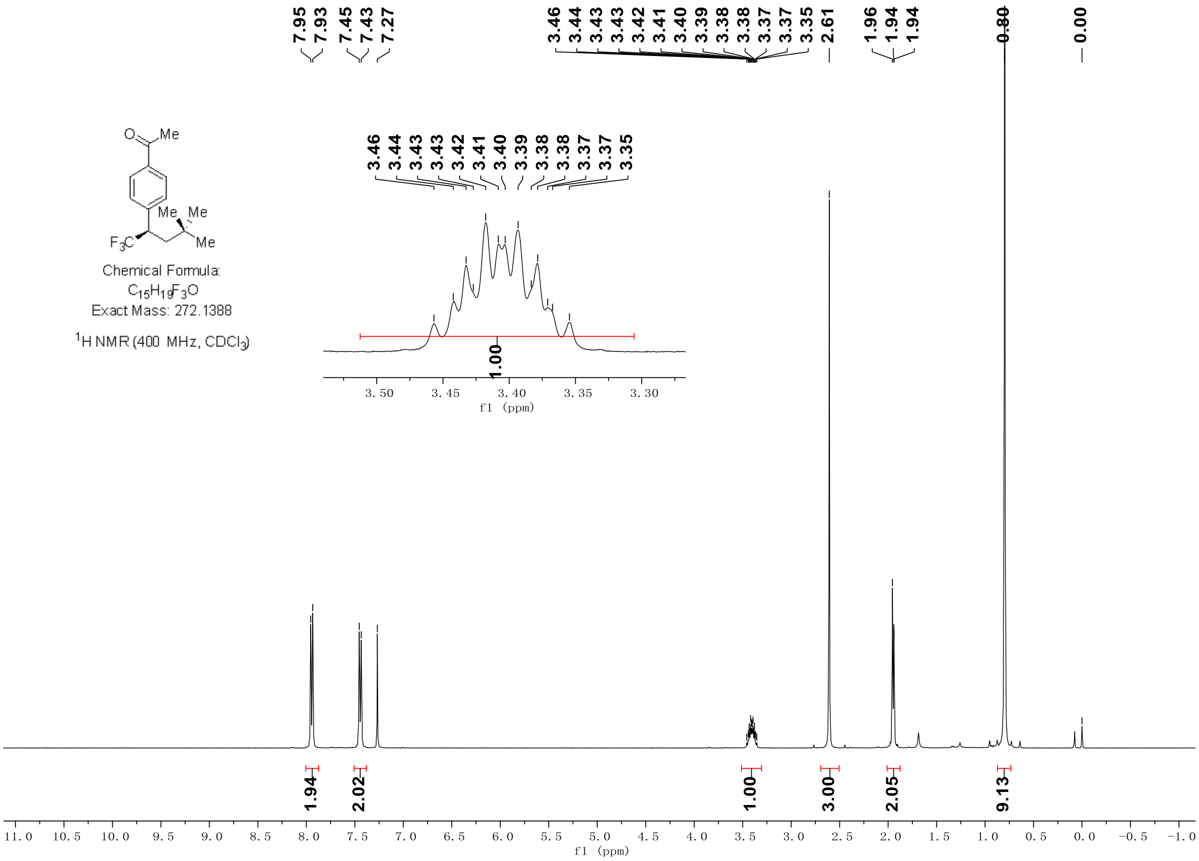

Supplementary Figure 36. ^{1}H NMR spectrum of compound 4d

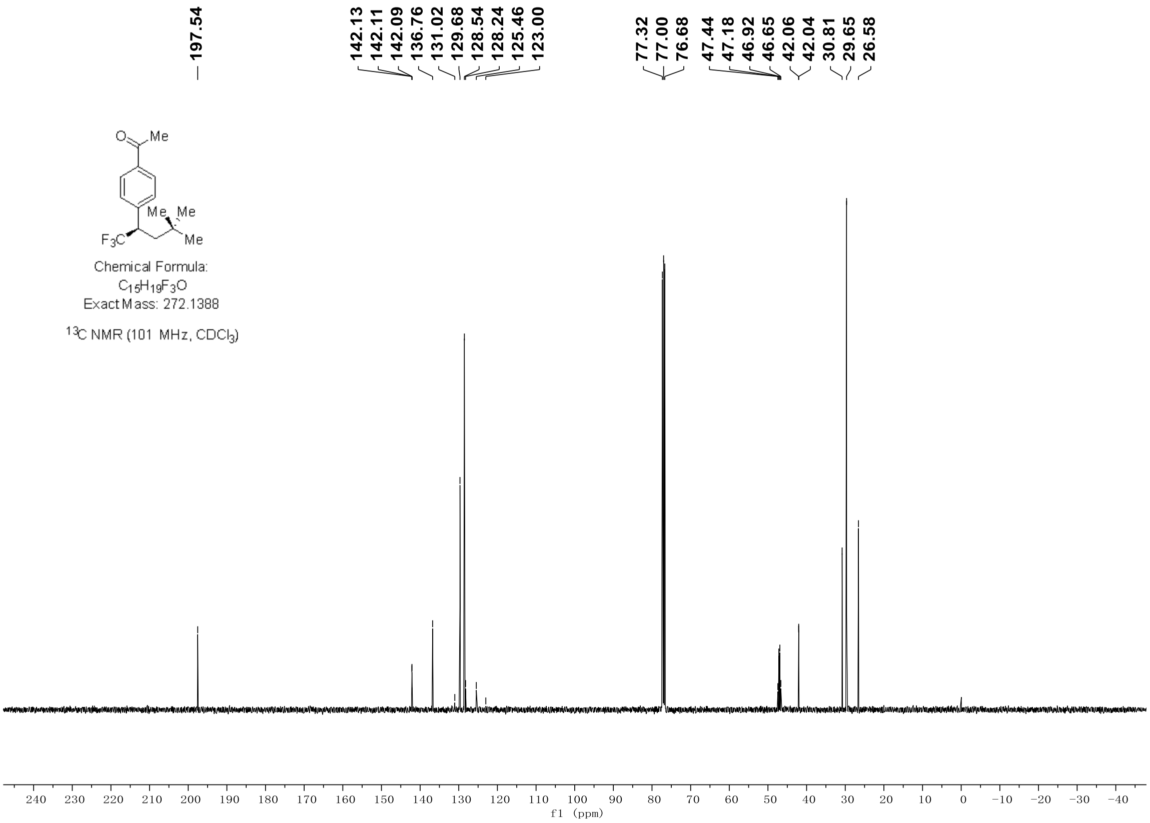

Supplementary Figure 37. ^{13}C NMR spectrum of compound 4d

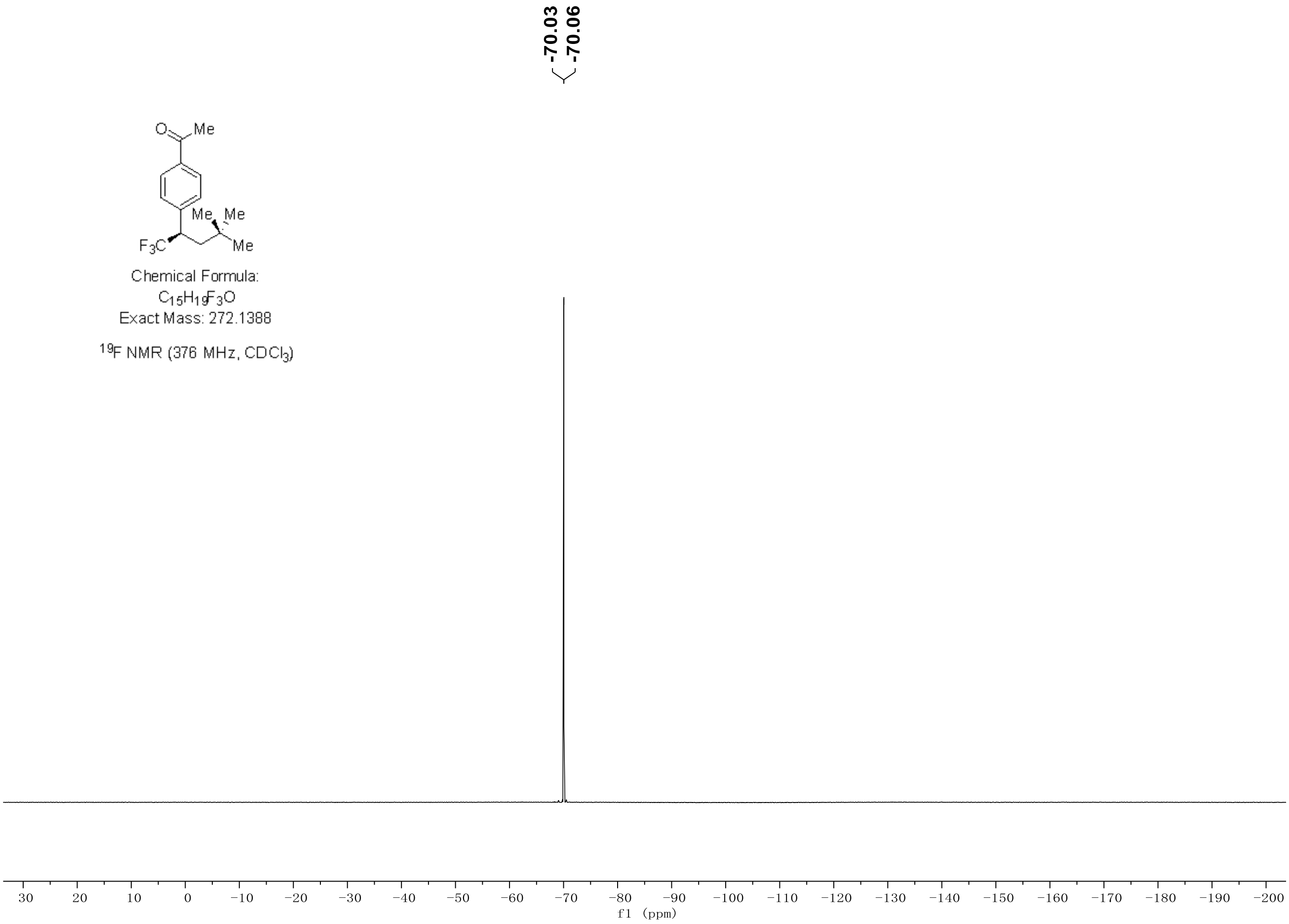

Supplementary Figure 38. ^{1}H NMR spectrum of compound 4d

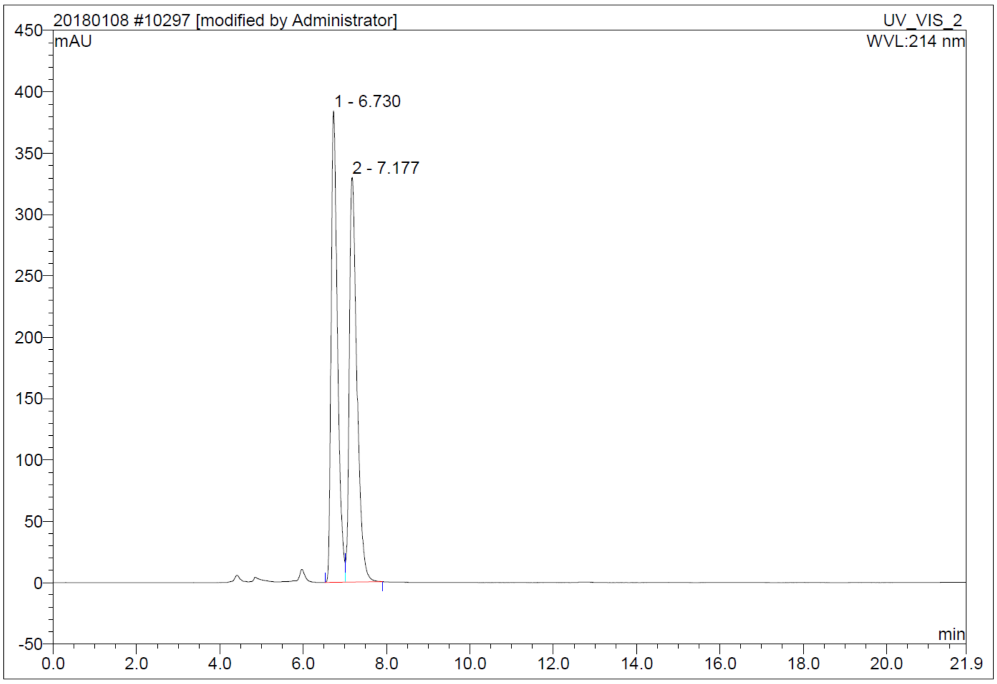

No.	Ret.Time min	Peak Name	Height mAU	Area mAU*min	Rel.Area %	Amount	Type
1	6.73	n.a.	384.417	67.566	49.07	n.a.	BM
2	7.18	n.a.	330.025	70.128	50.93	n.a.	MB
Total:			714.442	137.694	100.00	0.000	

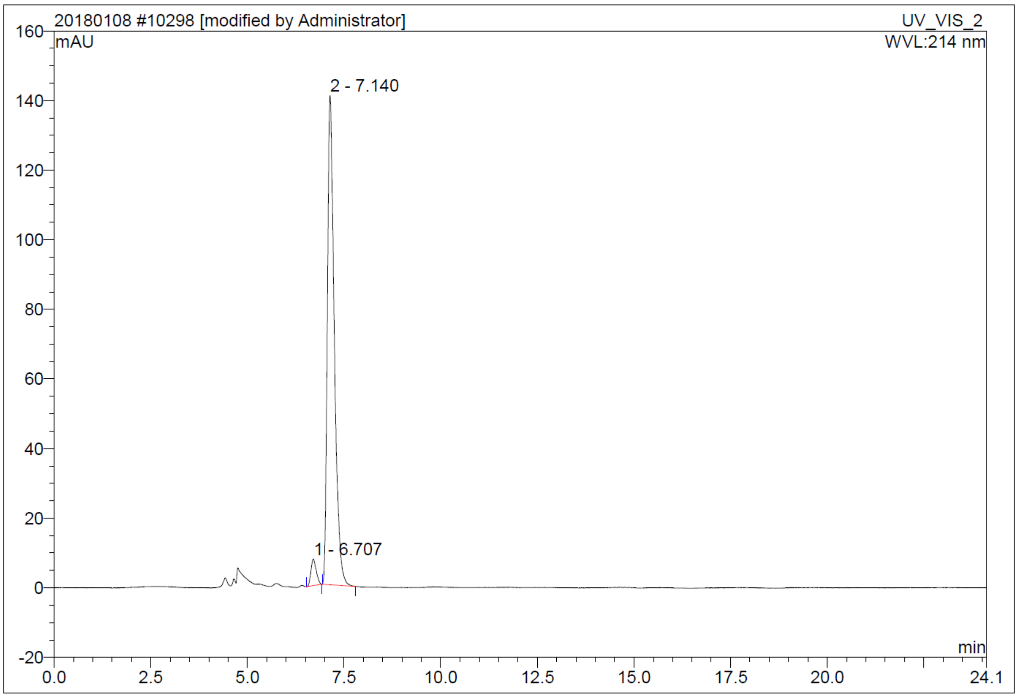

No.	Ret.Time min	Peak Name	Height mAU	Area mAU*min	Rel.Area %	Amount	Type
1	6.71	n.a.	7.666	1.210	3.99	n.a.	BMB*
2	7.14	n.a.	140.670	29.124	96.01	n.a.	BMB*
Total:			148.336	30.334	100.00	0.000	

Supplementary Figure 39. Chiral HPLC analysis of compound 4d

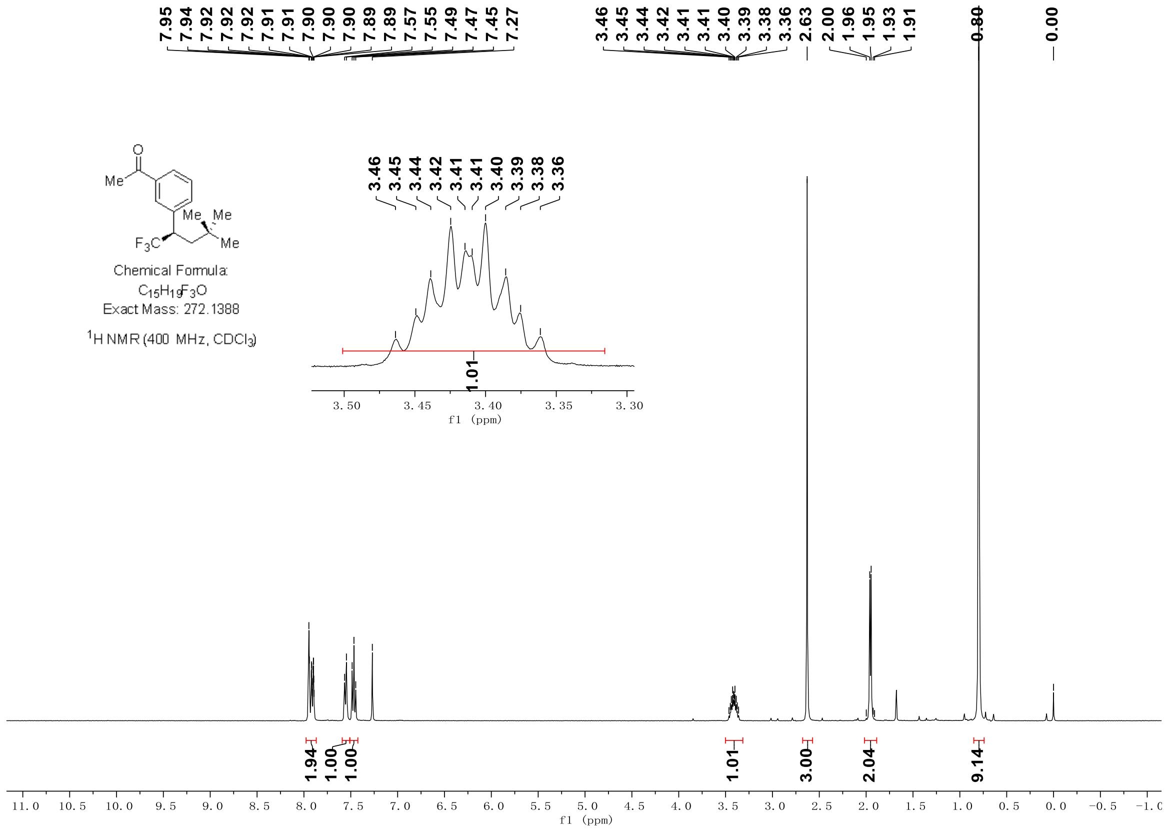

Supplementary Figure 40. ^{1}H NMR spectrum of compound 4e

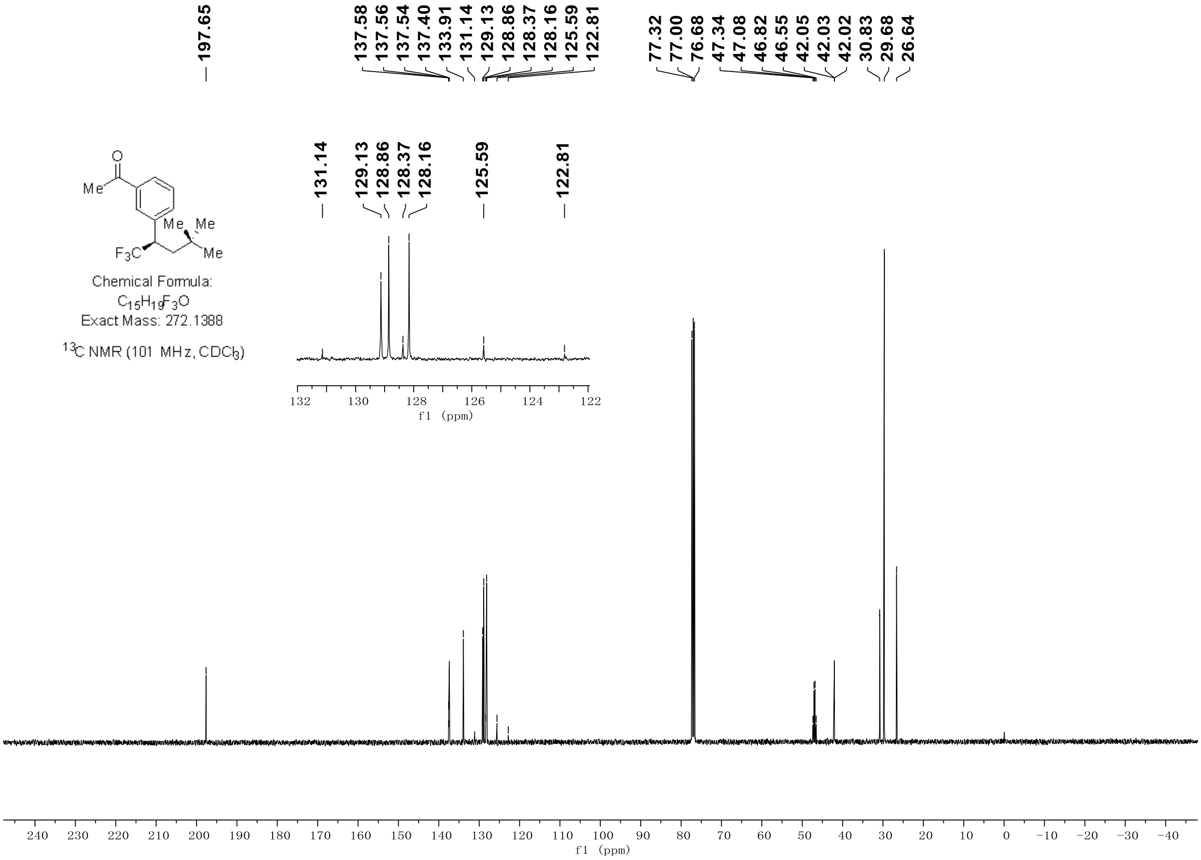

Supplementary Figure 41. ^{13}C NMR spectrum of compound 4e

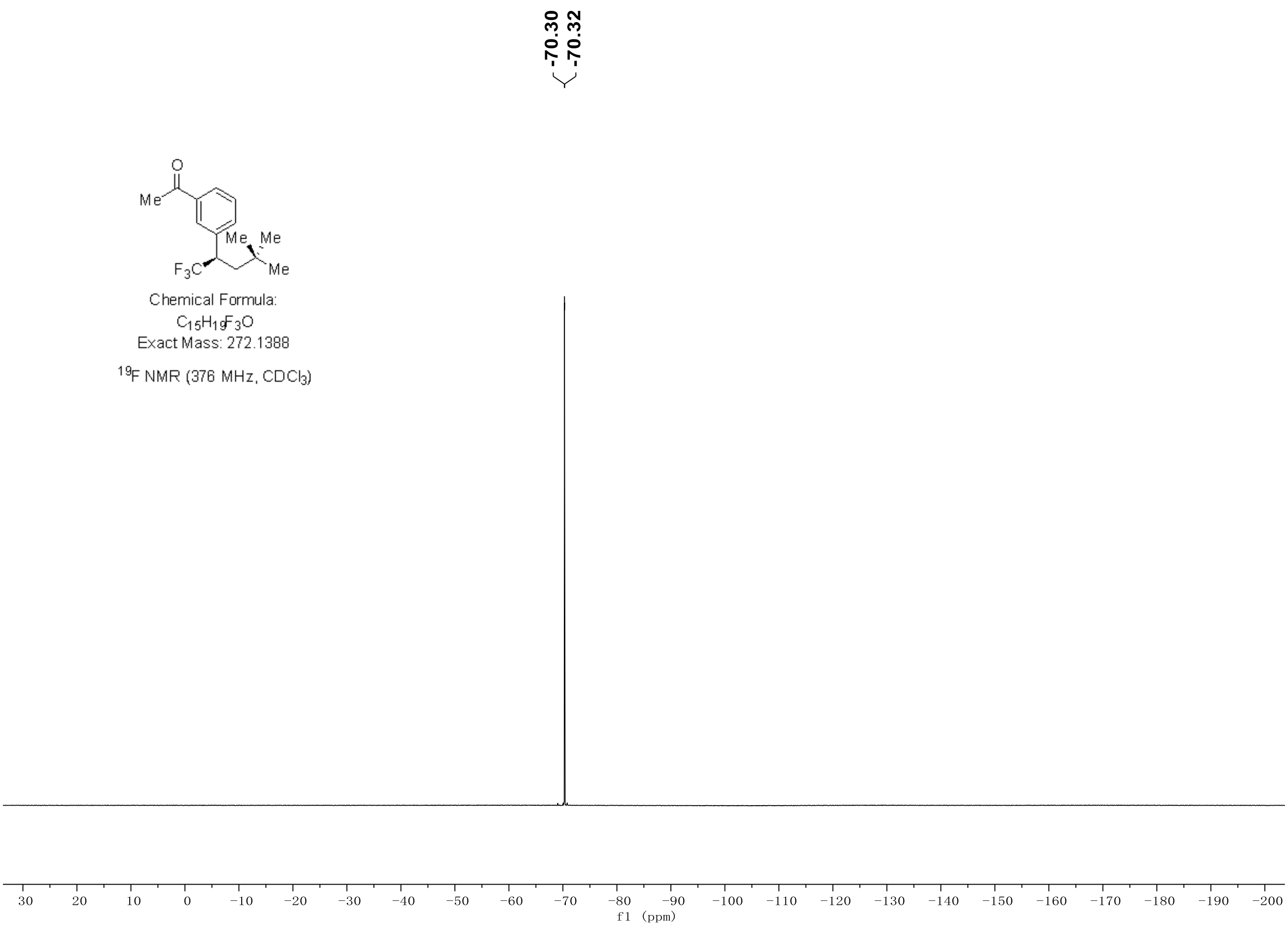

Supplementary Figure 42. ^{19}F NMR spectrum of compound 4e

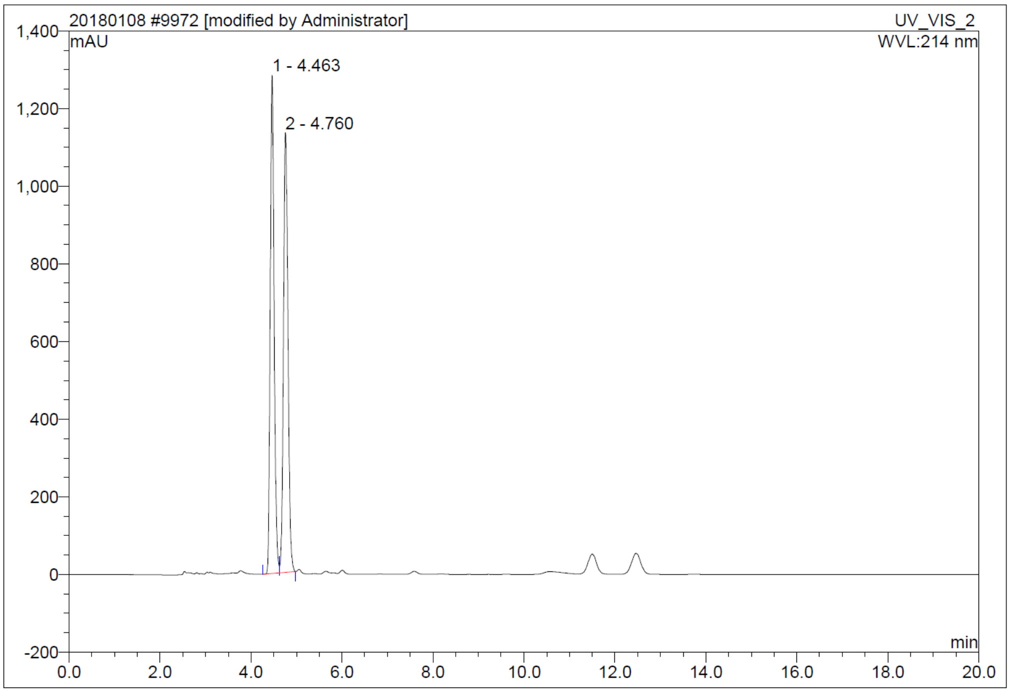

No.	Ret.Time min	Peak Name	Height mAU	Area mAU*min	Rel.Area %	Amount	Type
1	4.46	n.a.	1282.809	128.108	49.73	n.a.	BM *
2	4.76	n.a.	1132.410	129.502	50.27	n.a.	MB*
Total:			2415.219	257.610	100.00	0.000	

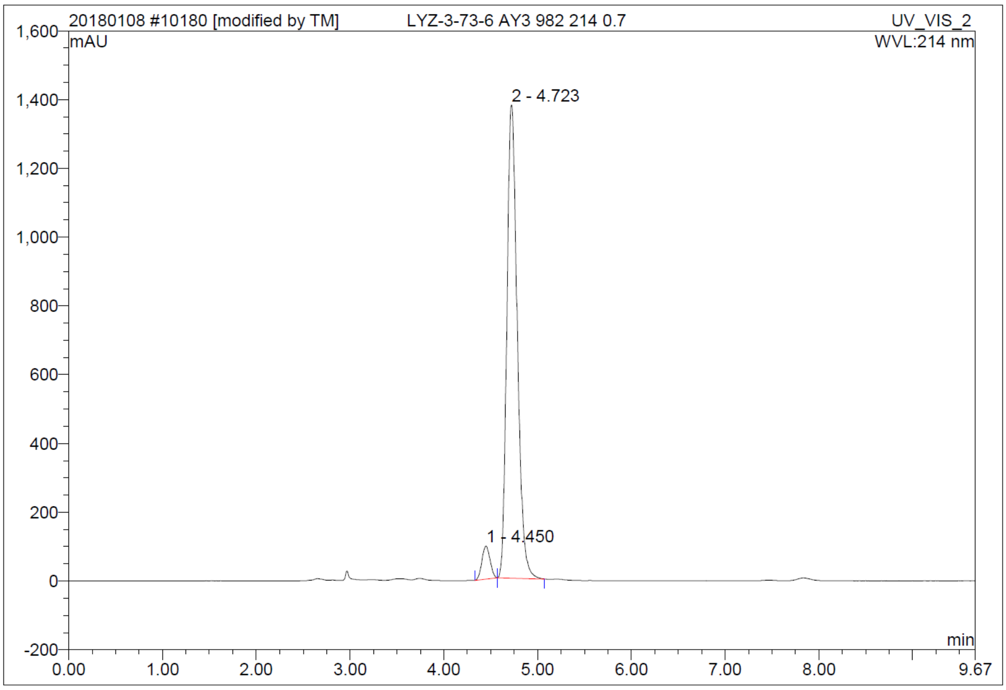

No.	Ret.Time min	Peak Name	Height mAU	Area mAU*min	Rel.Area %	Amount	Type
1	4.45	n.a.	95.842	9.605	5.16	n.a.	BMb*
2	4.72	n.a.	1376.813	176.652	94.84	n.a.	bMB*
Total:			1472.655	186.257	100.00	0.000	

Supplementary Figure 43. Chiral HPLC analysis of compound 4e

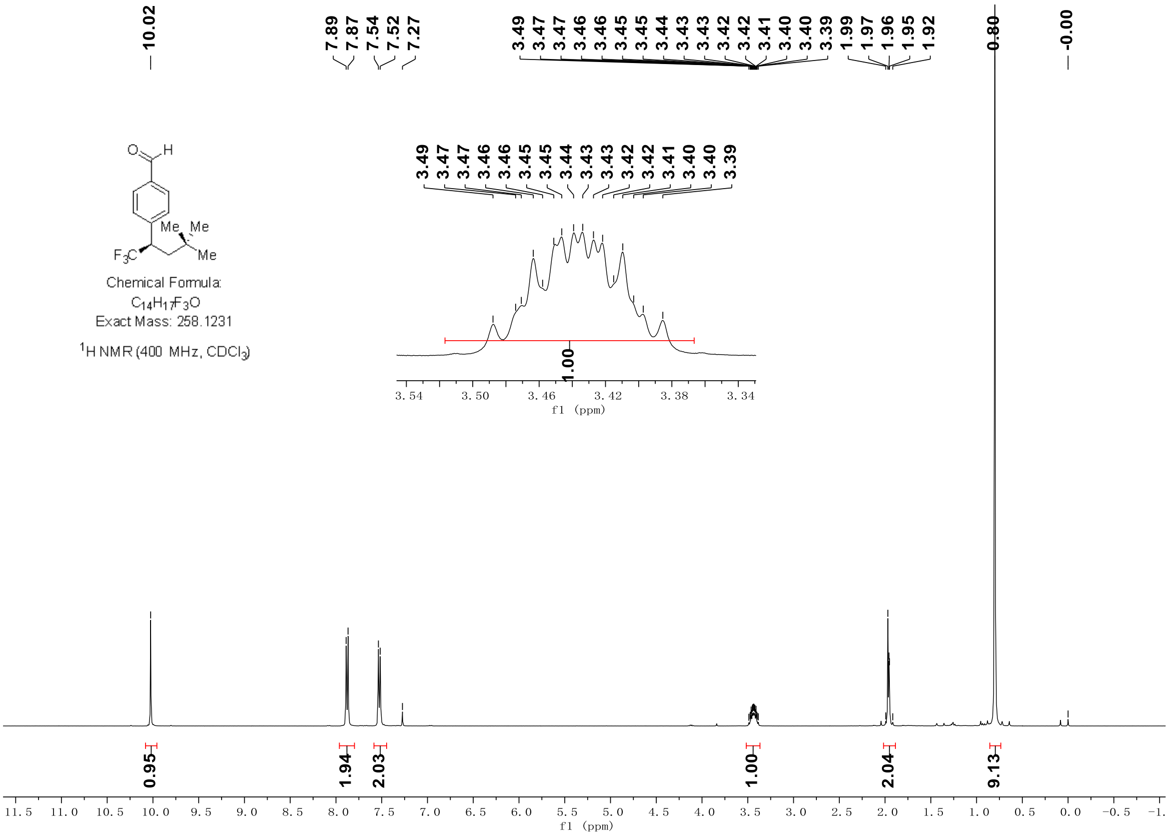

Supplementary Figure 44. ^{1}**H NMR spectrum of compound 4f**

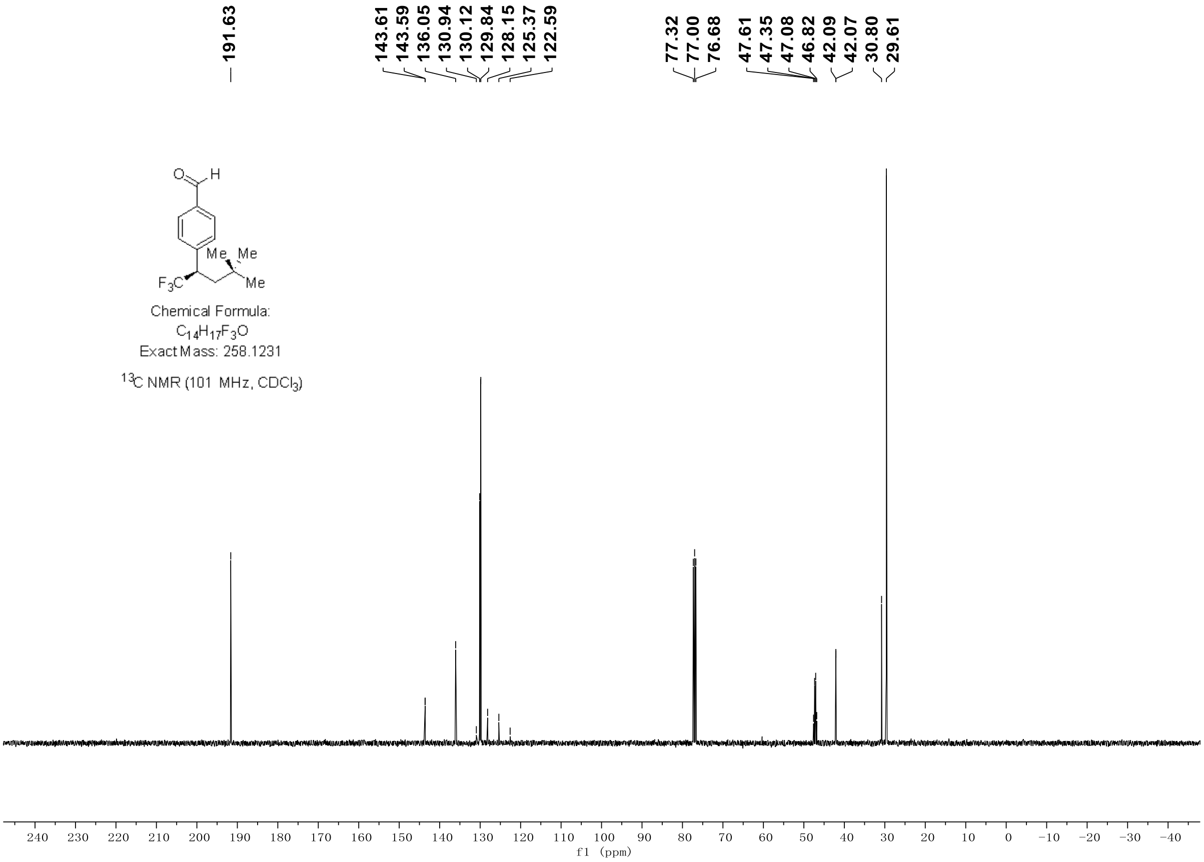

Supplementary Figure 45. ^{13}**C NMR spectrum of compound 4f**

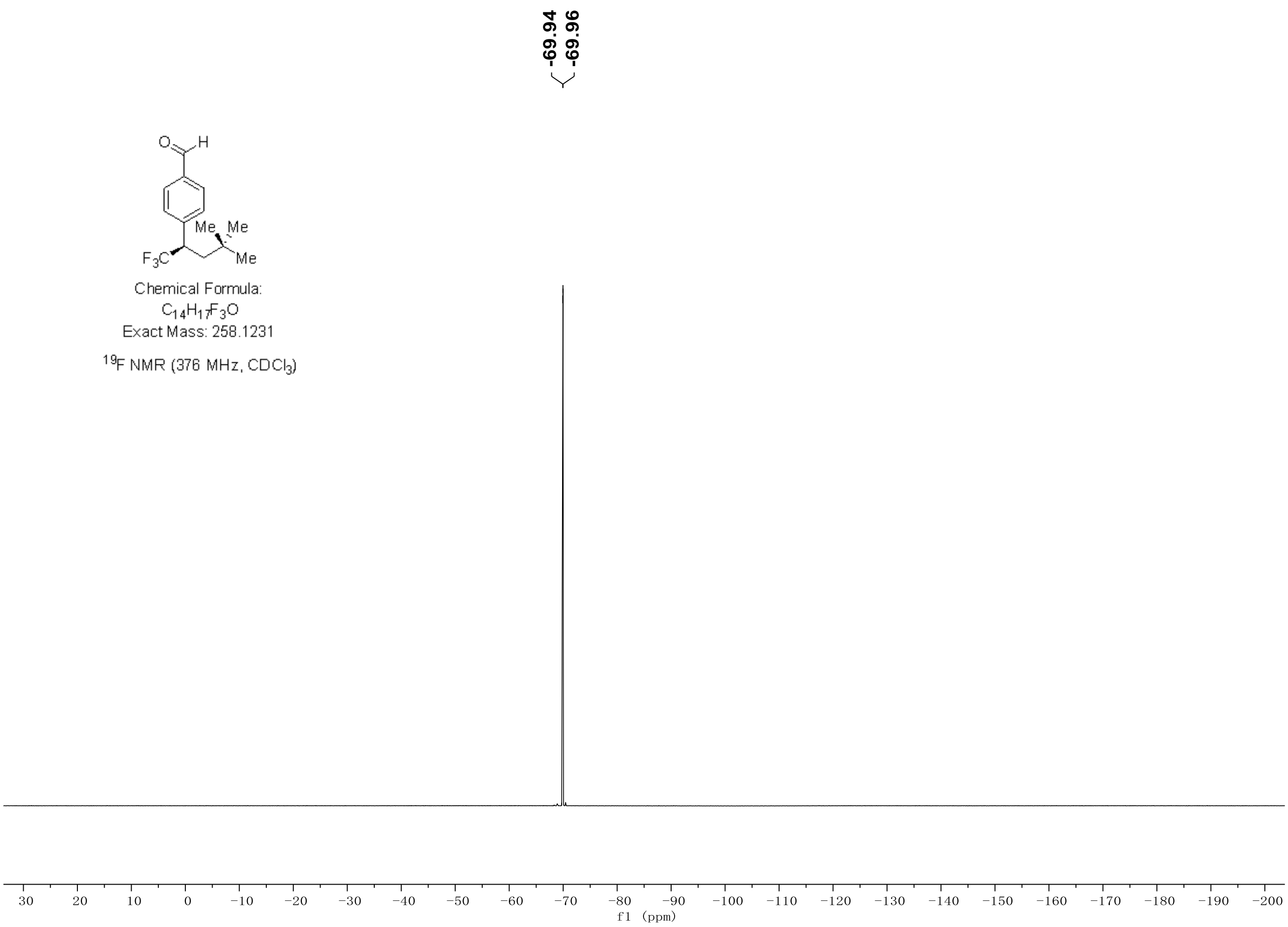

Supplementary Figure 46. ^{19}F NMR spectrum of compound 4f

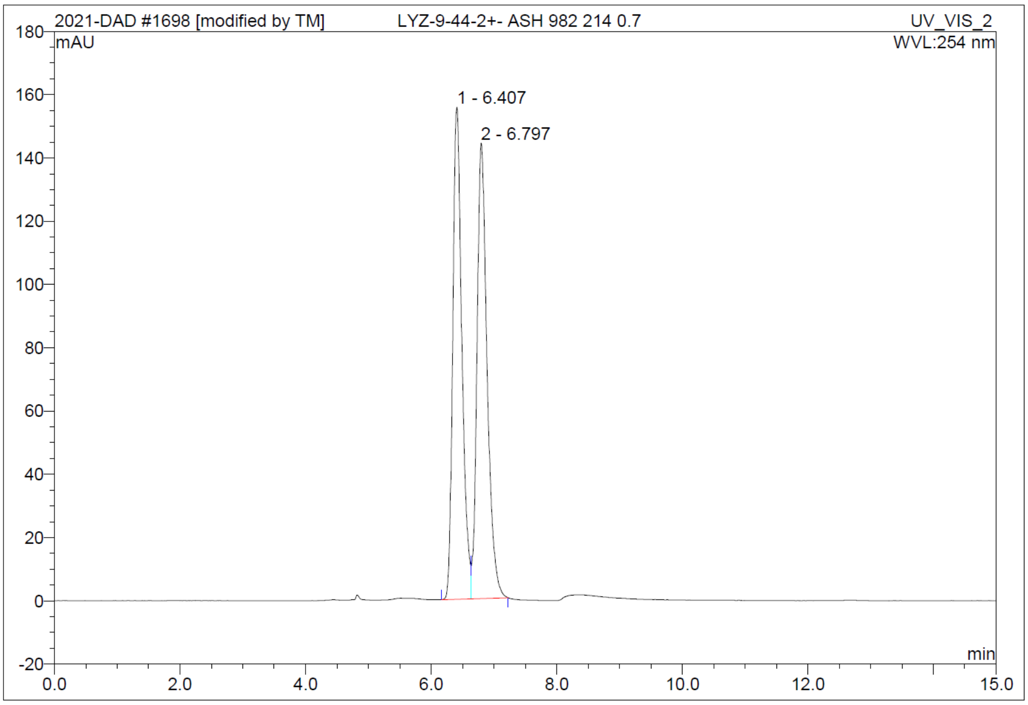

No.	Ret.Time min	Peak Name	Height mAU	Area mAU*min	Rel.Area %	Amount	Type
1	6.41	n.a.	155.631	26.167	49.32	n.a.	BM *
2	6.80	n.a.	143.989	26.889	50.68	n.a.	MB*
Total:			299.620	53.056	100.00	0.000	

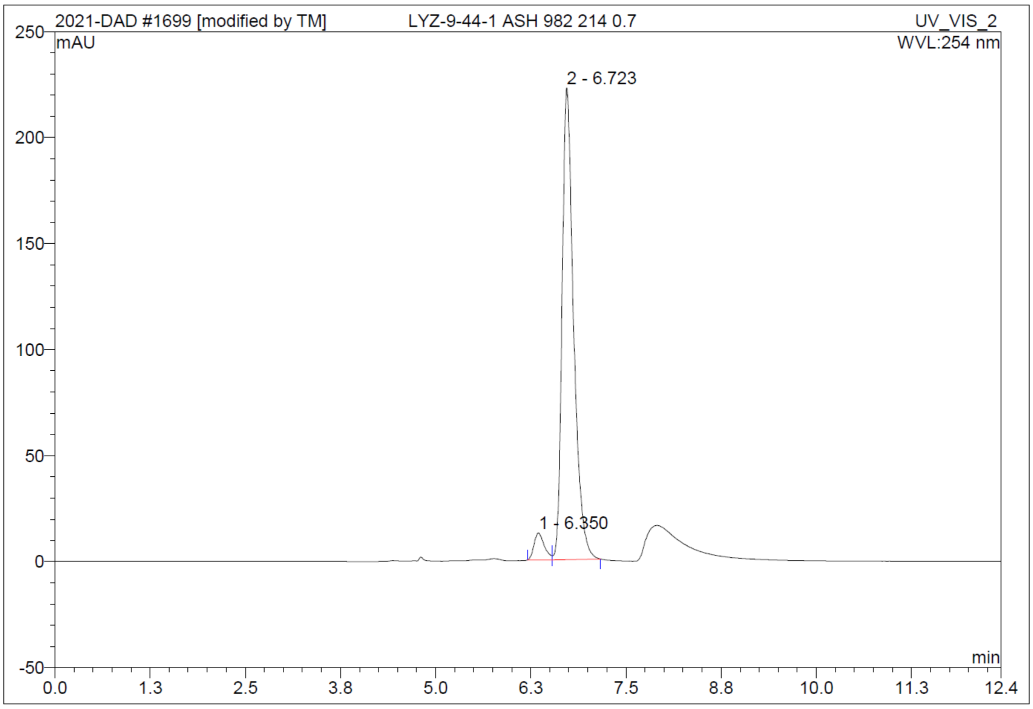

No.	Ret.Time min	Peak Name	Height mAU	Area mAU*min	Rel.Area %	Amount	Type
1	6.35	n.a.	12.869	2.009	4.89	n.a.	BM *
2	6.72	n.a.	222.571	39.073	95.11	n.a.	MB*
Total:			235.440	41.082	100.00	0.000	

Supplementary Figure 47. Chiral HPLC analysis of compound 4e

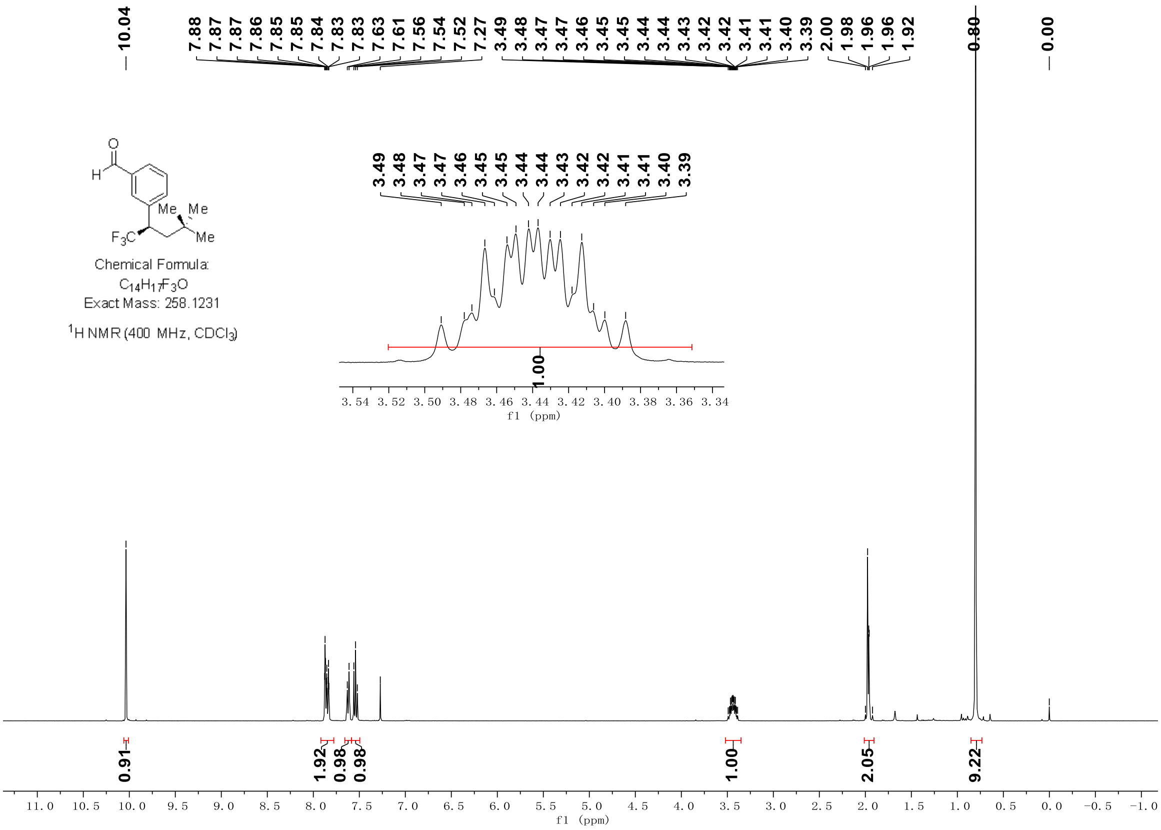

Supplementary Figure 48. ^1H NMR spectrum of compound 4g

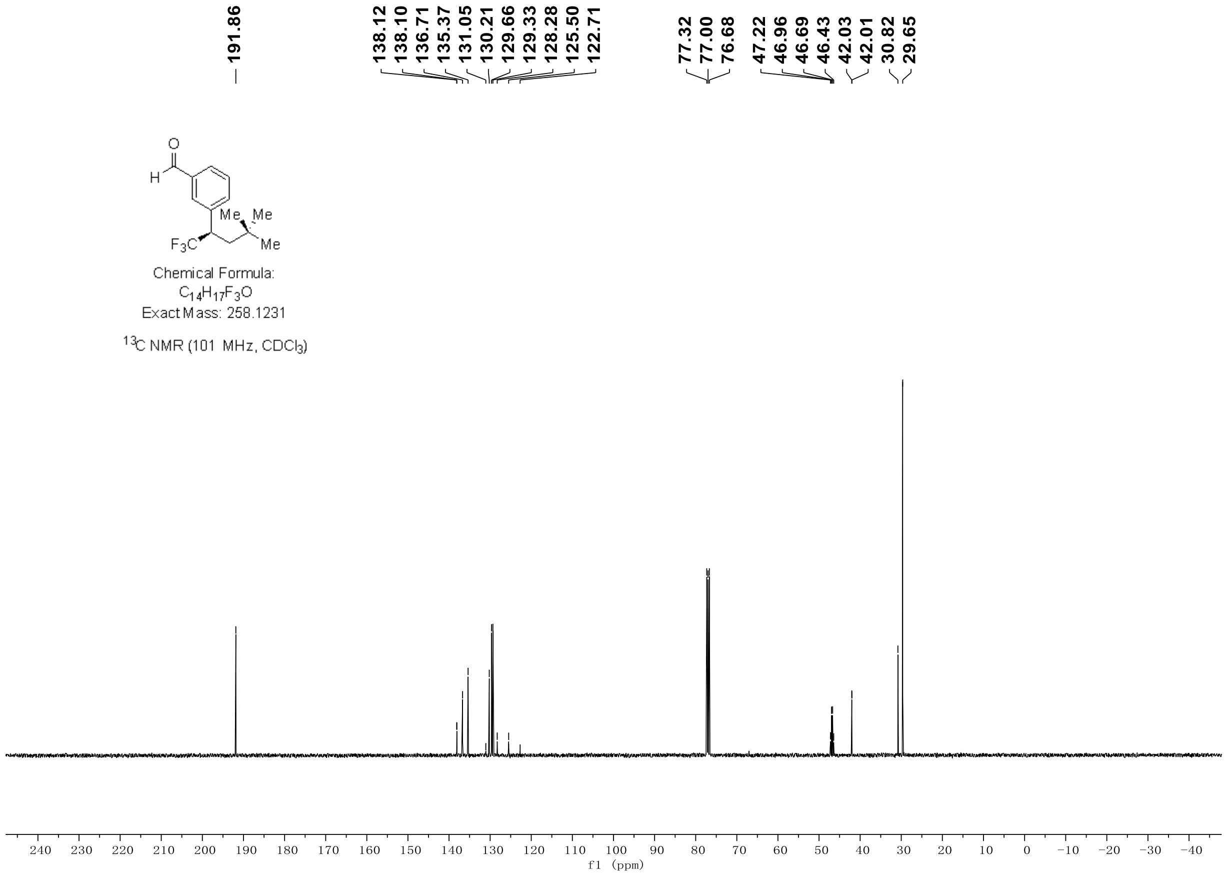

Supplementary Figure 49. ^{13}C NMR spectrum of compound 4g

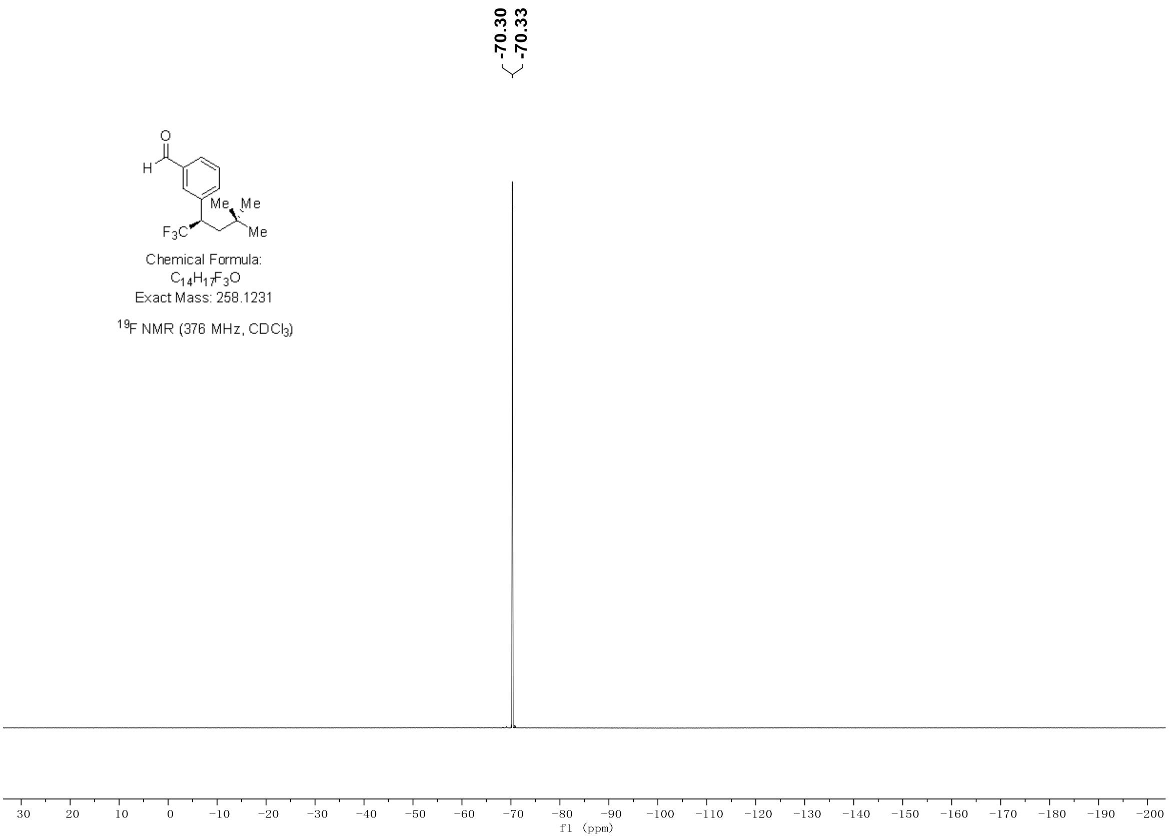

Supplementary Figure 50. ^{19}F NMR spectrum of compound 4g

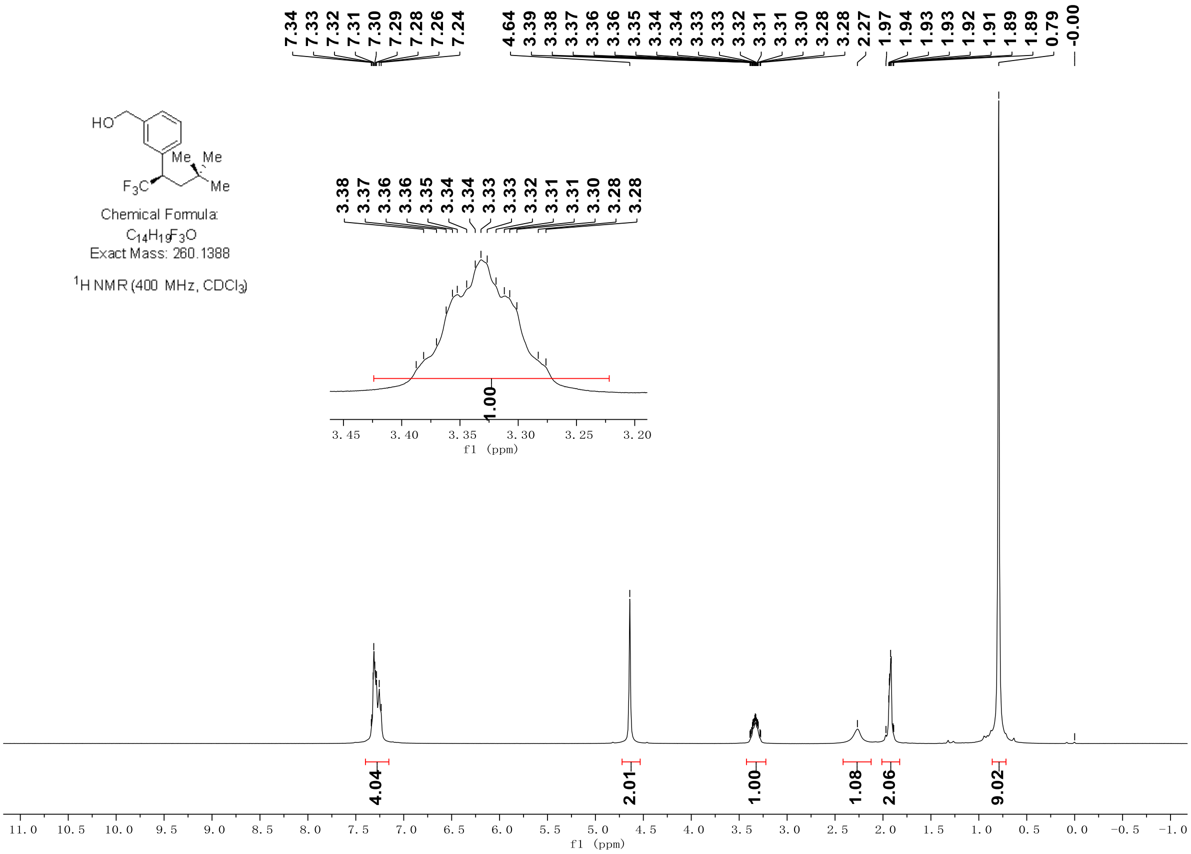

Supplementary Figure 51. ^{1}H NMR spectrum of compound 4g'

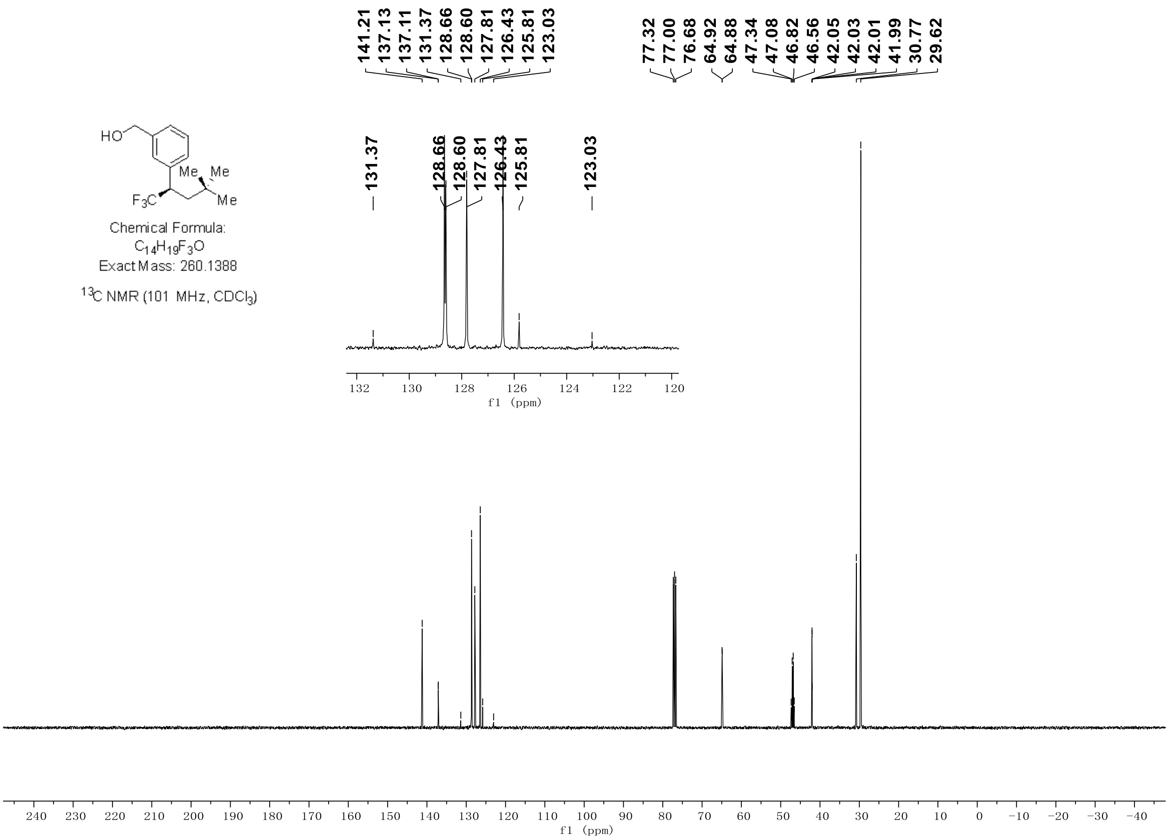

Supplementary Figure 52. ^{13}**C NMR spectrum of compound 4g'**

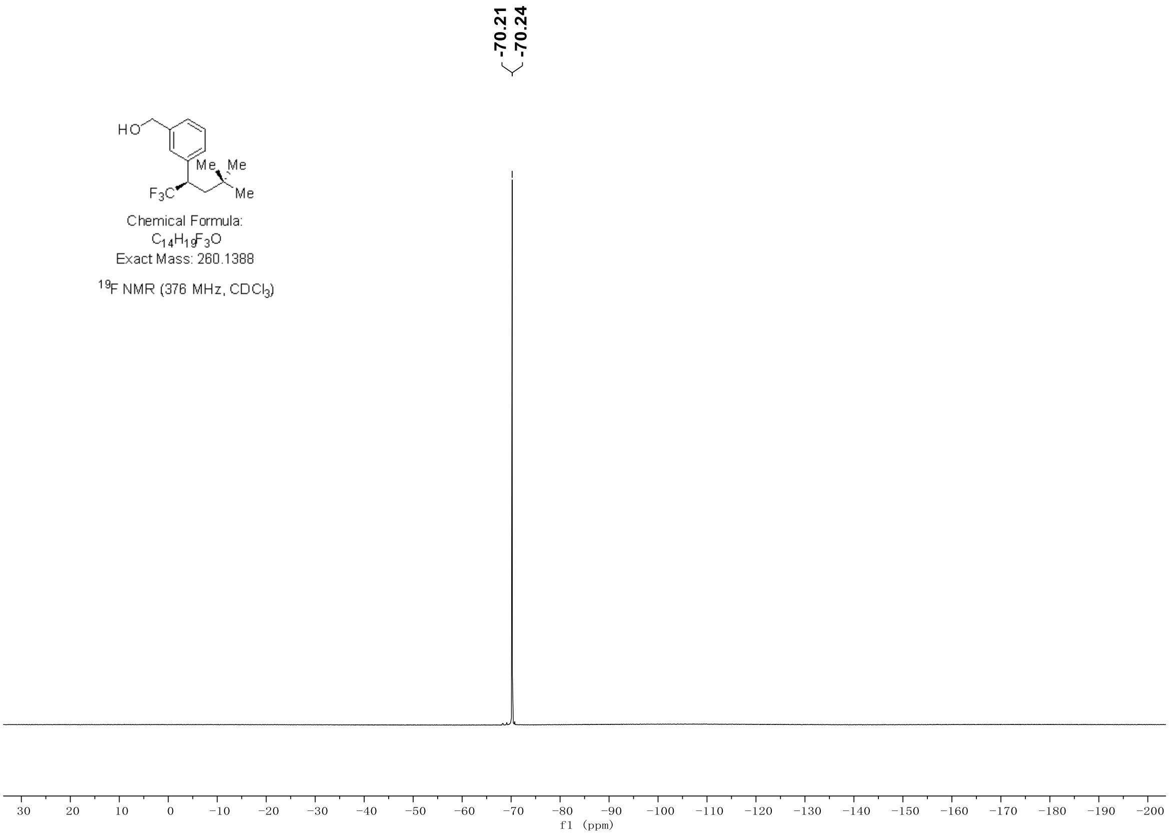

Supplementary Figure 53. ^{19}**F NMR spectrum of compound 4g'**

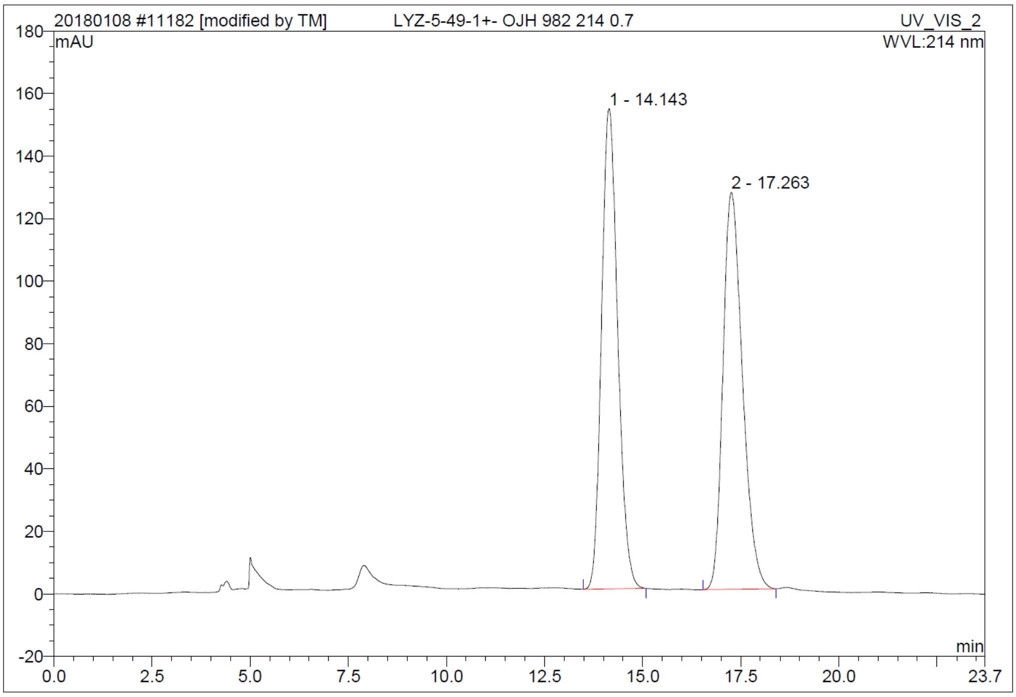

No.	Ret.Time min	Peak Name	Height mAU	Area mAU*min	Rel.Area %	Amount	Type
1	14.14	n.a.	153.672	73.407	50.06	n.a.	BMB
2	17.26	n.a.	126.986	73.228	49.94	n.a.	BMB*
Total:			280.658	146.635	100.00	0.000	

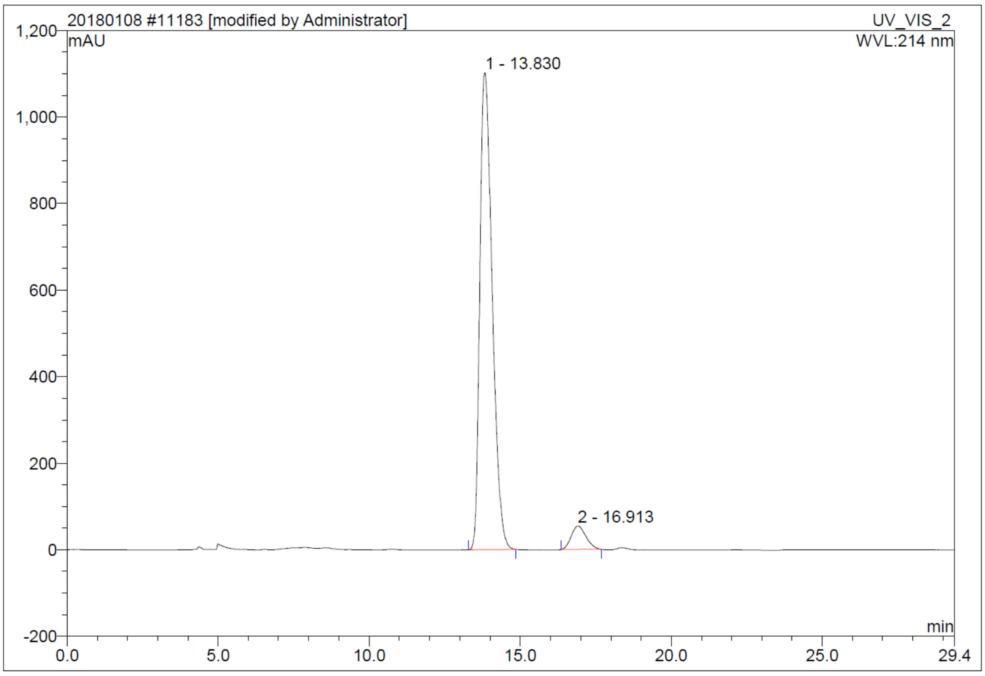

No.	Ret.Time min	Peak Name	Height mAU	Area mAU*min	Rel.Area %	Amount	Type
1	13.83	n.a.	1101.828	523.143	94.48	n.a.	BMB*
2	16.91	n.a.	54.066	30.572	5.52	n.a.	BMB*
Total:			1155.894	553.715	100.00	0.000	

Supplementary Figure 54. Chiral HPLC analysis of compound 4g'

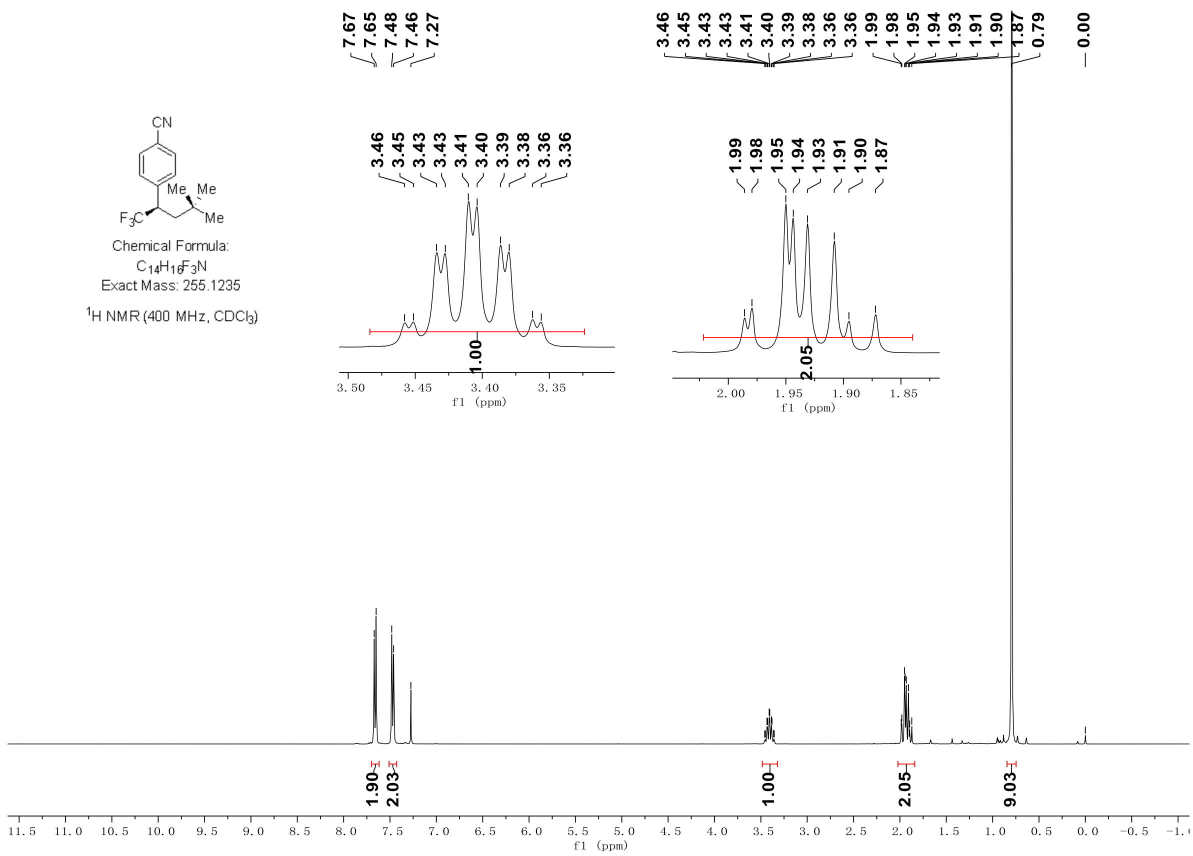

Supplementary Figure 55. ^{1}H NMR spectrum of compound 4h

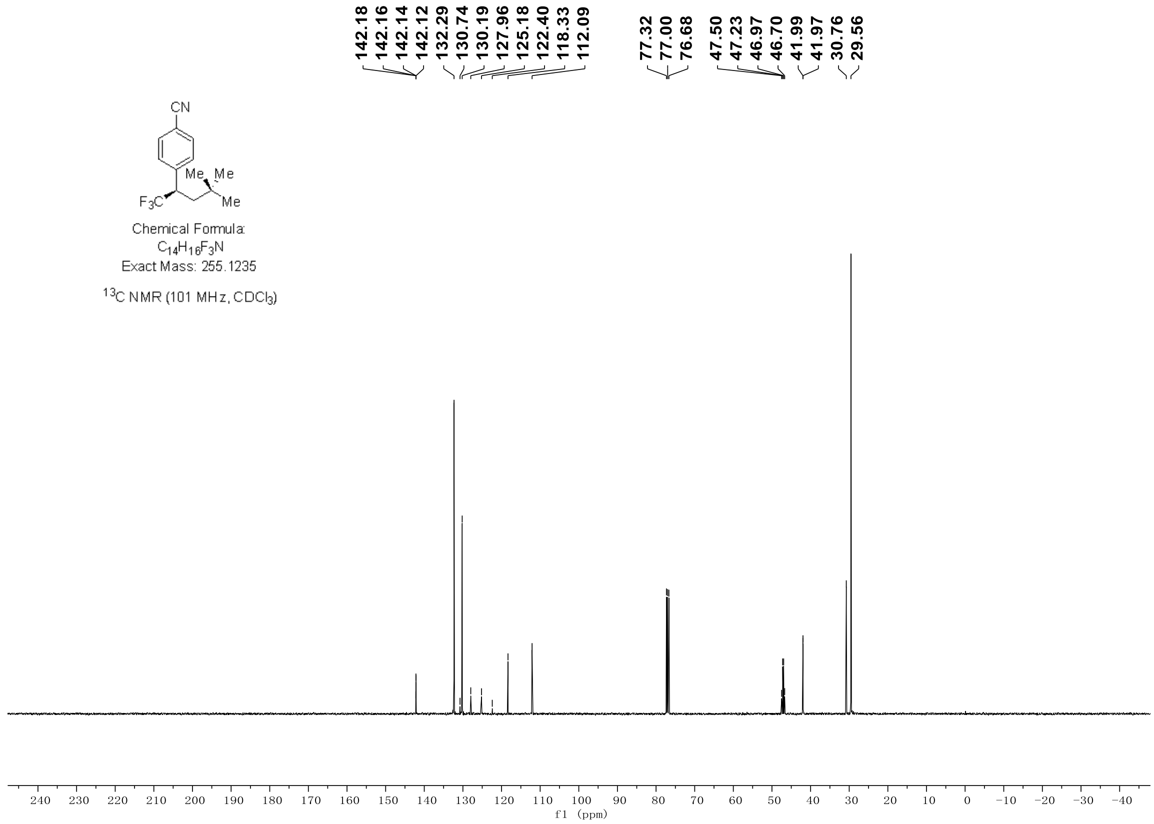

Supplementary Figure 56. ^{13}C NMR spectrum of compound 4h

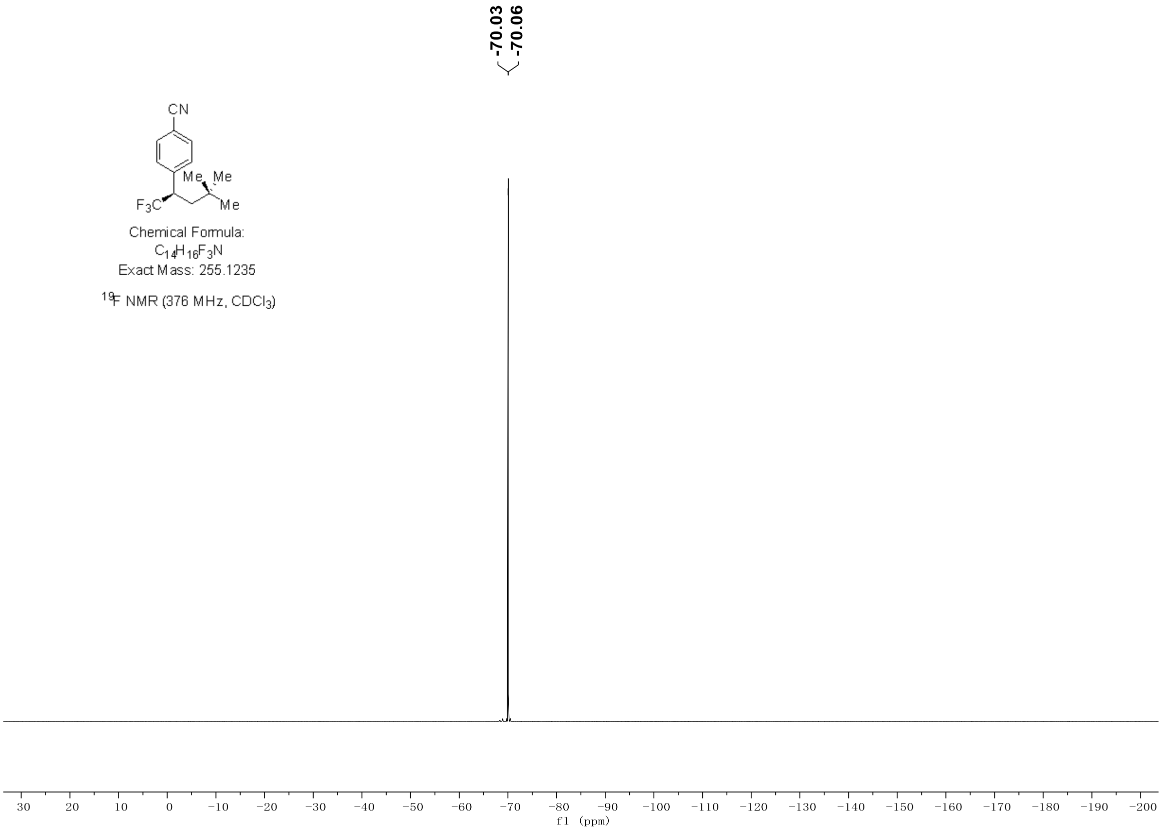

Supplementary Figure 57. ^{19}F NMR spectrum of compound 4h

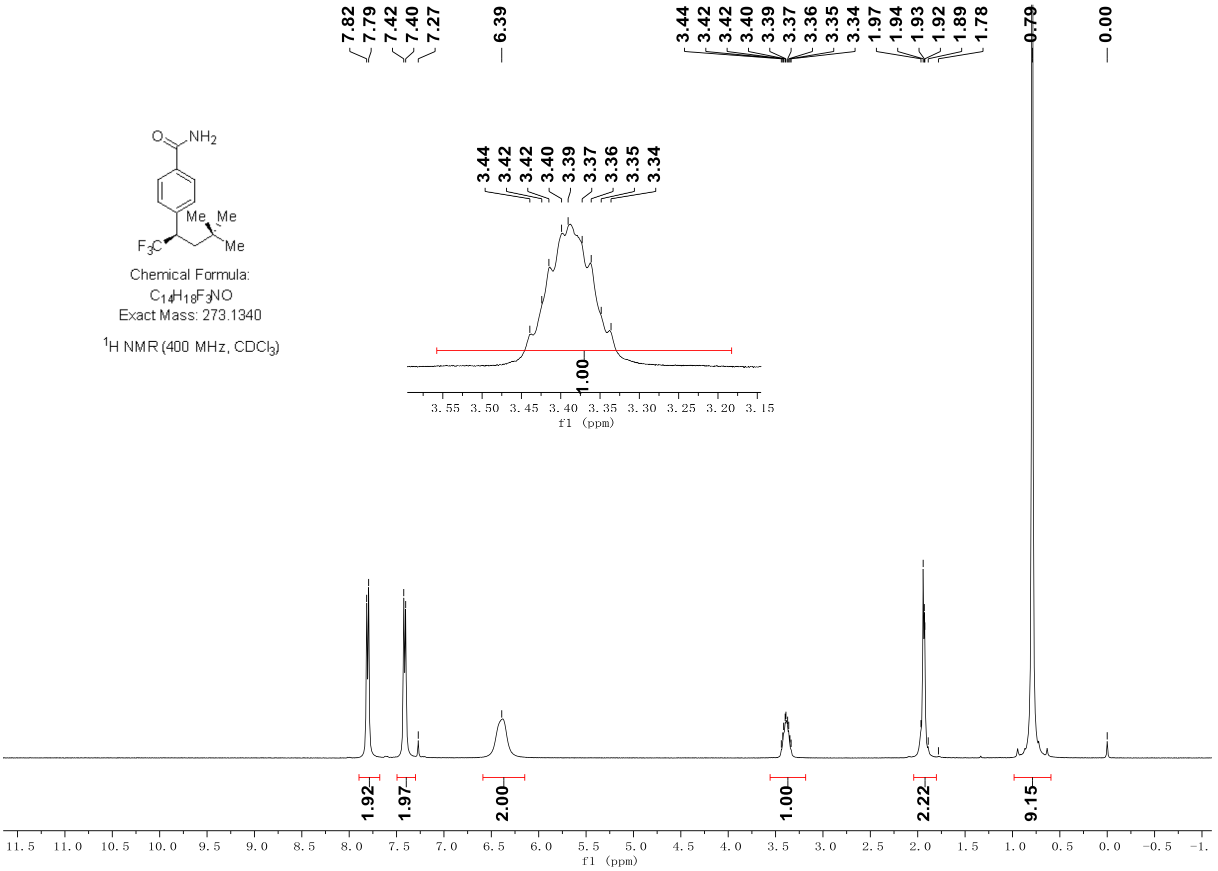

Supplementary Figure 58. ^{1}H NMR spectrum of compound 4h'

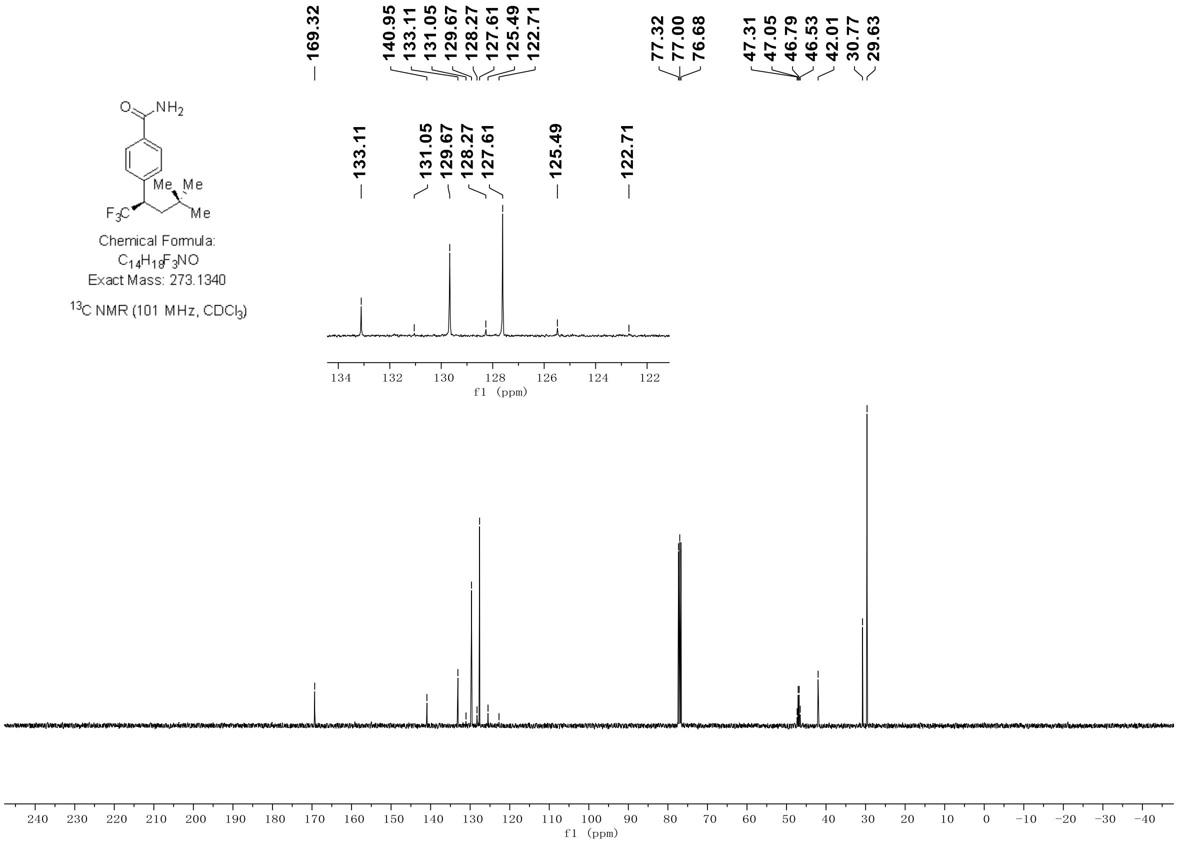

Supplementary Figure 59. ^{13}C NMR spectrum of compound 4h'

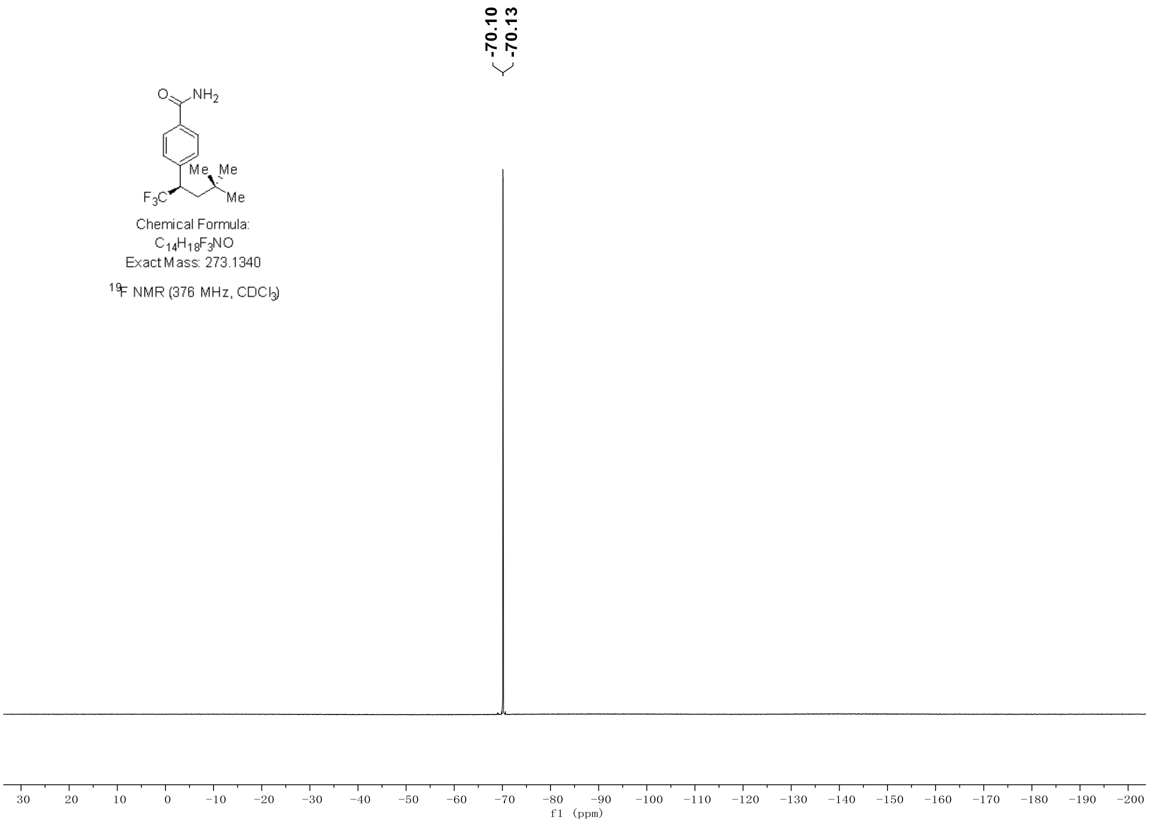

Supplementary Figure 60. ^{19}F NMR spectrum of compound 4h'

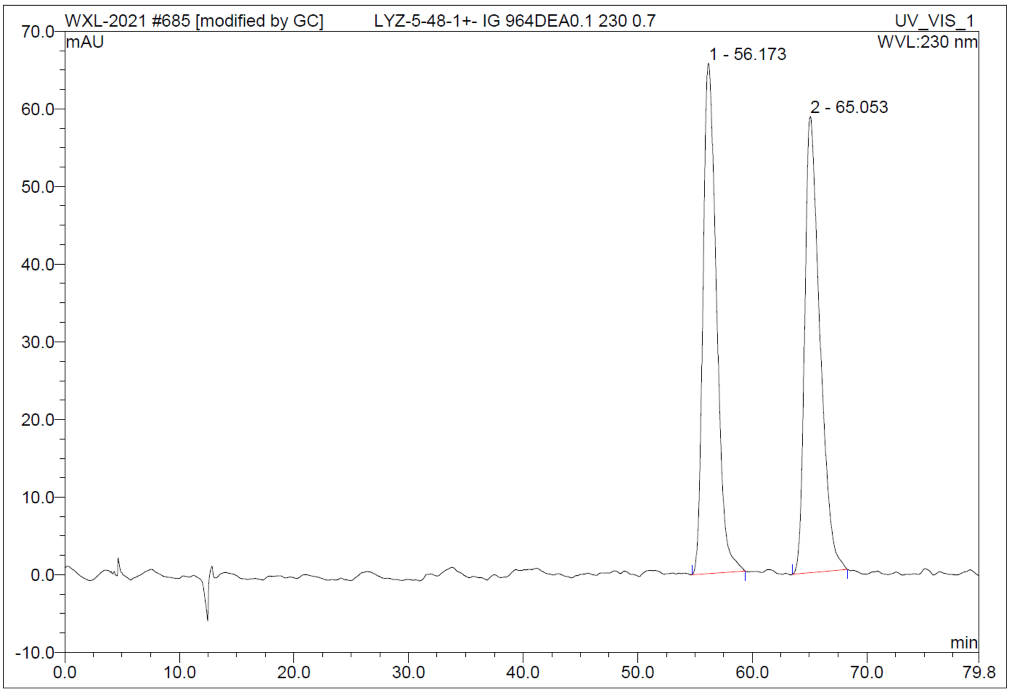

No.	Ret.Time min	Peak Name	Height mAU	Area mAU*min	Rel.Area %	Amount	Type
1	56.17	n.a.	65.699	90.034	49.87	n.a.	BMB
2	65.05	n.a.	58.757	90.505	50.13	n.a.	BMB
Total:			124.455	180.539	100.00	0.000	

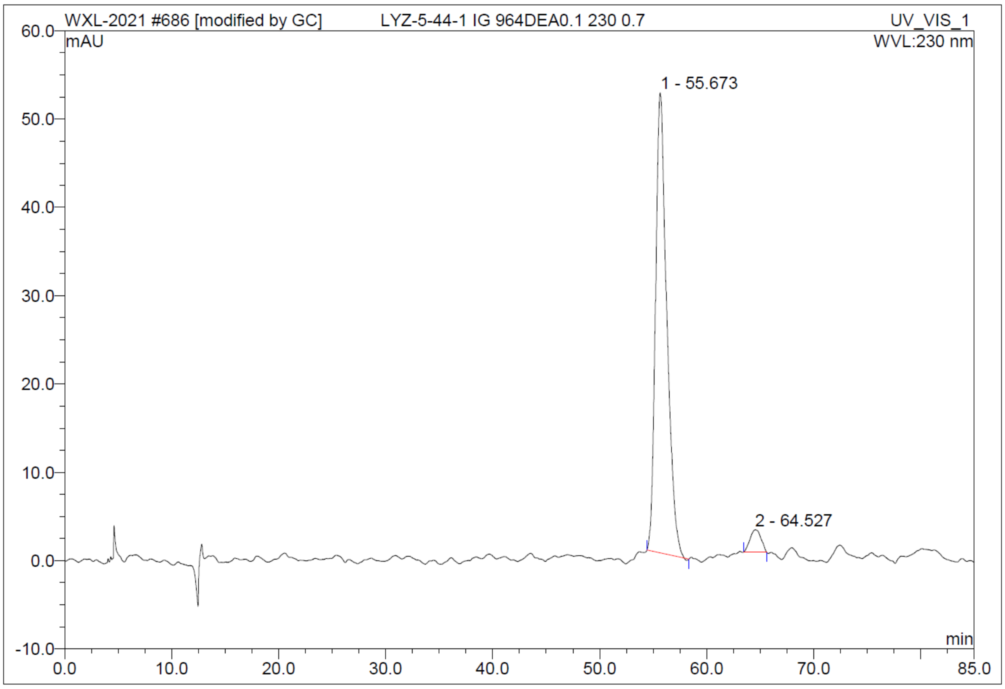

No.	Ret.Time min	Peak Name	Height mAU	Area mAU*min	Rel.Area %	Amount	Type
1	55.67	n.a.	52.106	63.394	95.54	n.a.	BMB*
2	64.53	n.a.	2.514	2.961	4.46	n.a.	BMB*
Total:			54.621	66.354	100.00	0.000	

Supplementary Figure 61. Chiral HPLC analysis of compound 4h'

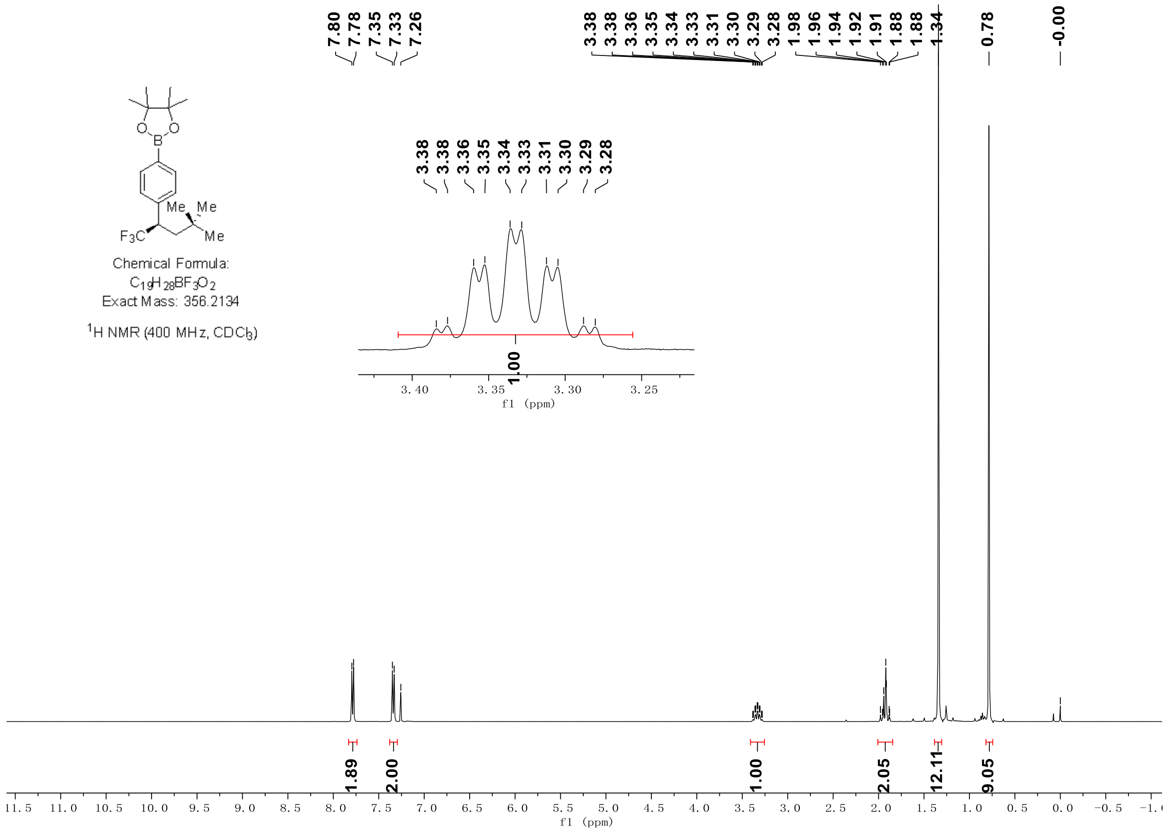

Supplementary Figure 62. ^{1}**H NMR spectrum of compound 4i**

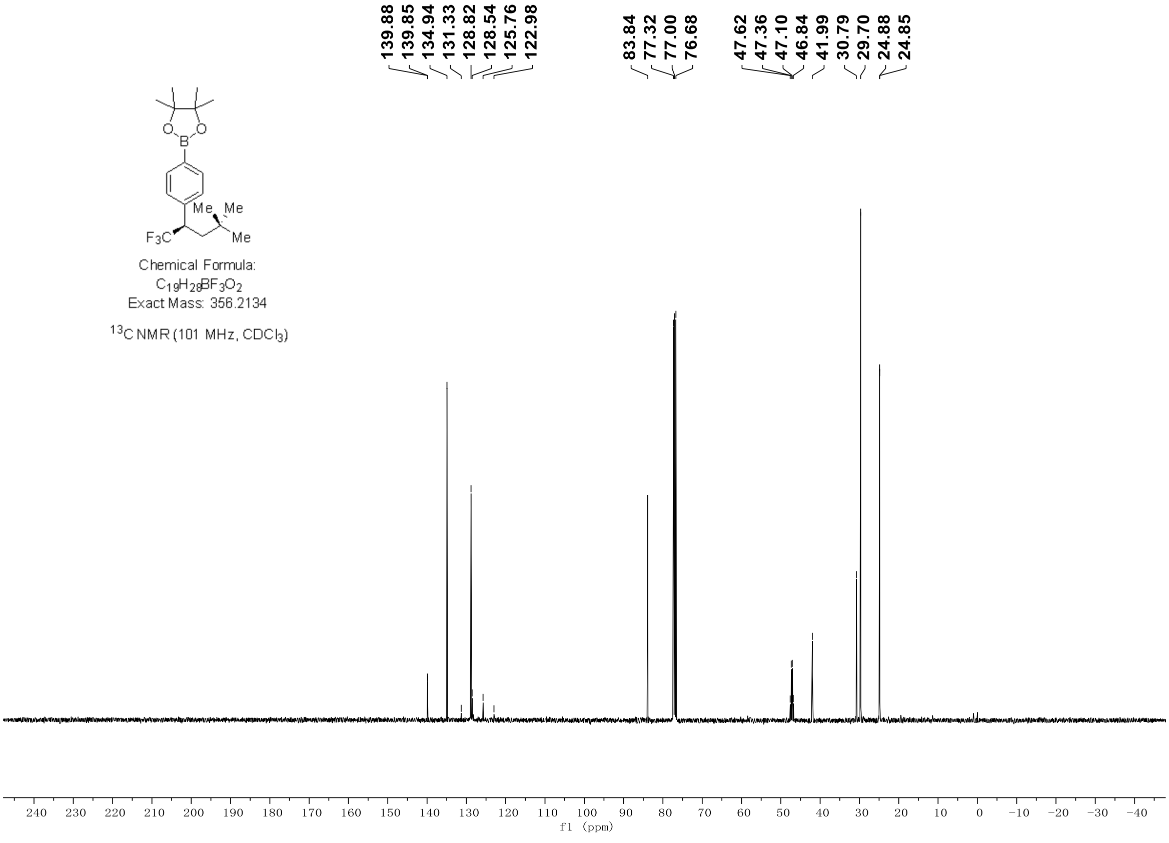

Supplementary Figure 63. ^{13}**C NMR spectrum of compound 4i**

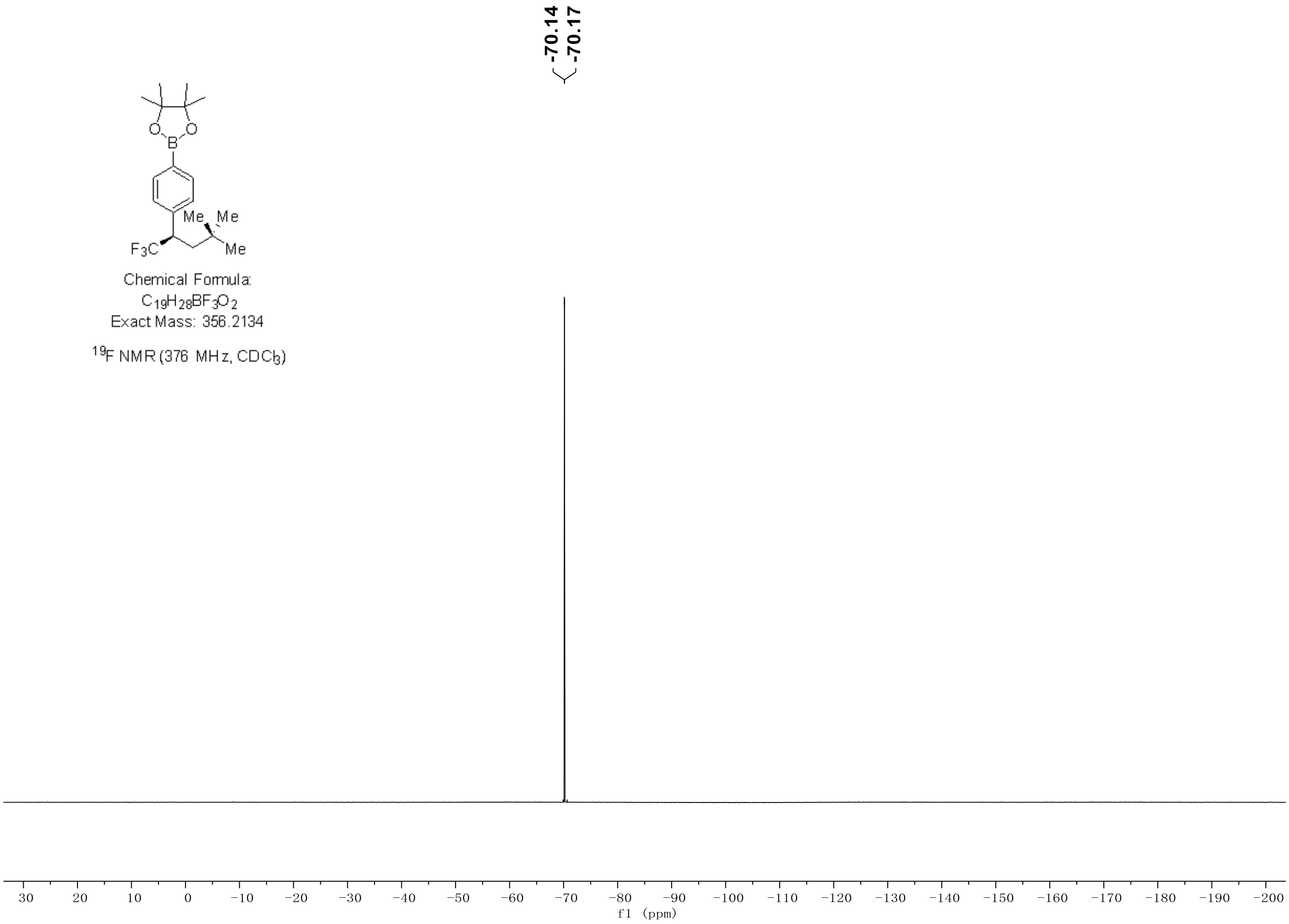

Supplementary Figure 64. ^{19}F NMR spectrum of compound 4i

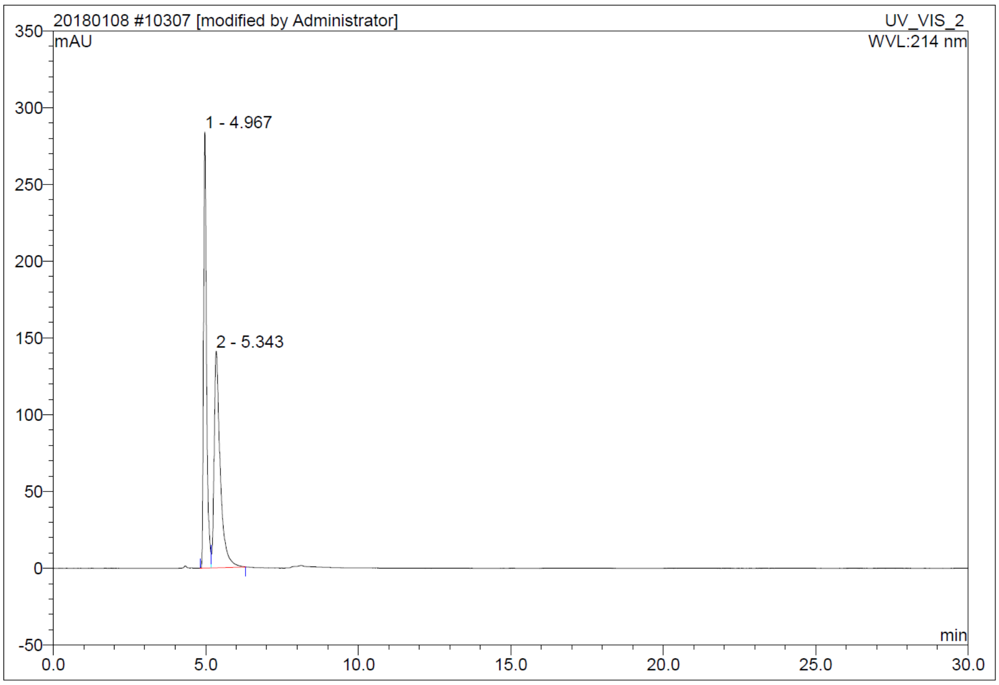

No.	Ret.Time min	Peak Name	Height mAU	Area mAU*min	Rel.Area %	Amount	Type
1	4.97	n.a.	284.161	32.097	49.59	n.a.	BM
2	5.34	n.a.	141.131	32.633	50.41	n.a.	MB
Total:			425.292	64.730	100.00	0.000	

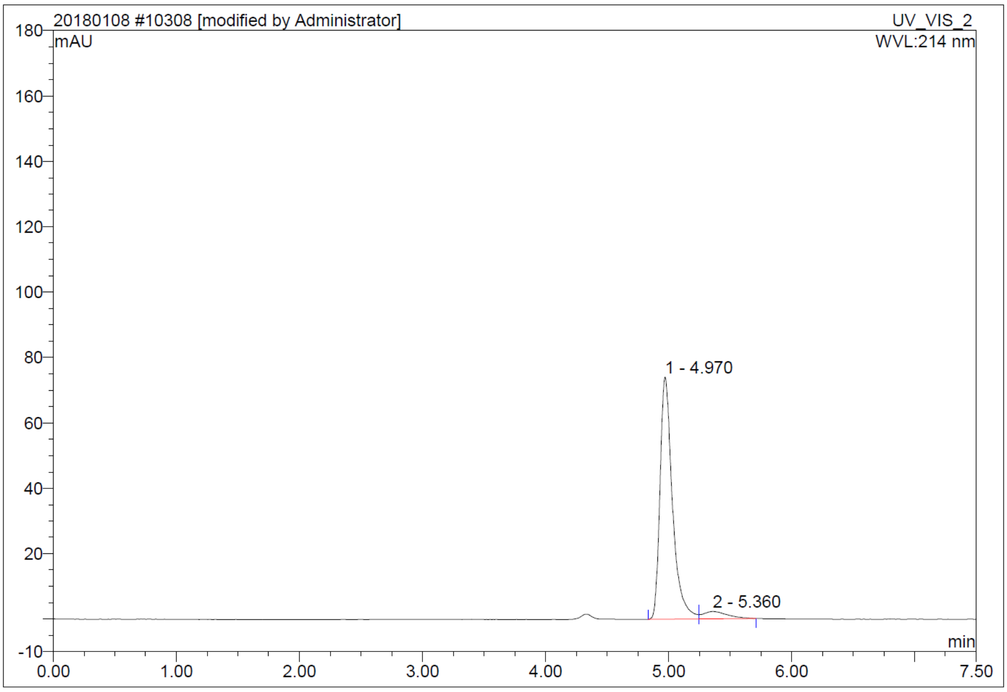

No.	Ret.Time min	Peak Name	Height mAU	Area mAU*min	Rel.Area %	Amount	Type
1	4.97	n.a.	74.047	8.667	94.40	n.a.	BM
2	5.36	n.a.	2.262	0.514	5.60	n.a.	MB
Total:			76.309	9.181	100.00	0.000	

Supplementary Figure 65. Chiral HPLC analysis of compound 4i

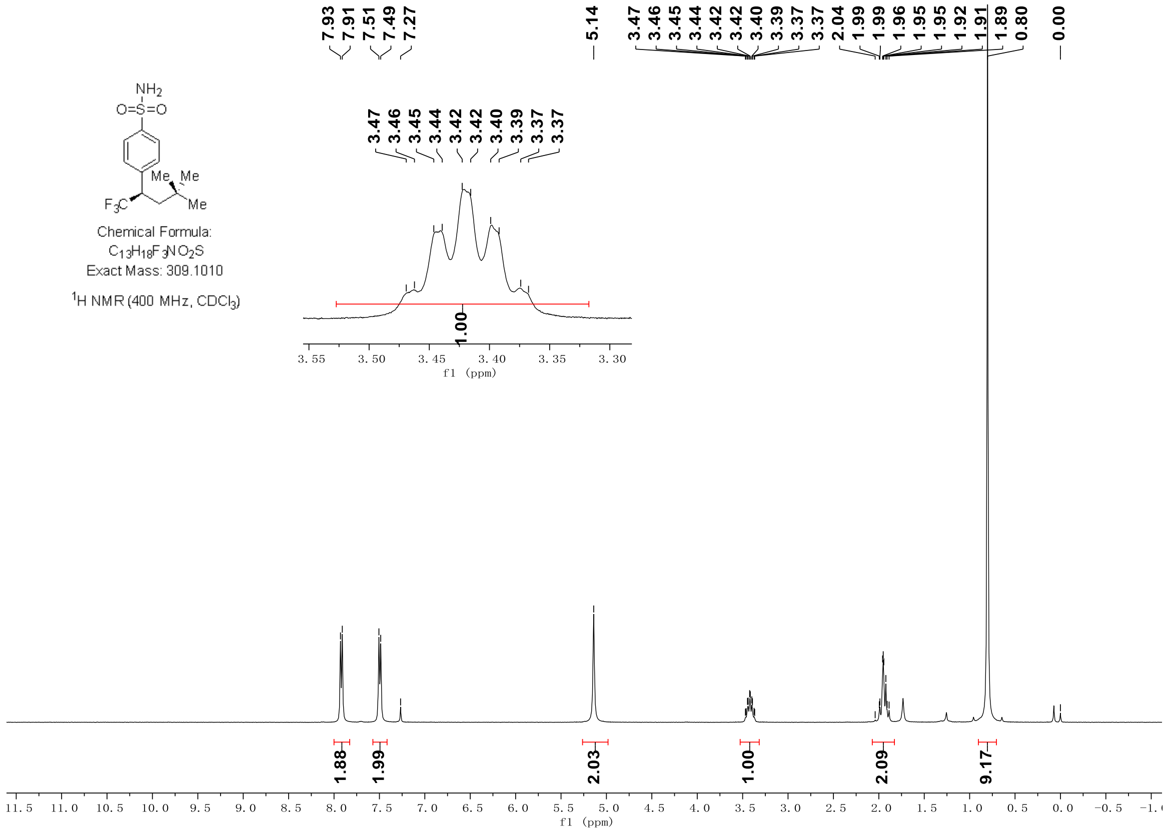

Supplementary Figure 66. ^{1}H NMR spectrum of compound 4j

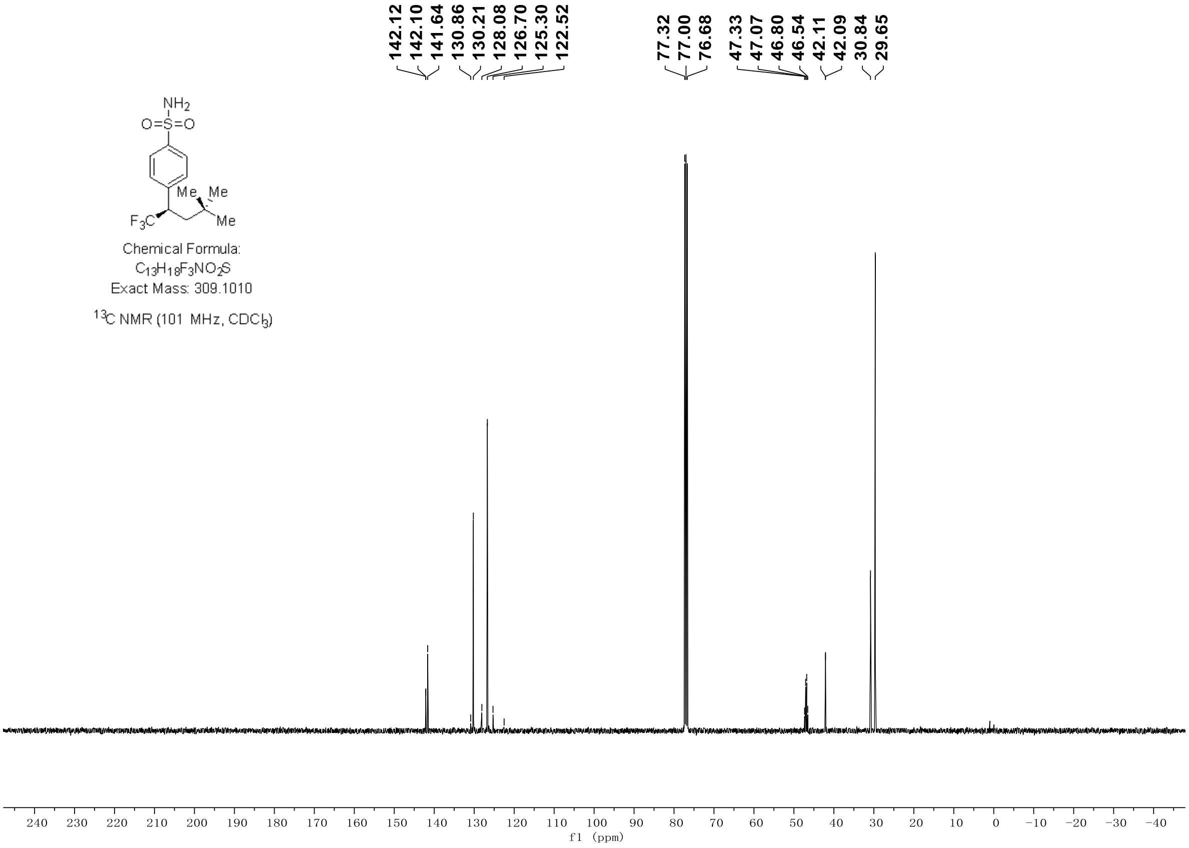

Supplementary Figure 67. ^{13}C NMR spectrum of compound 4j

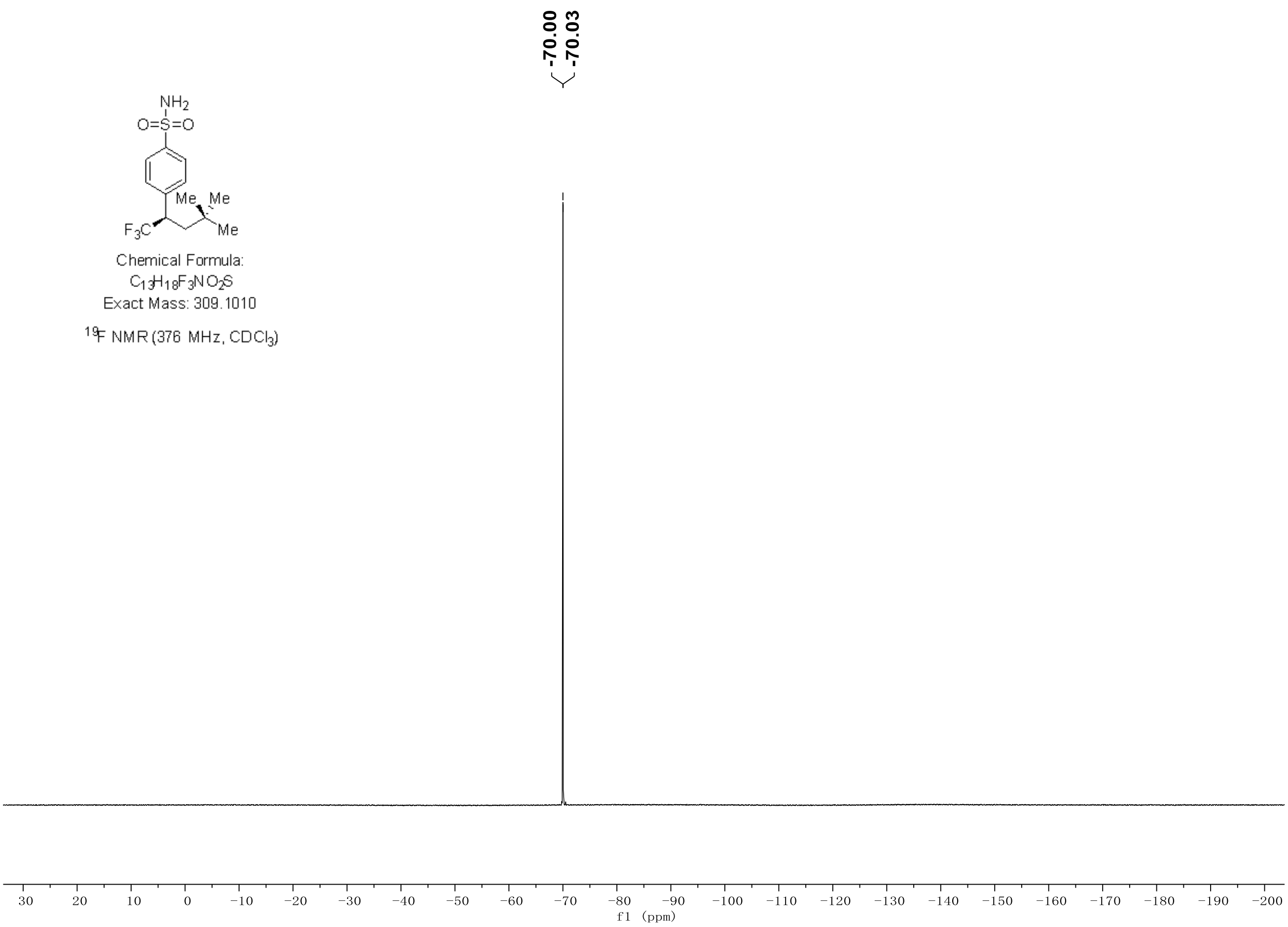

Supplementary Figure 68. ^{19}F NMR spectrum of compound 4j

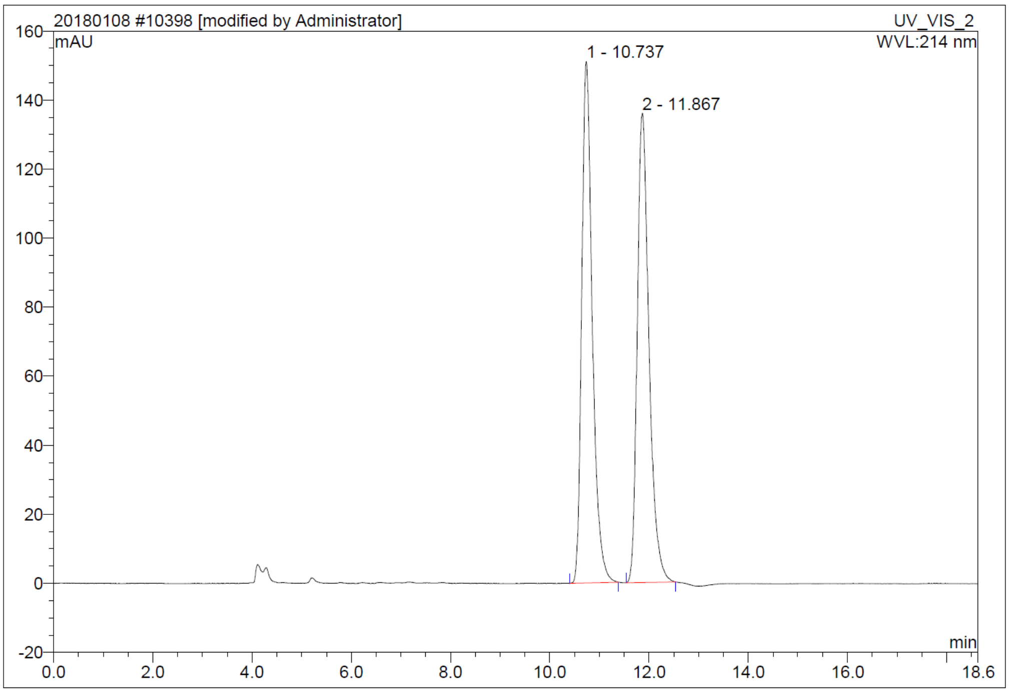

No.	Ret.Time min	Peak Name	Height mAU	Area mAU*min	Rel.Area %	Amount	Type
1	10.74	n.a.	151.094	37.347	50.07	n.a.	BMB
2	11.87	n.a.	135.996	37.244	49.93	n.a.	BMB
Total:			287.090	74.591	100.00	0.000	

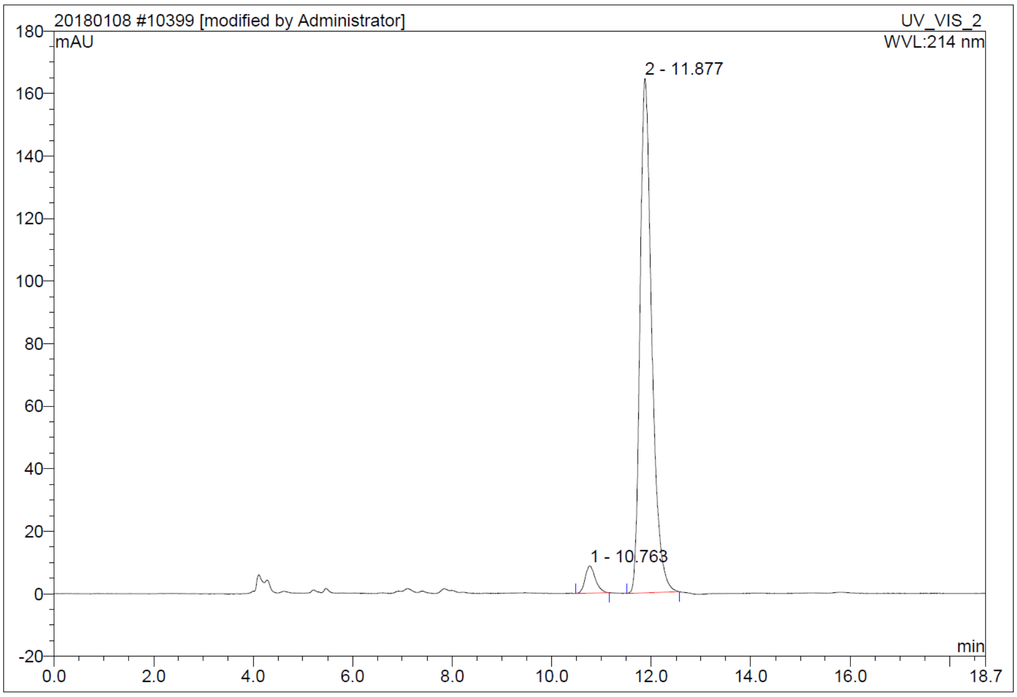

No.	Ret.Time min	Peak Name	Height mAU	Area mAU*min	Rel.Area %	Amount	Type
1	10.76	n.a.	8.697	2.079	4.41	n.a.	BMB
2	11.88	n.a.	164.545	45.105	95.59	n.a.	BMB
Total:			173.242	47.184	100.00	0.000	

Supplementary Figure 69. Chiral HPLC analysis of compound 4j

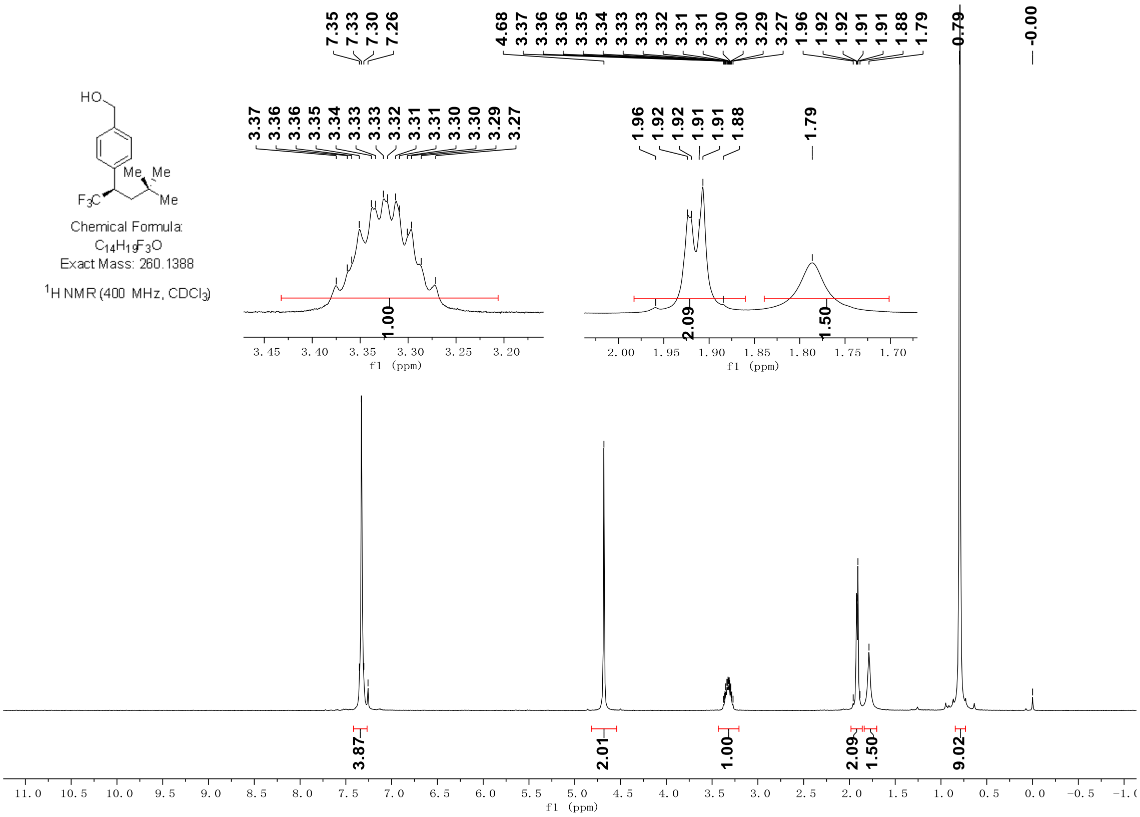

Supplementary Figure 70. ^{1}H **NMR spectrum of compound 4k**

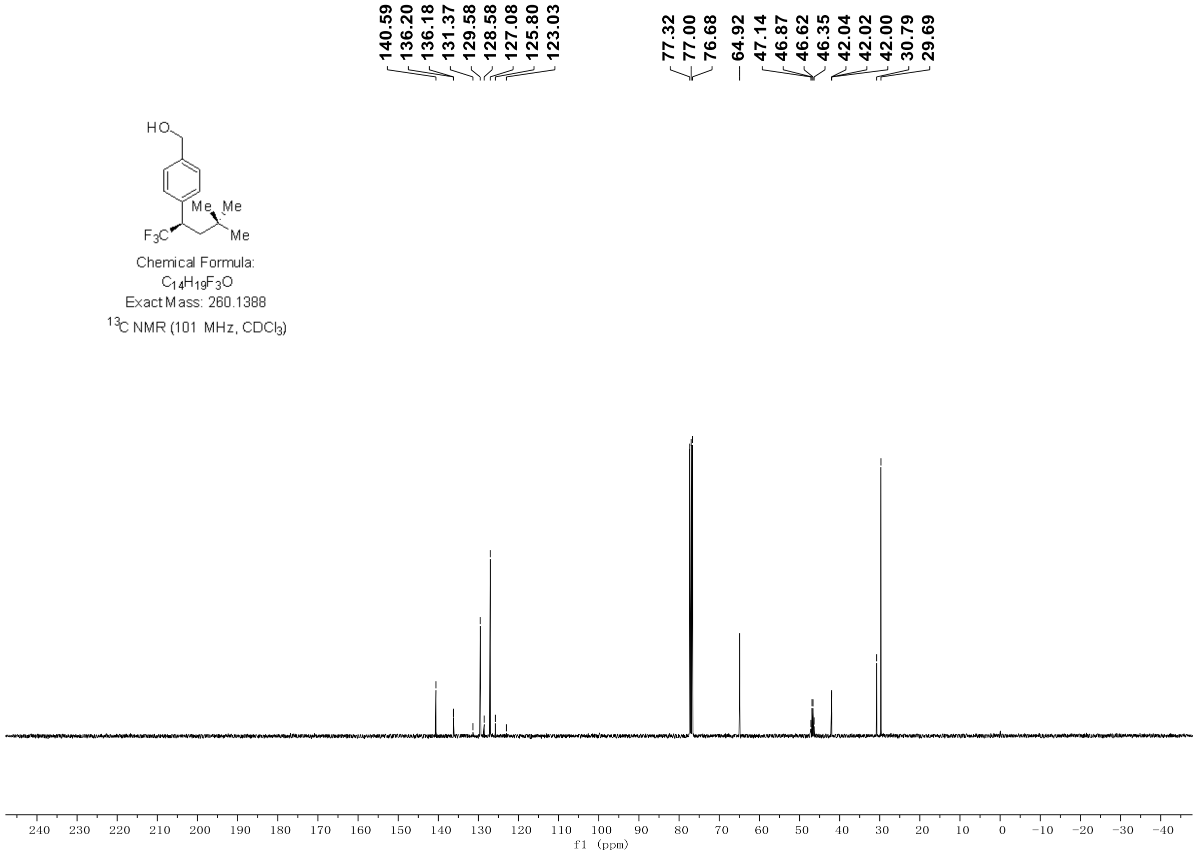

Supplementary Figure 71. ^{13}C **NMR spectrum of compound 4k**

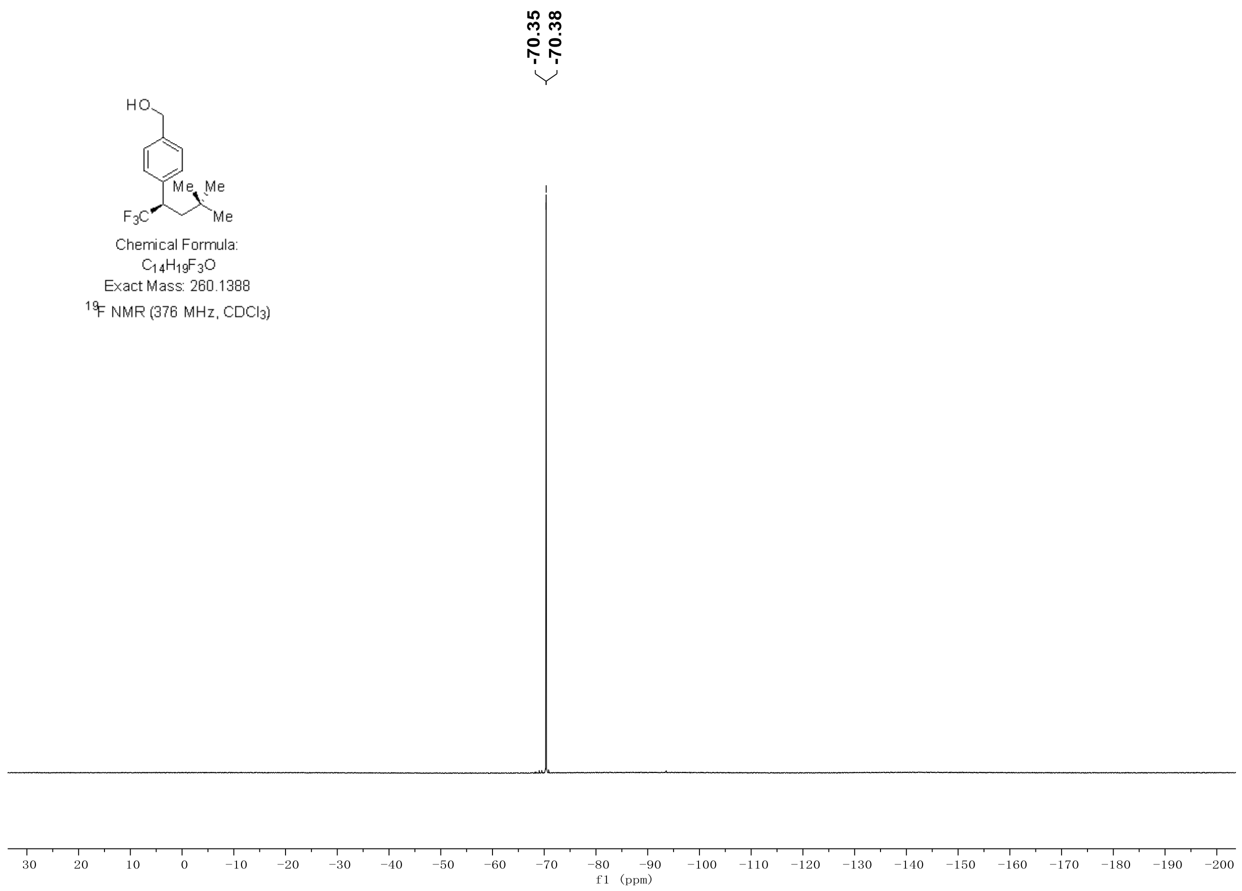

Supplementary Figure 72. ^{19}F NMR spectrum of compound 4k

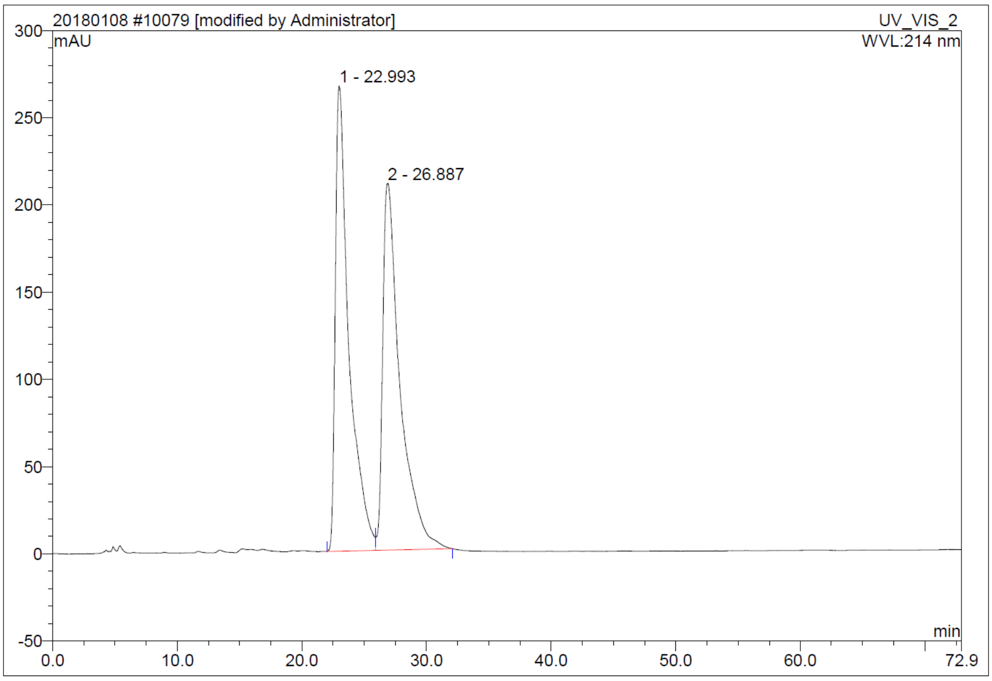

No.	Ret.Time min	Peak Name	Height mAU	Area mAU*min	Rel.Area %	Amount	Type
1	22.99	n.a.	266.692	338.539	49.92	n.a.	BM
2	26.89	n.a.	210.351	339.568	50.08	n.a.	MB
Total:			477.043	678.107	100.00	0.000	

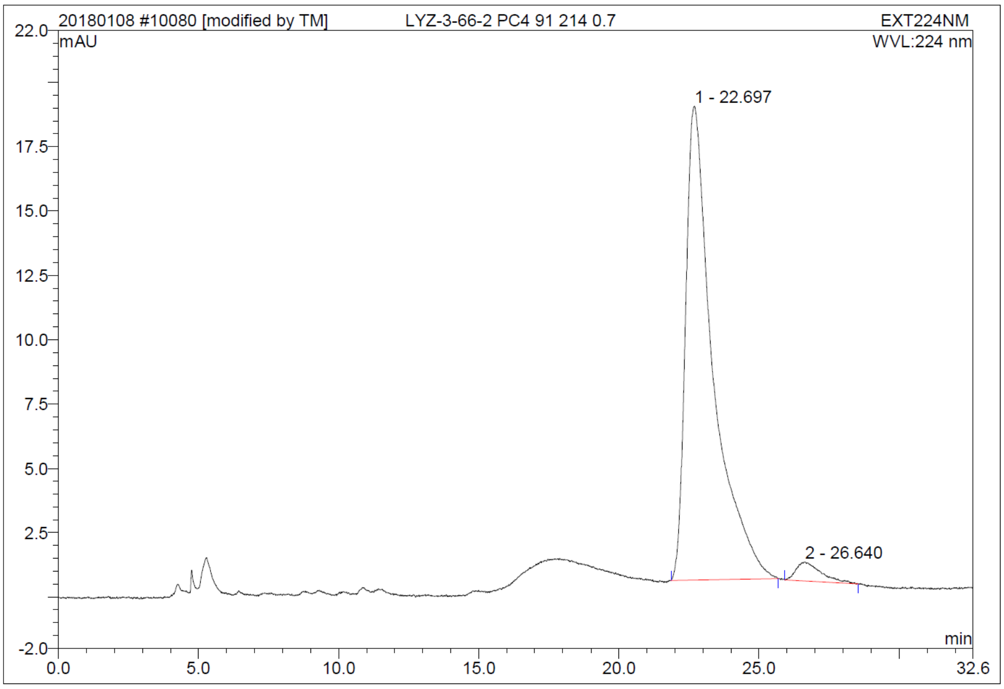

No.	Ret.Time min	Peak Name	Height mAU	Area mAU*min	Rel.Area %	Amount	Type
1	22.70	n.a.	18.417	20.914	96.50	n.a.	BMB*
2	26.64	n.a.	0.740	0.758	3.50	n.a.	BMB*
Total:			19.157	21.672	100.00	0.000	

Supplementary Figure 73. Chiral HPLC analysis of compound 4k

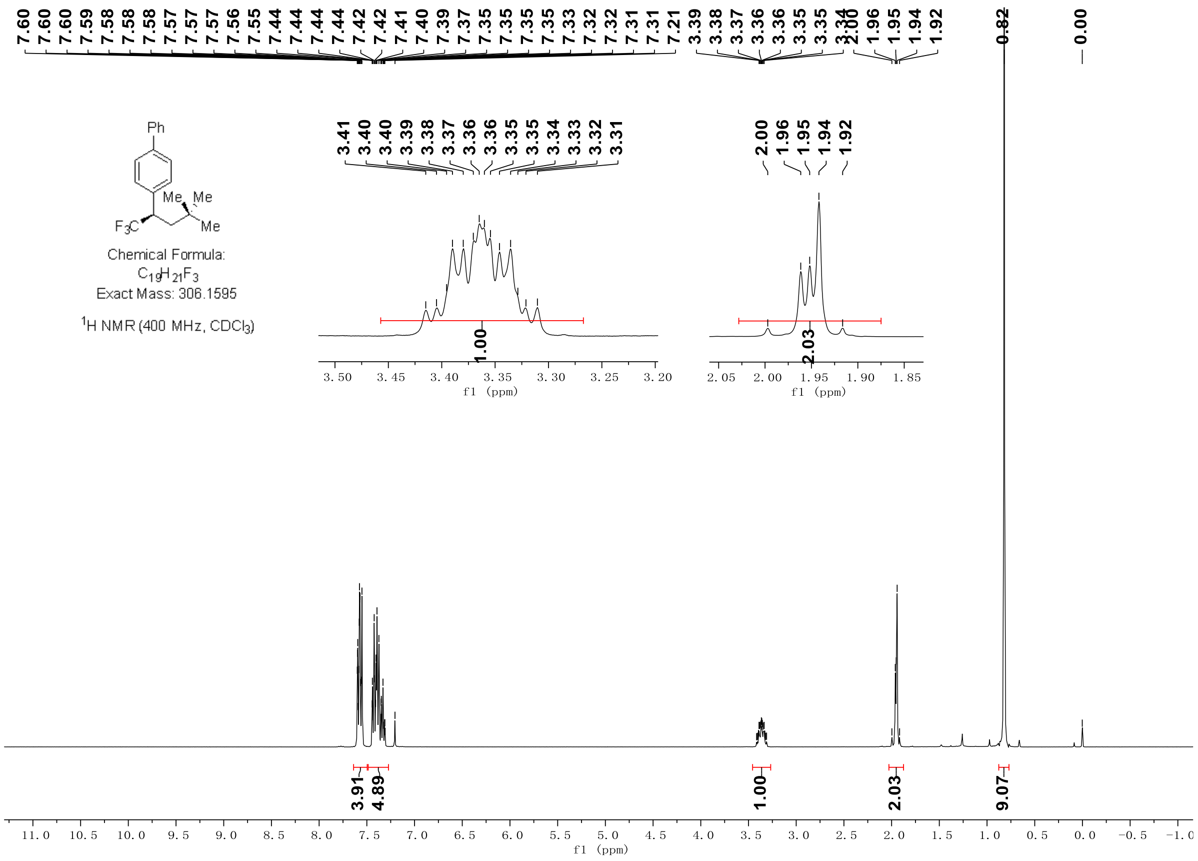

Supplementary Figure 74. ^{1}H NMR spectrum of compound 4l

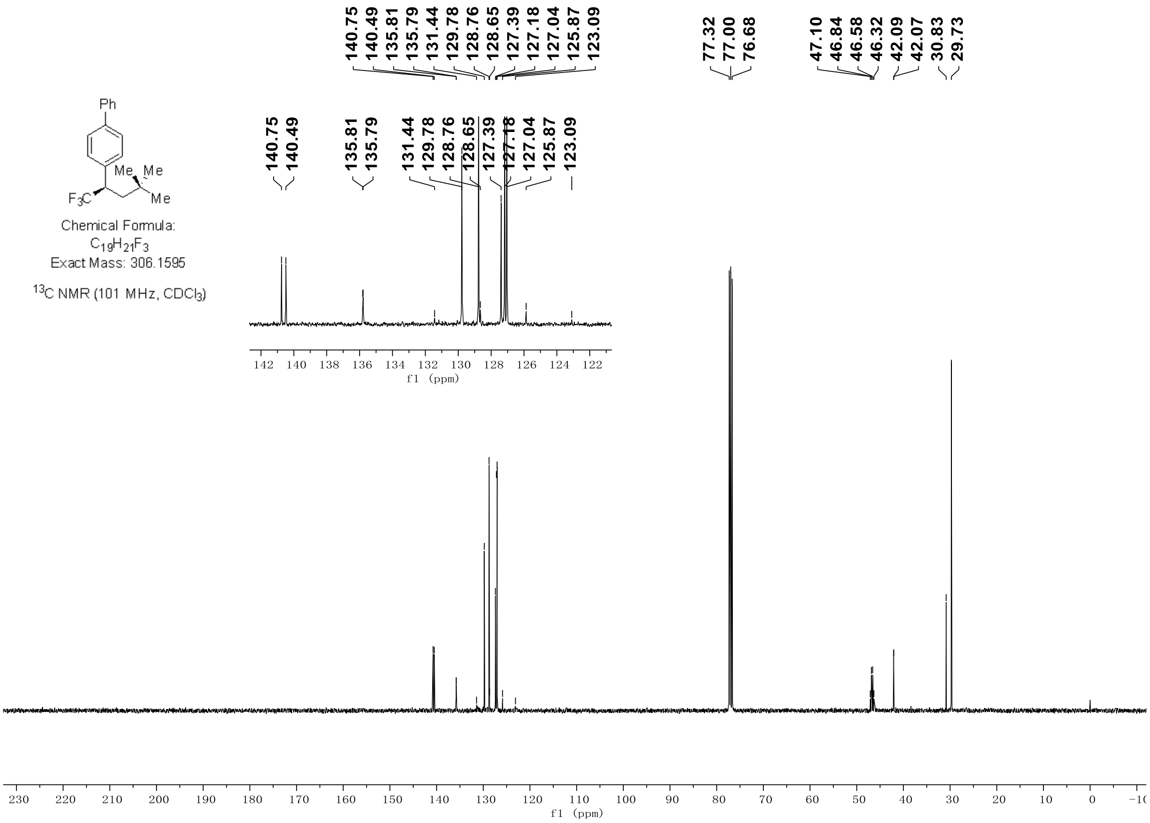

Supplementary Figure 75. ^{19}F NMR spectrum of compound 4l

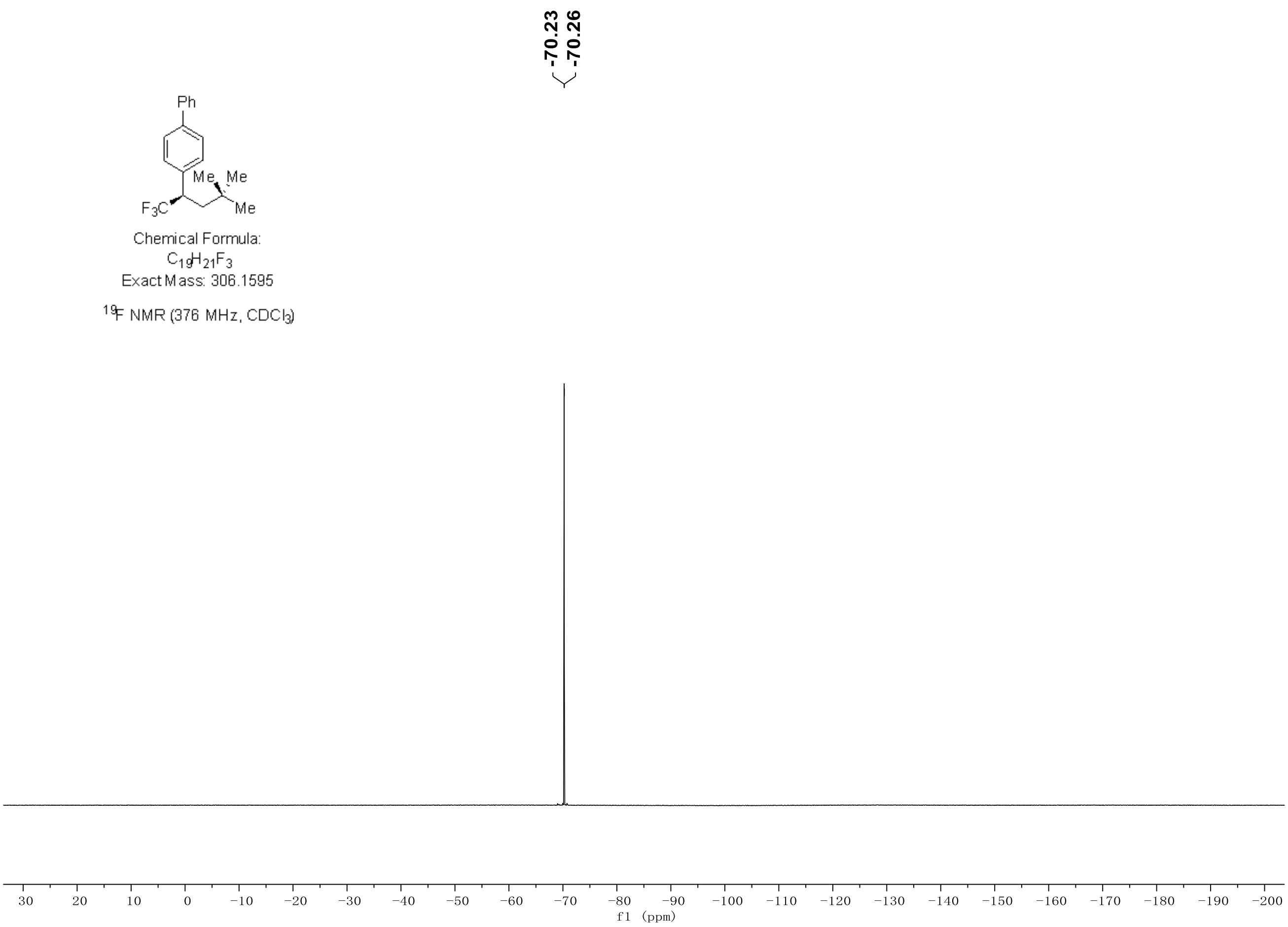

Supplementary Figure 76. ^{19}F NMR spectrum of compound 4l

Chromatogram

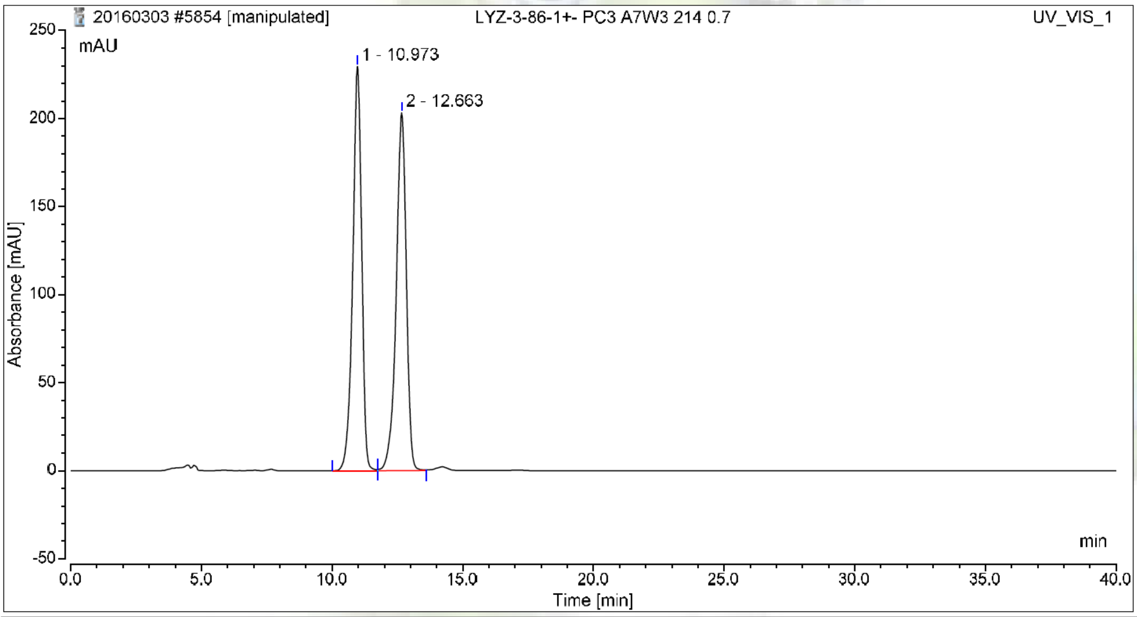

Integration Results

No.	Retention Time<br>min	Area<br>mAU*min	Height<br>mAU	Relative Area<br>%	Resolution (EP)	Asymmetry (EP)	K'	Plates (EP)
1	10.973	90.9648	229.5956	49.897	2.63	0.89	n.a.	5243
2	12.663	91.3389	203.1842	50.103	n.a.	0.88	n.a.	5508
**Total:**		**182.304**	**1401.998**	**100.000**				

Chromatogram

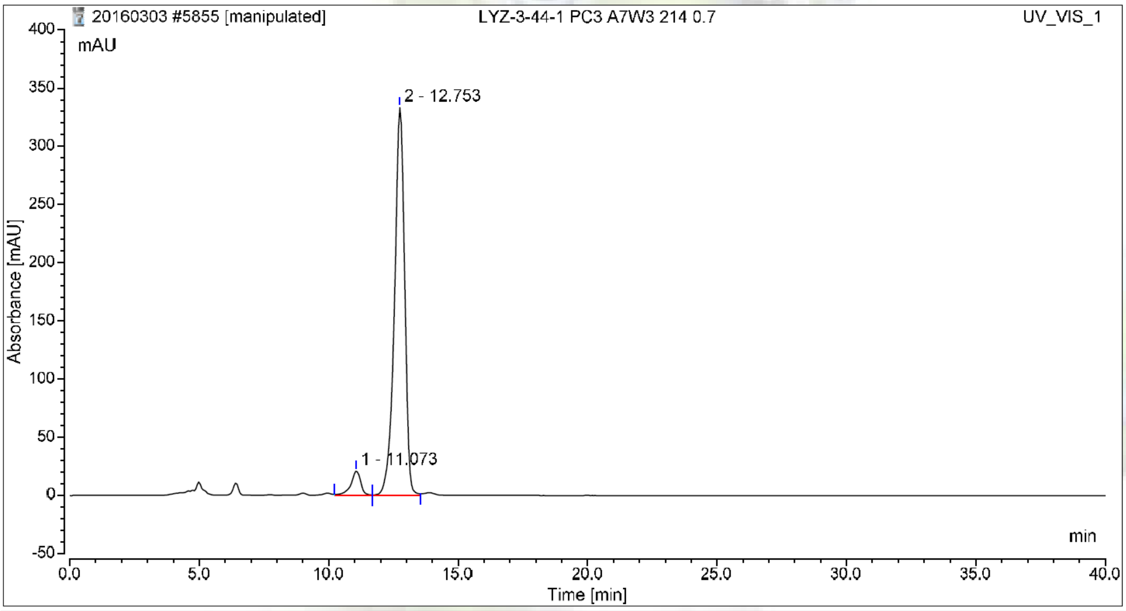

Integration Results

No.	Retention Time<br>min	Area<br>mAU*min	Height<br>mAU	Relative Area<br>%	Resolution (EP)	Asymmetry (EP)	K'	Plates (EP)
1	11.073	9.3609	20.8859	5.768	2.53	0.79	n.a.	4892
2	12.753	152.9219	333.3006	94.232	n.a.	0.86	n.a.	5345
**Total:**		**162.283**	**1401.998**	**100.000**				

Supplementary Figure 77. Chiral HPLC analysis of compound 4l

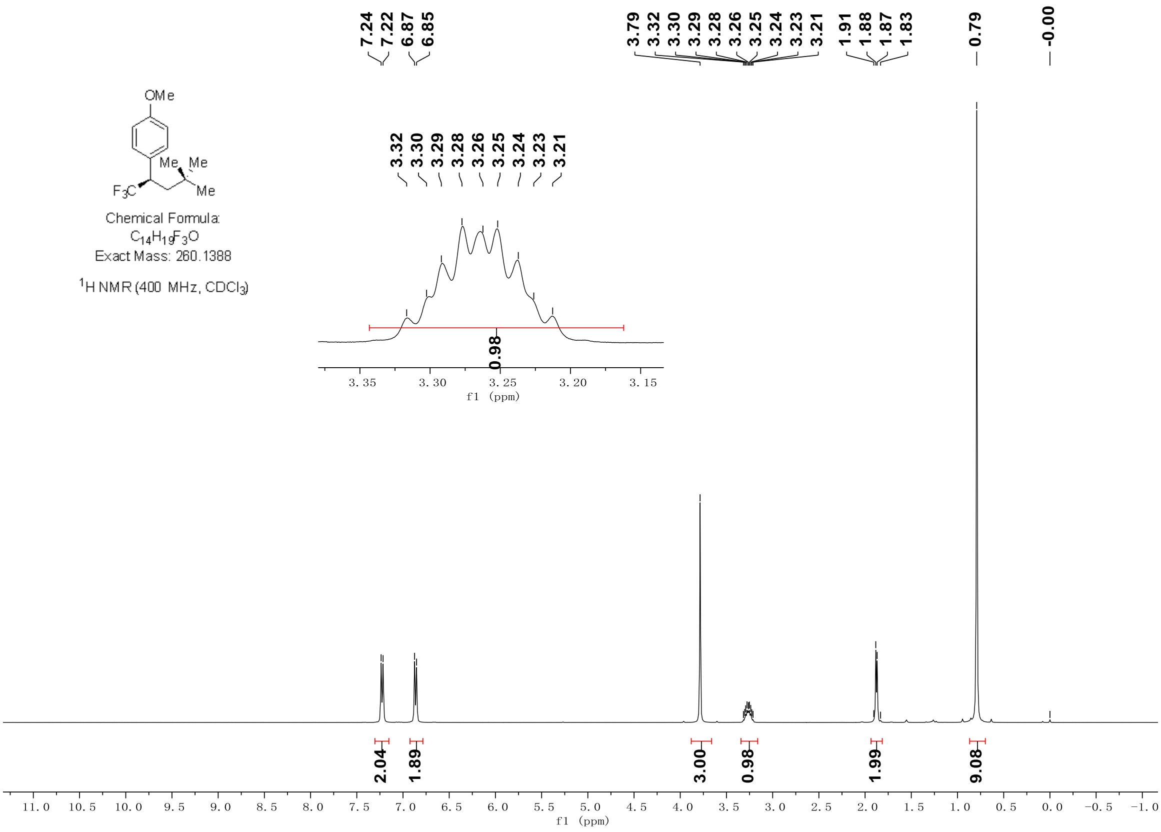

Supplementary Figure 78. ^{1}H NMR spectrum of compound 4m

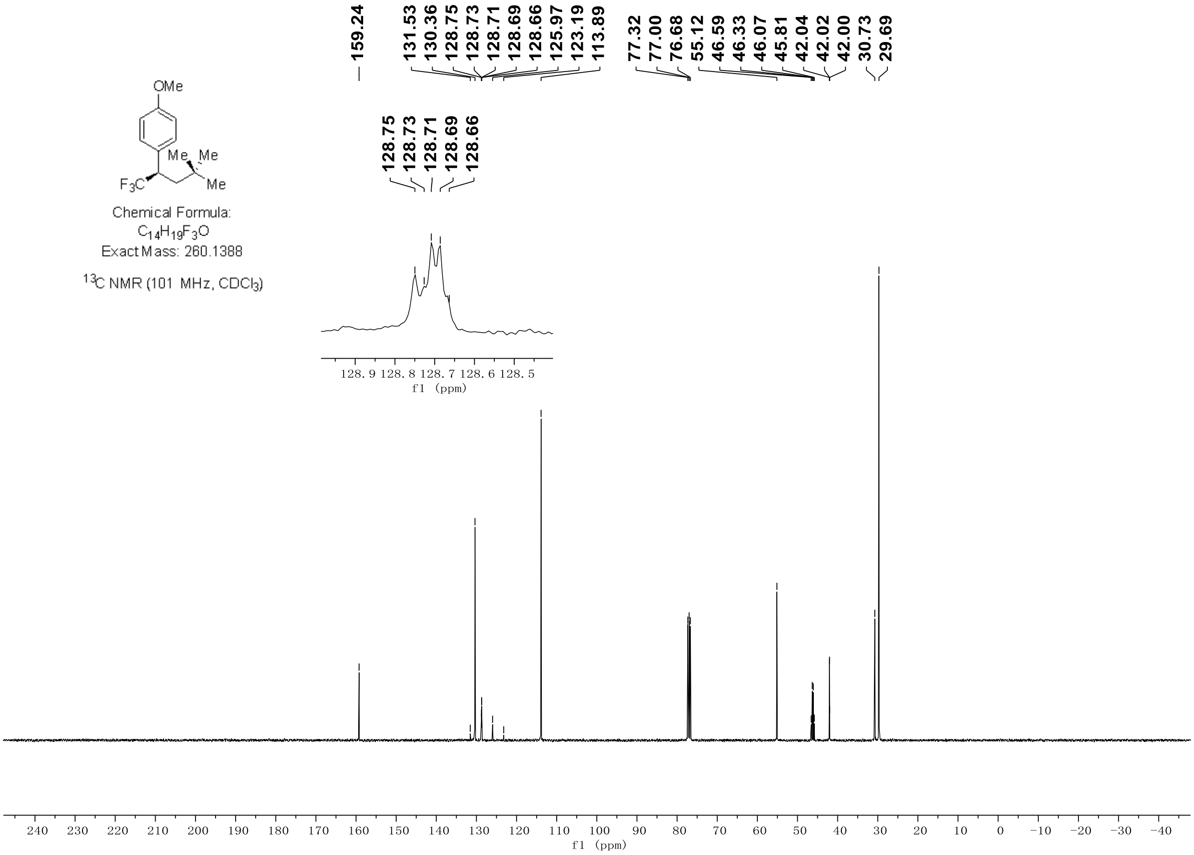

Supplementary Figure 79. ^{13}C NMR spectrum of compound 4m

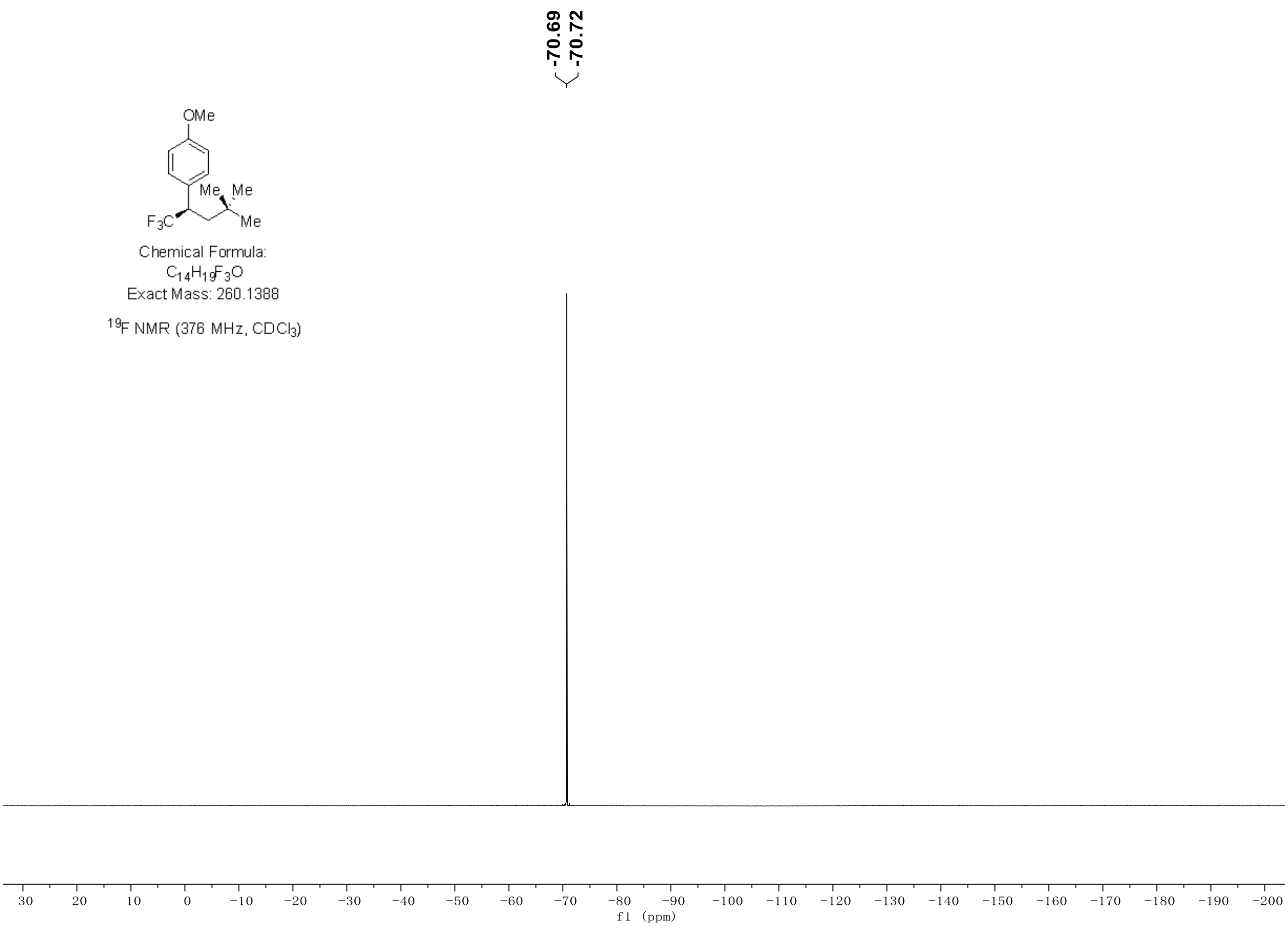

Supplementary Figure 80. ^{19}F NMR spectrum of compound 4m

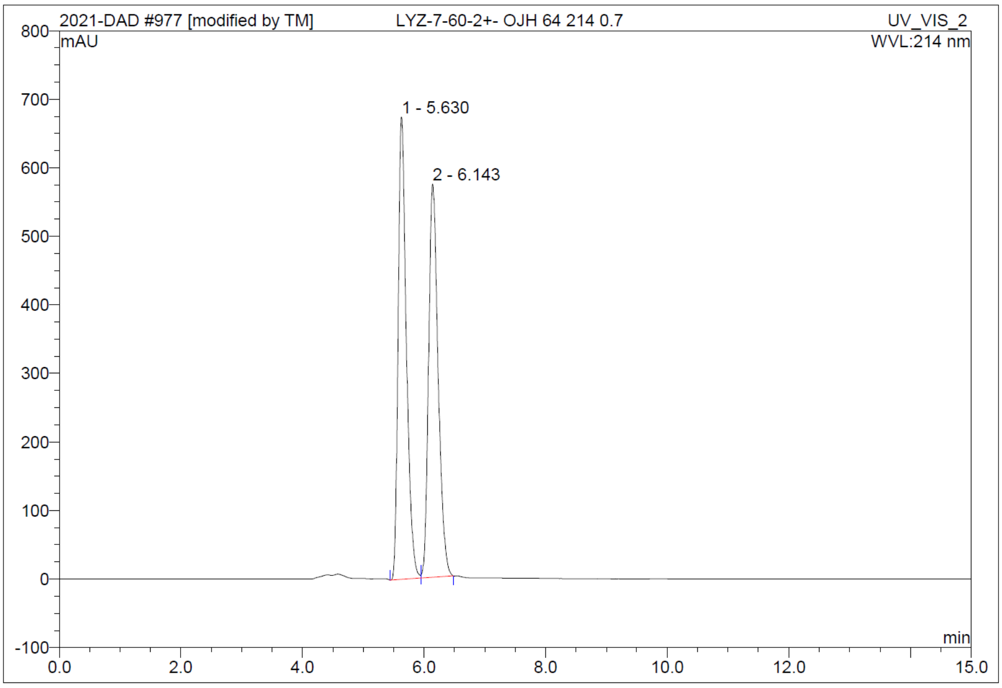

No.	Ret.Time min	Peak Name	Height mAU	Area mAU*min	Rel.Area %	Amount	Type
1	5.63	n.a.	674.642	105.500	50.36	n.a.	BM *
2	6.14	n.a.	573.849	103.982	49.64	n.a.	MB*
Total:			1248.491	209.482	100.00	0.000	

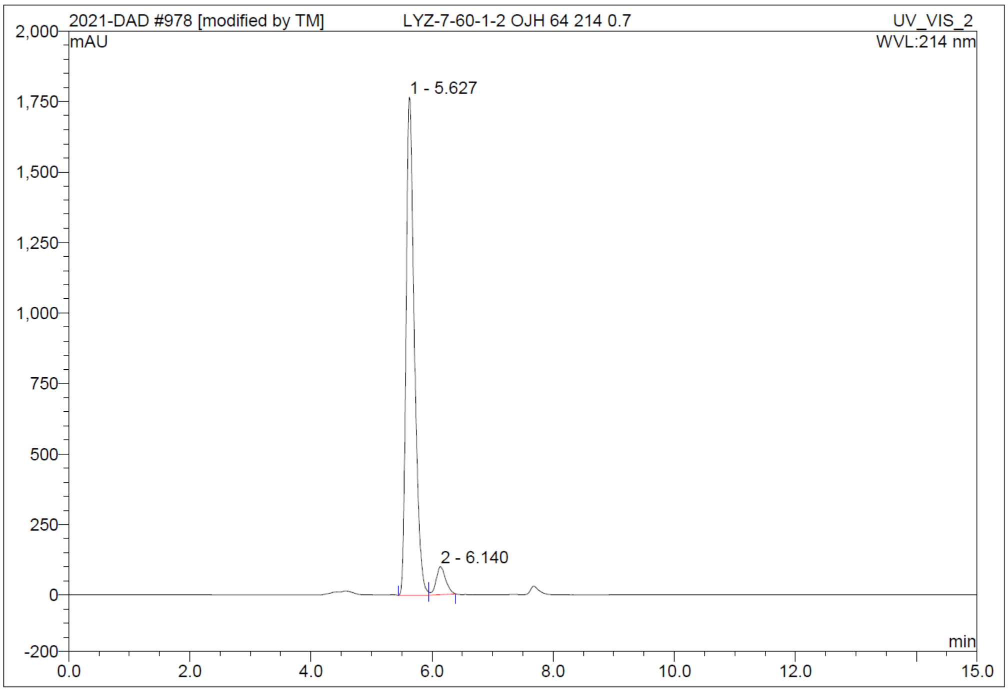

No.	Ret.Time min	Peak Name	Height mAU	Area mAU*min	Rel.Area %	Amount	Type
1	5.63	n.a.	1766.439	281.272	93.91	n.a.	BM *
2	6.14	n.a.	99.054	18.250	6.09	n.a.	MB*
Total:			1865.493	299.522	100.00	0.000	

Supplementary Figure 81. Chiral HPLC analysis of compound 4m

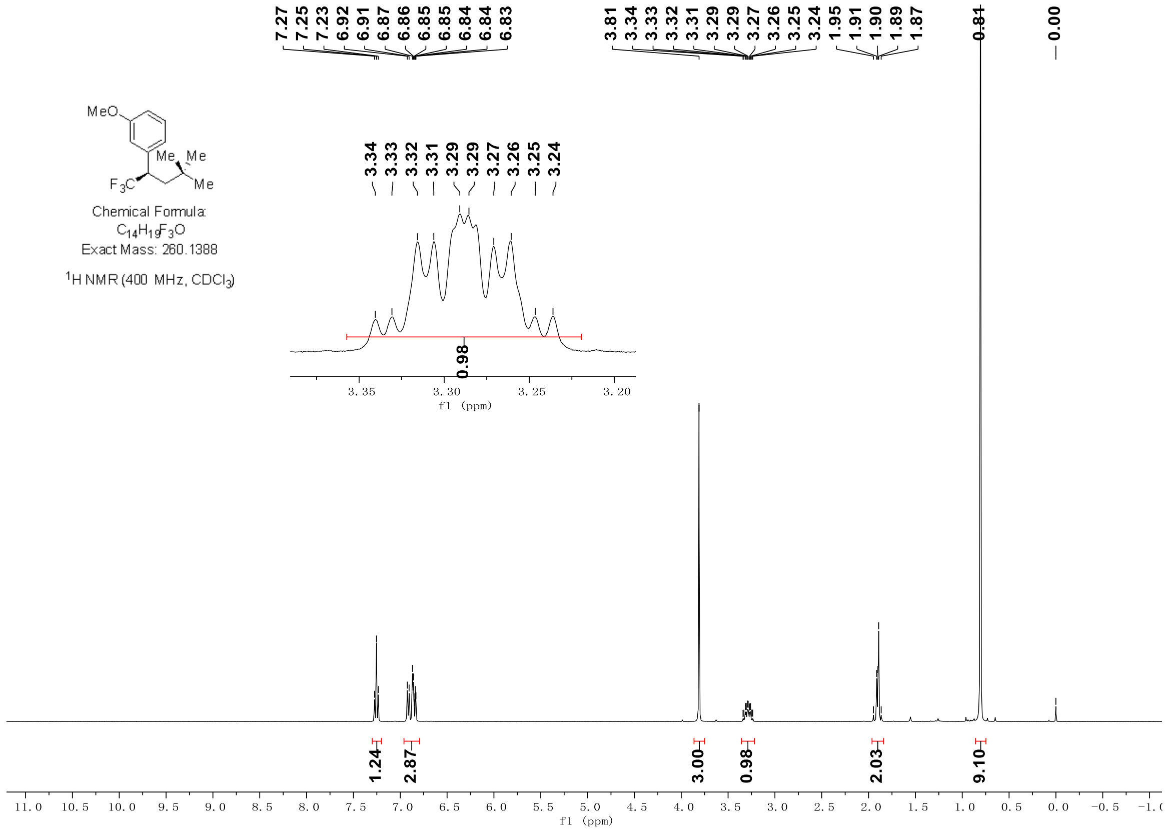

Supplementary Figure 82. ^{1}H NMR spectrum of compound 4n

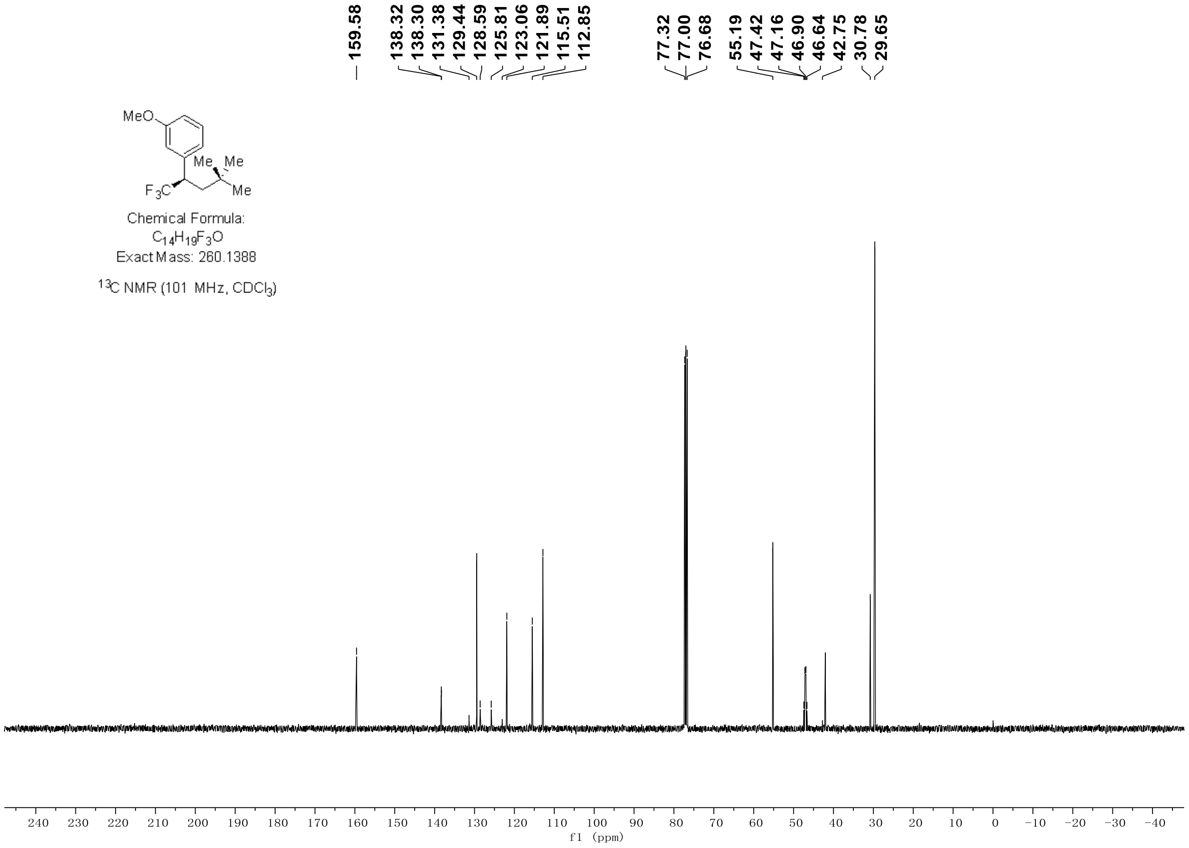

Supplementary Figure 83. ^{13}C NMR spectrum of compound 4n

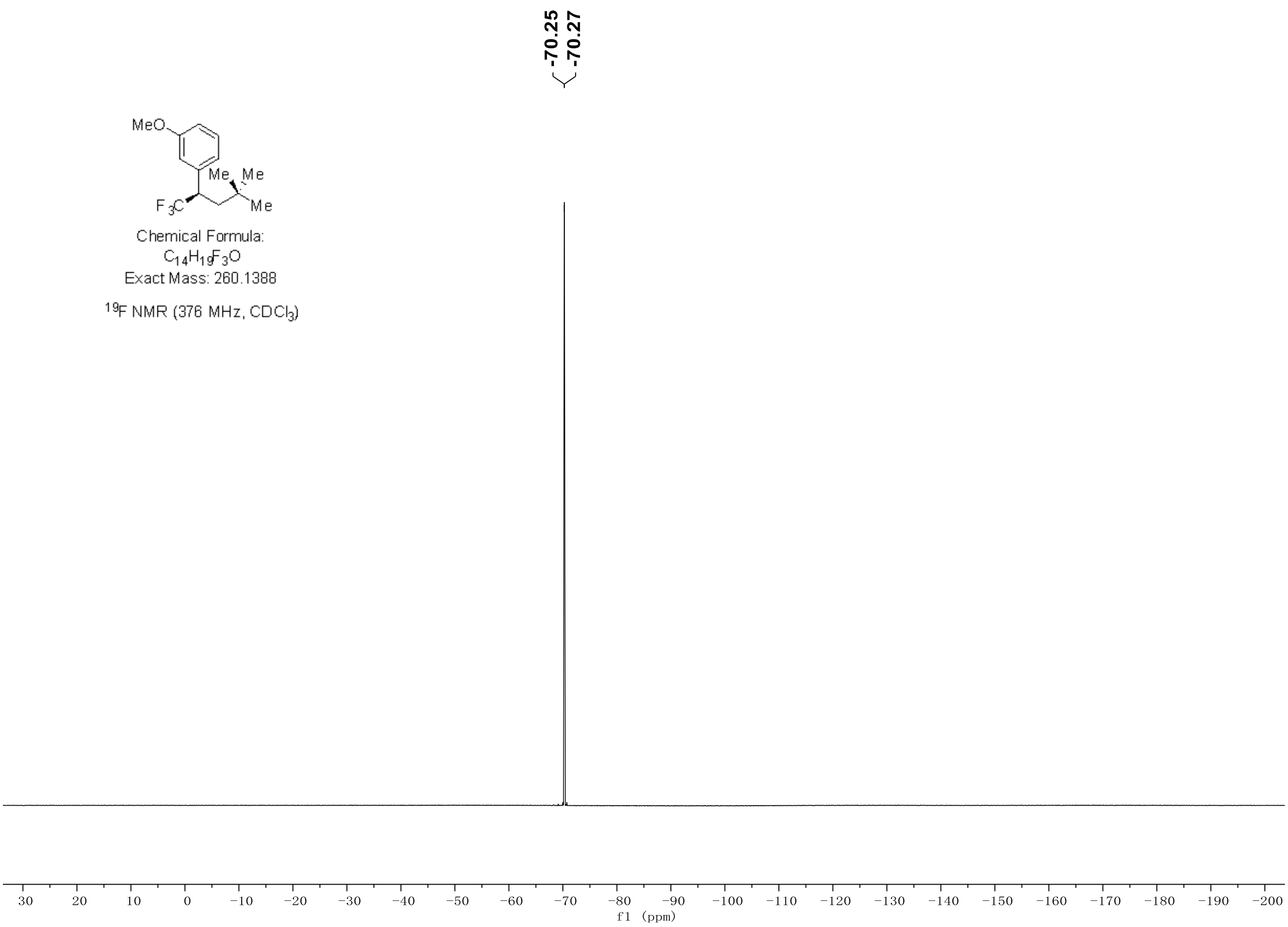

Supplementary Figure 84. ^{19}F NMR spectrum of compound 4n

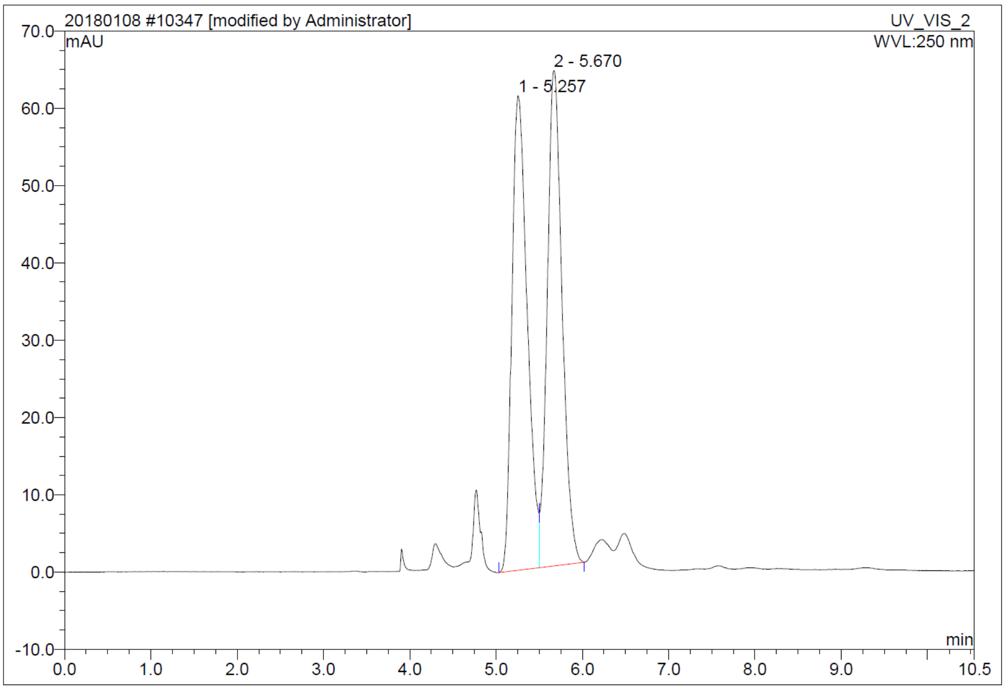

No.	Ret.Time min	Peak Name	Height mAU	Area mAU*min	Rel.Area %	Amount	Type
1	5.26	n.a.	61.378	12.590	49.50	n.a.	BM *
2	5.67	n.a.	64.055	12.845	50.50	n.a.	MB*
Total:			125.433	25.435	100.00	0.000	

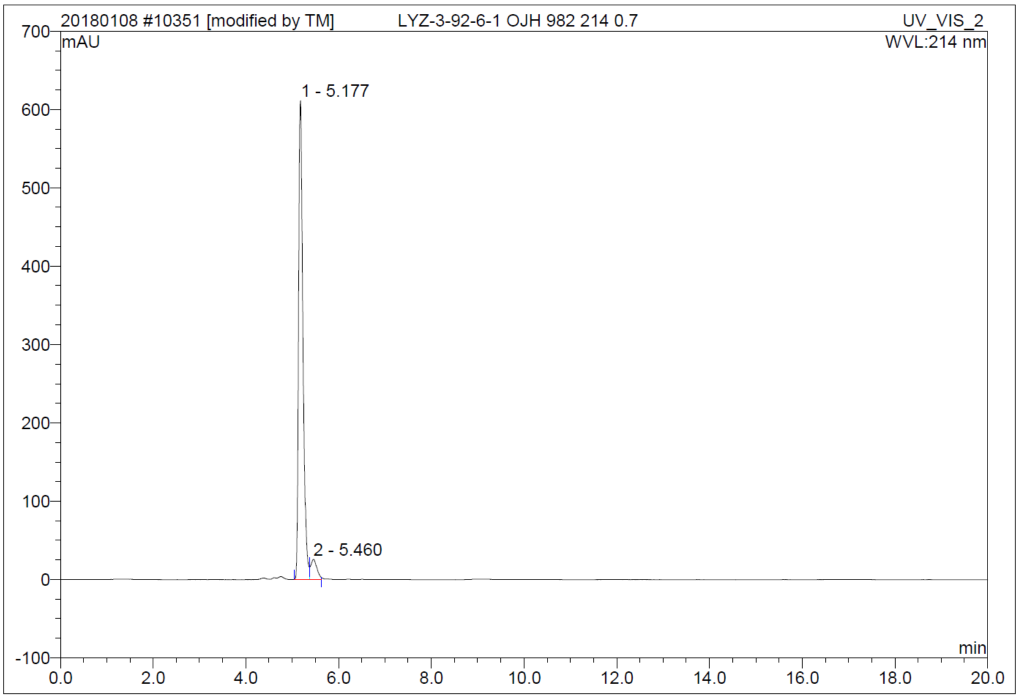

No.	Ret.Time min	Peak Name	Height mAU	Area mAU*min	Rel.Area %	Amount	Type
1	5.18	n.a.	611.276	66.100	94.27	n.a.	BM *
2	5.46	n.a.	25.766	4.014	5.73	n.a.	M *
Total:			637.041	70.114	100.00	0.000	

Supplementary Figure 85. Chiral HPLC analysis of compound 4n

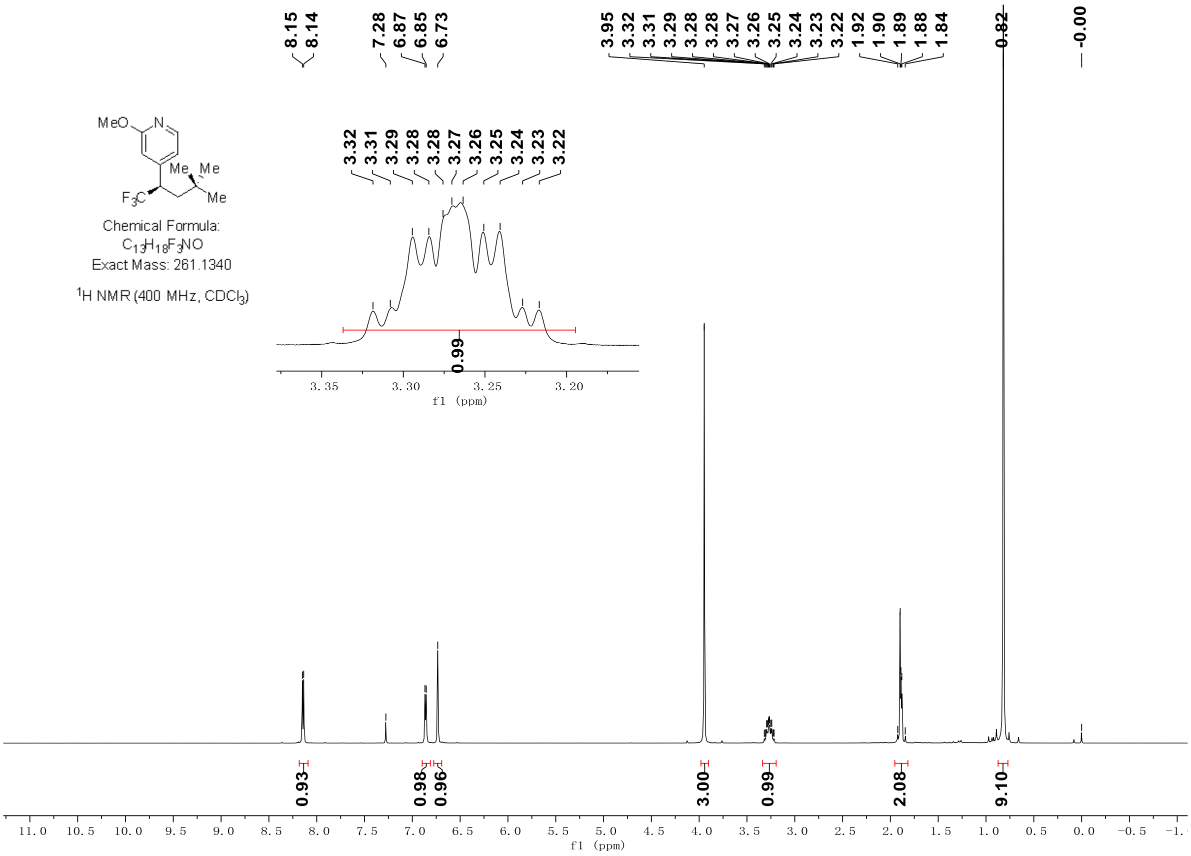

Supplementary Figure 86. ^{1}**H NMR spectrum of compound 4o**

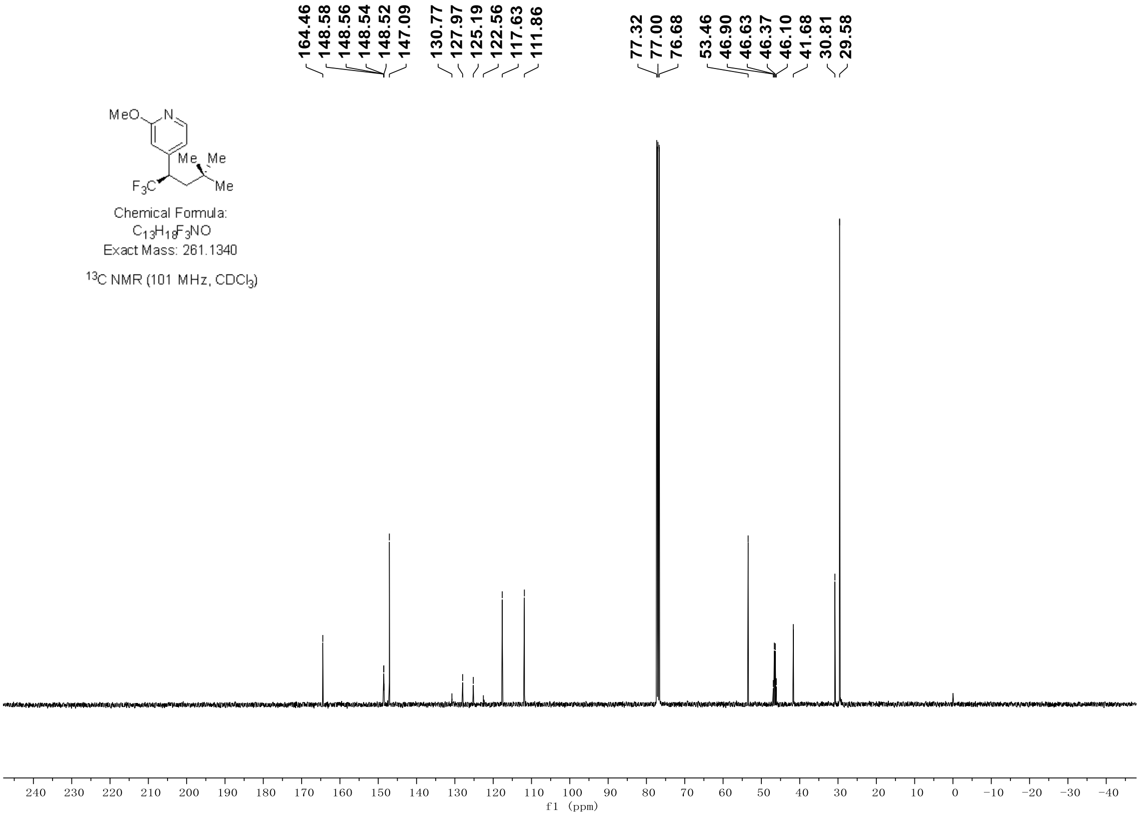

Supplementary Figure 87. ^{13}**C NMR spectrum of compound 4o**

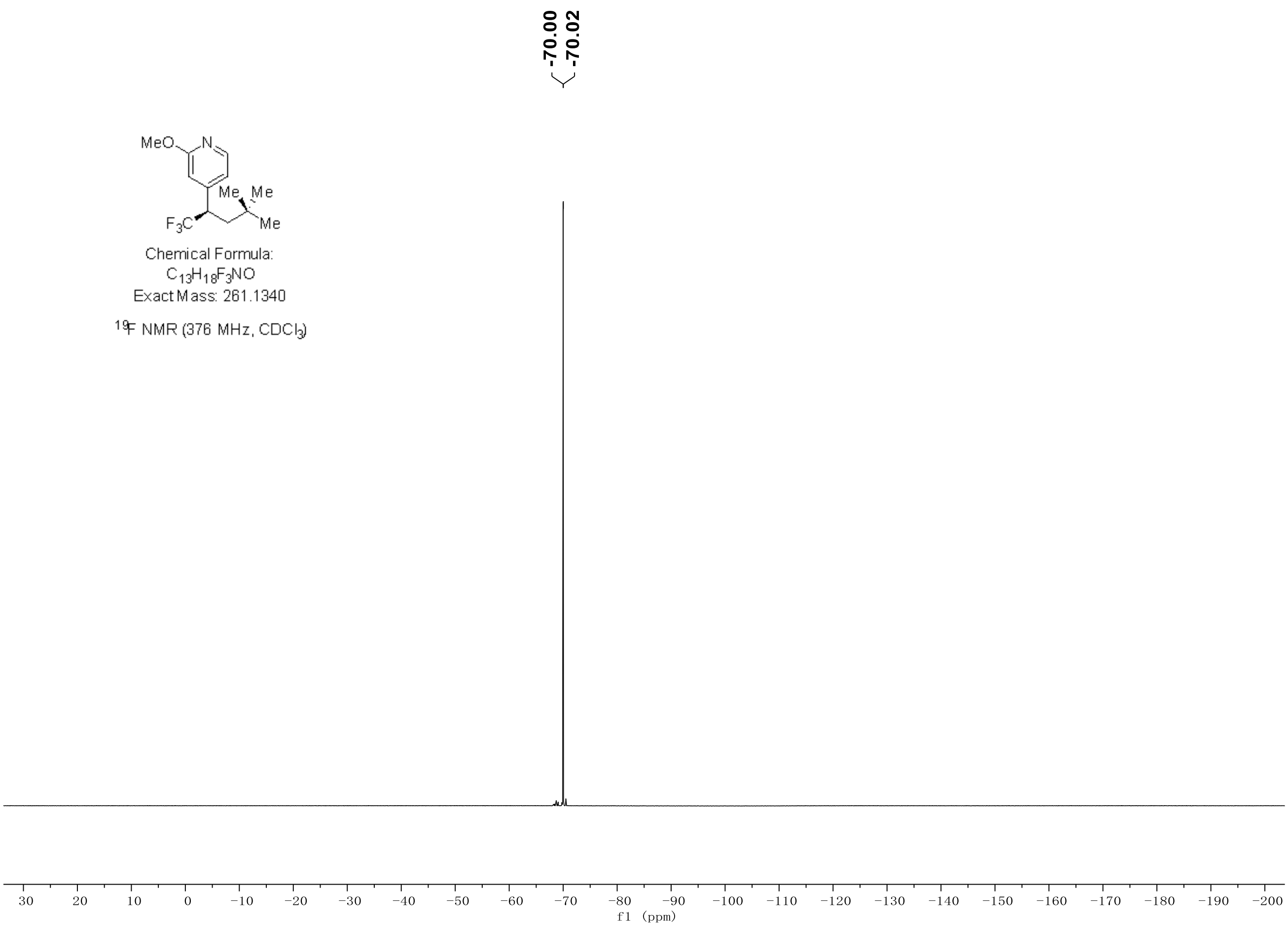

Supplementary Figure 88. ^{19}F NMR spectrum of compound 4o

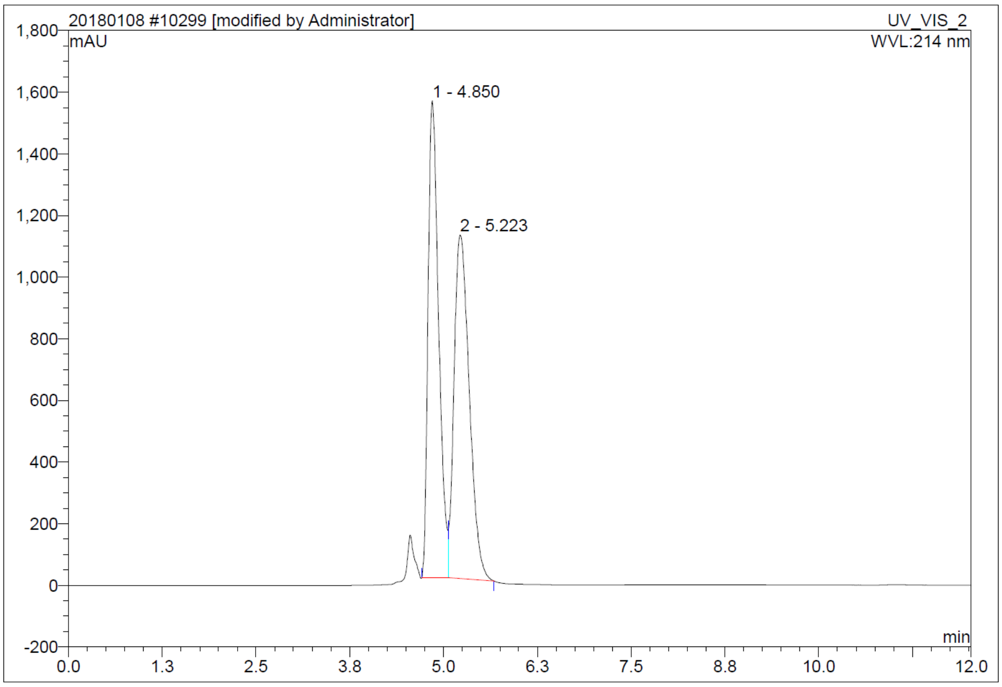

No.	Ret.Time min	Peak Name	Height mAU	Area mAU*min	Rel.Area %	Amount	Type
1	4.85	n.a.	1548.056	252.800	49.01	n.a.	BM *
2	5.22	n.a.	1115.188	263.054	50.99	n.a.	MB*
Total:			2663.243	515.854	100.00	0.000	

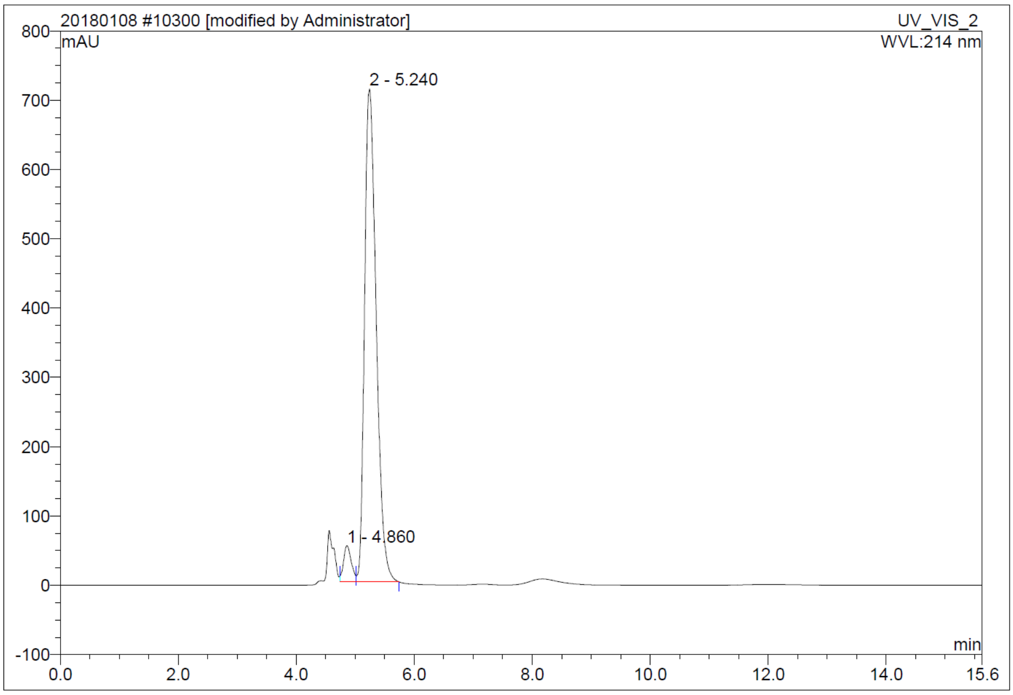

No.	Ret.Time min	Peak Name	Height mAU	Area mAU*min	Rel.Area %	Amount	Type
1	4.86	n.a.	51.999	8.010	4.70	n.a.	M *
2	5.24	n.a.	711.538	162.457	95.30	n.a.	MB*
Total:			763.537	170.467	100.00	0.000	

Supplementary Figure 89. Chiral HPLC analysis of compound 4o

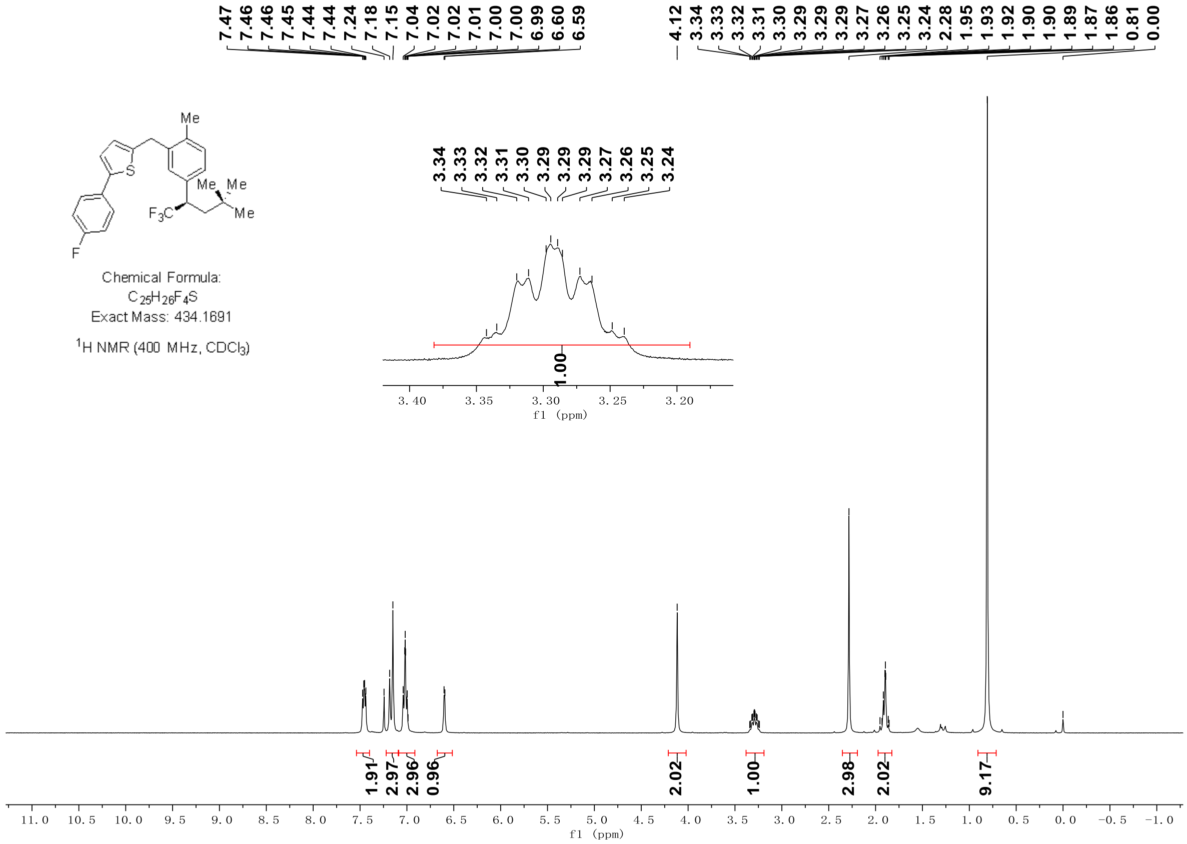

Supplementary Figure 90. ^{1}H NMR spectrum of compound 4p

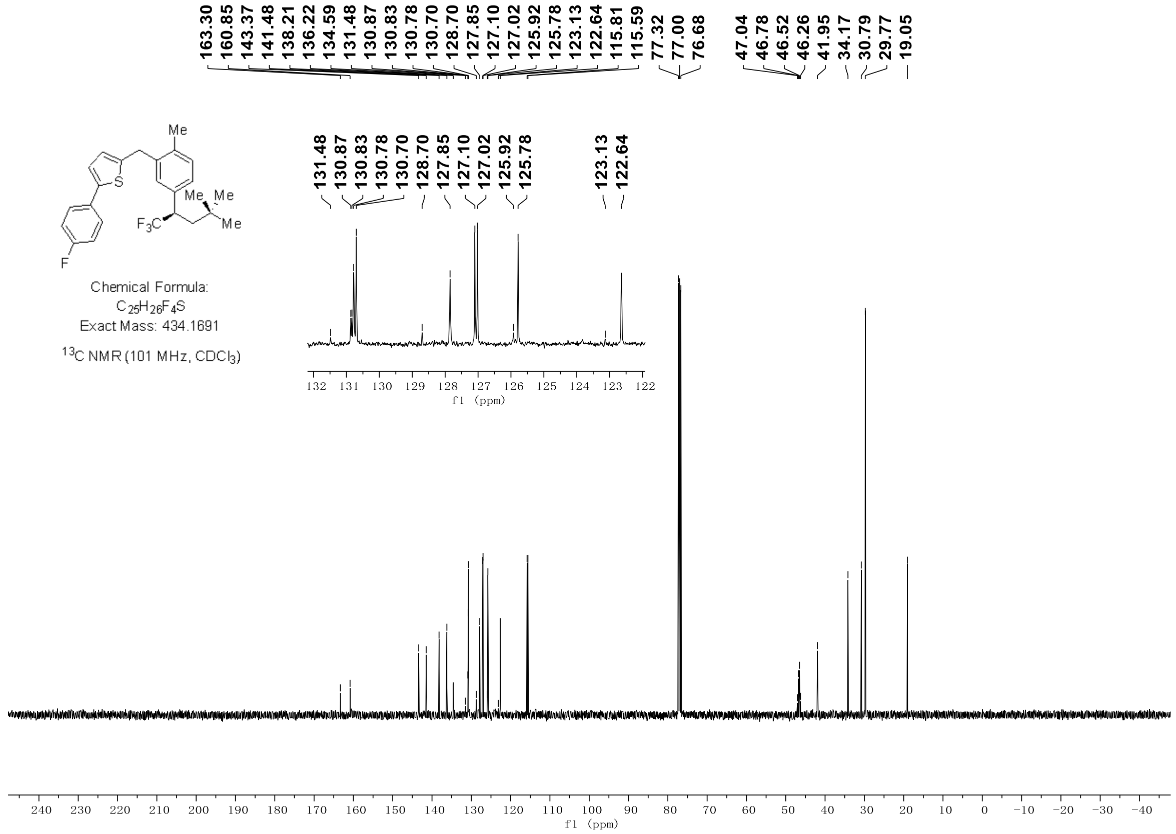

Supplementary Figure 91. ^{13}C NMR spectrum of compound 4p

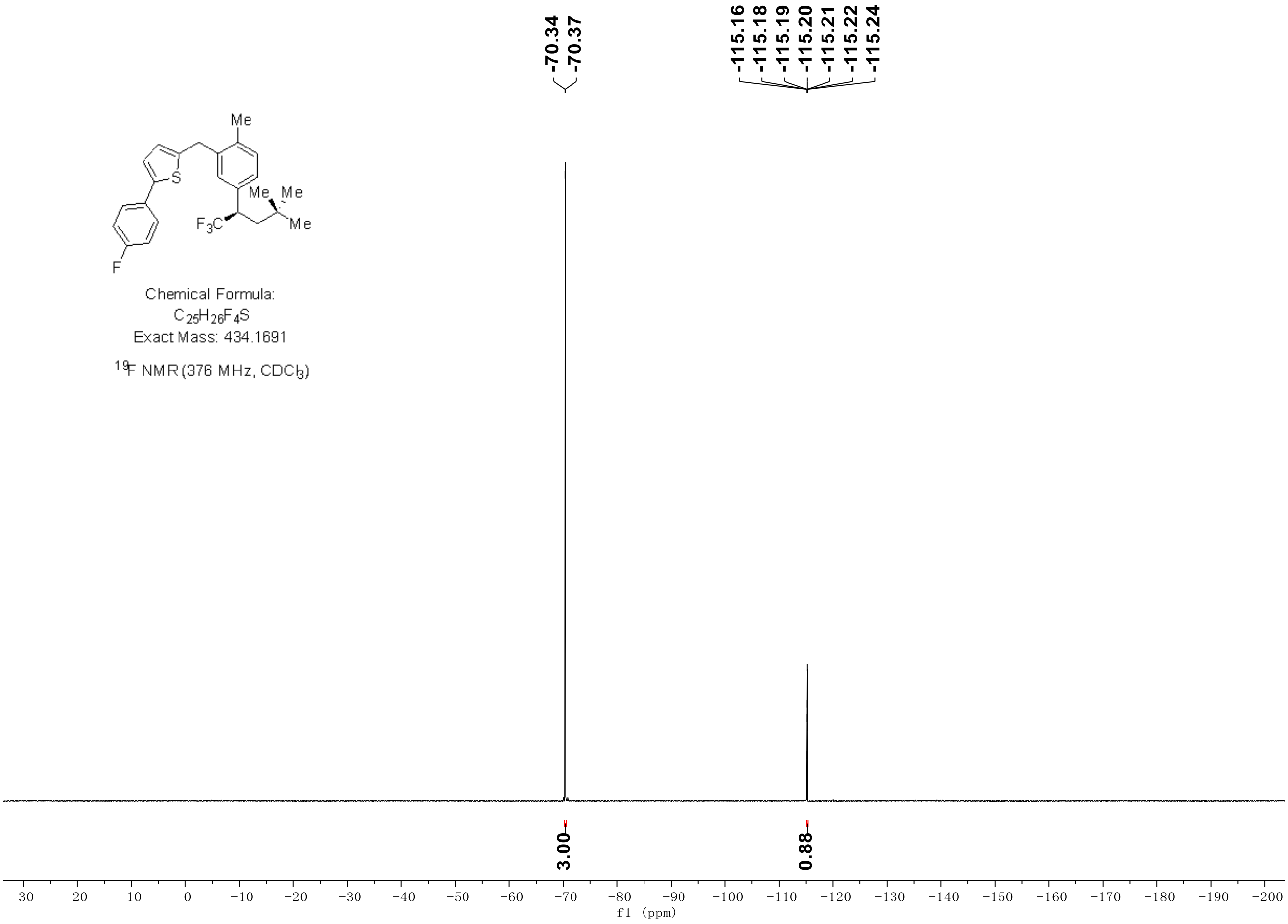

Supplementary Figure 92. ^{19}F NMR spectrum of compound 4p

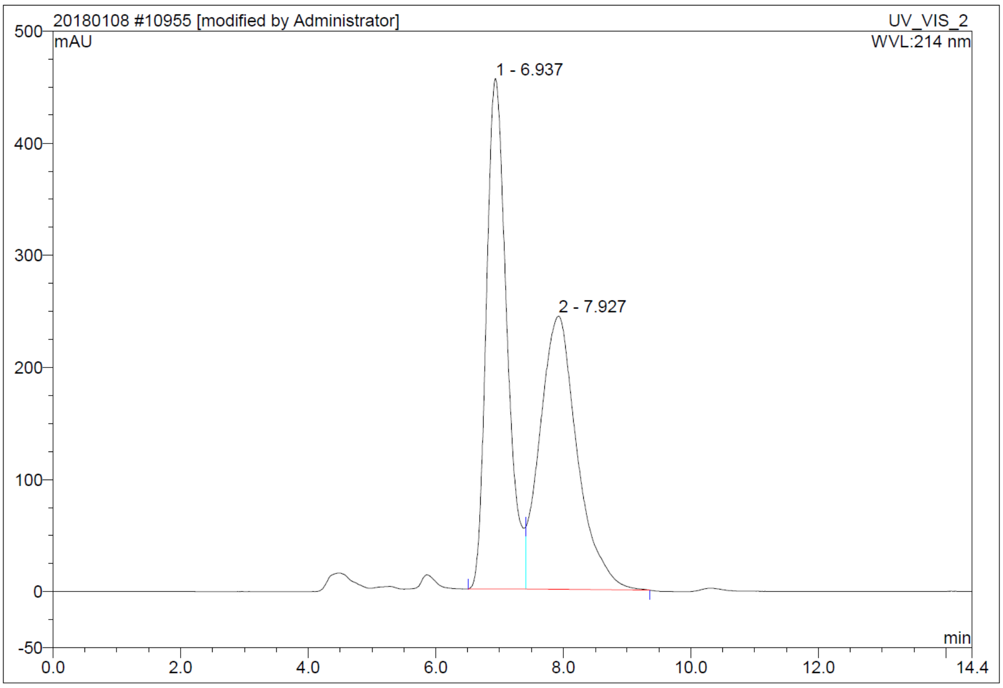

No.	Ret.Time min	Peak Name	Height mAU	Area mAU*min	Rel.Area %	Amount	Type
1	6.94	n.a.	455.062	169.663	50.66	n.a.	BM *
2	7.93	n.a.	243.539	165.243	49.34	n.a.	MB*
Total:			698.601	334.906	100.00	0.000	

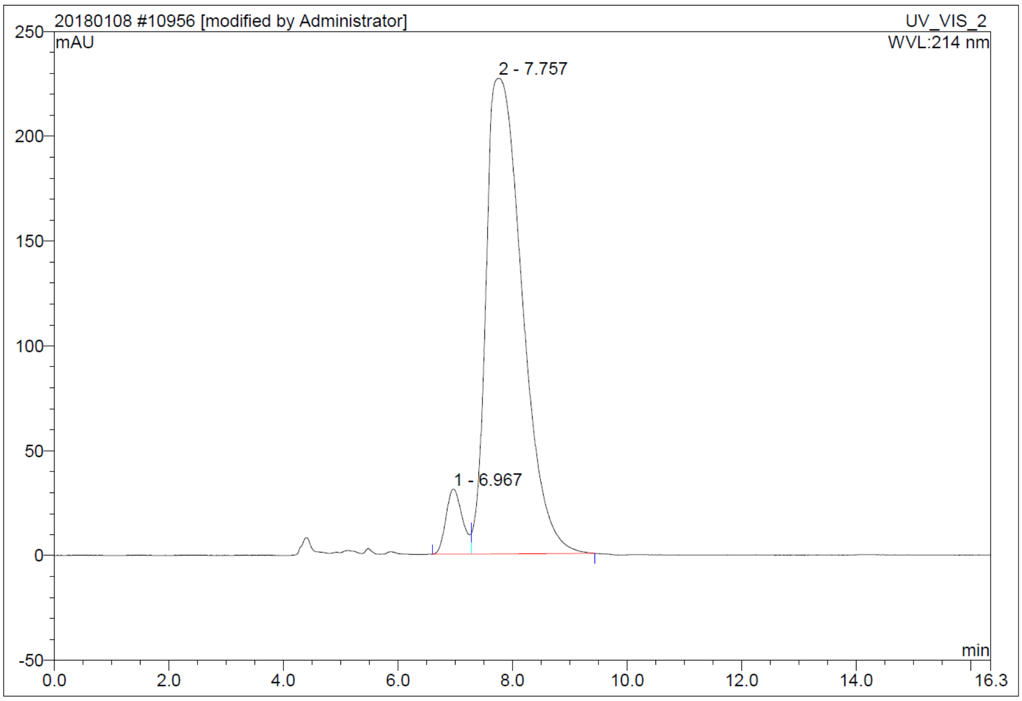

No.	Ret.Time min	Peak Name	Height mAU	Area mAU*min	Rel.Area %	Amount	Type
1	6.97	n.a.	31.155	10.552	5.98	n.a.	BM *
2	7.76	n.a.	227.028	165.919	94.02	n.a.	MB*
Total:			258.183	176.471	100.00	0.000	

Supplementary Figure 93. Chiral HPLC analysis of compound 4p

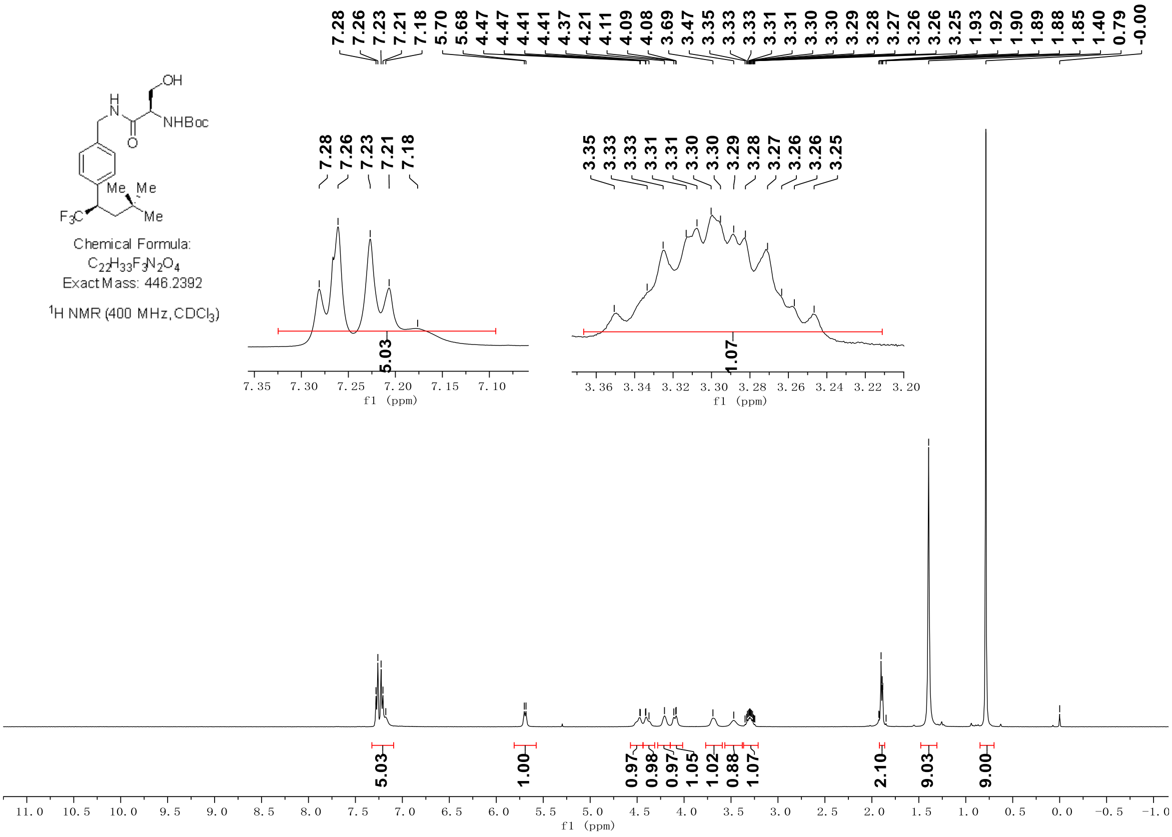

Supplementary Figure 94. ^{1}H NMR spectrum of compound 4q

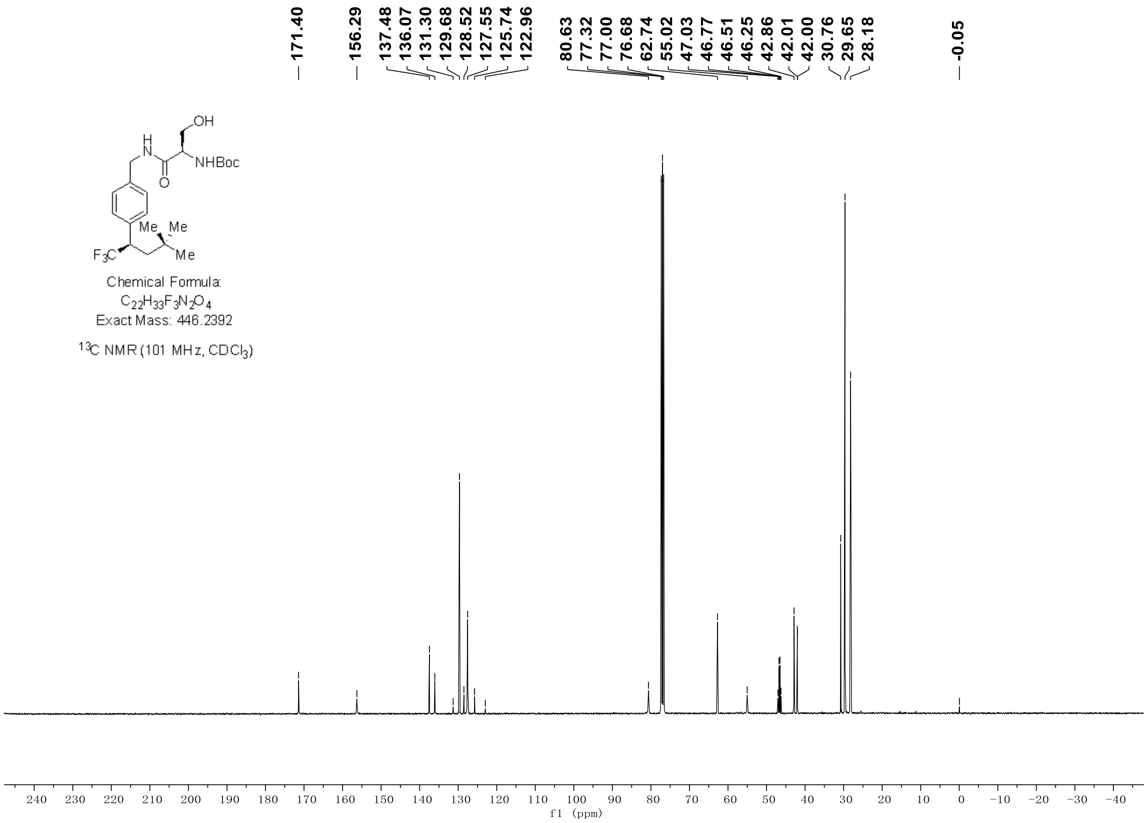

Supplementary Figure 95. ^{13}C NMR spectrum of compound 4q

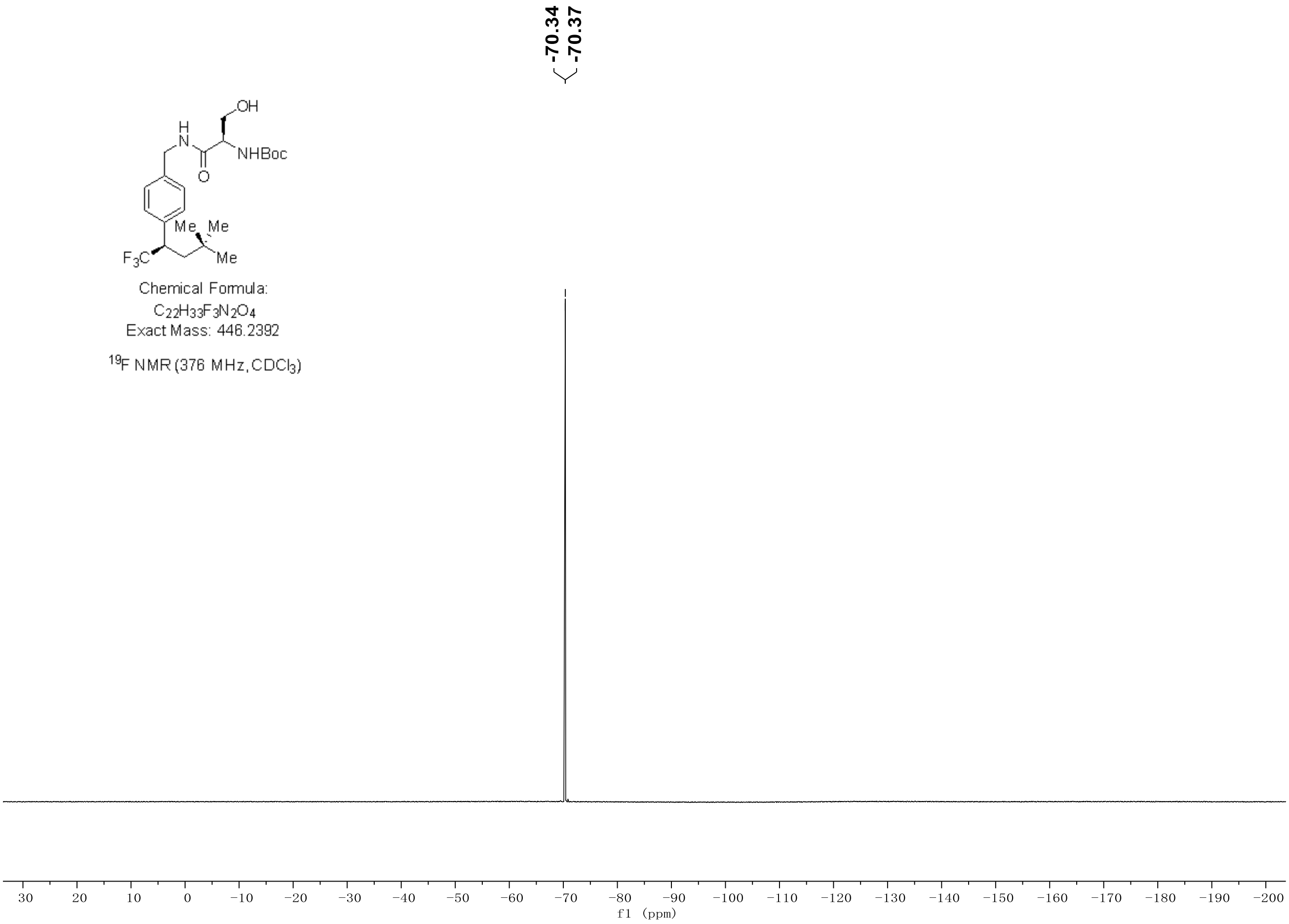

Supplementary Figure 96. ^{19}F NMR spectrum of compound 4q

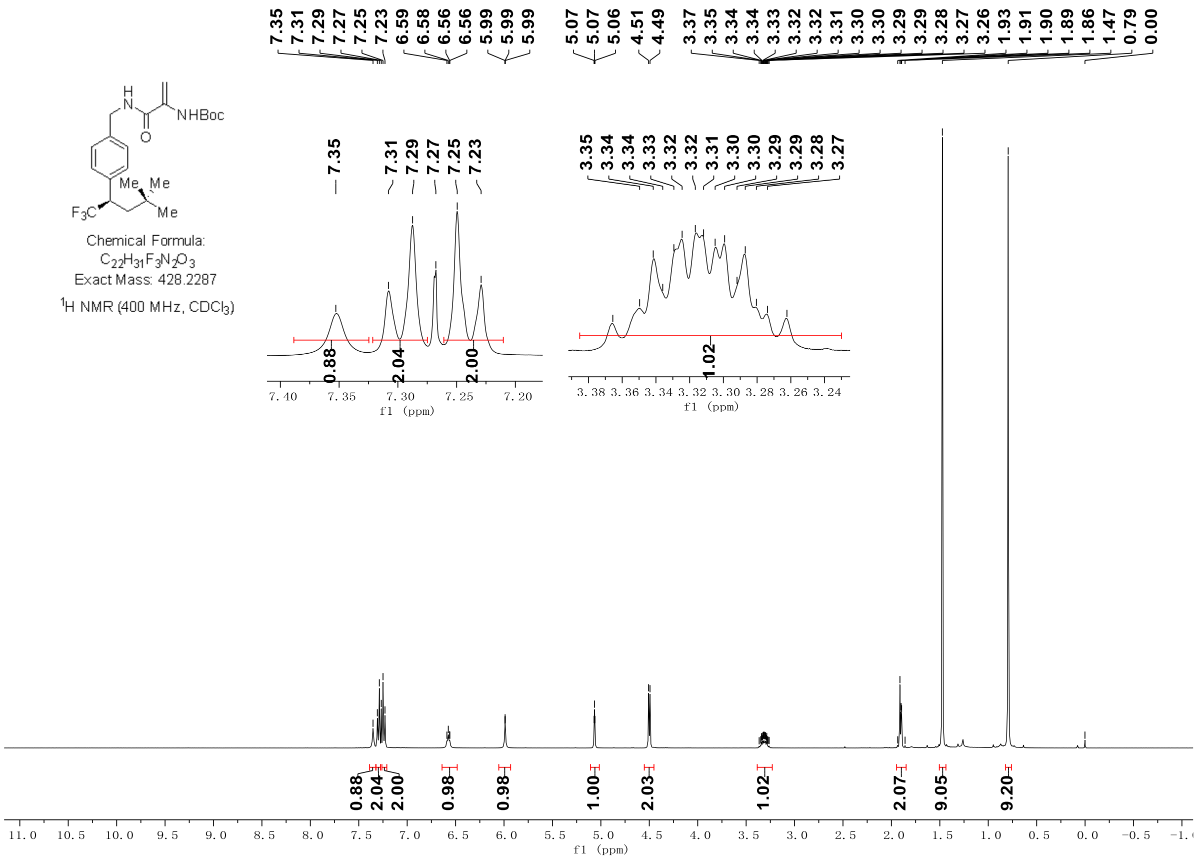

Supplementary Figure 97. ^{1}H NMR spectrum of compound 4q'

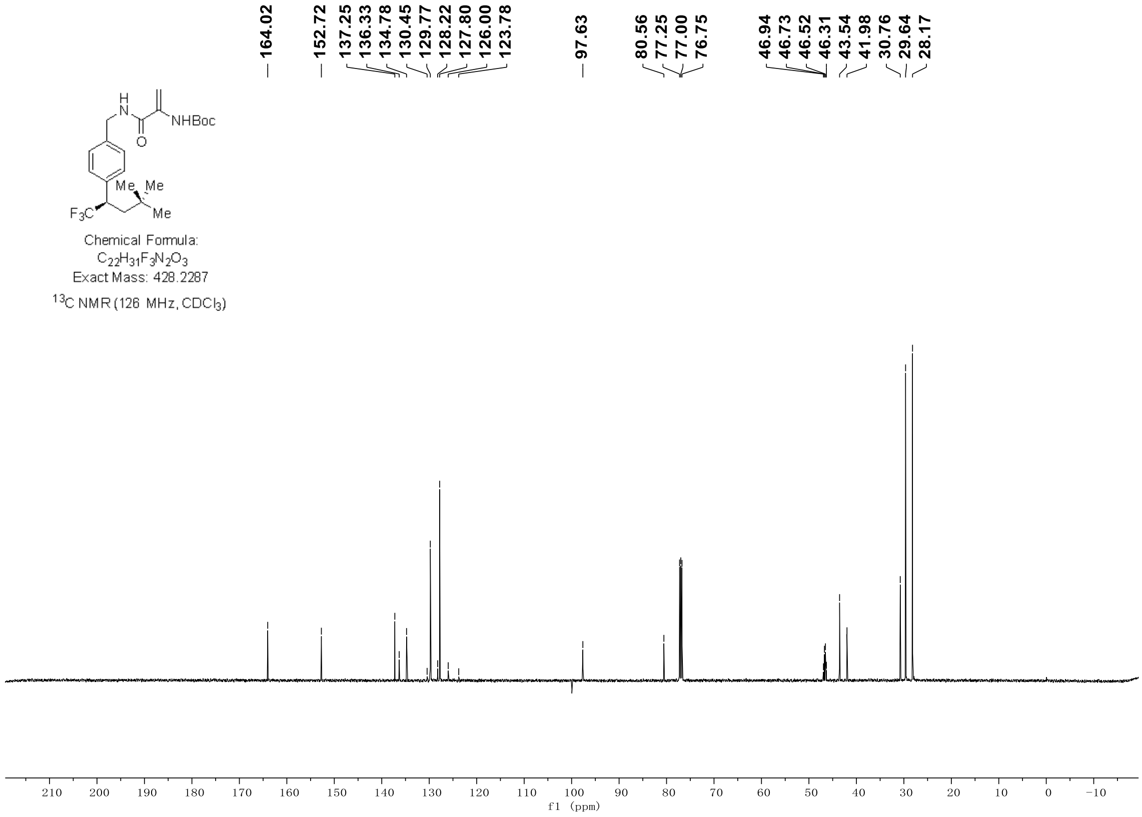

Supplementary Figure 98. ^{13}C NMR spectrum of compound 4q'

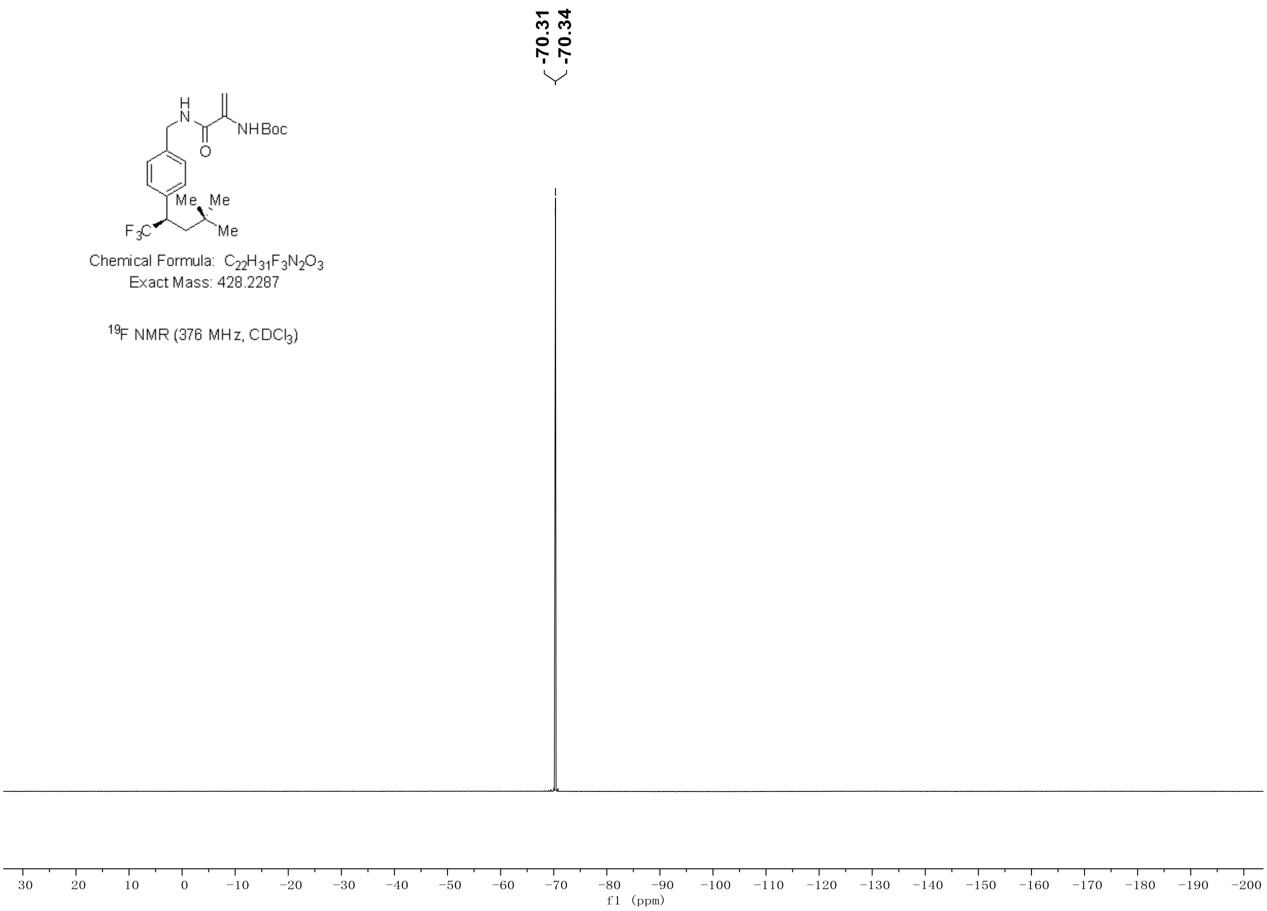

Supplementary Figure 99. ^{19}F NMR spectrum of compound 4q'

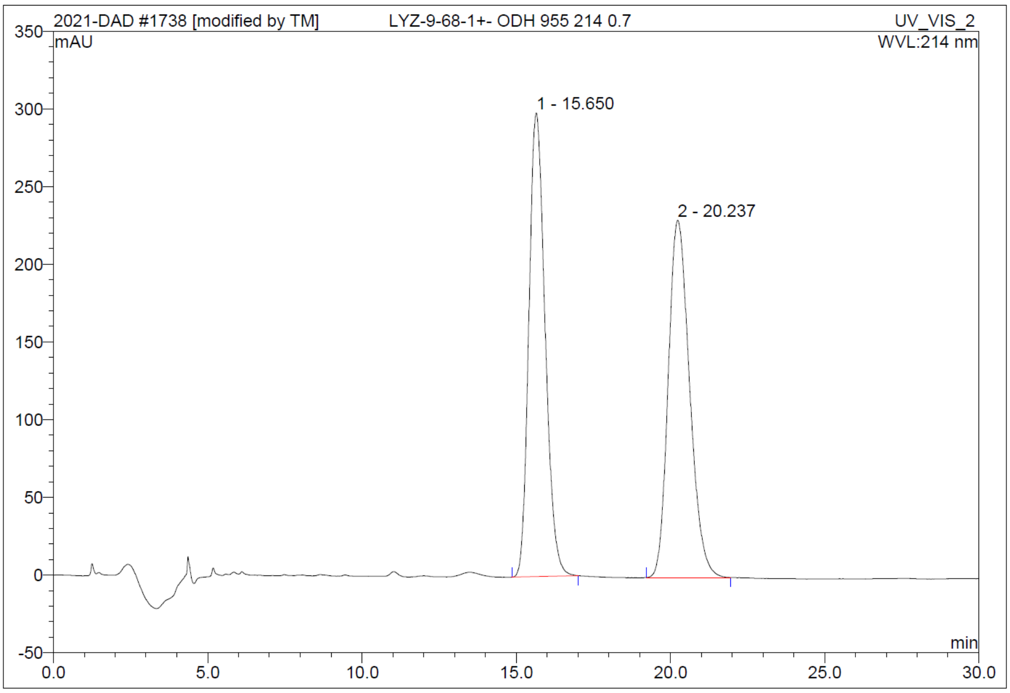

No.	Ret.Time min	Peak Name	Height mAU	Area mAU*min	Rel.Area %	Amount	Type
1	15.65	n.a.	298.522	181.682	49.61	n.a.	BMB
2	20.24	n.a.	229.905	184.547	50.39	n.a.	BMB
Total:			528.427	366.229	100.00	0.000	

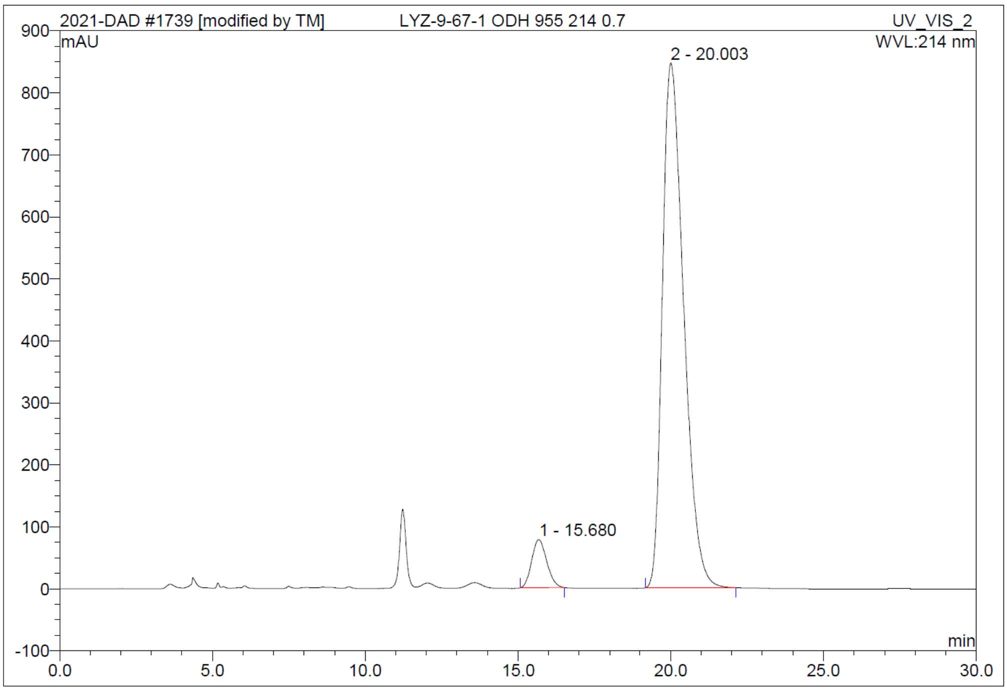

No.	Ret.Time min	Peak Name	Height mAU	Area mAU*min	Rel.Area %	Amount	Type
1	15.68	n.a.	77.573	44.386	6.10	n.a.	BMB*
2	20.00	n.a.	846.717	683.222	93.90	n.a.	BMB*
Total:			924.291	727.607	100.00	0.000	

Supplementary Figure 100. Chiral HPLC analysis of compound 4q'

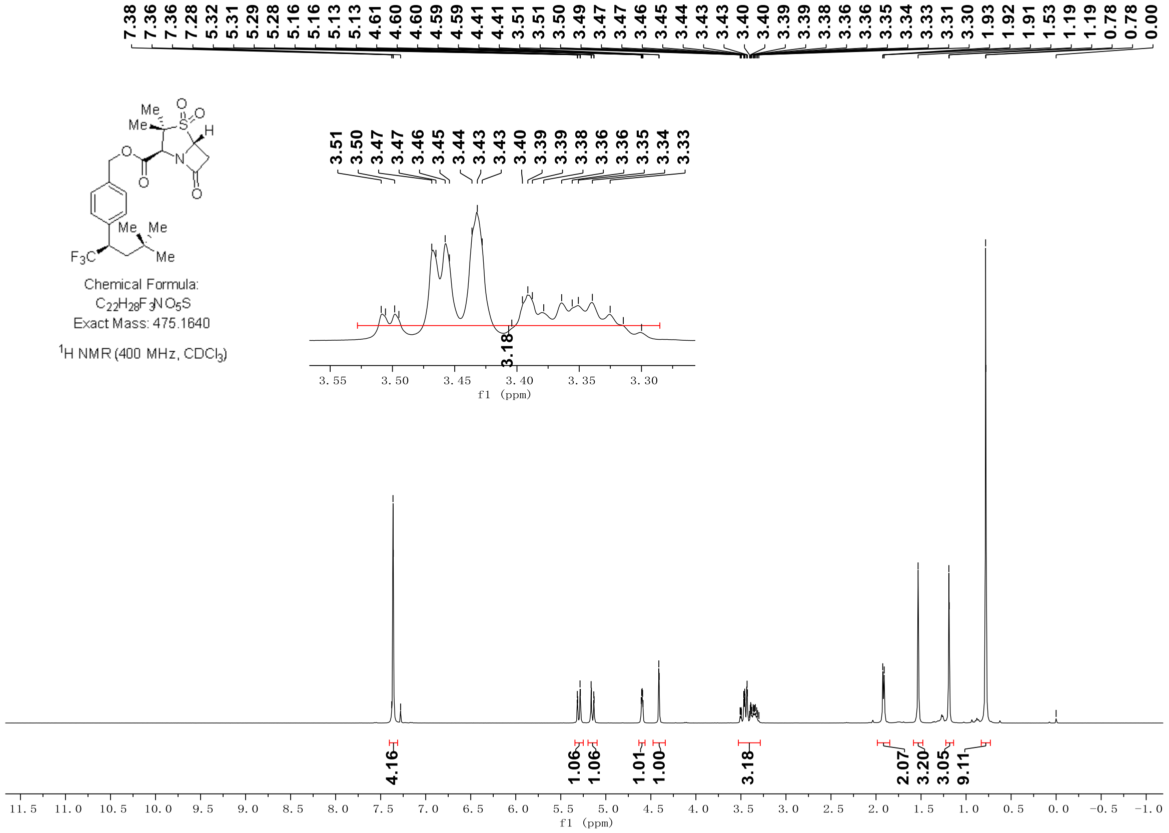

Supplementary Figure 101. ^{1}H NMR spectrum of compound 4r

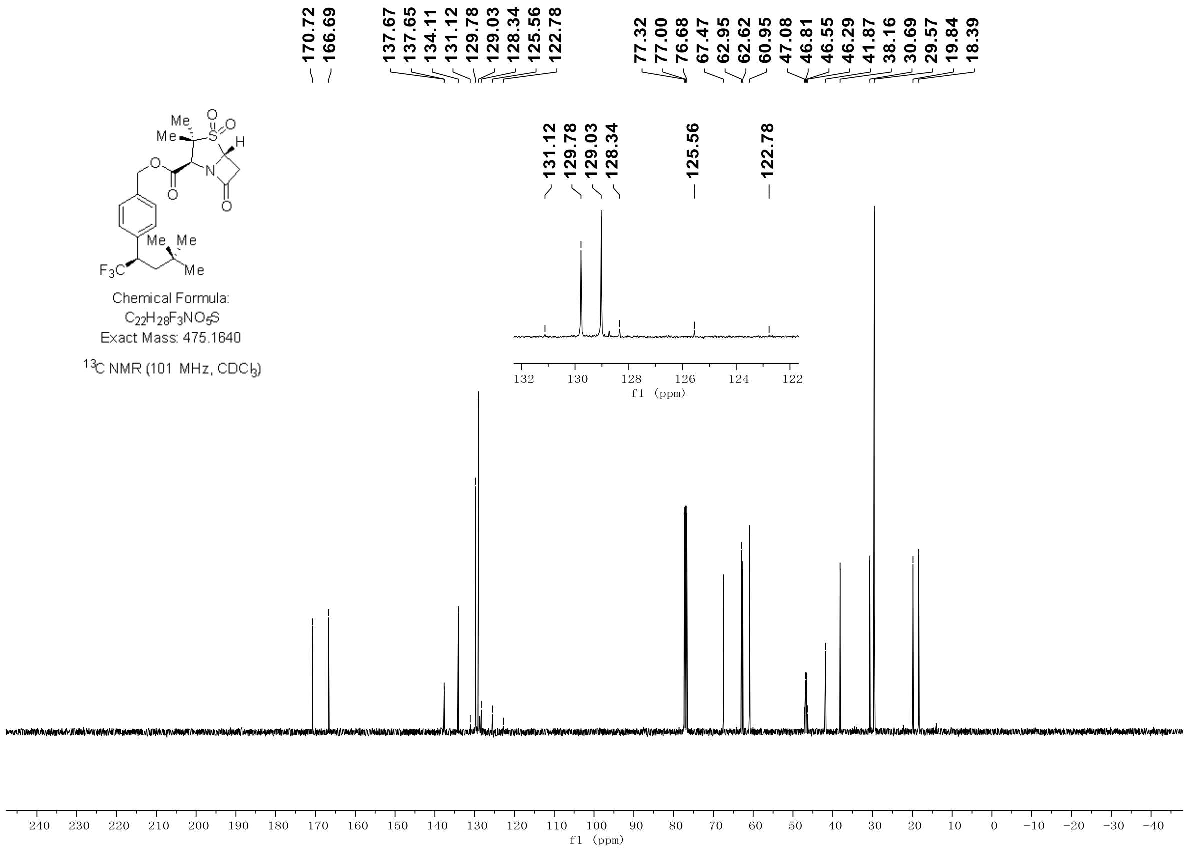

Supplementary Figure 102. ^{13}C NMR spectrum of compound 4r

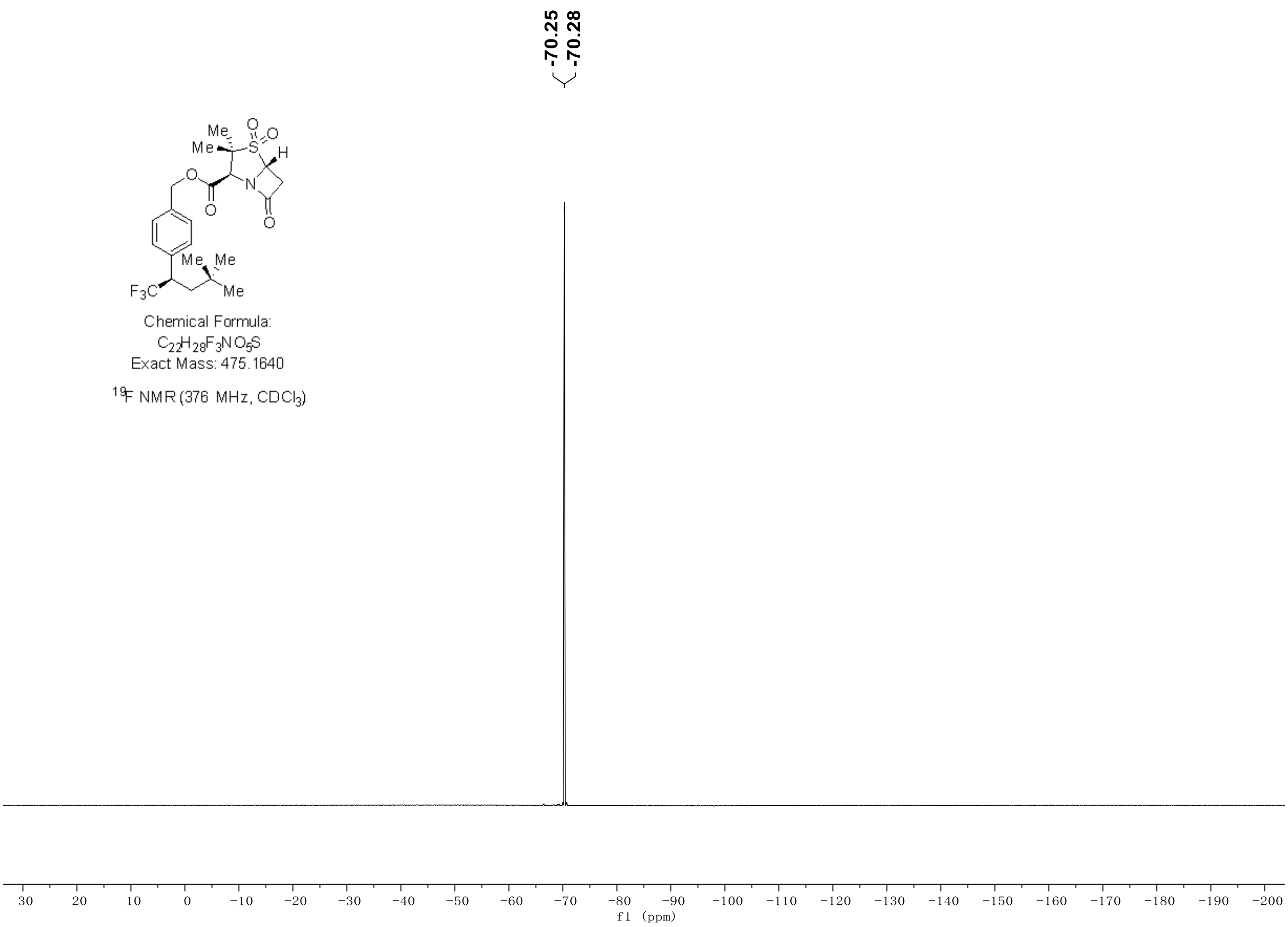

Supplementary Figure 103. ^{19}F NMR spectrum of compound 4r

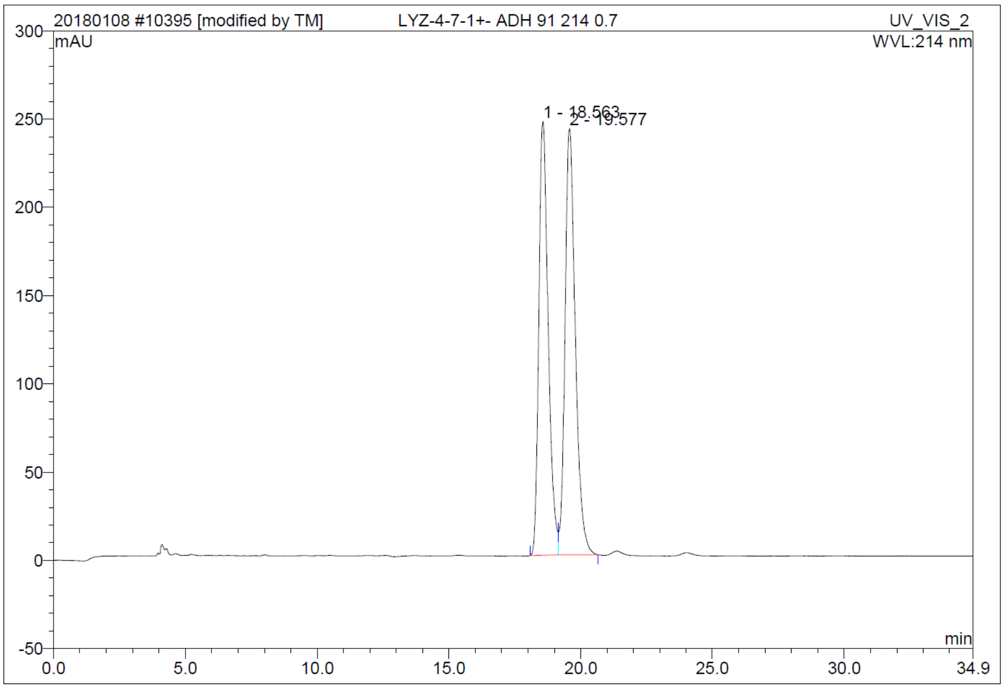

No.	Ret.Time min	Peak Name	Height mAU	Area mAU*min	Rel.Area %	Amount	Type
1	18.56	n.a.	245.918	101.449	48.14	n.a.	BM *
2	19.58	n.a.	241.688	109.278	51.86	n.a.	MB*
Total:			487.606	210.727	100.00	0.000	

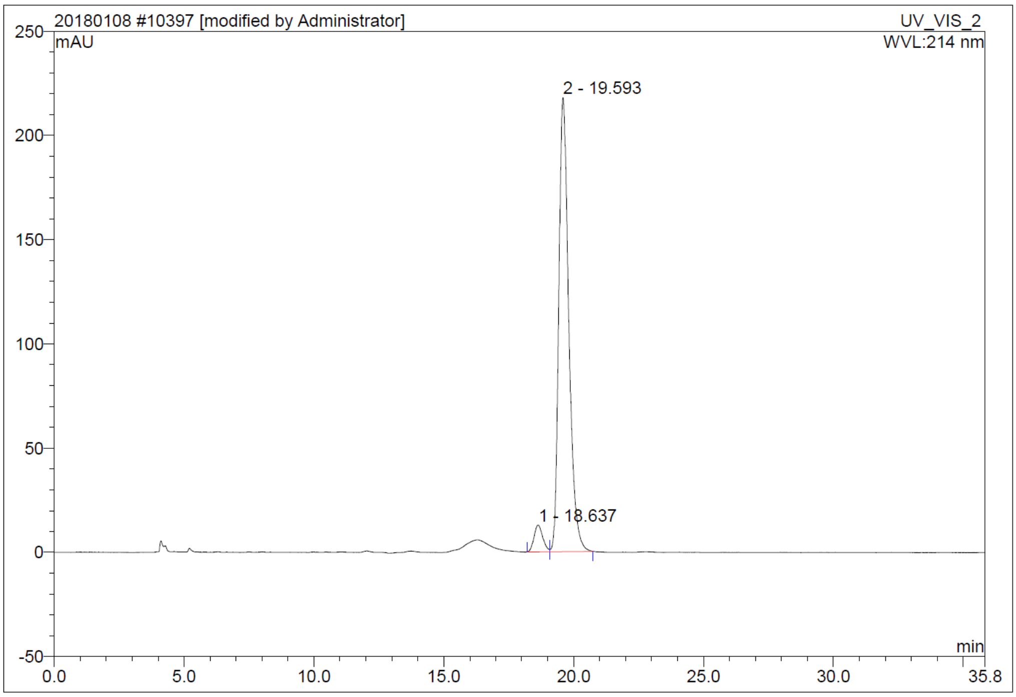

No.	Ret.Time min	Peak Name	Height mAU	Area mAU*min	Rel.Area %	Amount	Type
1	18.64	n.a.	12.883	5.017	4.92	n.a.	BM
2	19.59	n.a.	217.823	97.026	95.08	n.a.	MB
Total:			230.706	102.043	100.00	0.000	

Supplementary Figure 104. Chiral HPLC analysis of compound 4r

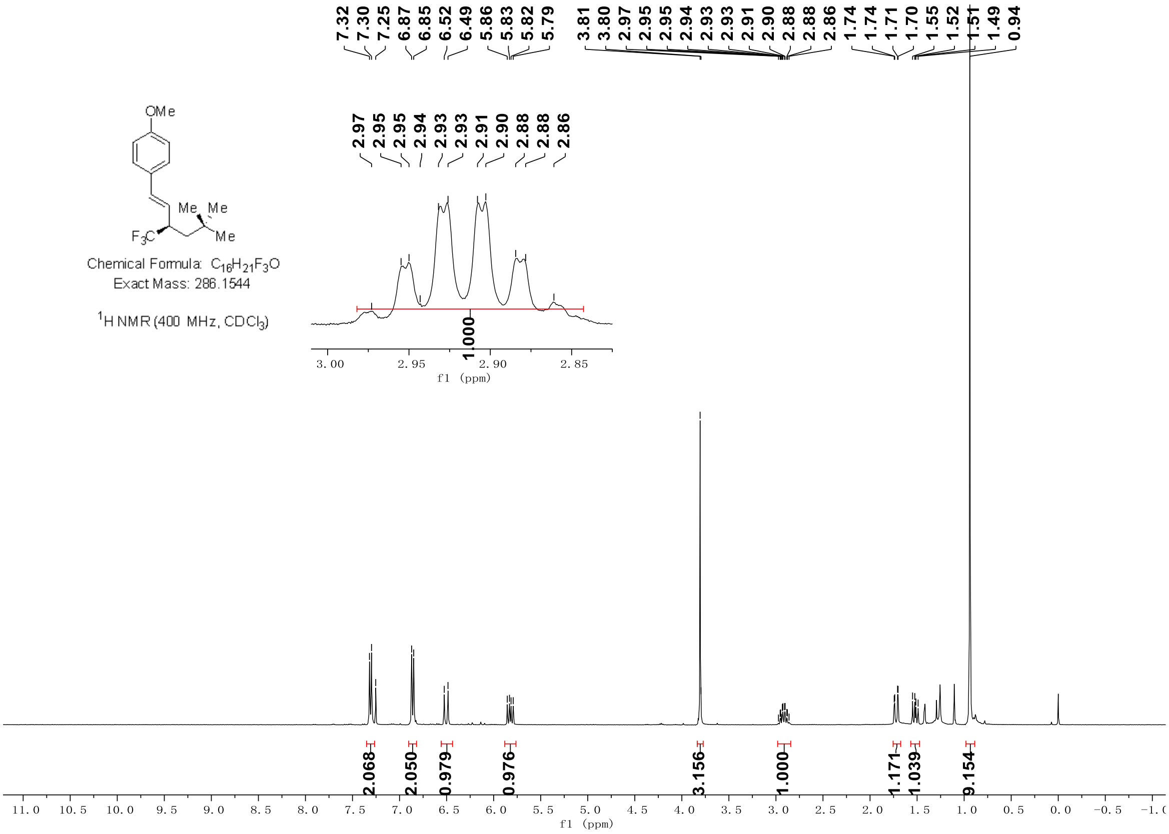

Supplementary Figure 105. ^{1}H NMR spectrum of compound 4s

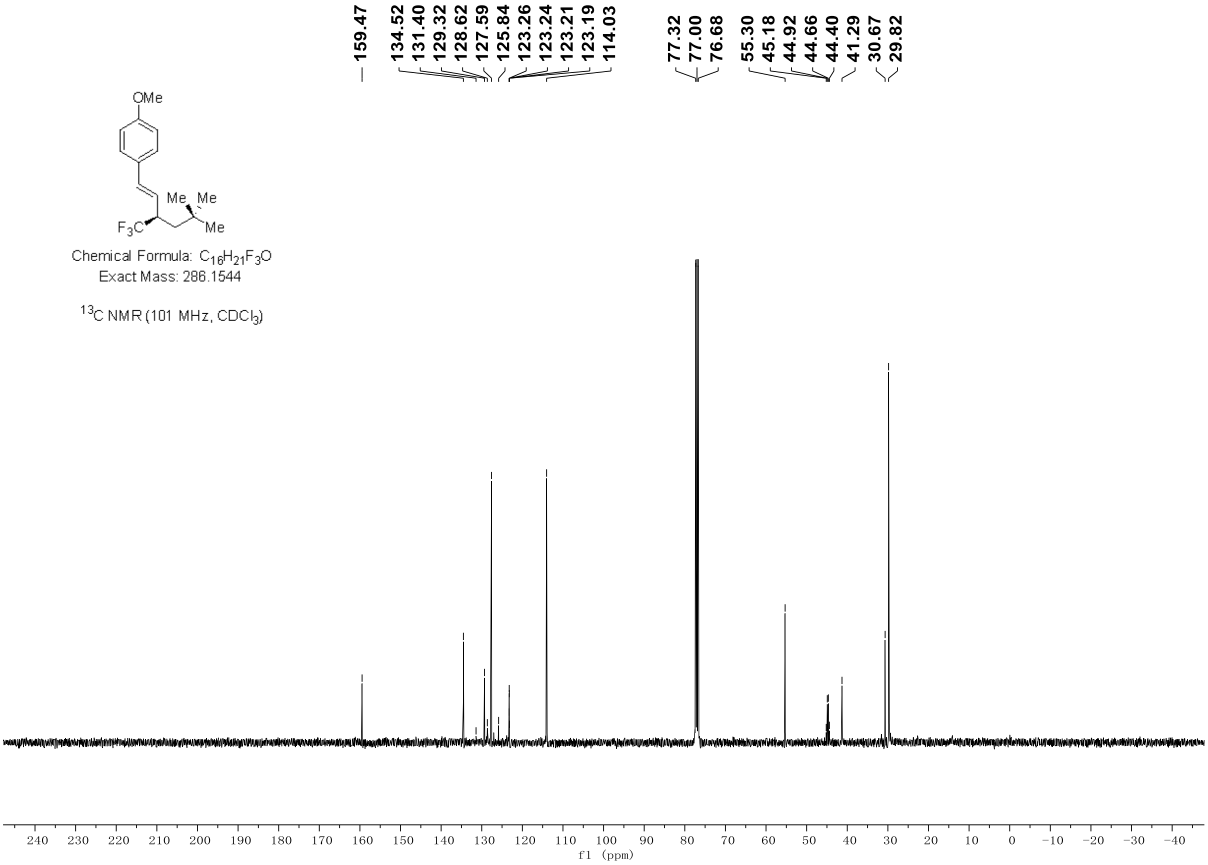

Supplementary Figure 106. ^{13}C NMR spectrum of compound 4s

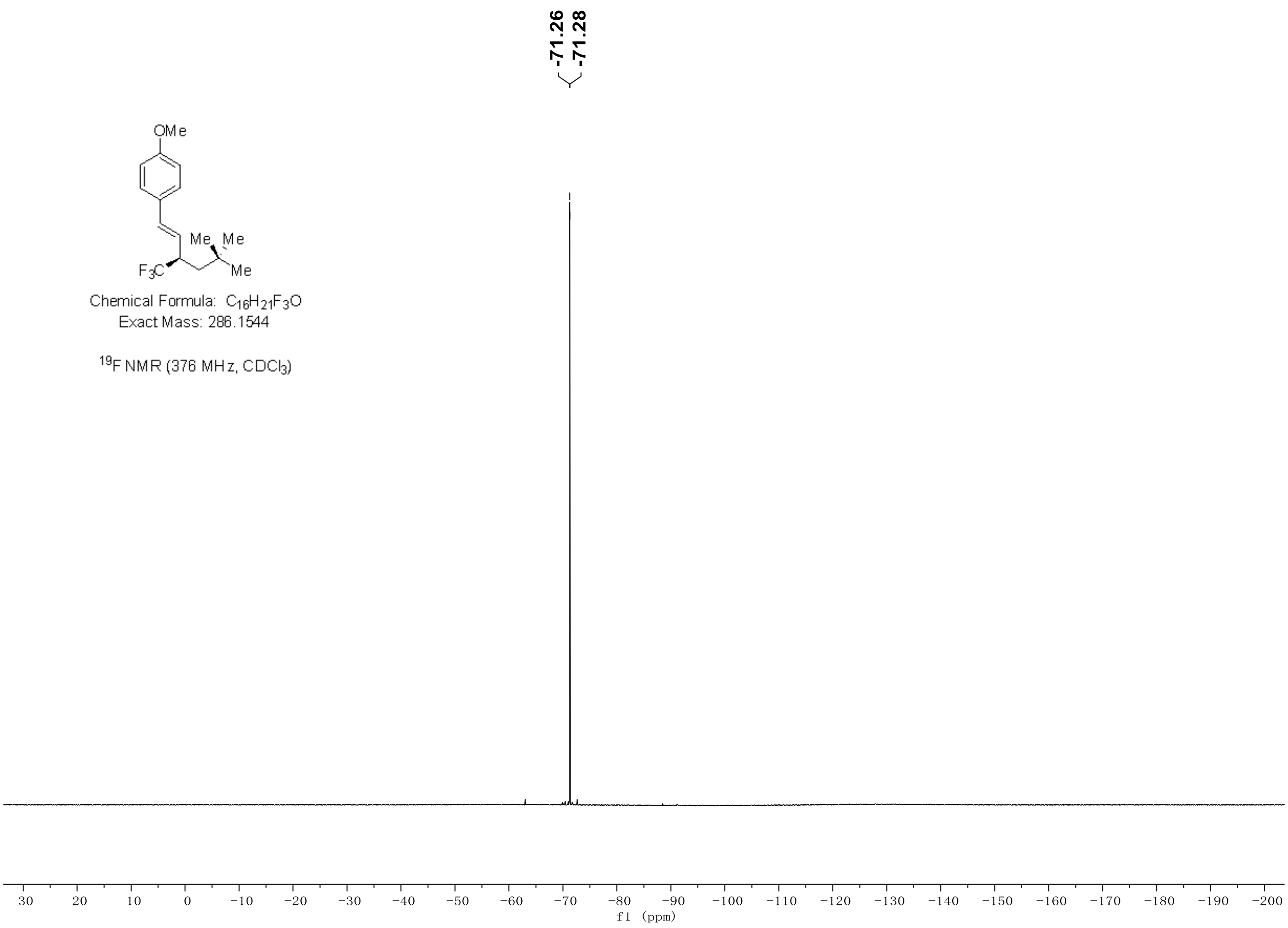

Supplementary Figure 107. ^{19}F NMR spectrum of compound 4s

Chromatogram

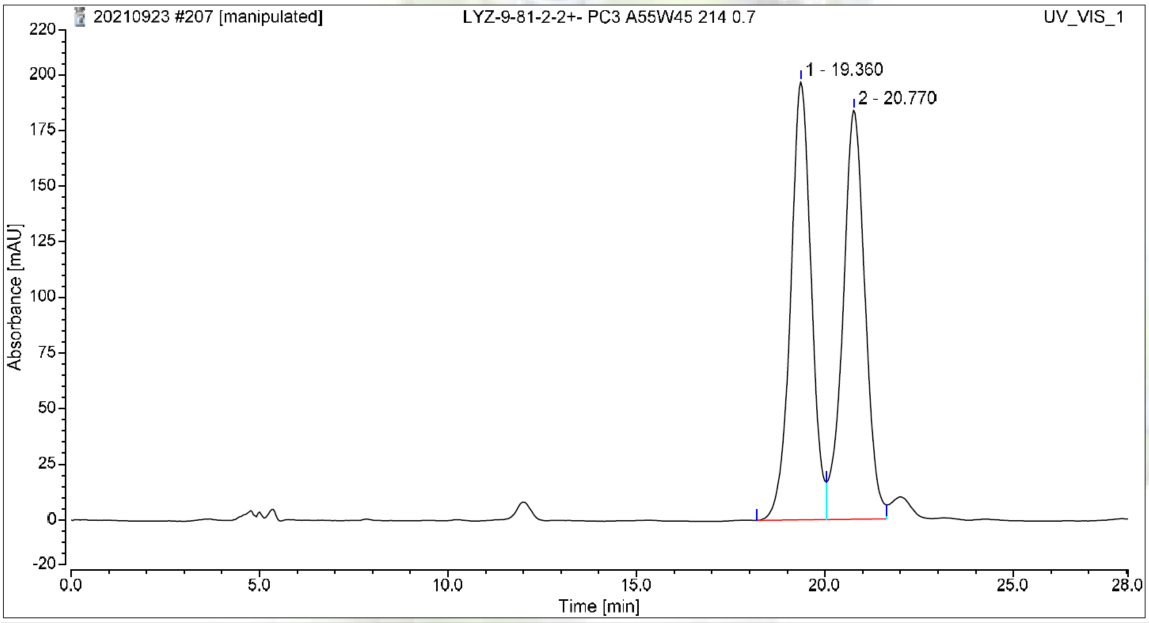

Integration Results								
No.	Retention Time min	Area mAU*min	Height mAU	Relative Area %	Resolution (EP)	Asymmetry (EP)	K'	Plates (EP)
n.a.	19.360	124.8155	196.9774	49.962	1.40	n.a.	n.a.	6276
n.a.	20.770	125.0074	184.0092	50.038	n.a.	n.a.	n.a.	6354
**Total:**		**249.823**	**1401.998**	**100.000**				

Chromatogram

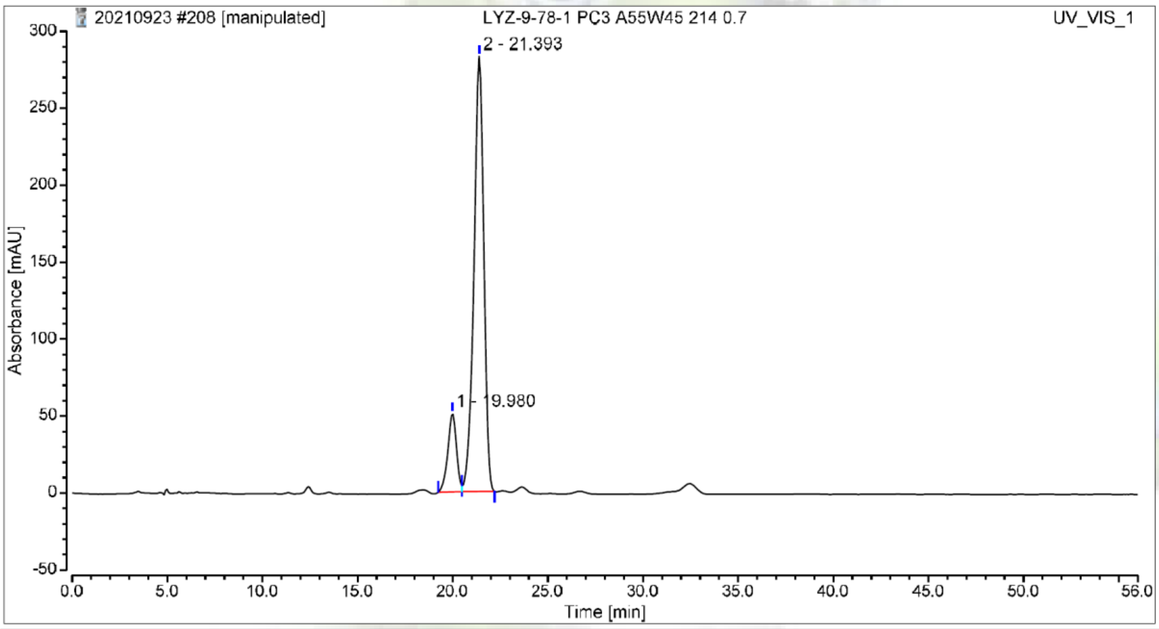

Integration Results								
No.	Retention Time min	Area mAU*min	Height mAU	Relative Area %	Resolution (EP)	Asymmetry (EP)	K'	Plates (EP)
n.a.	19.980	26.8421	50.9868	13.659	1.62	n.a.	n.a.	9493
n.a.	21.393	169.6788	282.8467	86.341	n.a.	0.90	n.a.	8522
**Total:**		**196.521**	**1401.998**	**100.000**				

Supplementary Figure 108. Chiral HPLC analysis of compound 4s

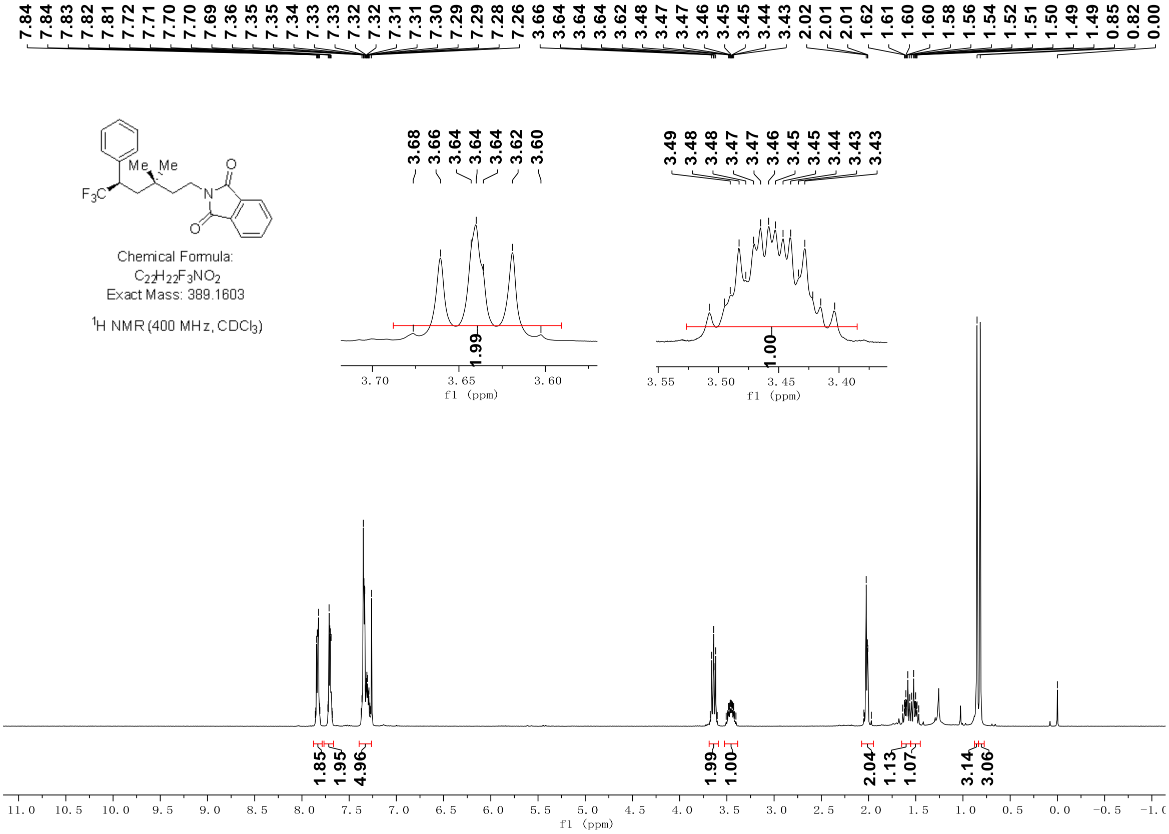

Supplementary Figure 109. ^{1}H NMR spectrum of compound 5a

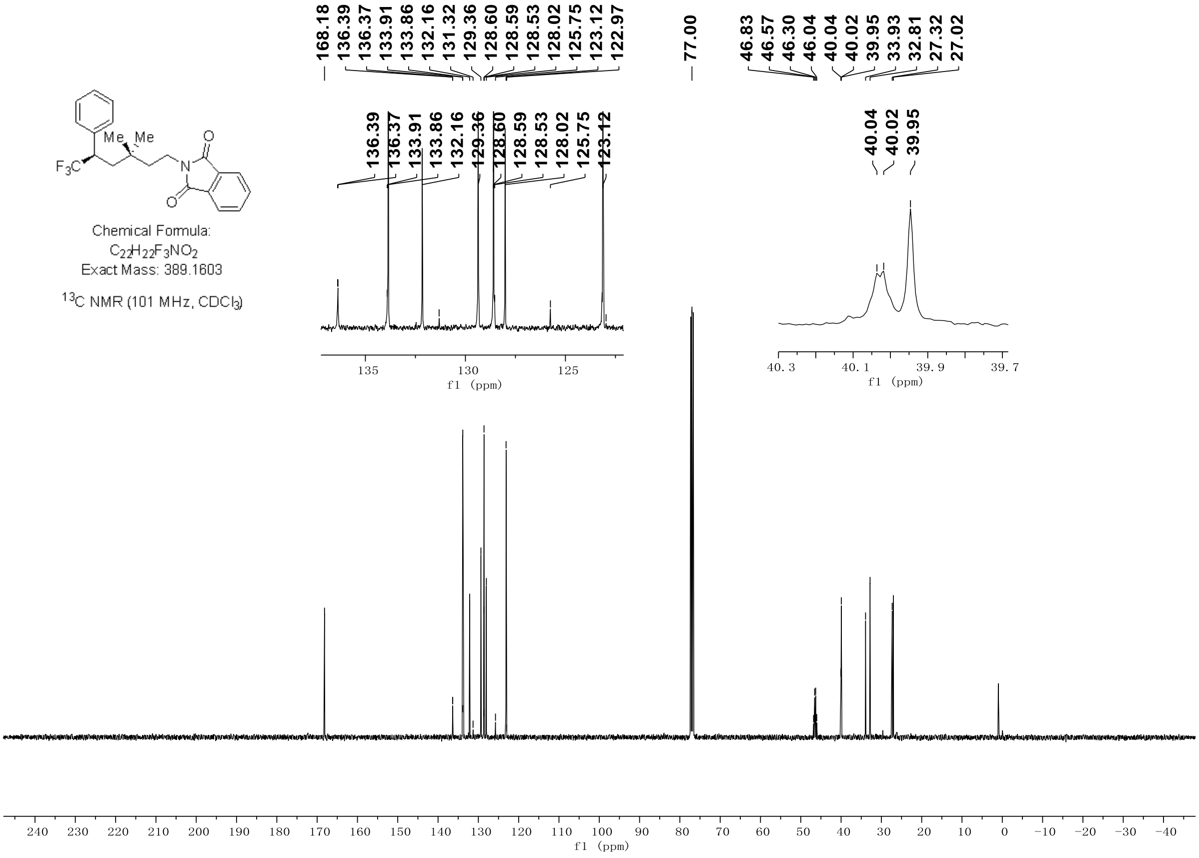

Supplementary Figure 110. ^{13}C NMR spectrum of compound 5a

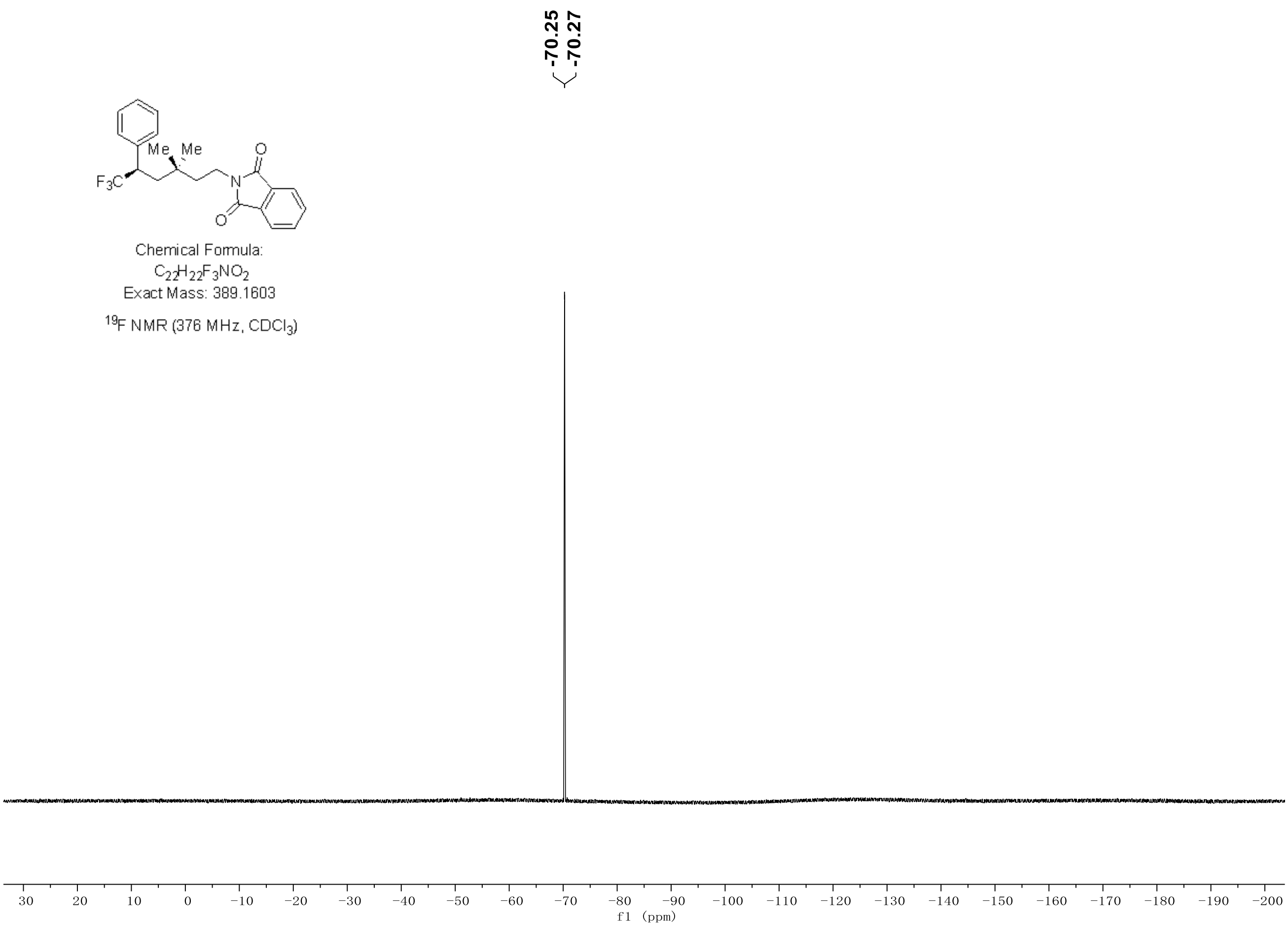

Supplementary Figure 111. ^{19}F NMR spectrum of compound 5a

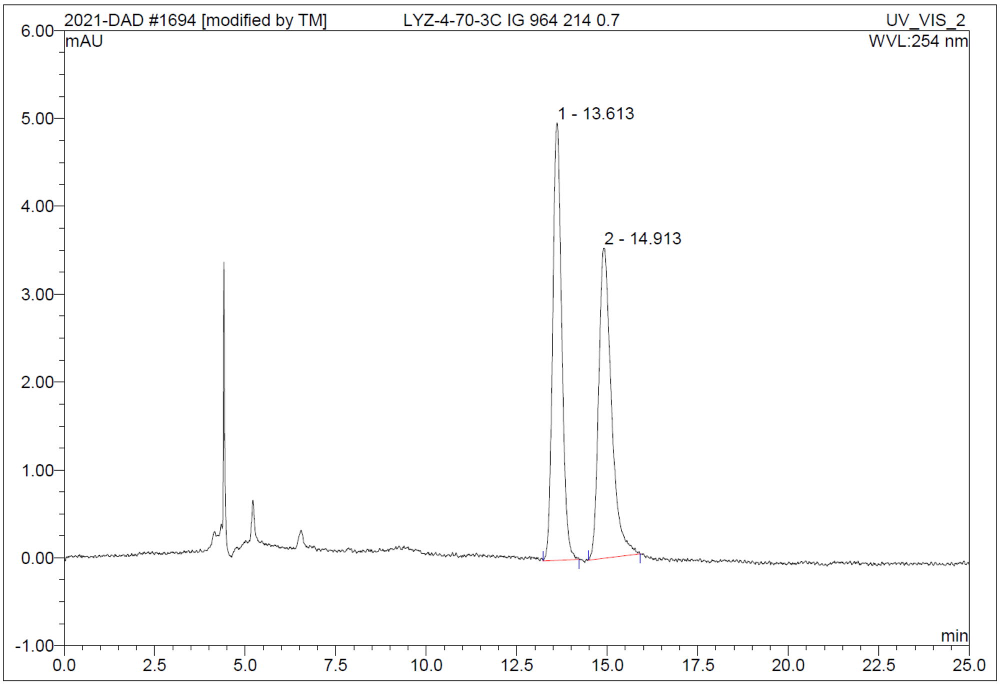

No.	Ret.Time min	Peak Name	Height mAU	Area mAU*min	Rel.Area %	Amount	Type
1	13.61	n.a.	4.981	1.437	49.91	n.a.	BMB*
2	14.91	n.a.	3.526	1.442	50.09	n.a.	BMB*
Total:			8.508	2.879	100.00	0.000	

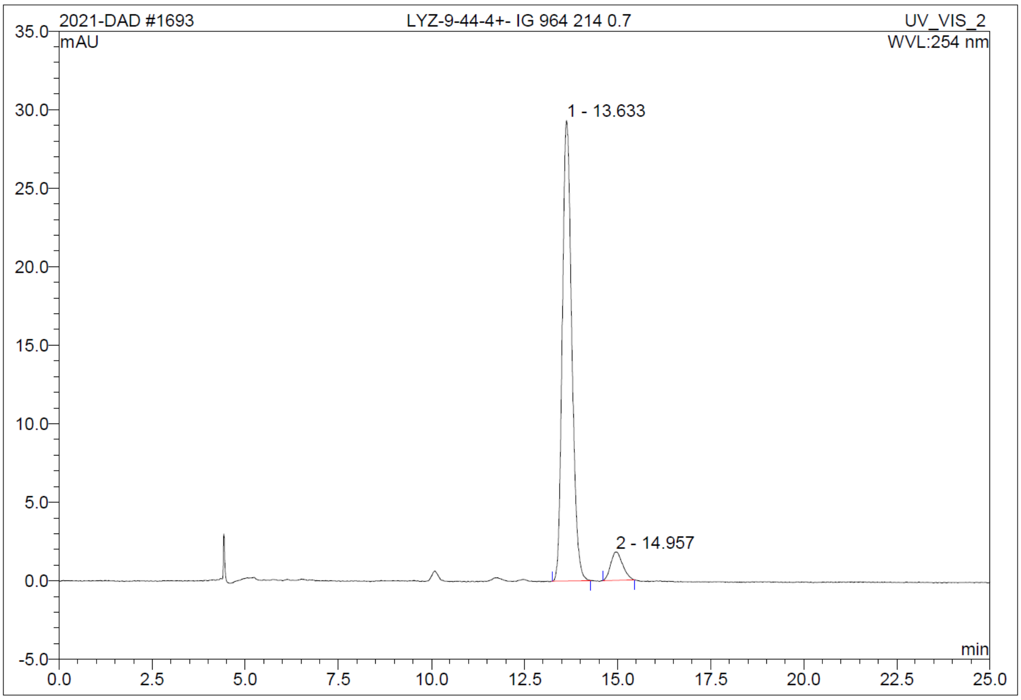

No.	Ret.Time min	Peak Name	Height mAU	Area mAU*min	Rel.Area %	Amount	Type
1	13.63	n.a.	29.326	8.639	92.79	n.a.	BMB
2	14.96	n.a.	1.793	0.671	7.21	n.a.	BMB
Total:			31.119	9.311	100.00	0.000	

Supplementary Figure 112. Chiral HPLC analyses of compound 5a

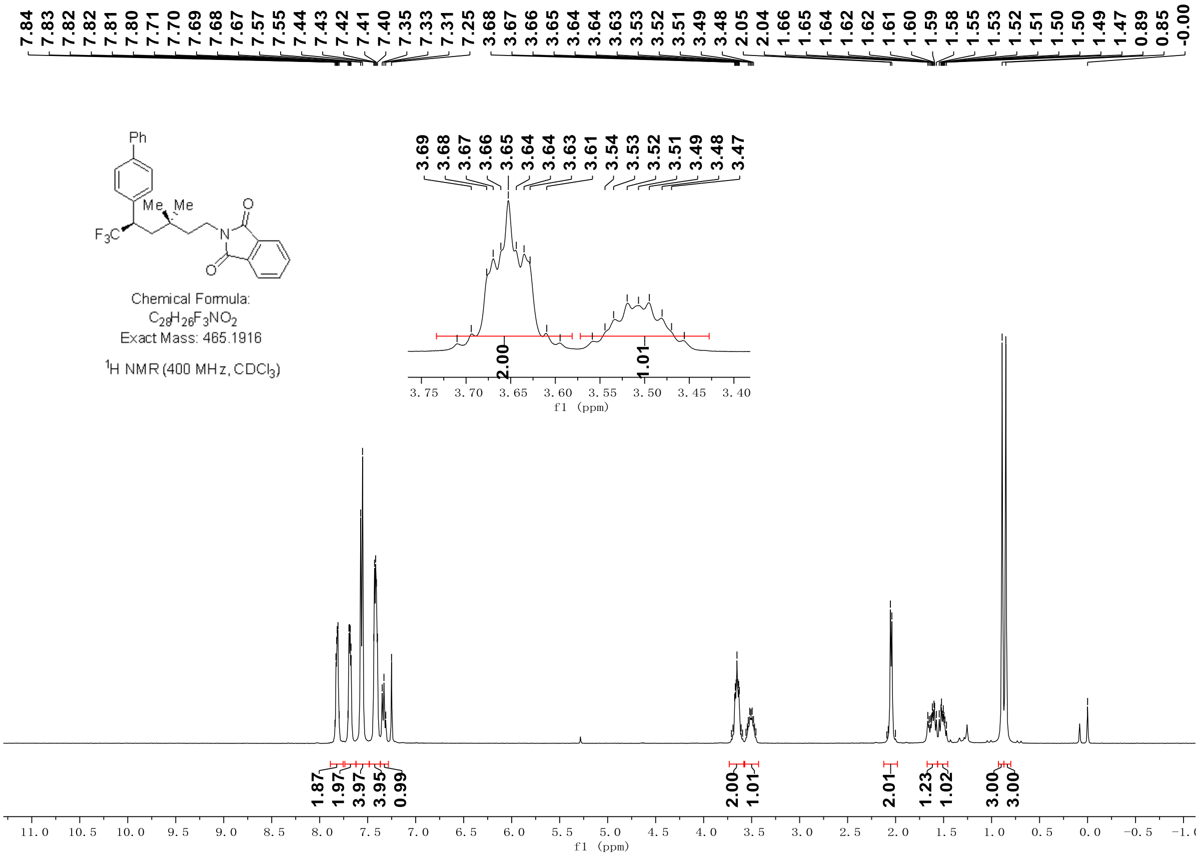

Supplementary Figure 113. ^{1}H NMR spectrum of compound 5b

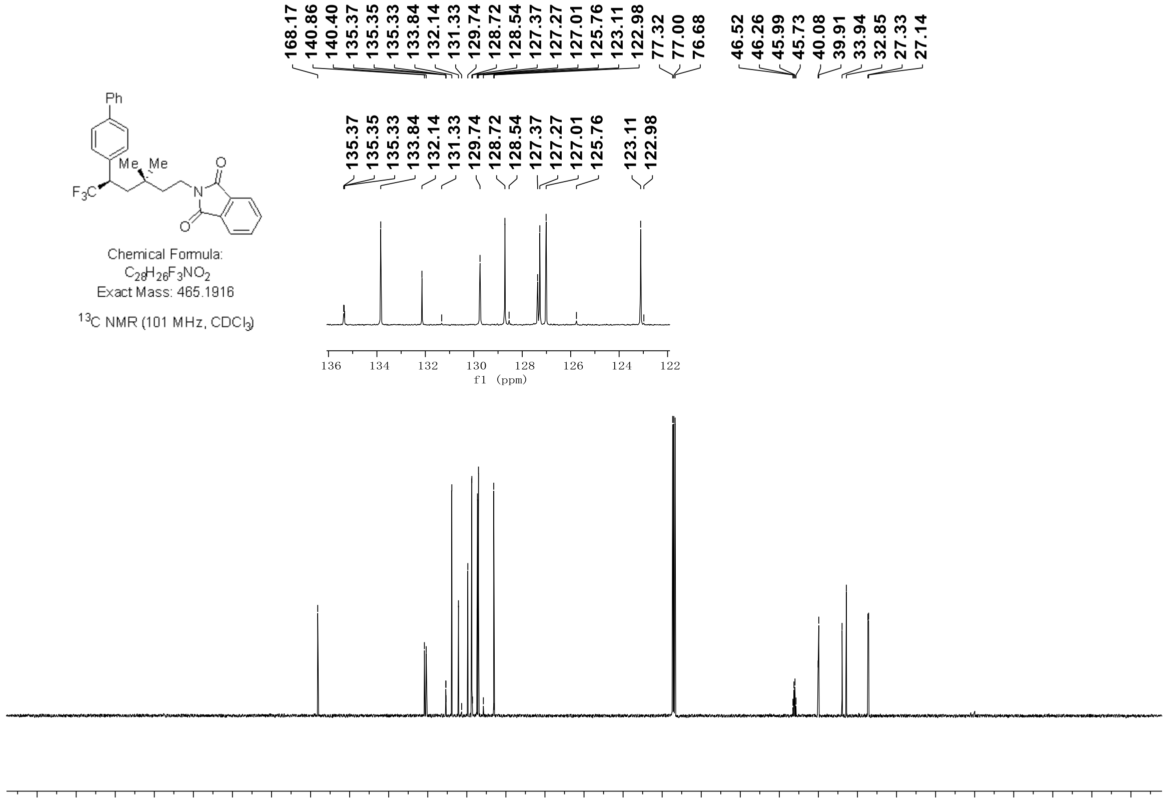

Supplementary Figure 114. ^{13}C NMR spectrum of compound 5b

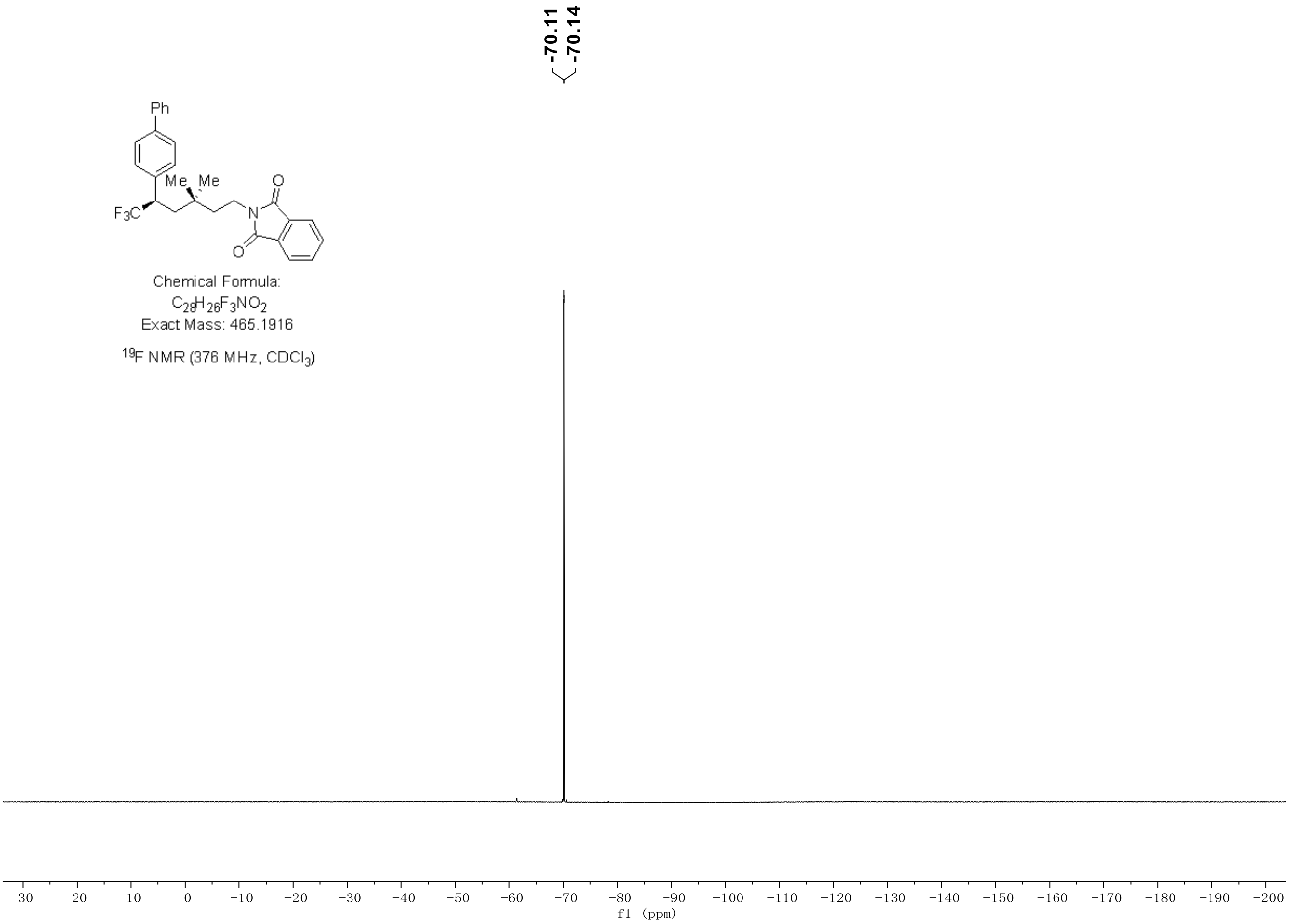

Supplementary Figure 115. ^{19}F NMR spectrum of compound 5b

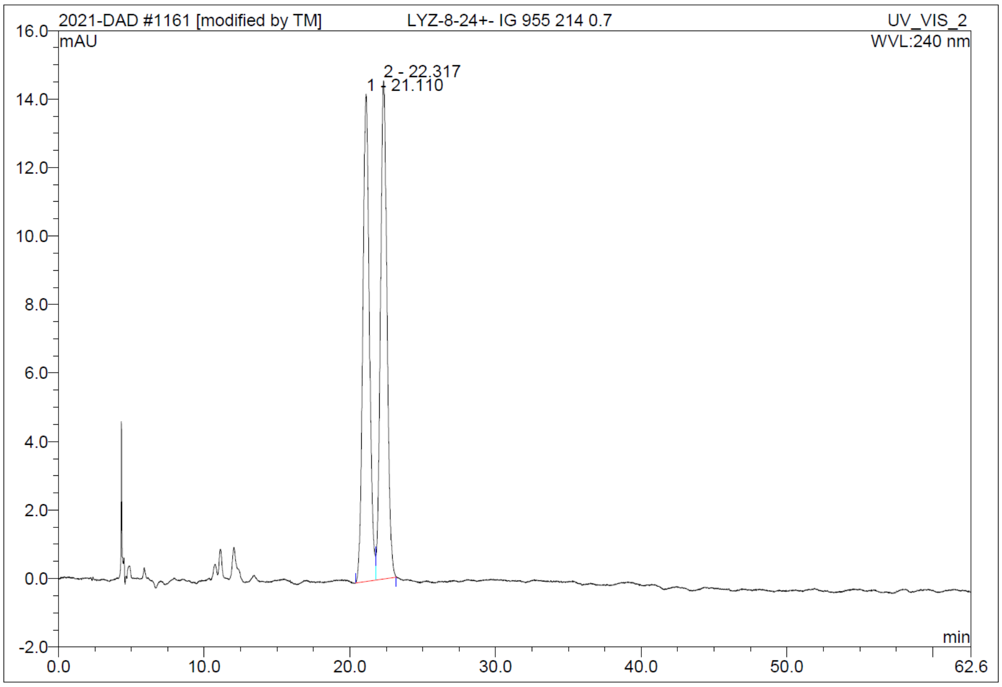

No.	Ret.Time min	Peak Name	Height mAU	Area mAU*min	Rel.Area %	Amount	Type
1	21.11	n.a.	14.237	7.452	49.46	n.a.	BM
2	22.32	n.a.	14.560	7.614	50.54	n.a.	MB
Total:			28.797	15.066	100.00	0.000	

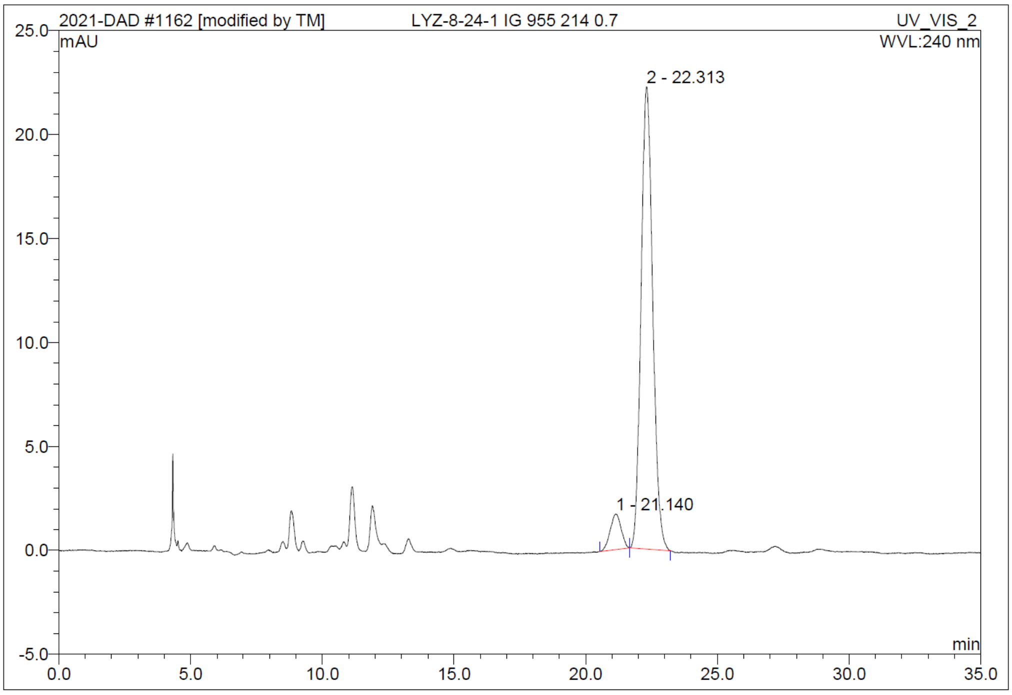

No.	Ret.Time min	Peak Name	Height mAU	Area mAU*min	Rel.Area %	Amount	Type
1	21.14	n.a.	1.724	0.827	6.78	n.a.	BMb*
2	22.31	n.a.	22.253	11.381	93.22	n.a.	bMB*
Total:			23.976	12.208	100.00	0.000	

Supplementary Figure 116. Chiral HPLC analysis of compound 5b

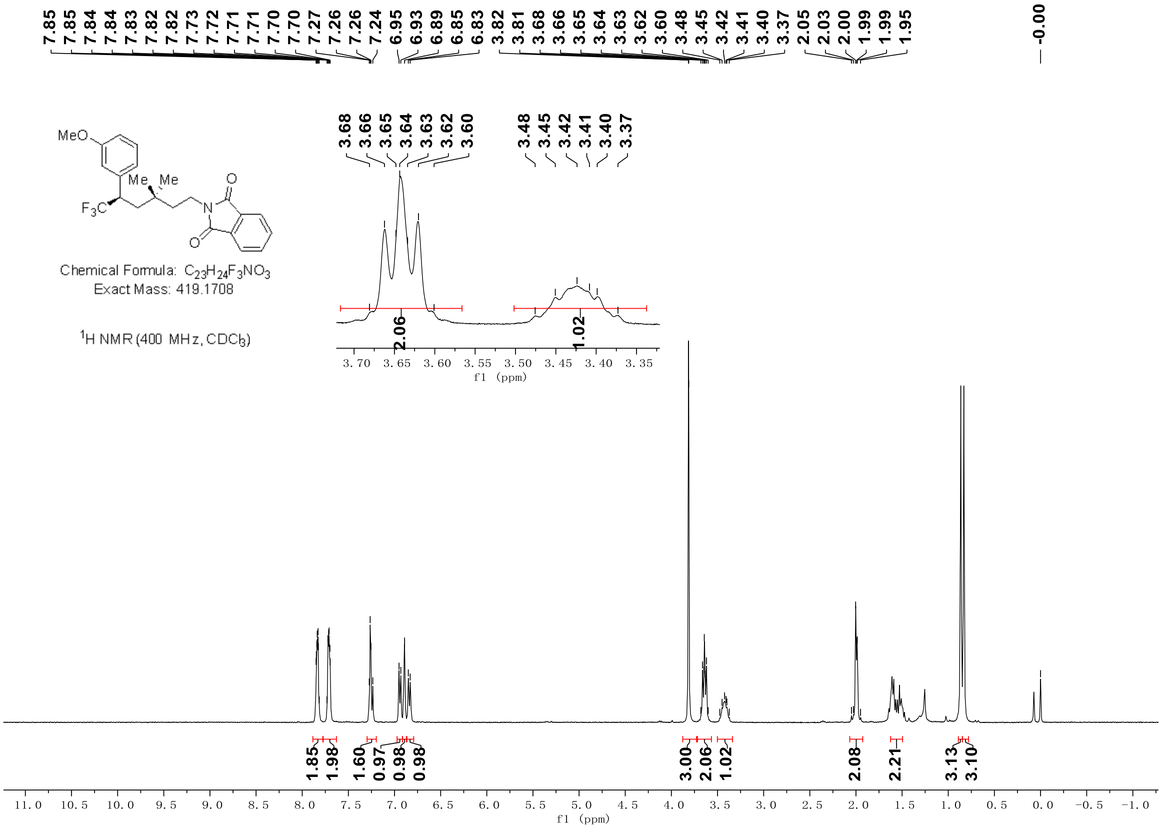

Supplementary Figure 117. ^{1}H NMR spectrum of compound 5c

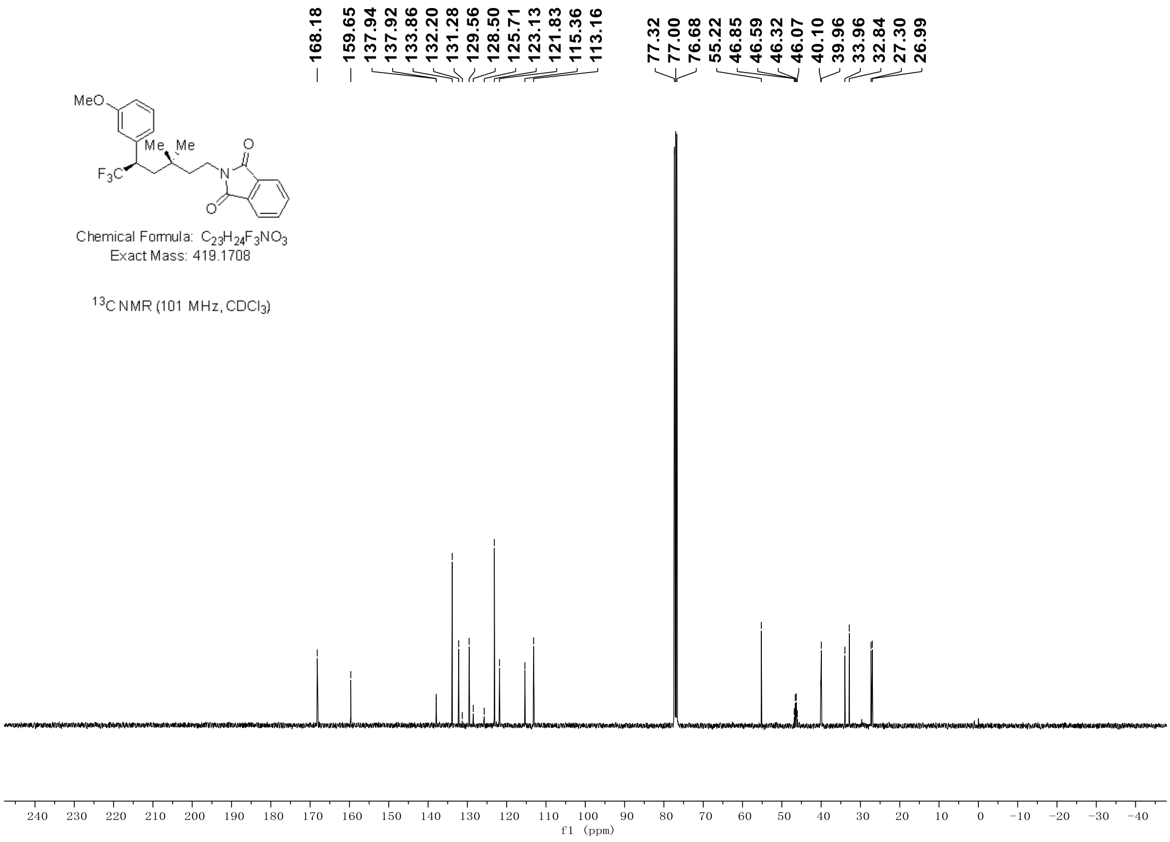

Supplementary Figure 118. ^{13}C NMR spectrum of compound 5c

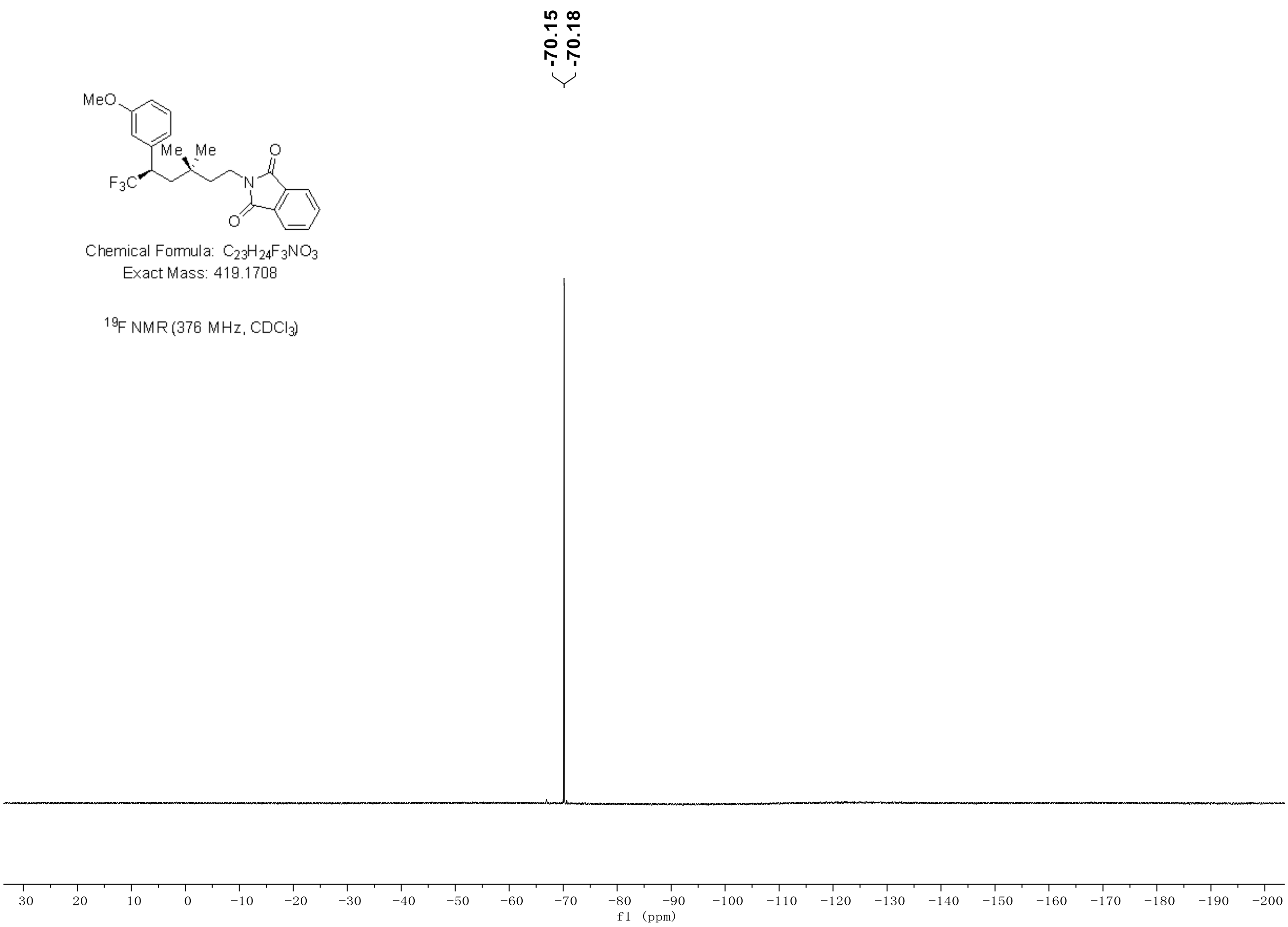

Supplementary Figure 119. ^{19}F NMR spectrum of compound 5c

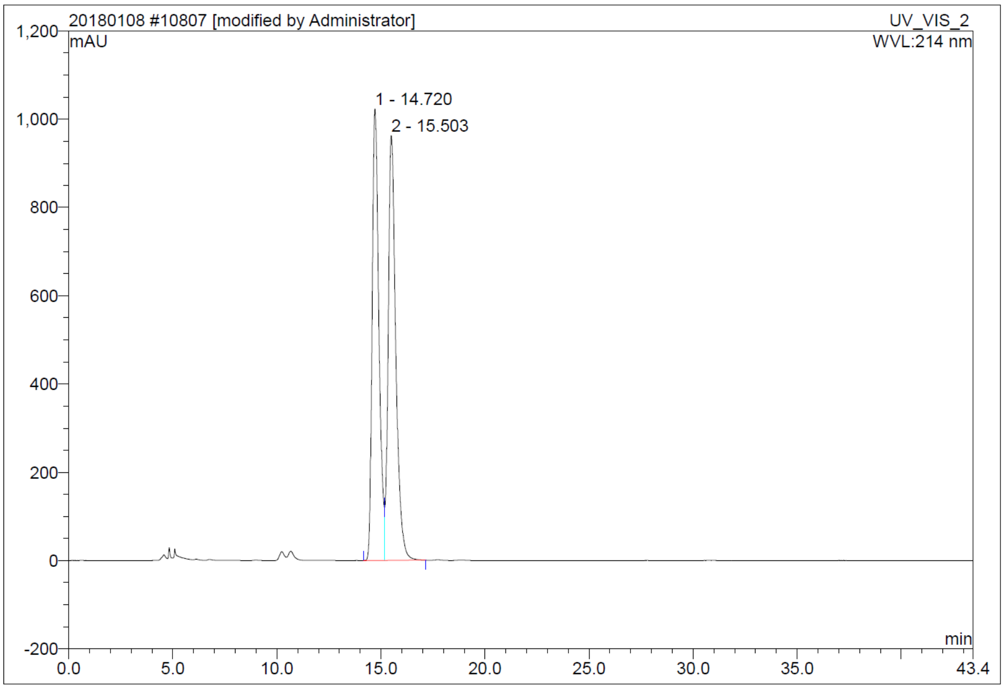

No.	Ret.Time min	Peak Name	Height mAU	Area mAU*min	Rel.Area %	Amount	Type
1	14.72	n.a.	1023.178	383.895	48.40	n.a.	BM
2	15.50	n.a.	962.943	409.200	51.60	n.a.	MB
Total:			1986.121	793.095	100.00	0.000	

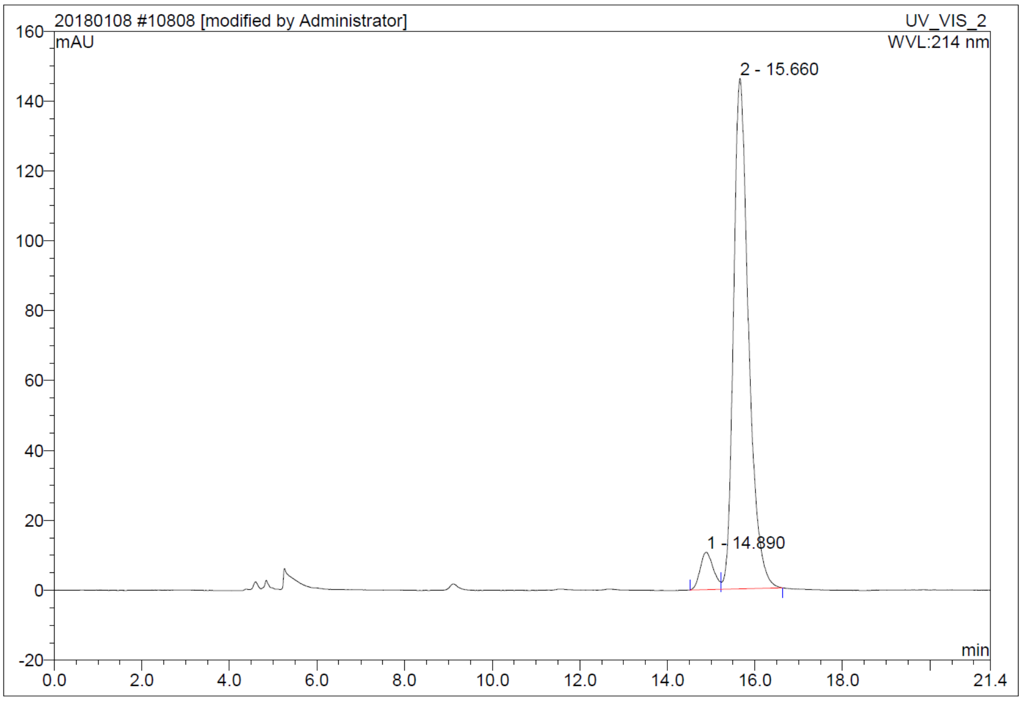

No.	Ret.Time min	Peak Name	Height mAU	Area mAU*min	Rel.Area %	Amount	Type
1	14.89	n.a.	10.685	3.725	6.00	n.a.	BM
2	15.66	n.a.	146.053	58.337	94.00	n.a.	MB
Total:			156.738	62.062	100.00	0.000	

Supplementary Figure 120. Chiral HPLC analysis of compound 5c

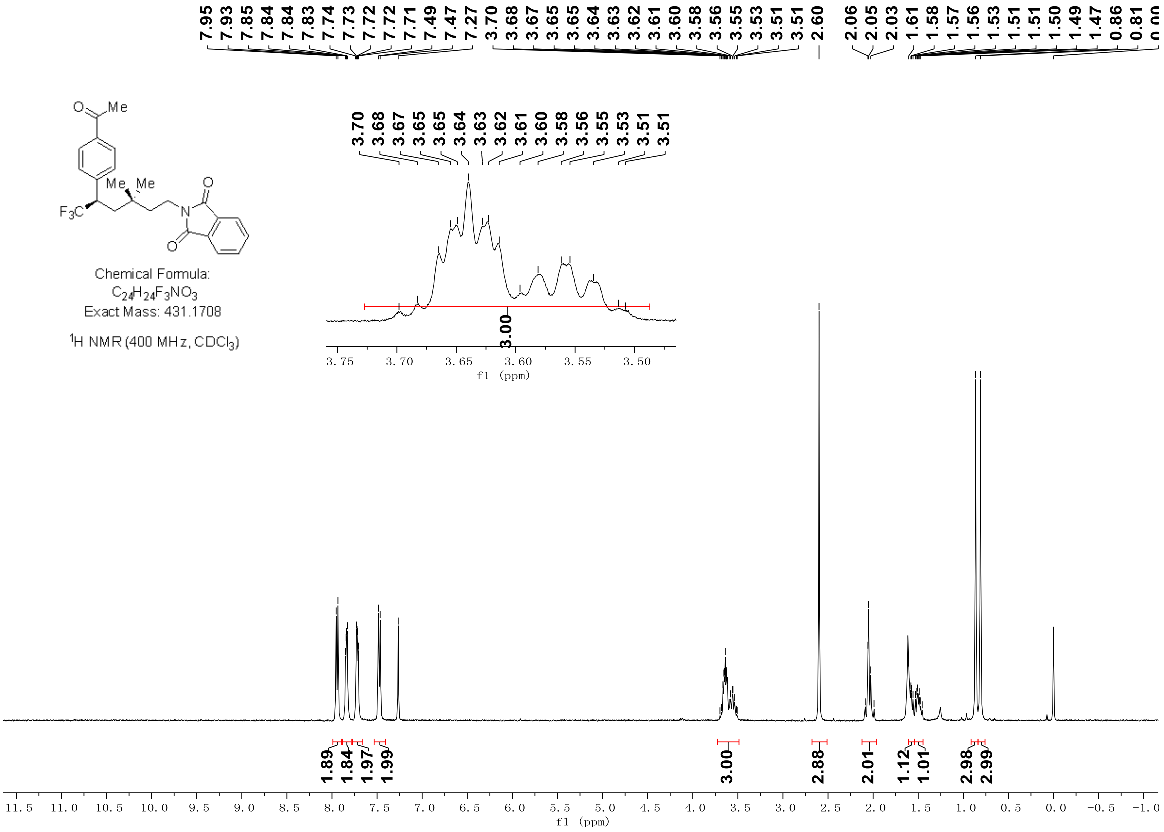

Supplementary Figure 121. ^{1}H NMR spectrum of compound 5d

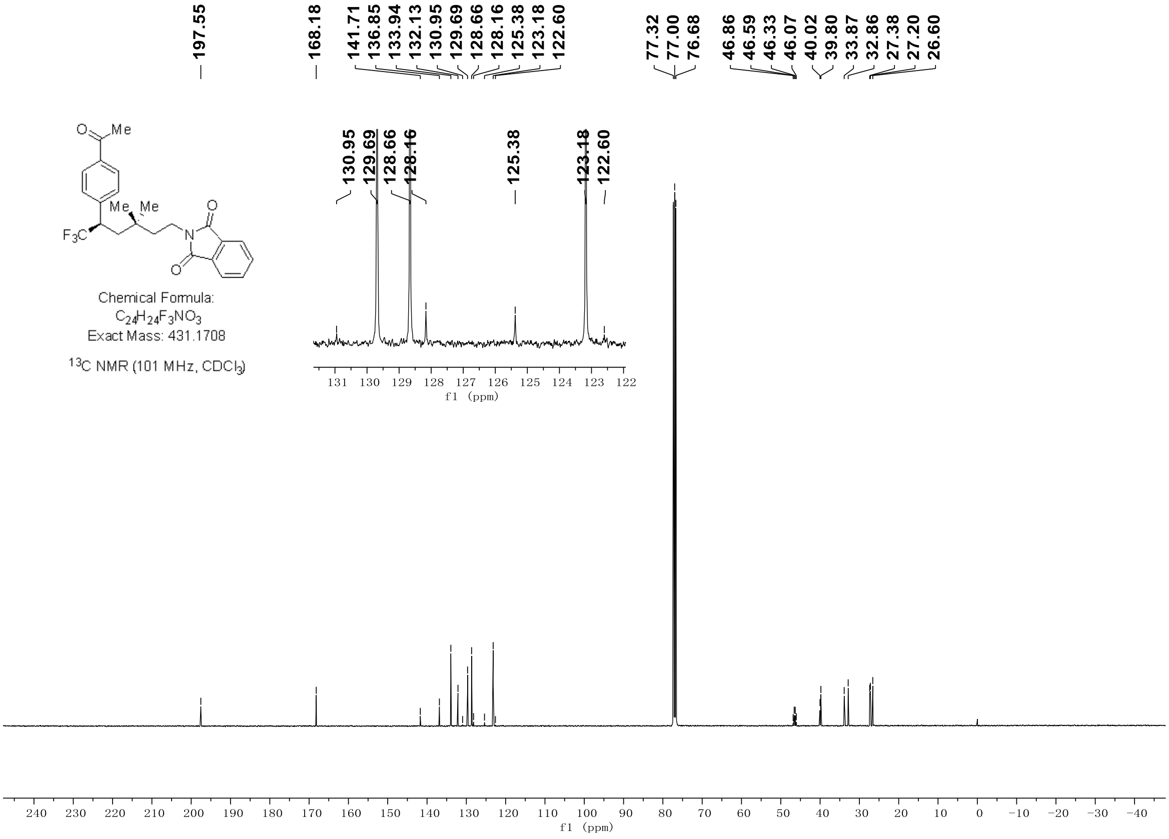

Supplementary Figure 122. ^{13}C NMR spectrum of compound 5d

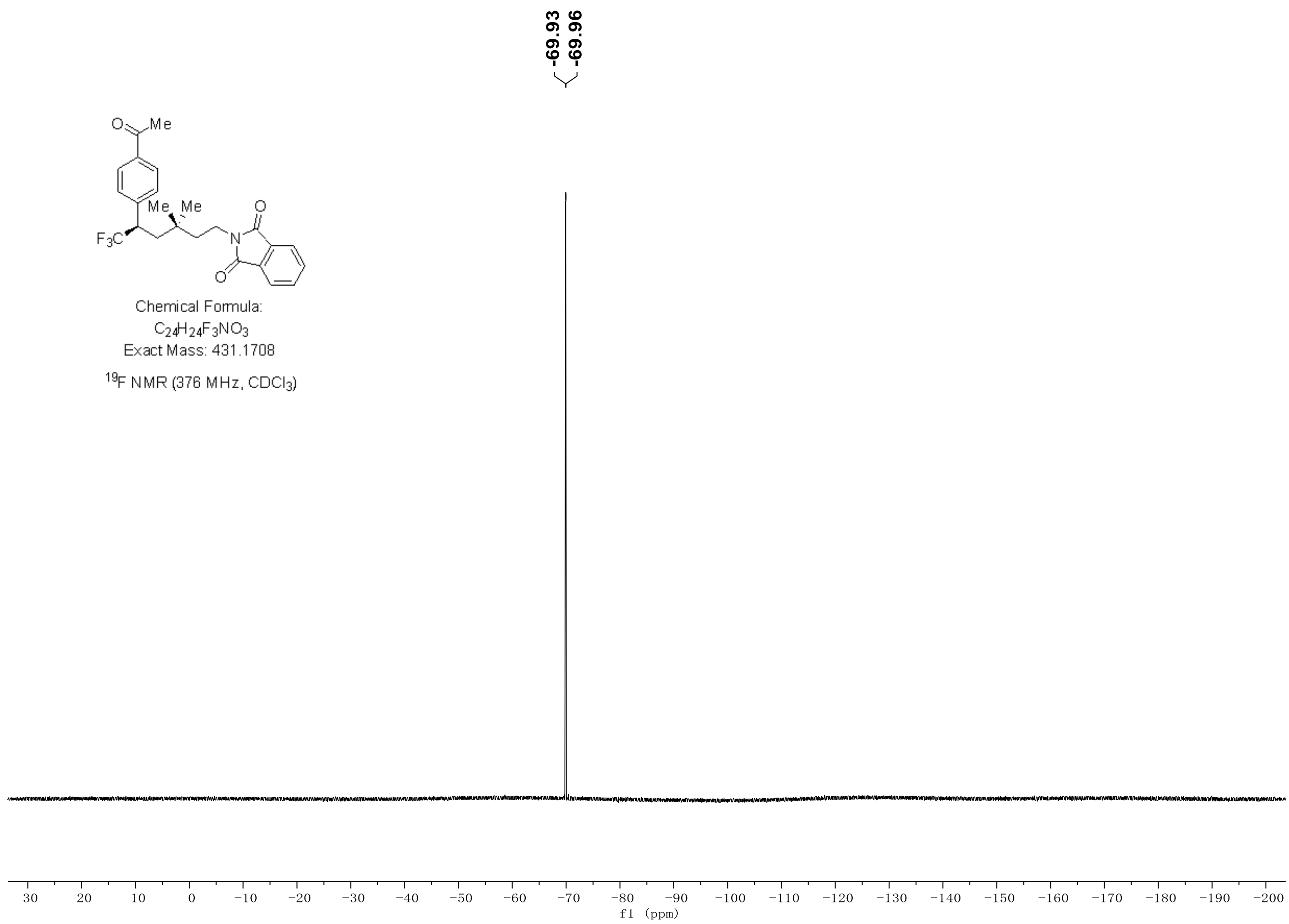

Supplementary Figure 123. ^{19}F NMR spectrum of compound 5d

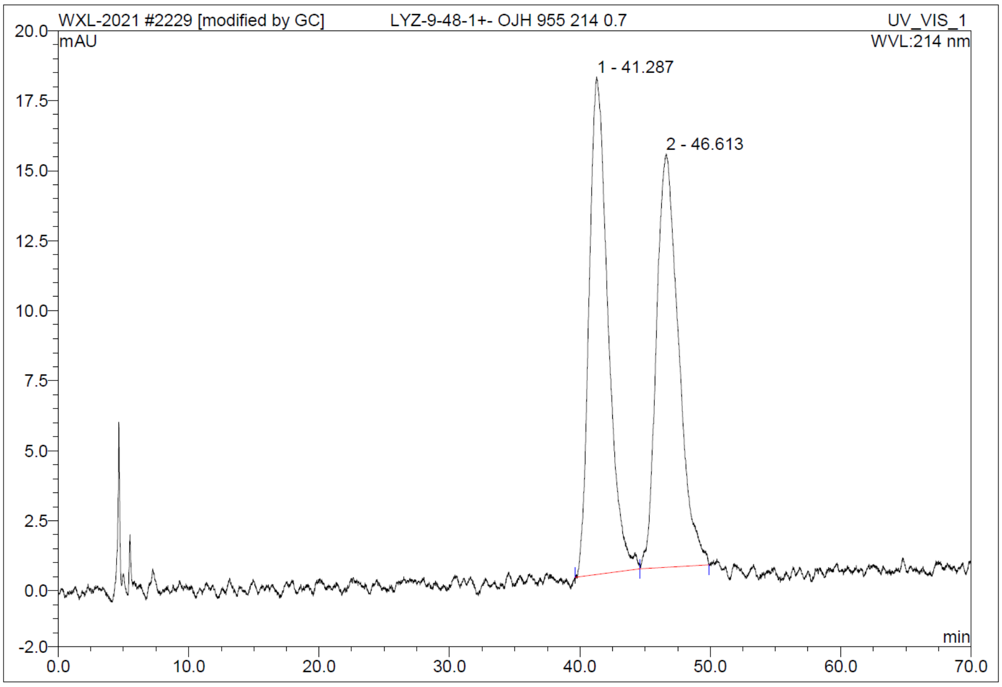

No.	Ret.Time min	Peak Name	Height mAU	Area mAU*min	Rel.Area %	Amount	Type
1	41.29	n.a.	17.769	29.786	50.57	n.a.	BMb*
2	46.61	n.a.	14.778	29.109	49.43	n.a.	bMB*
Total:			32.547	58.895	100.00	0.000	

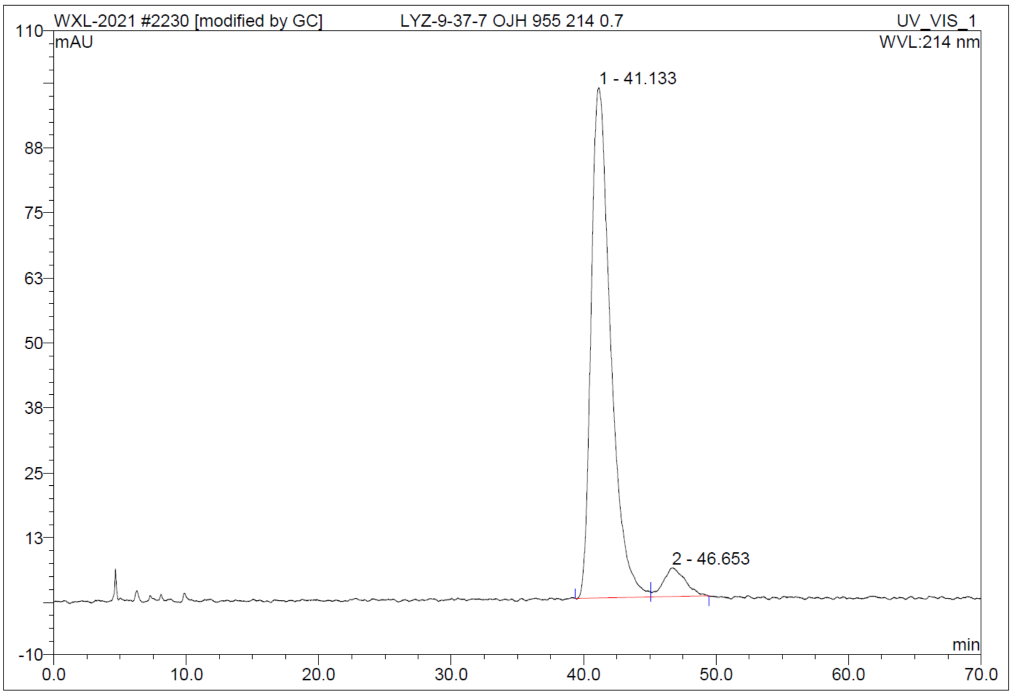

No.	Ret.Time min	Peak Name	Height mAU	Area mAU*min	Rel.Area %	Amount	Type
1	41.13	n.a.	98.276	168.263	94.10	n.a.	BM *
2	46.65	n.a.	5.539	10.558	5.90	n.a.	MB*
Total:			103.815	178.821	100.00	0.000	

Supplementary Figure 124. Chiral HPLC analysis of compound 5d

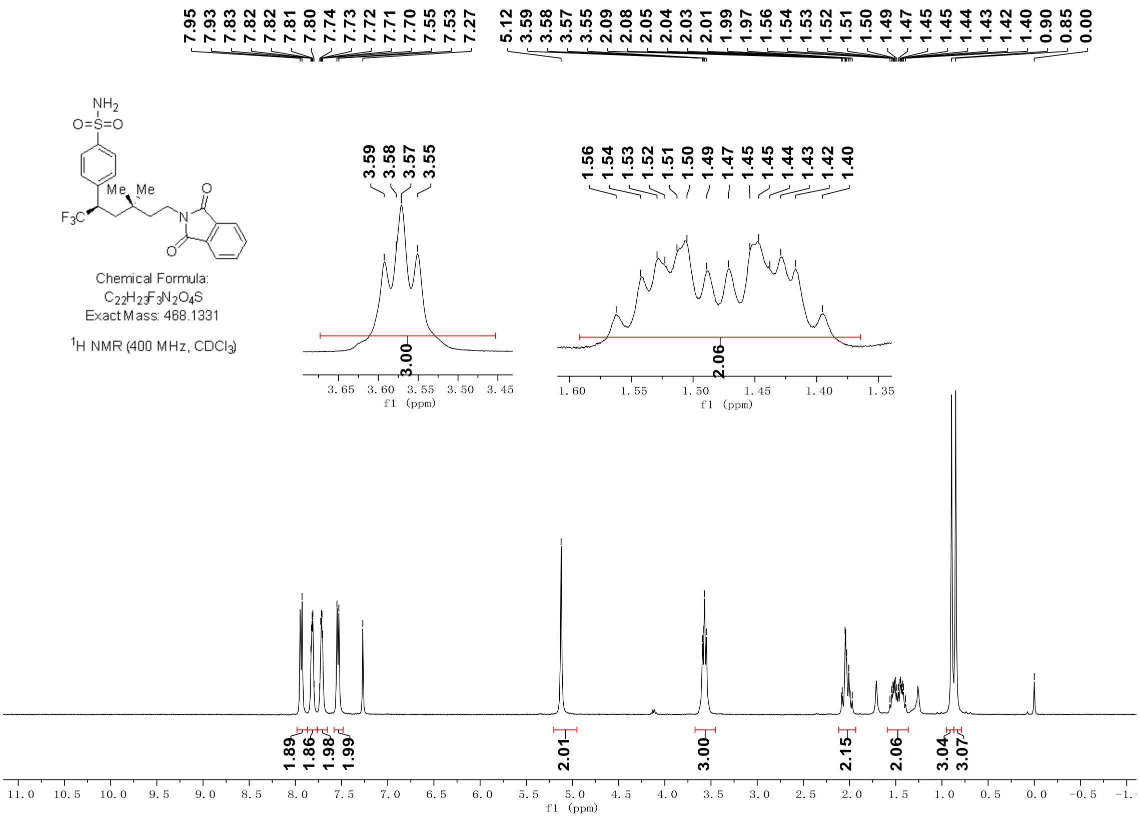

Supplementary Figure 125. ^{1}H NMR spectrum of compound 5e

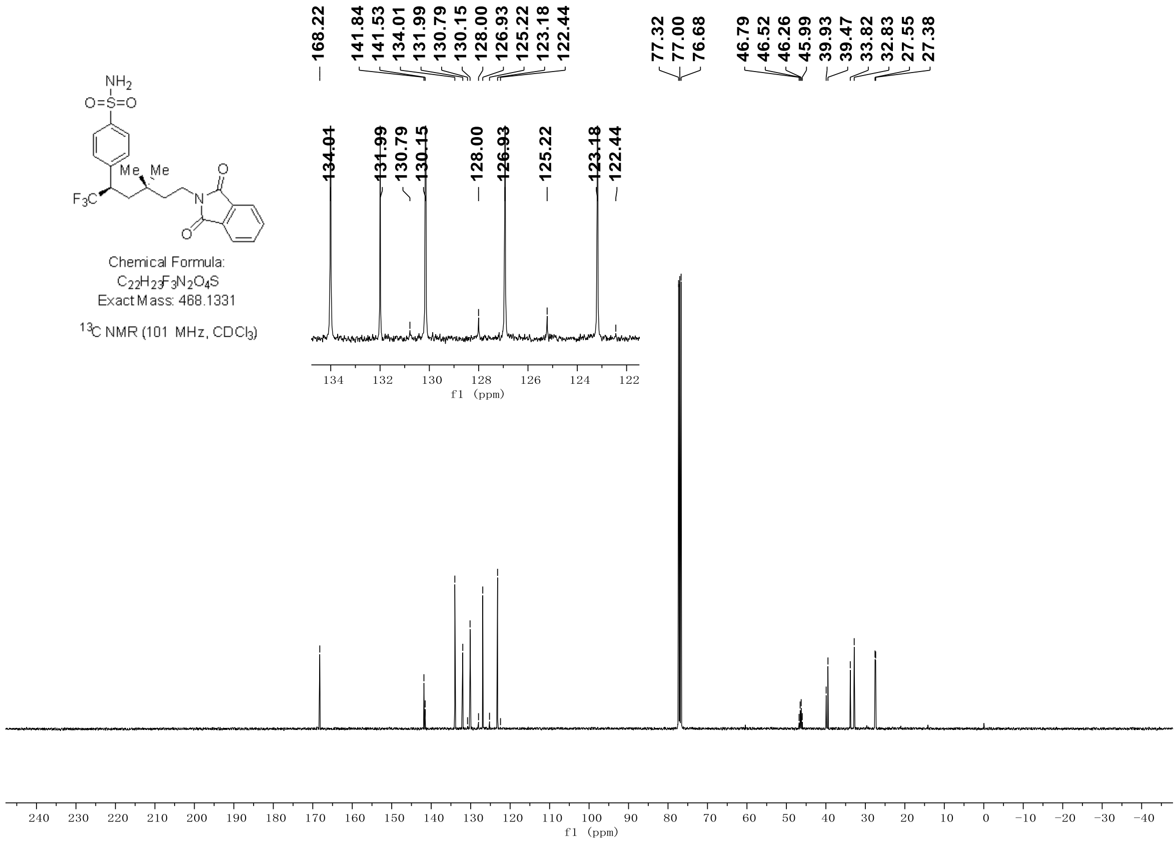

Supplementary Figure 126. ^{13}C NMR spectrum of compound 5e

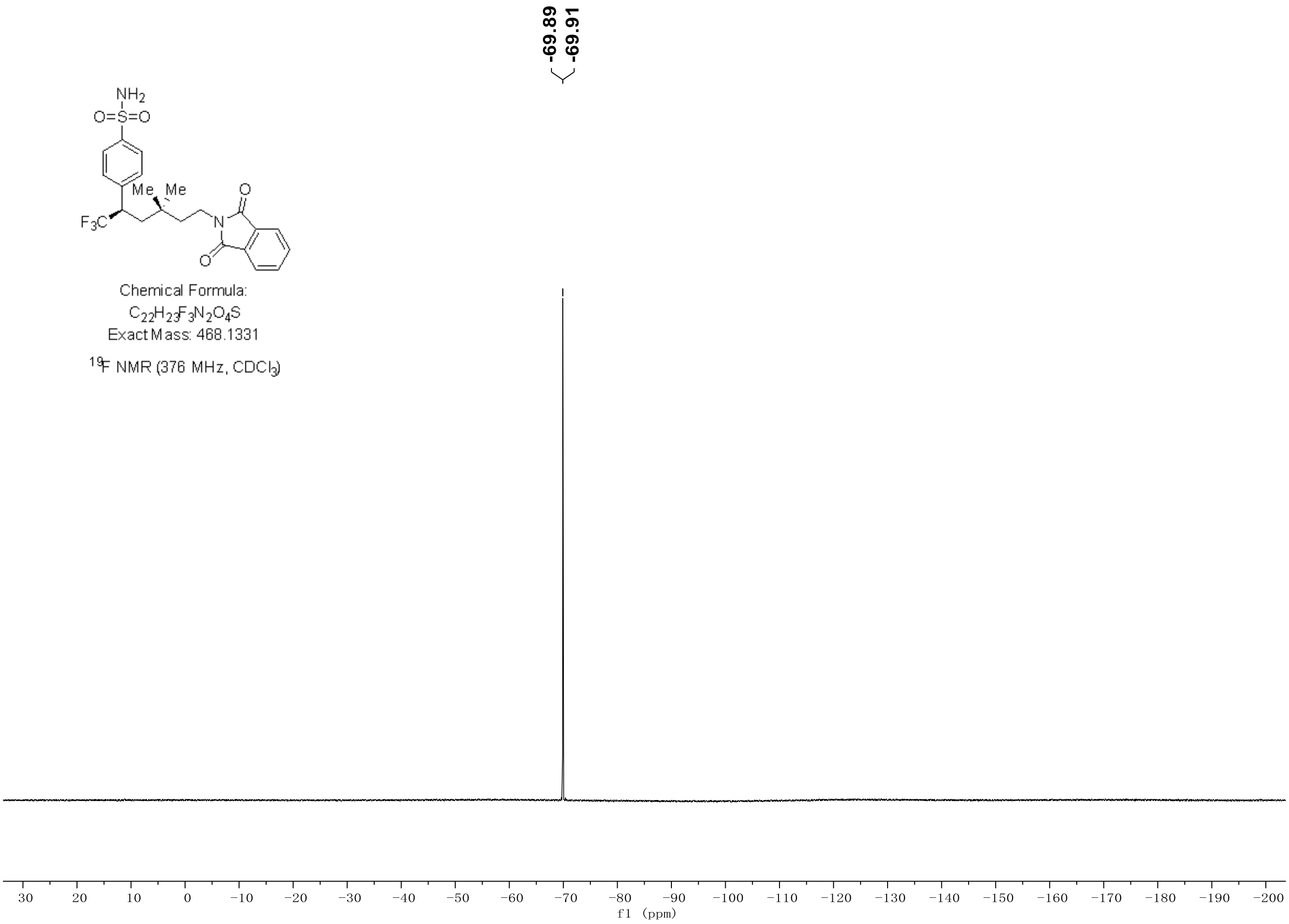

Supplementary Figure 127. ^{19}F NMR spectrum of compound 5e

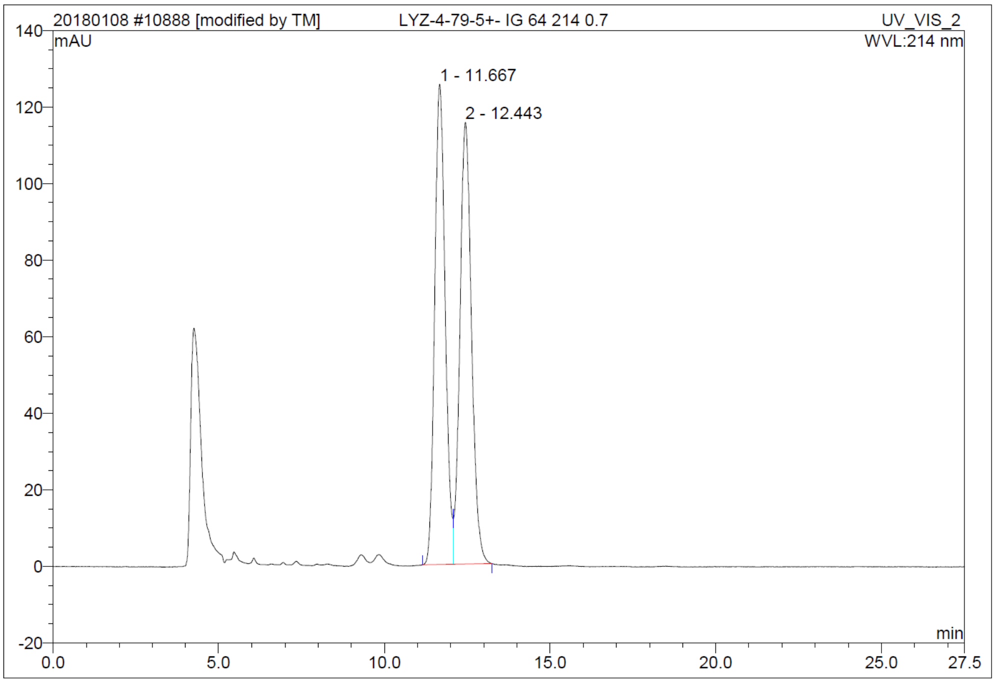

No.	Ret.Time min	Peak Name	Height mAU	Area mAU*min	Rel.Area %	Amount	Type
1	11.67	n.a.	125.415	45.808	49.39	n.a.	BM
2	12.44	n.a.	115.412	46.938	50.61	n.a.	MB
Total:			240.827	92.746	100.00	0.000	

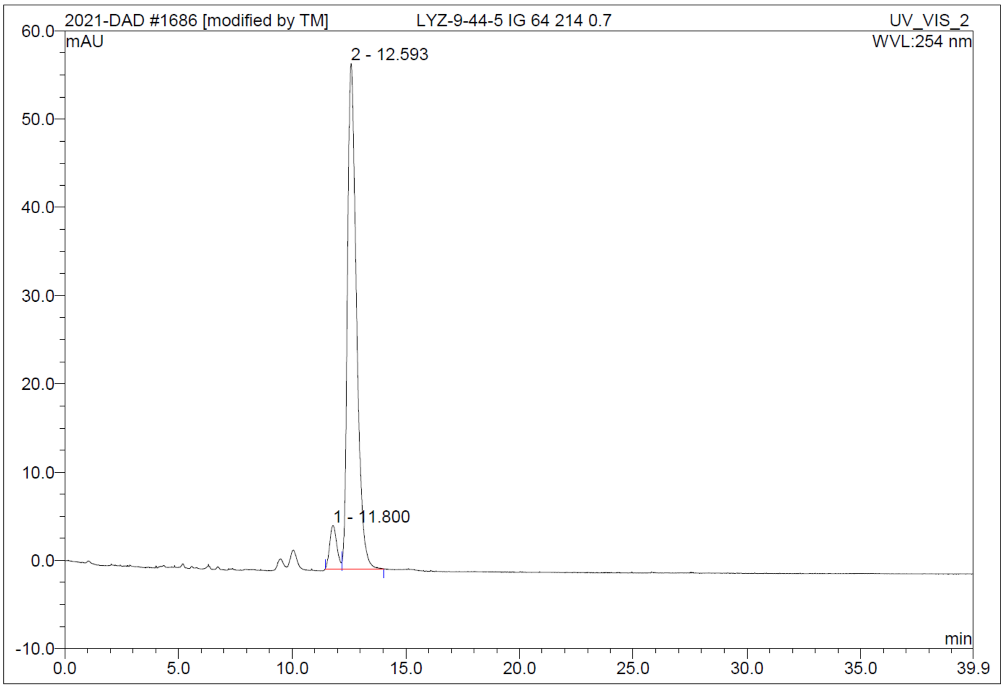

No.	Ret.Time min	Peak Name	Height mAU	Area mAU*min	Rel.Area %	Amount	Type
1	11.80	n.a.	4.890	1.805	6.42	n.a.	BM *
2	12.59	n.a.	57.264	26.289	93.58	n.a.	MB*
Total:			62.154	28.094	100.00	0.000	

Supplementary Figure 128. Chiral HPLC analysis of compound 5e

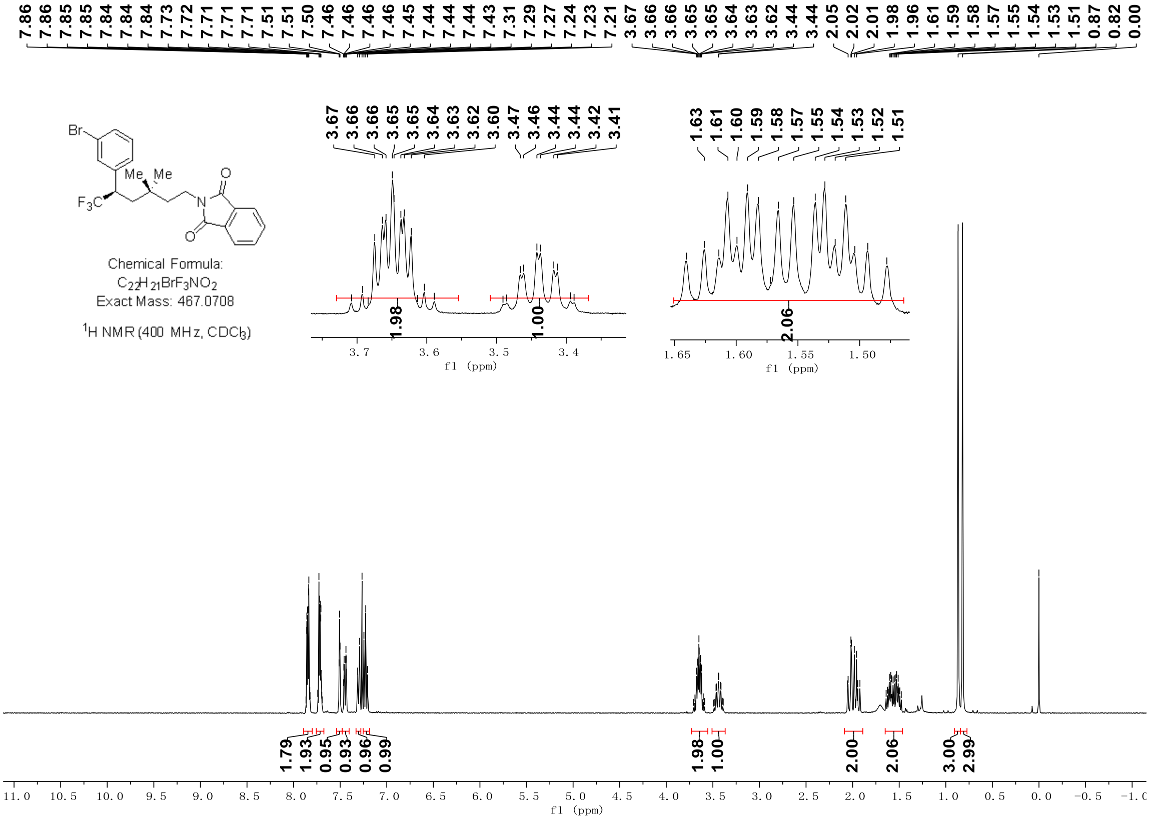

Supplementary Figure 129. ^{1}**H NMR spectrum of compound 5f**

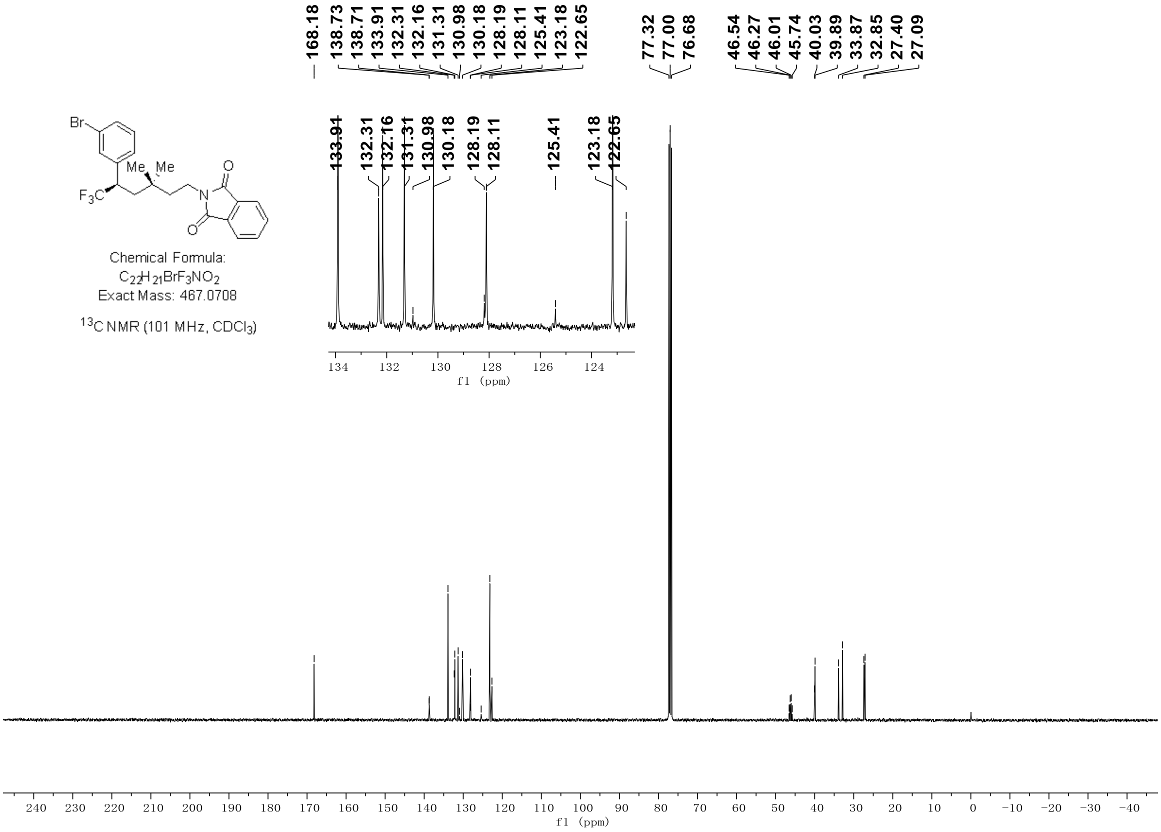

Supplementary Figure 130. ^{13}**C NMR spectrum of compound 5f**

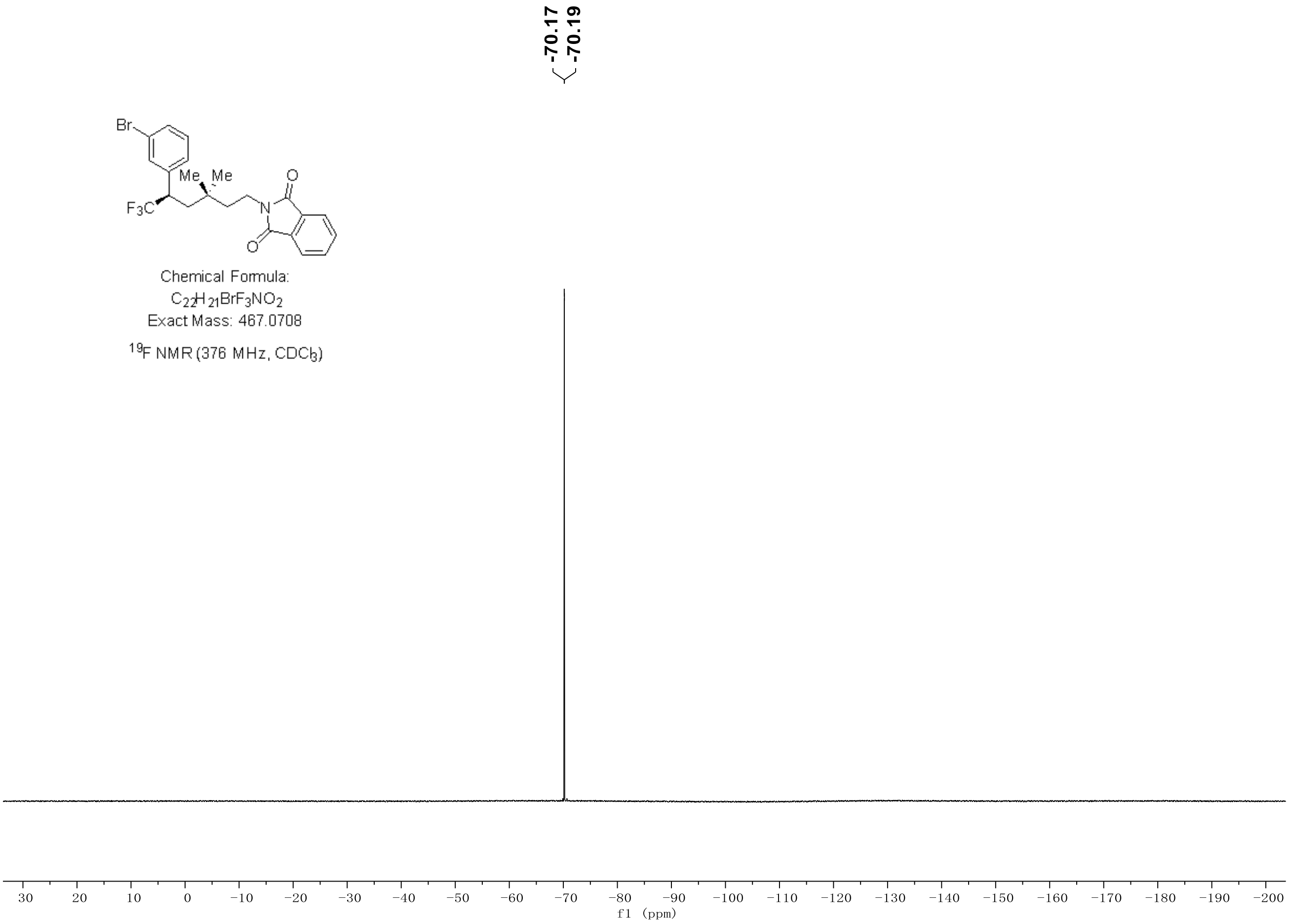

Supplementary Figure 131. ^{19}F NMR spectrum of compound 5f

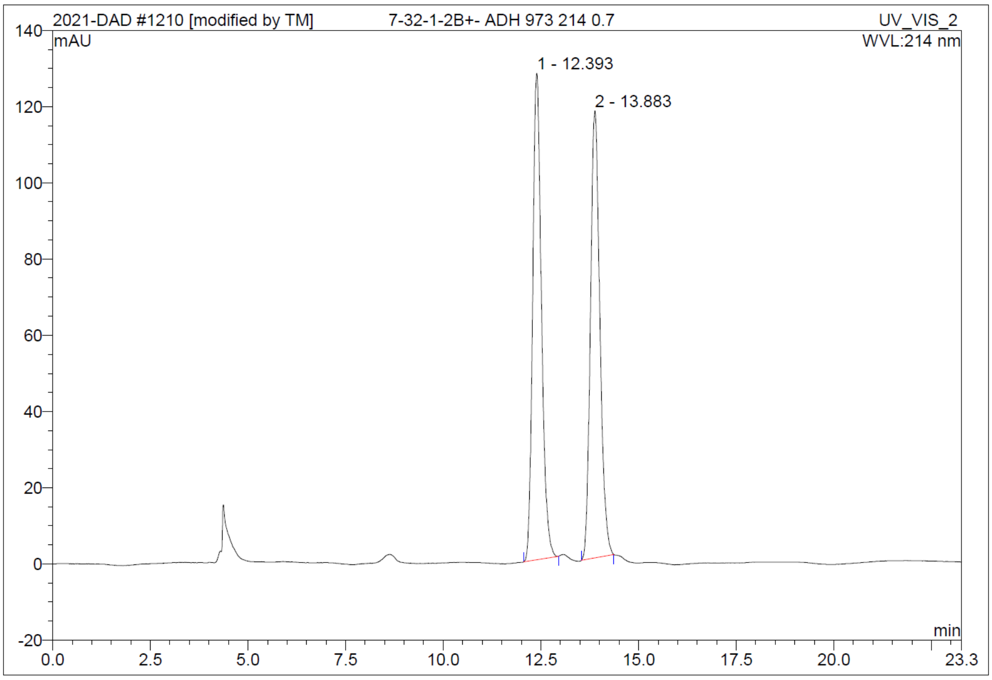

No.	Ret.Time min	Peak Name	Height mAU	Area mAU*min	Rel.Area %	Amount	Type
1	12.39	n.a.	127.649	33.185	50.27	n.a.	BMB*
2	13.88	n.a.	117.294	32.822	49.73	n.a.	BMB*
Total:			244.943	66.007	100.00	0.000	

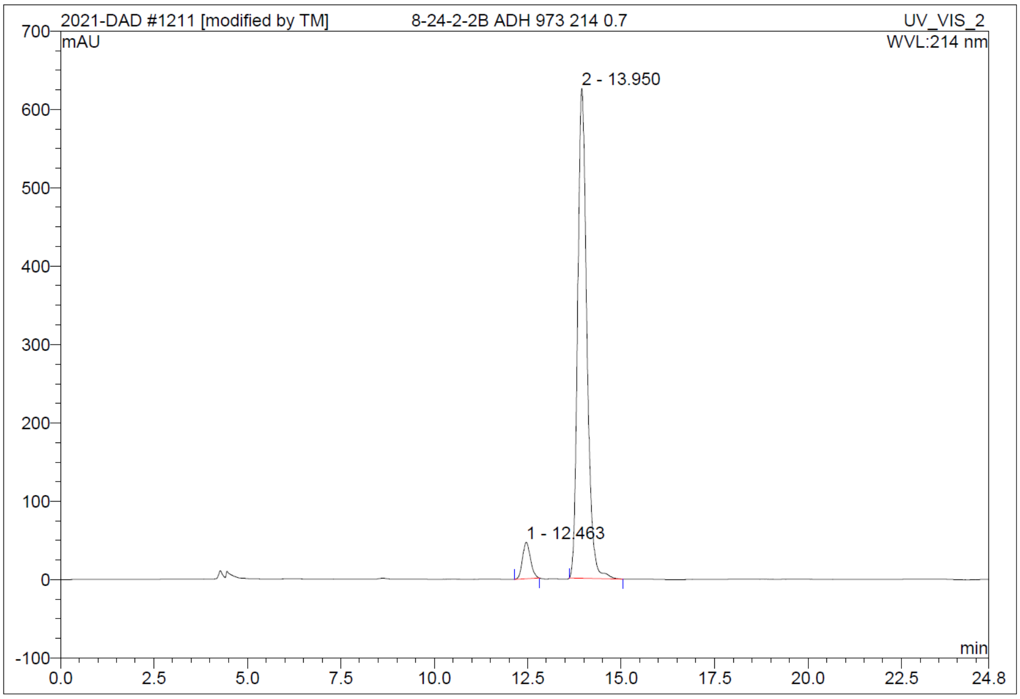

No.	Ret.Time min	Peak Name	Height mAU	Area mAU*min	Rel.Area %	Amount	Type
1	12.46	n.a.	46.484	11.496	6.06	n.a.	BMB*
2	13.95	n.a.	624.954	178.354	93.94	n.a.	BMB*
Total:			671.438	189.850	100.00	0.000	

Supplementary Figure 132. Chiral HPLC analysis of compound 5f

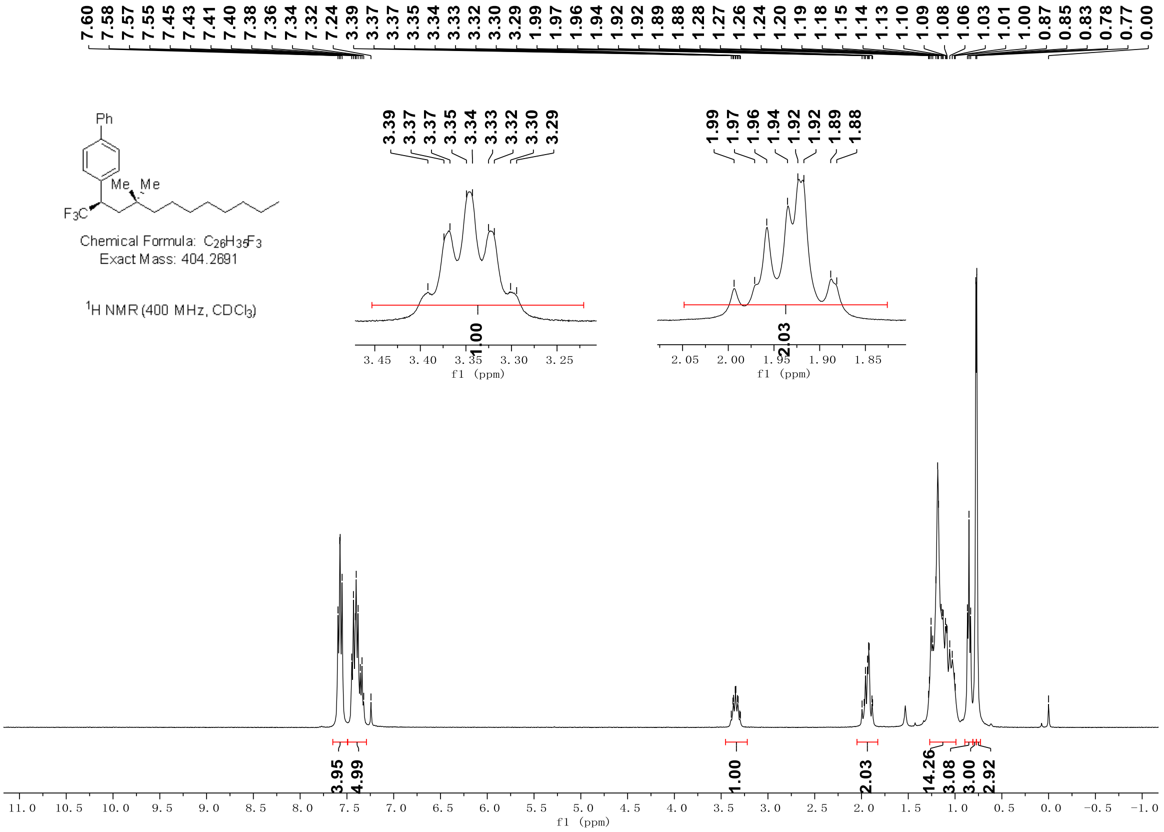

Supplementary Figure 133. ^{1}H NMR spectrum of compound 5g

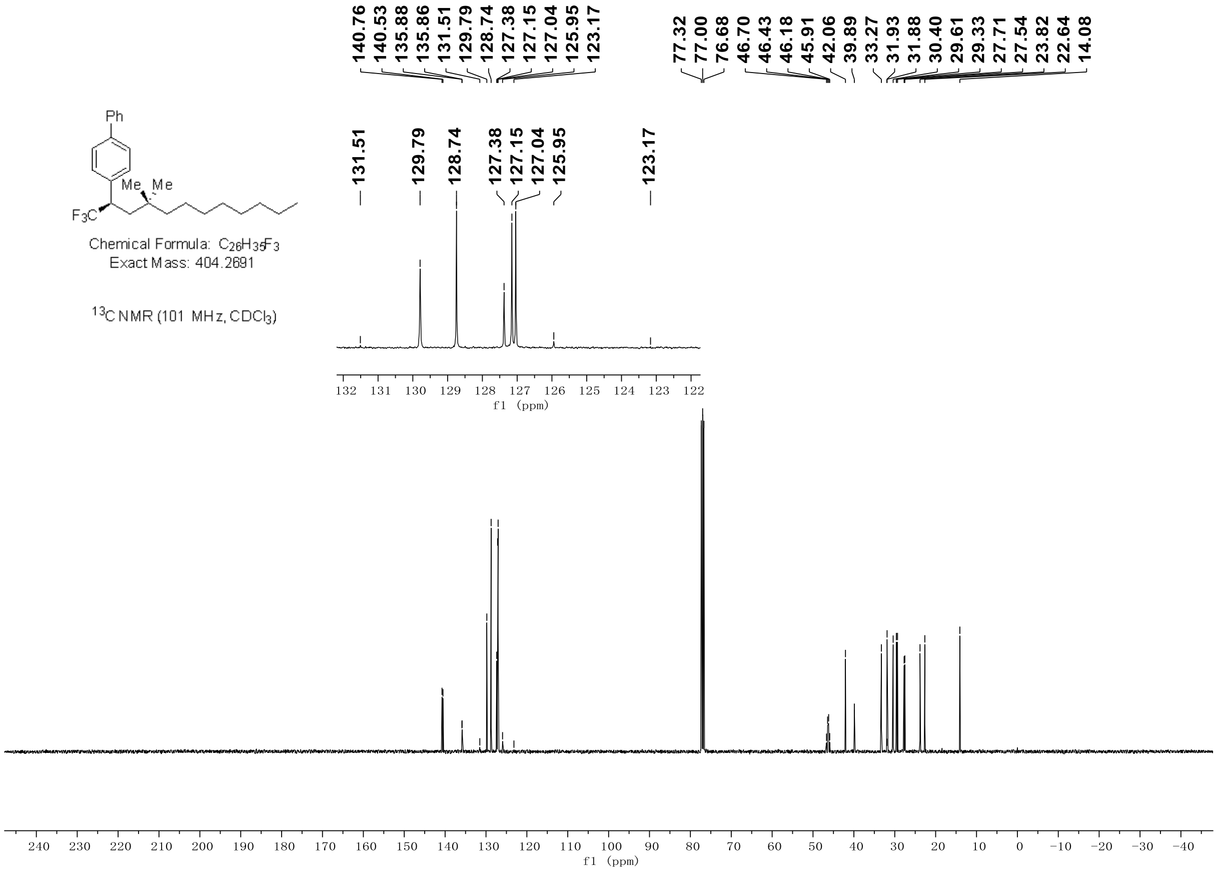

Supplementary Figure 134. ^{13}C NMR spectrum of compound 5g

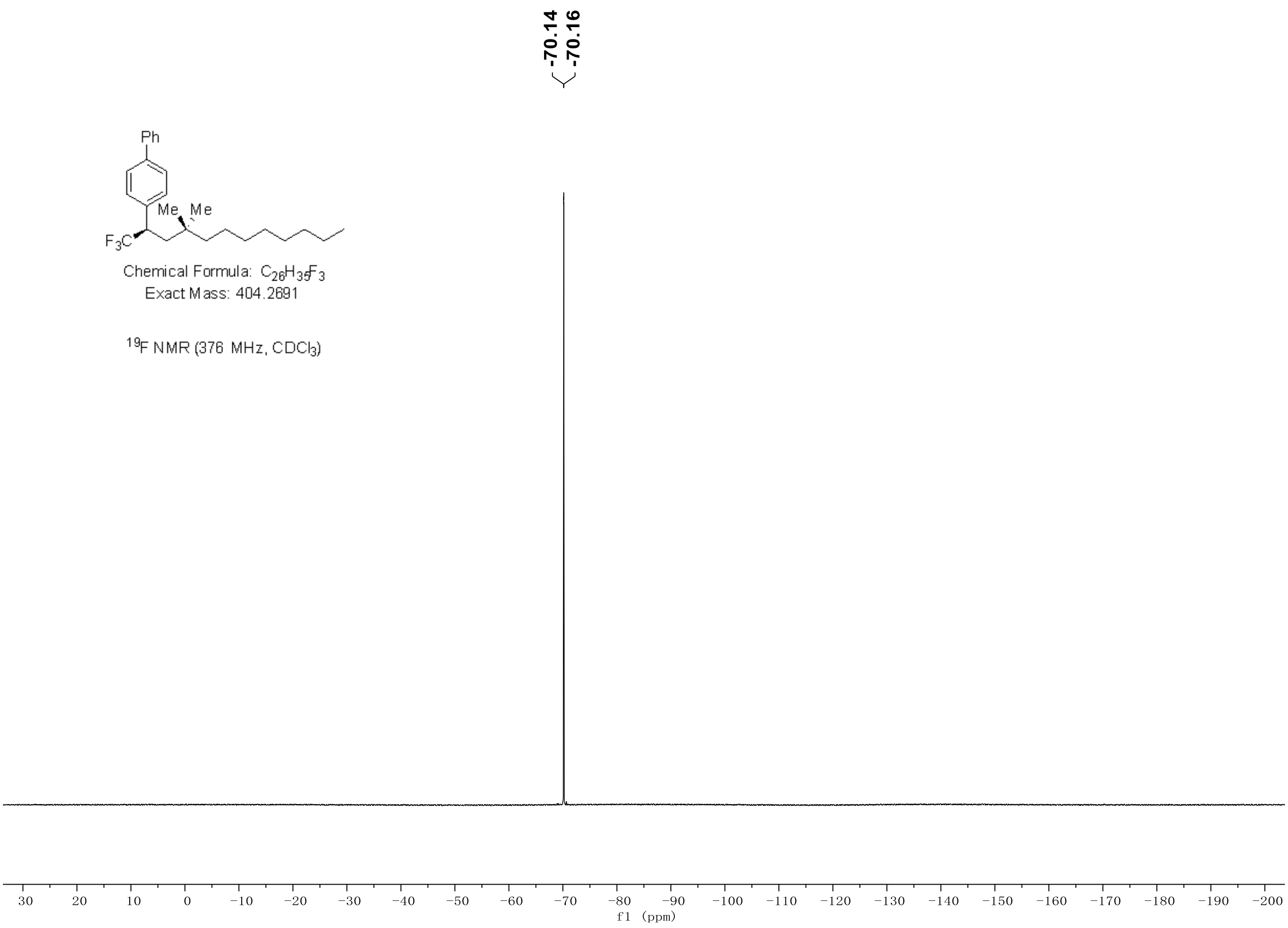

Supplementary Figure 135. ^{19}F NMR spectrum of compound 5g

Chromatogram

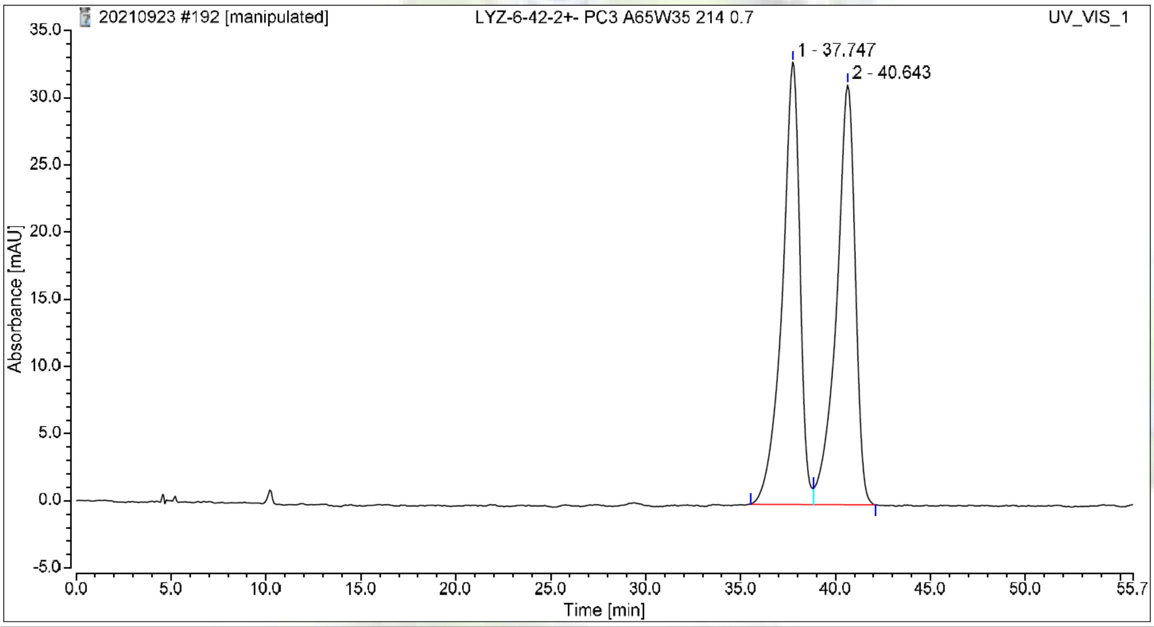

Integration Results

No.	Retention Time min	Area mAU*min	Height mAU	Relative Area %	Resolution (EP)	Asymmetry (EP)	K'	Plates (EP)
n.a.	37.747	35.7478	32.9644	50.025	1.77	0.79	n.a.	8949
n.a.	40.643	35.7122	31.3004	49.975	n.a.	0.78	n.a.	9305
**Total:**		**71.460**	**1401.998**	**100.000**				

Chromatogram

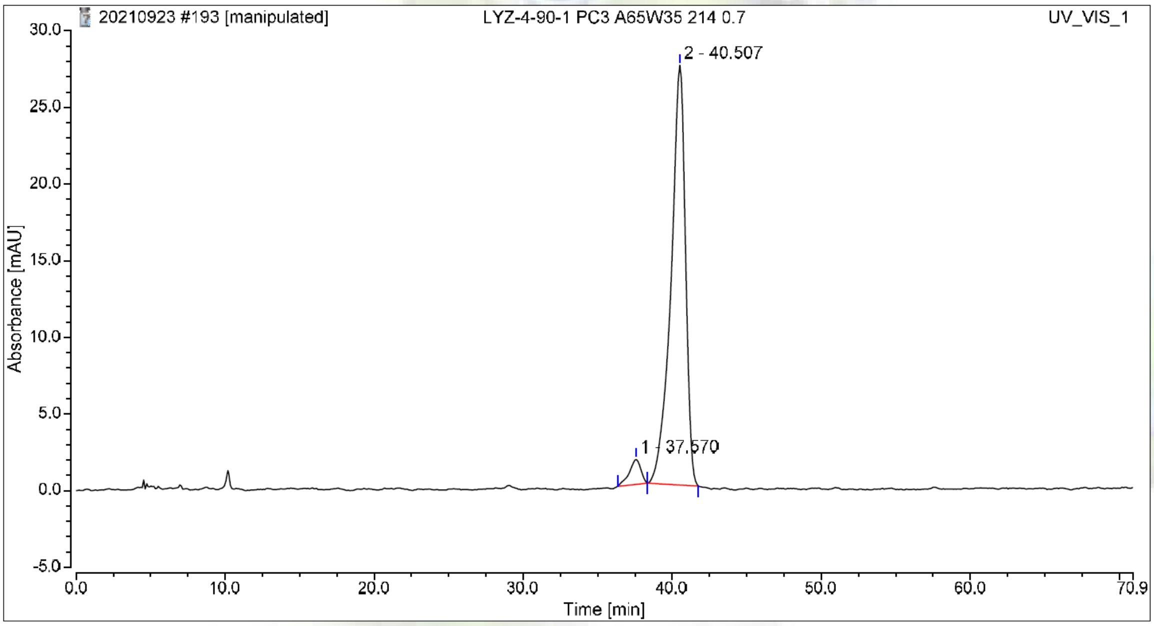

Integration Results

No.	Retention Time min	Area mAU*min	Height mAU	Relative Area %	Resolution (EP)	Asymmetry (EP)	K'	Plates (EP)
n.a.	37.570	1.4896	1.6481	4.601	1.91	0.79	n.a.	11475
n.a.	40.507	30.8845	27.3904	95.399	n.a.	0.78	n.a.	9235
**Total:**		**32.374**	**1401.998**	**100.000**				

Supplementary Figure 136. Chiral HPLC analysis of compound 5g

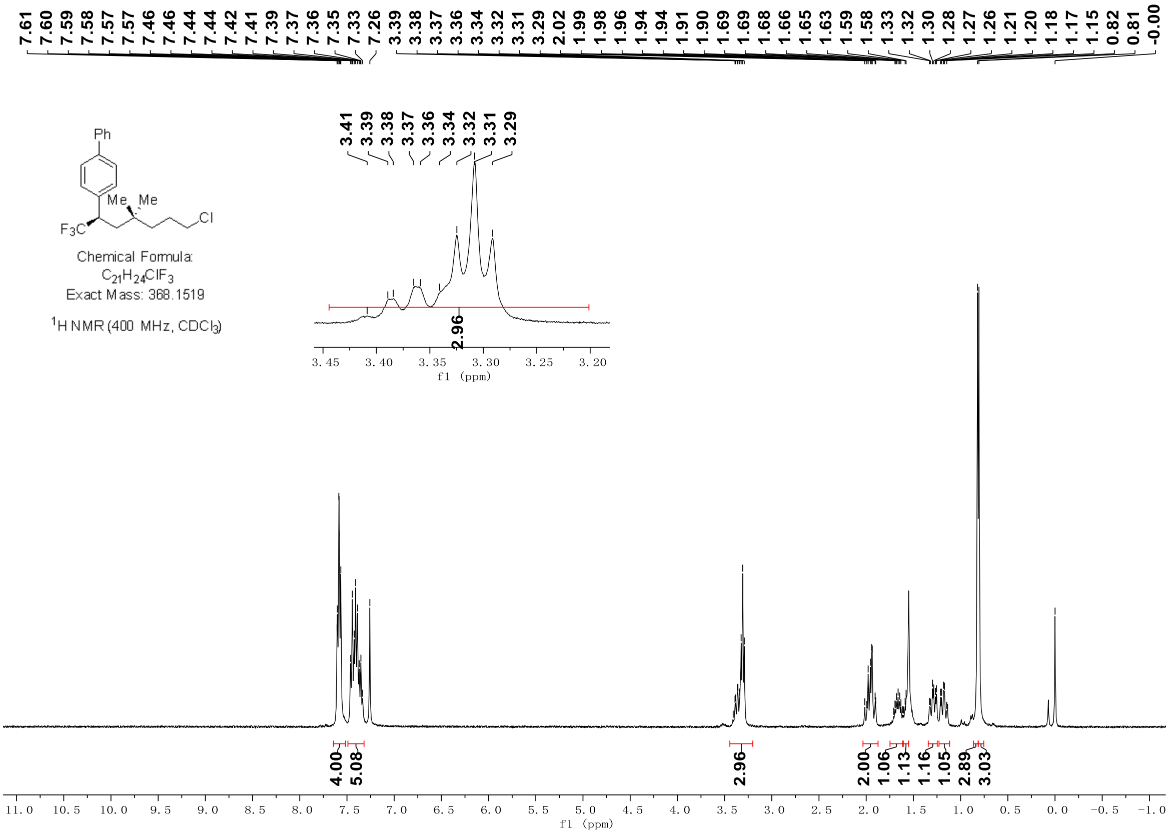

Supplementary Figure 137. ^{1}H NMR spectrum of compound 5h

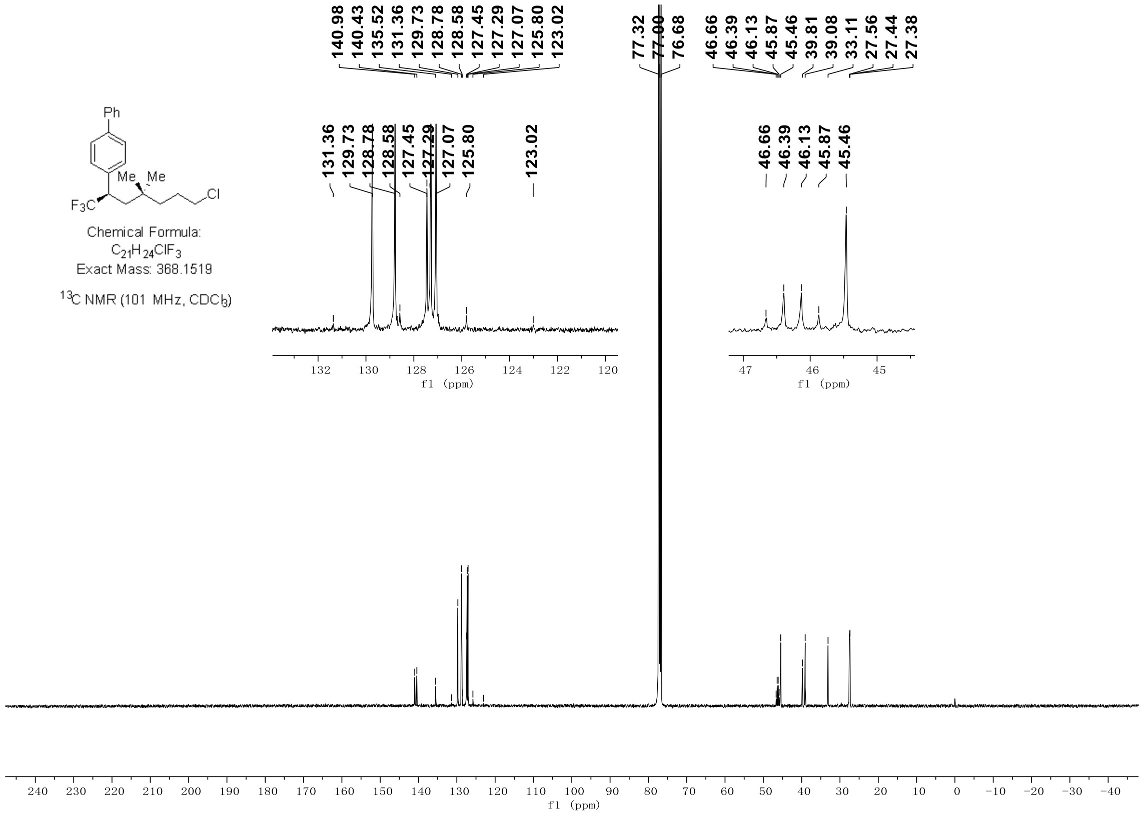

Supplementary Figure 138. ^{13}C NMR spectrum of compound 5h

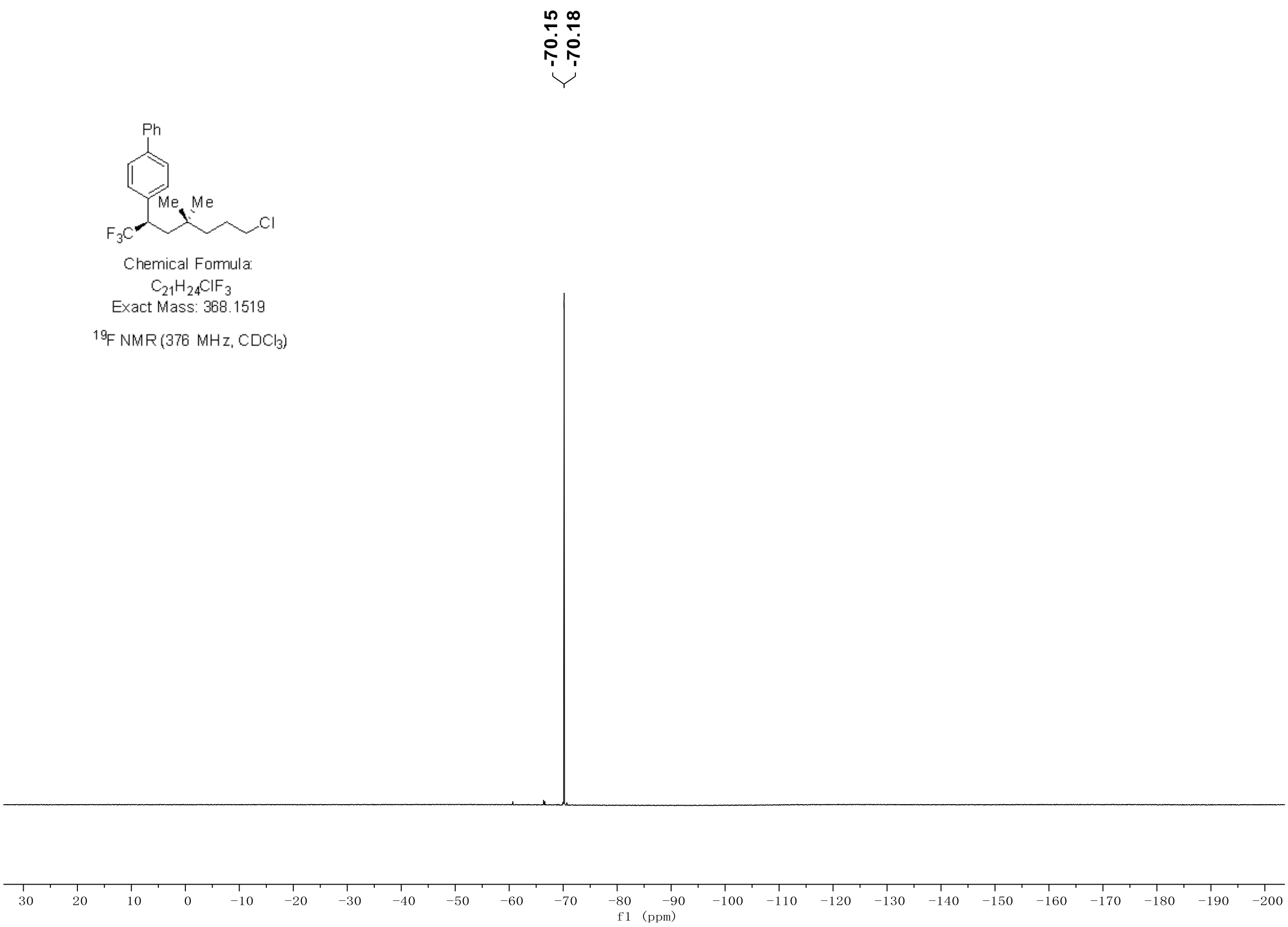

Supplementary Figure 139. ^{19}F NMR spectrum of compound 5h

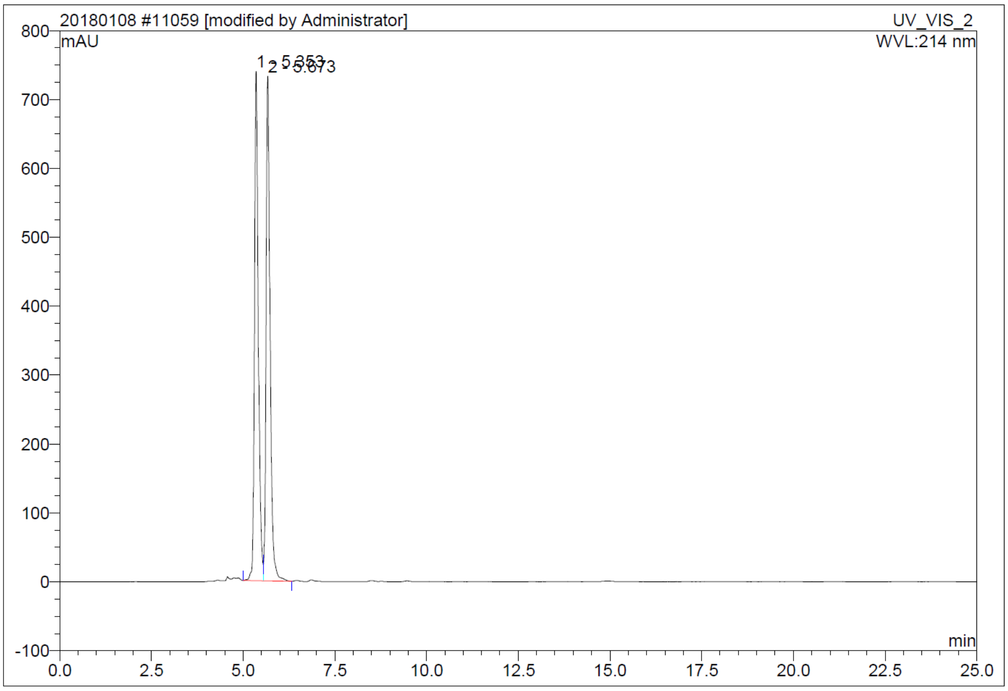

No.	Ret.Time min	Peak Name	Height mAU	Area mAU*min	Rel.Area %	Amount	Type
1	5.35	n.a.	739.550	91.118	49.55	n.a.	BM
2	5.67	n.a.	732.919	92.777	50.45	n.a.	MB
Total:			1472.468	183.895	100.00	0.000	

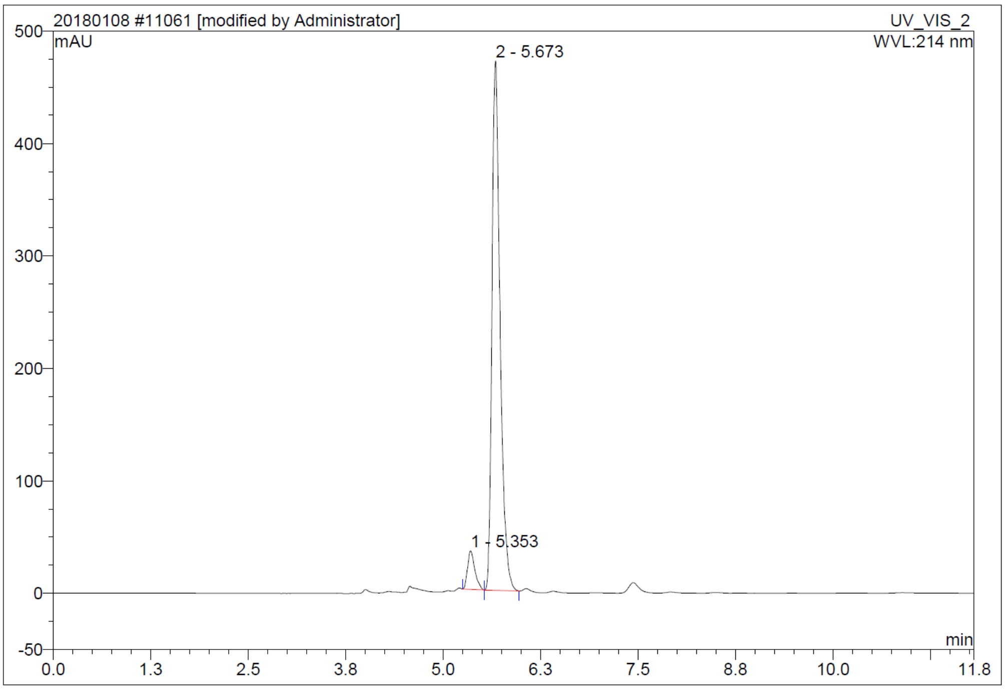

No.	Ret.Time min	Peak Name	Height mAU	Area mAU*min	Rel.Area %	Amount	Type
1	5.35	n.a.	34.159	3.707	6.16	n.a.	BMB
2	5.67	n.a.	470.595	56.523	93.84	n.a.	BMB
Total:			504.755	60.230	100.00	0.000	

Supplementary Figure 140. Chiral HPLC analysis of compound 5h

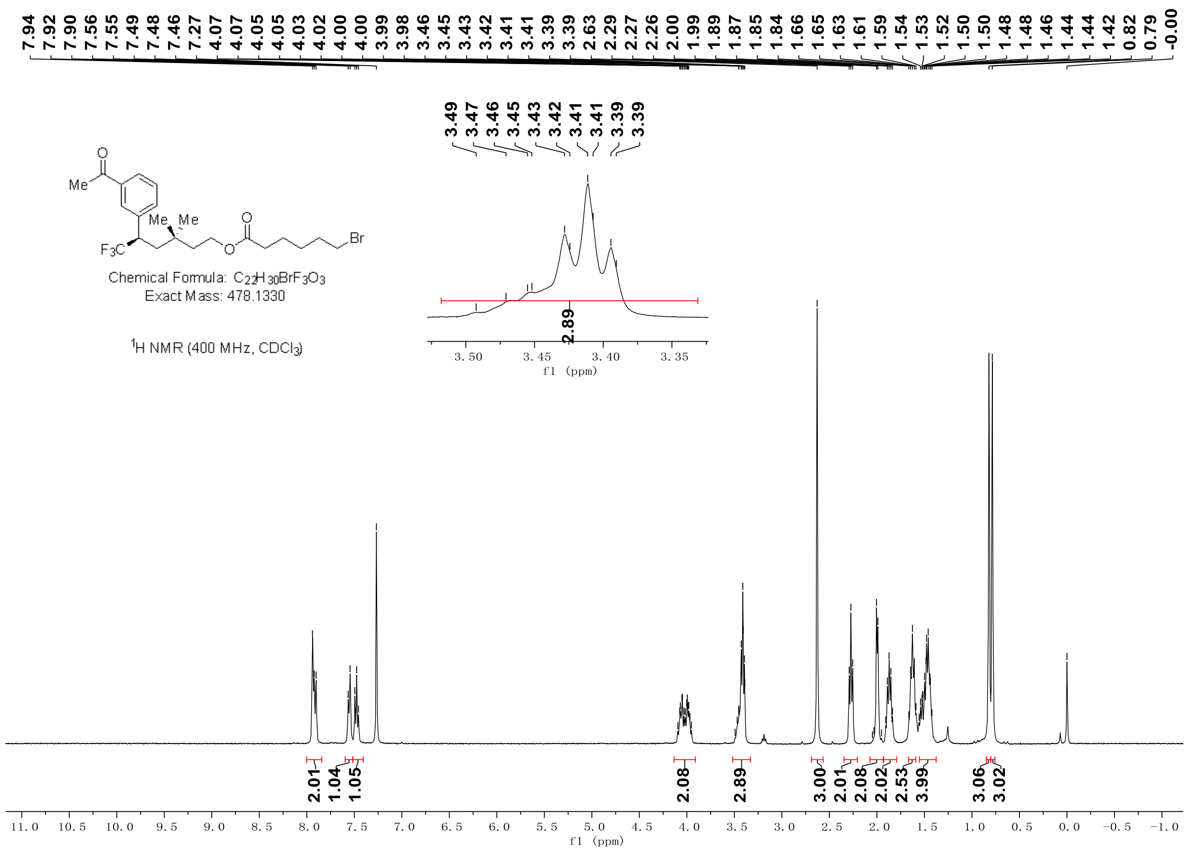

Supplementary Figure 141. ^{1}H NMR spectrum of compound 5i

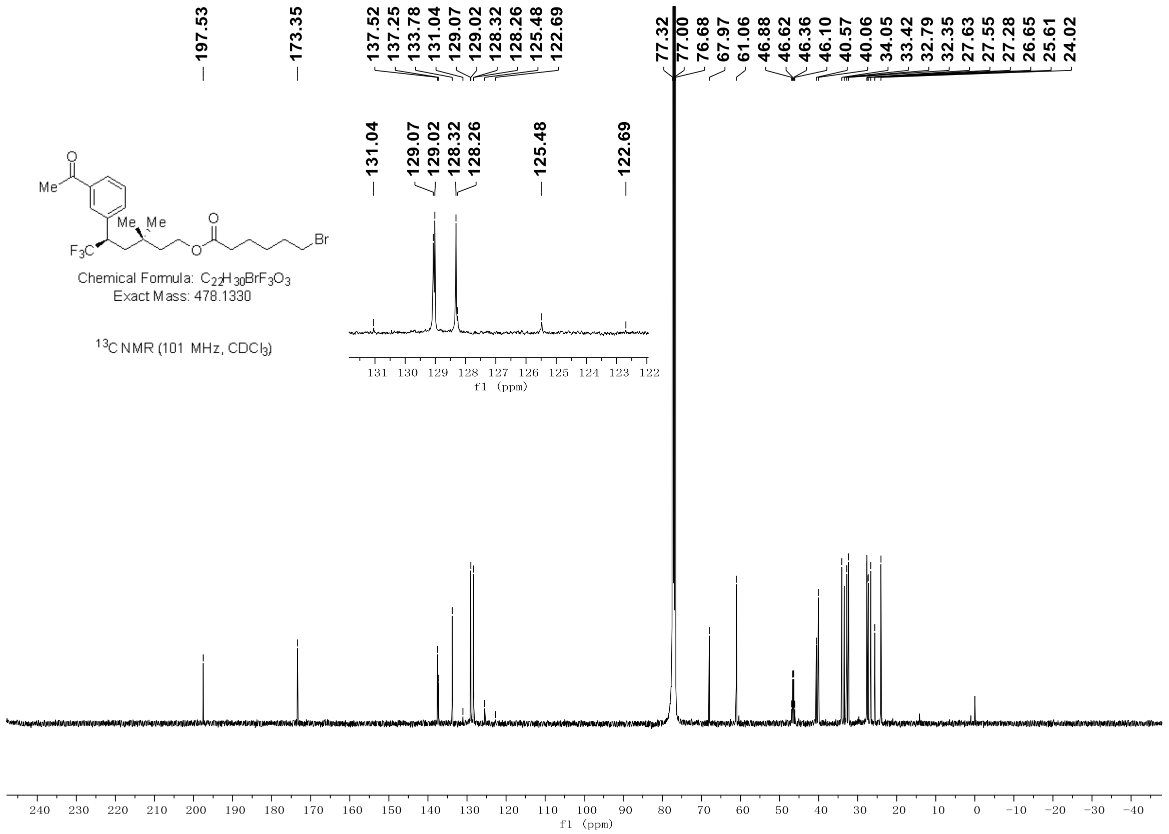

Supplementary Figure 142. ^{13}C NMR spectrum of compound 5i

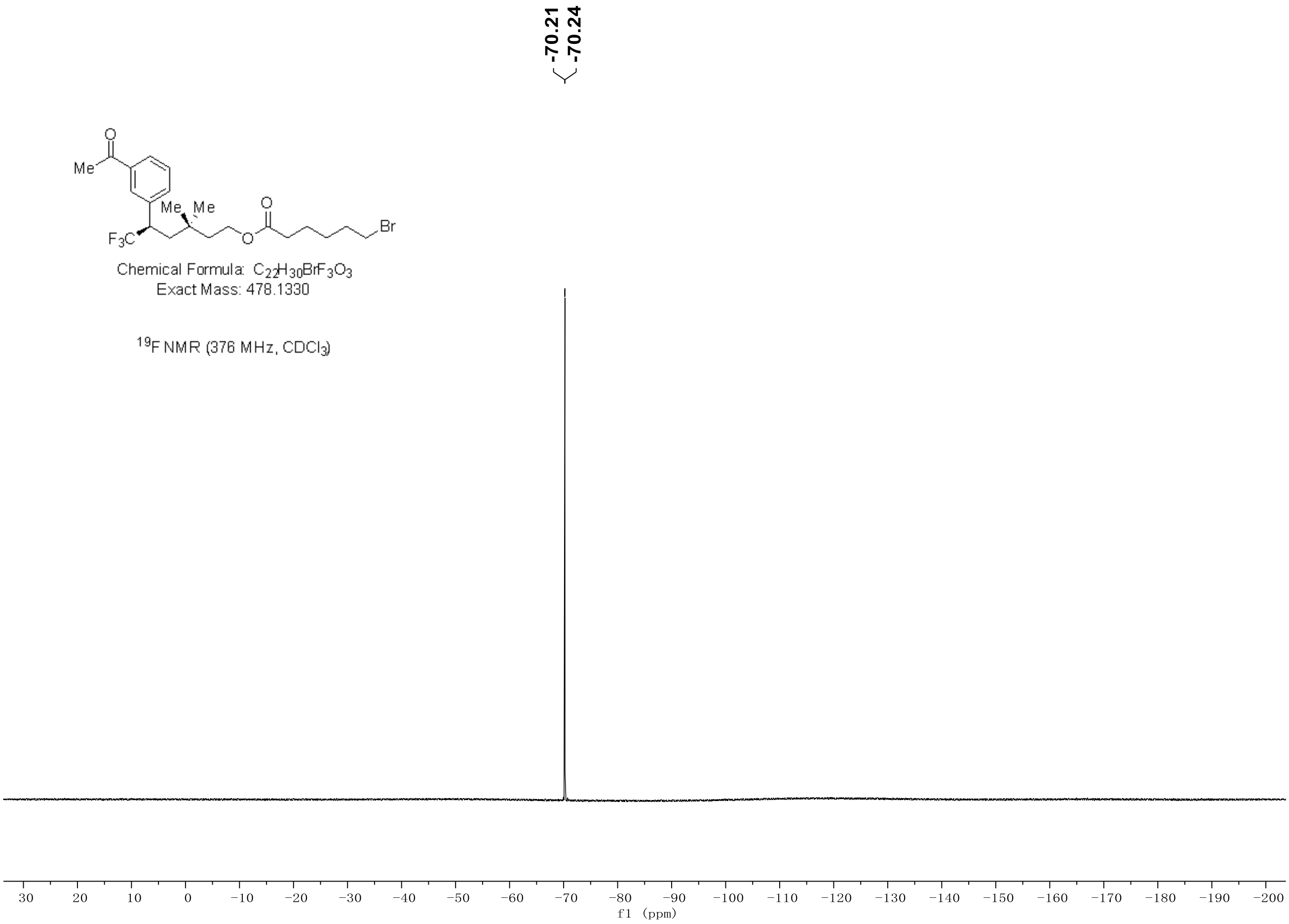

Supplementary Figure 143. ^{19}F NMR spectrum of compound 5i

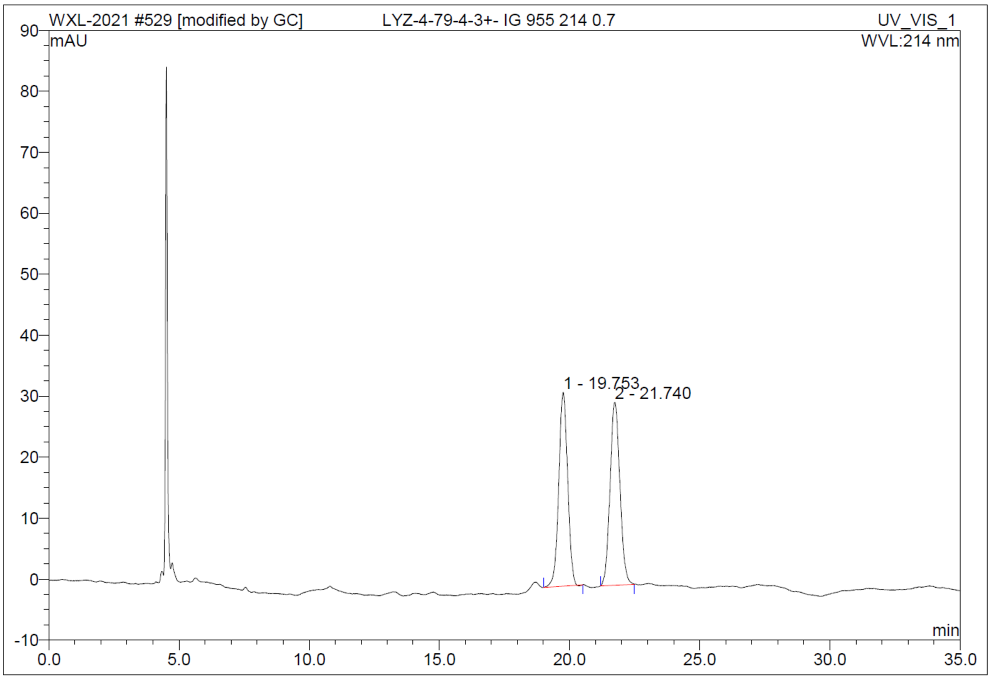

No.	Ret.Time min	Peak Name	Height mAU	Area mAU*min	Rel.Area %	Amount	Type
1	19.75	n.a.	31.800	12.618	48.84	n.a.	BMB*
2	21.74	n.a.	30.027	13.220	51.16	n.a.	BMB*
Total:			61.827	25.839	100.00	0.000	

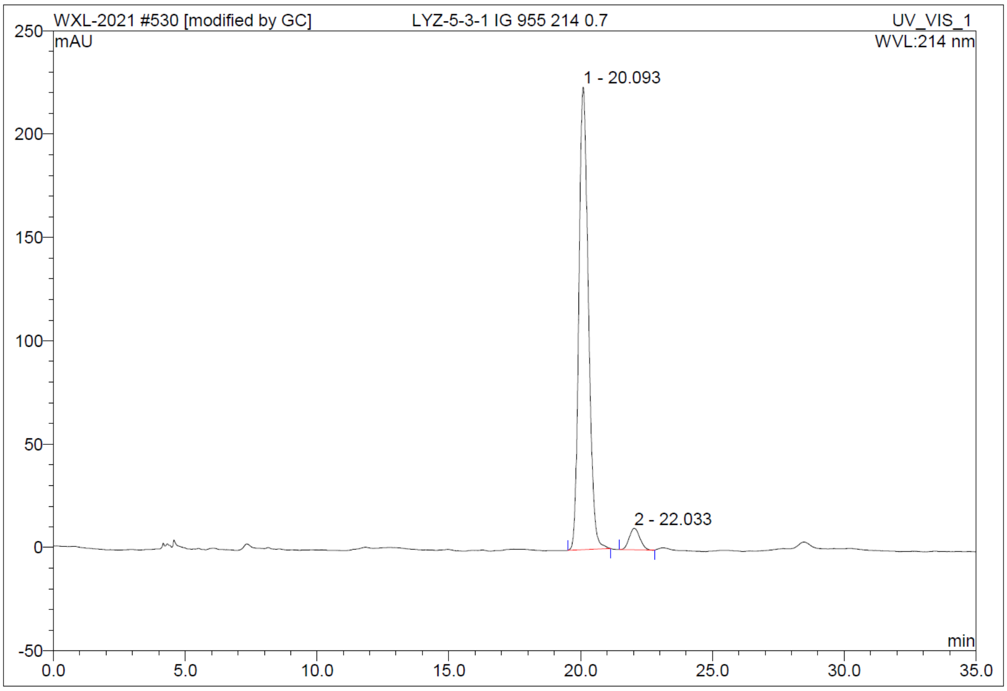

No.	Ret.Time min	Peak Name	Height mAU	Area mAU*min	Rel.Area %	Amount	Type
1	20.09	n.a.	223.899	93.295	95.10	n.a.	BMB*
2	22.03	n.a.	10.401	4.812	4.90	n.a.	BMB*
Total:			234.300	98.107	100.00	0.000	

Supplementary Figure 144. Chiral HPLC analysis of compound 5i

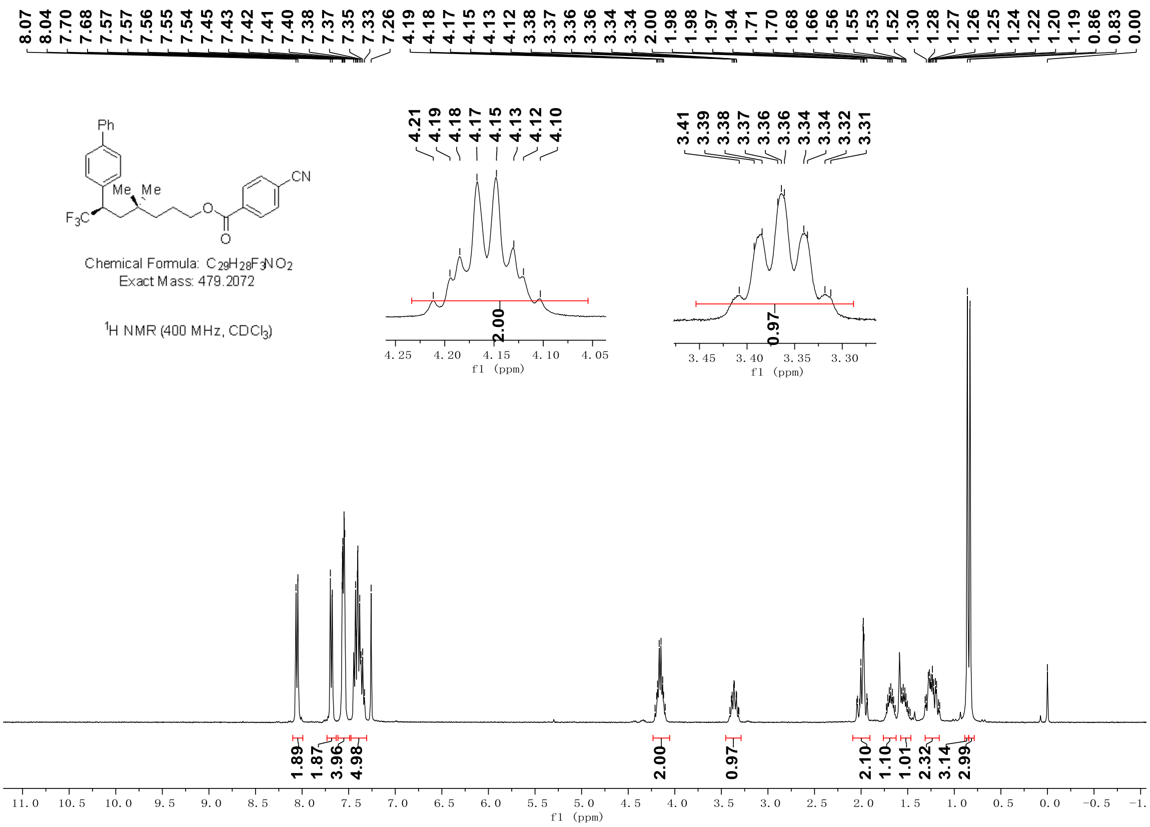

Supplementary Figure 145. ^{1}H NMR spectrum of compound 5j

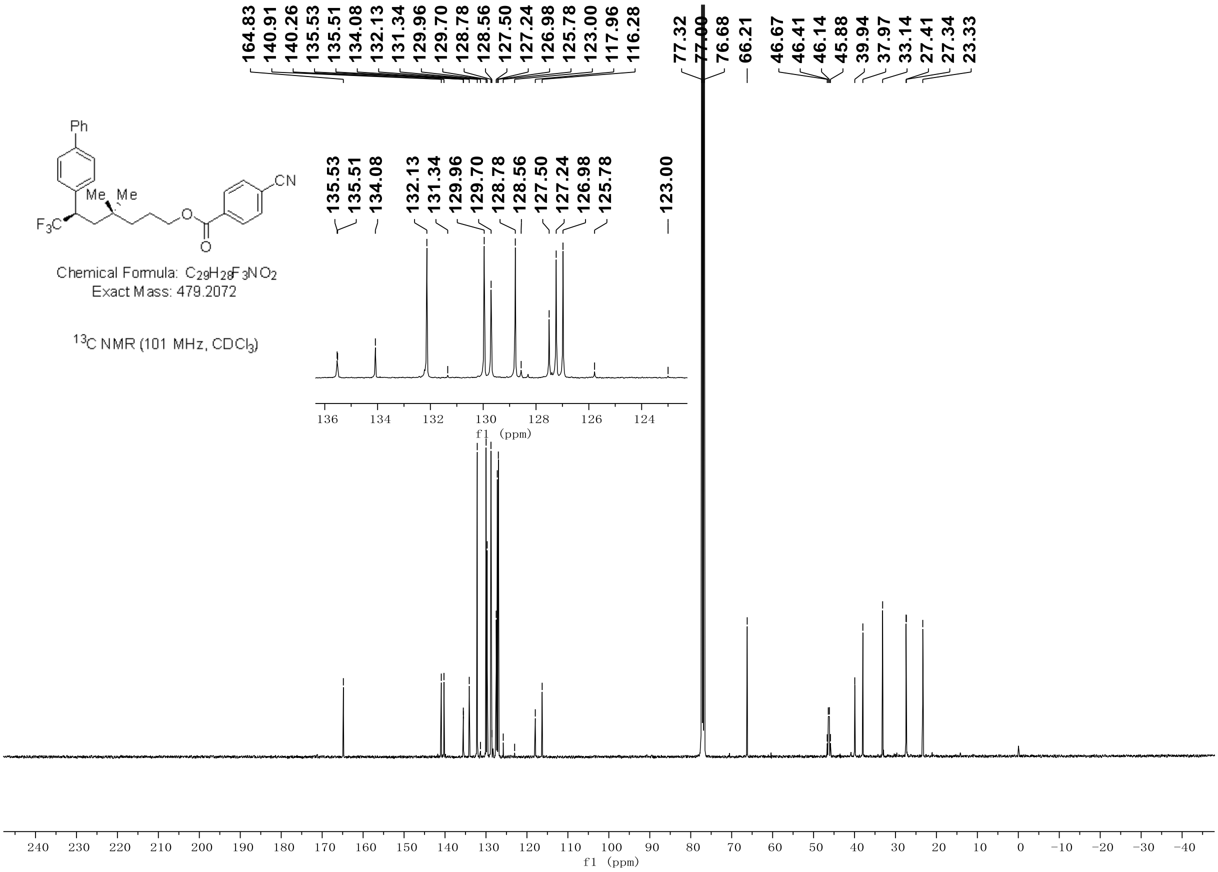

Supplementary Figure 146. ^{13}C NMR spectrum of compound 5j

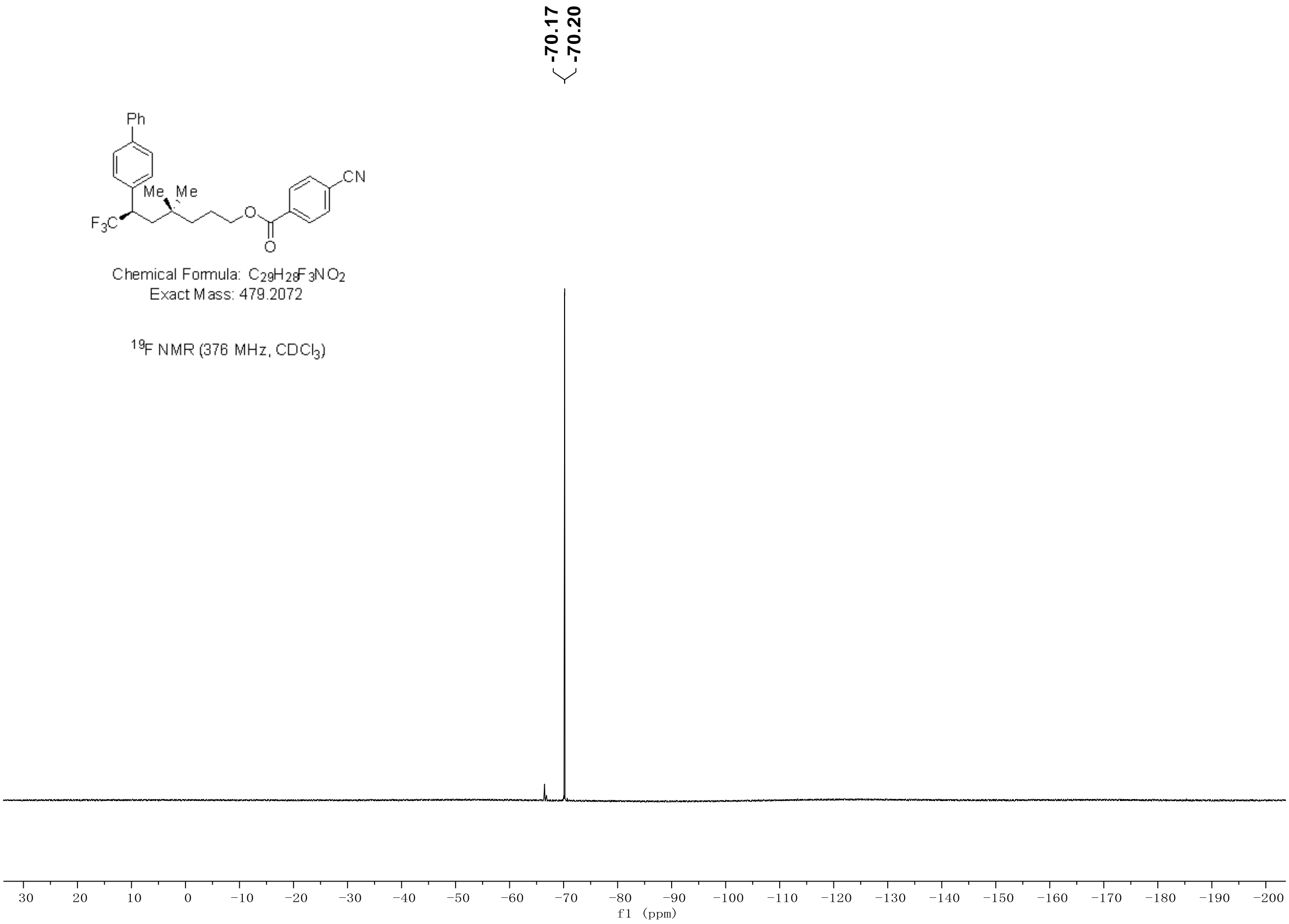

Supplementary Figure 147. ^{19}F NMR spectrum of compound 5j

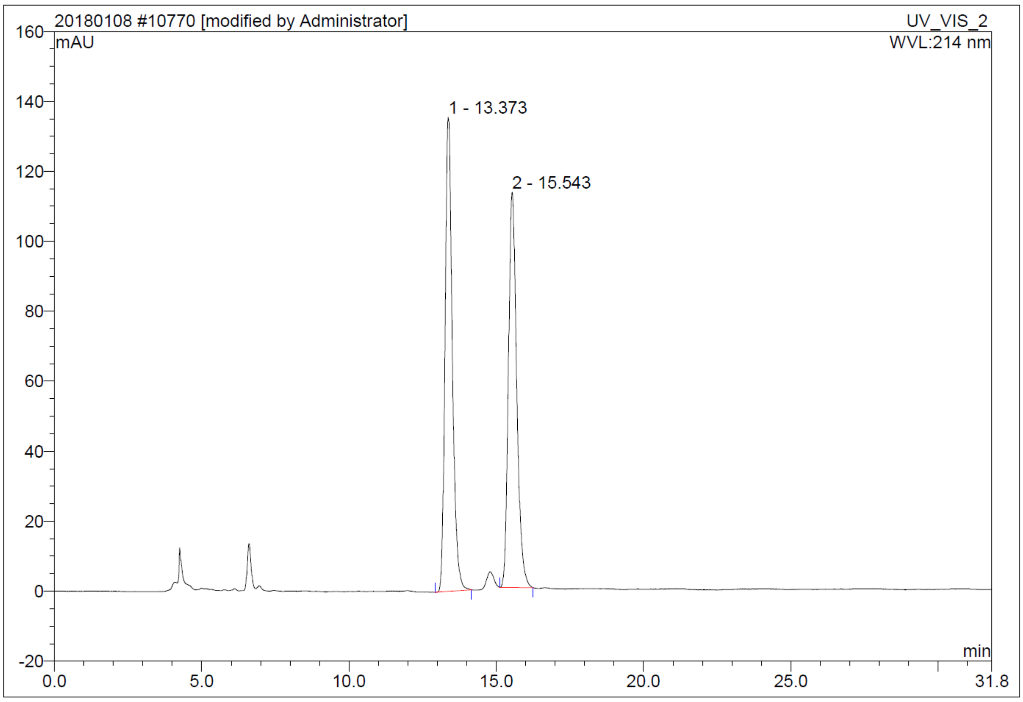

No.	Ret.Time min	Peak Name	Height mAU	Area mAU*min	Rel.Area %	Amount	Type
1	13.37	n.a.	135.507	38.657	51.23	n.a.	BMB*
2	15.54	n.a.	112.992	36.805	48.77	n.a.	BMB*
Total:			248.499	75.463	100.00	0.000	

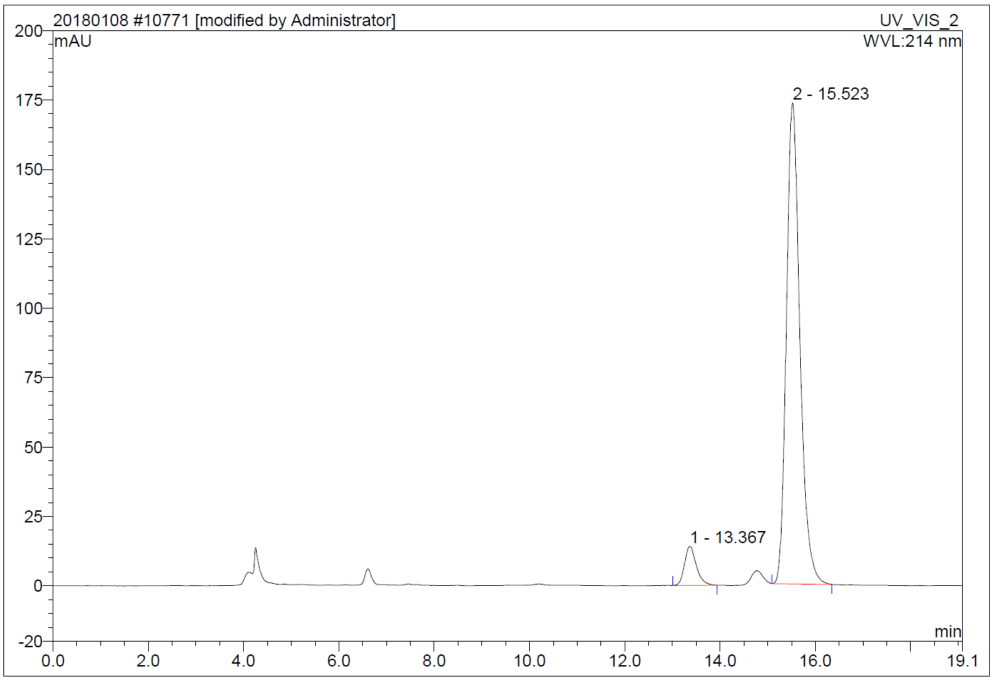

No.	Ret.Time min	Peak Name	Height mAU	Area mAU*min	Rel.Area %	Amount	Type
1	13.37	n.a.	14.011	4.015	6.59	n.a.	BMB
2	15.52	n.a.	173.279	56.952	93.41	n.a.	BMB*
Total:			187.290	60.966	100.00	0.000	

Supplementary Figure 148. Chiral HPLC analysis of compound 5j

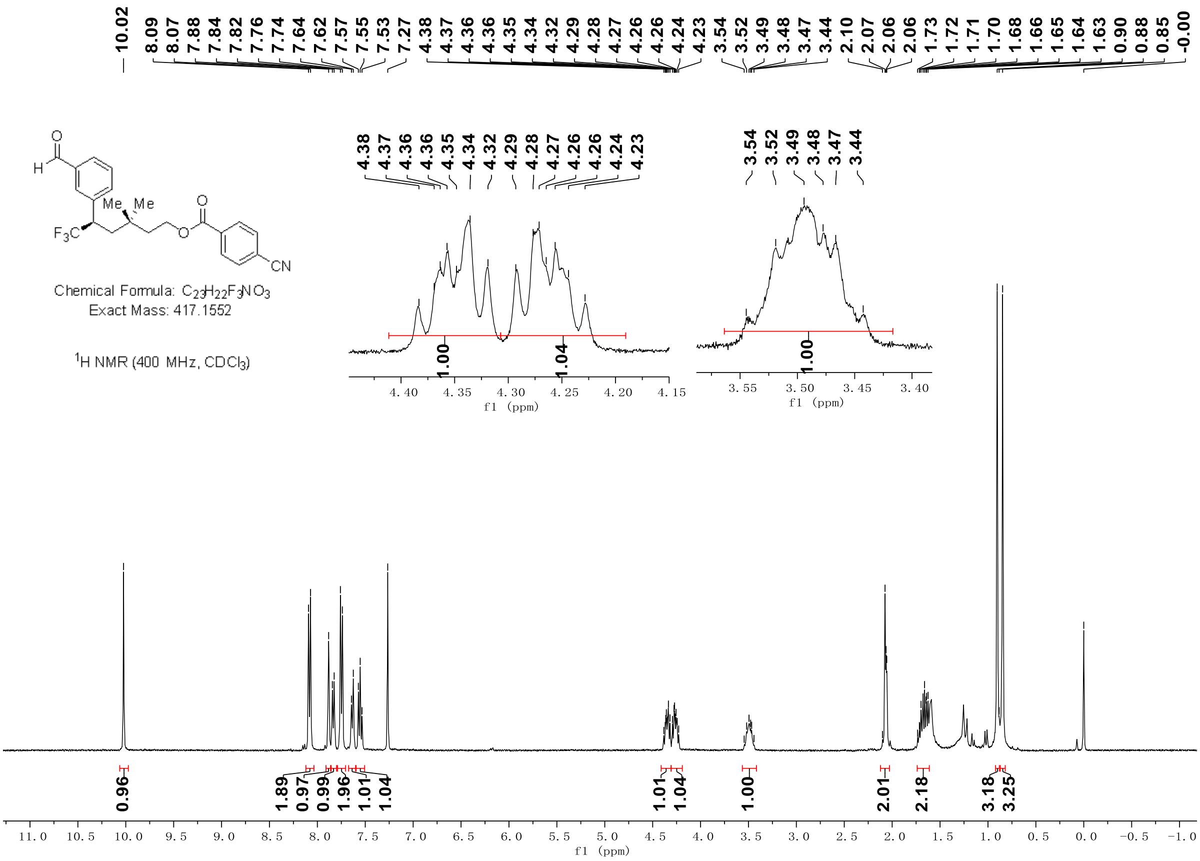

Supplementary Figure 149. ^{1}H NMR spectrum of compound 5k

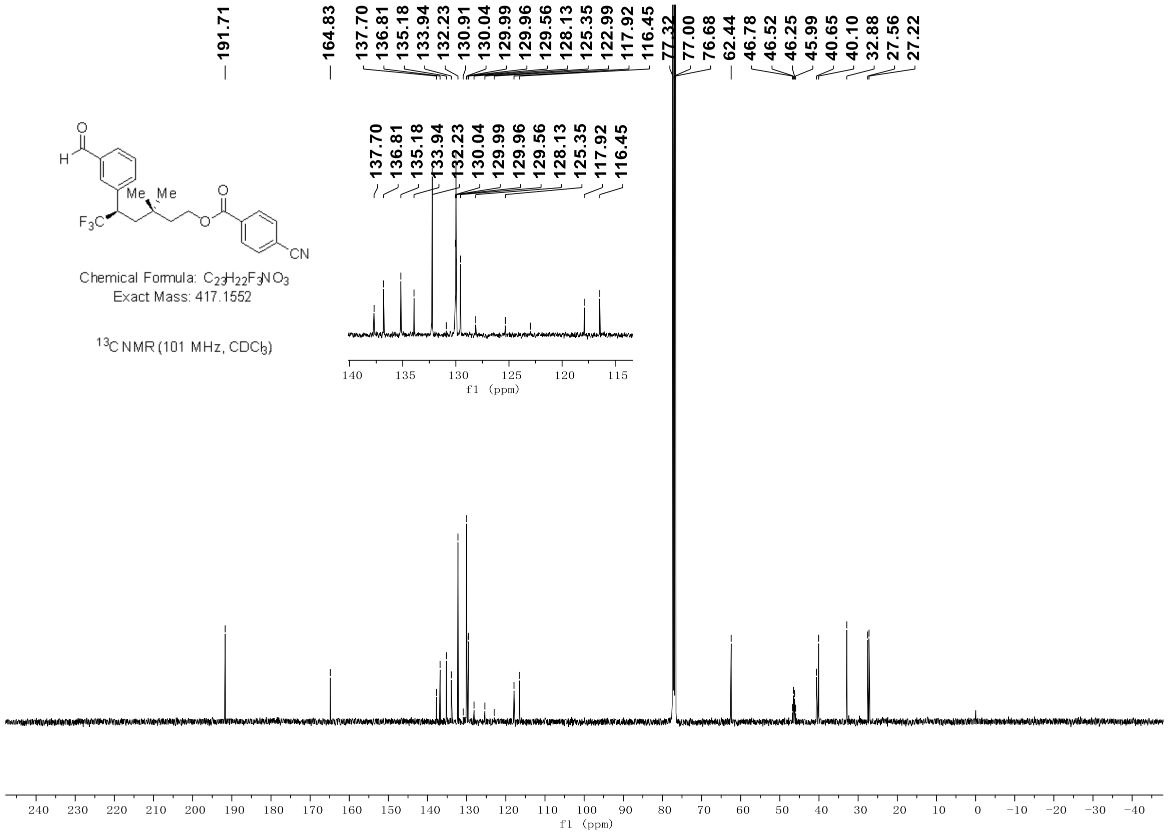

Supplementary Figure 150. ^{13}C NMR spectrum of compound 5k

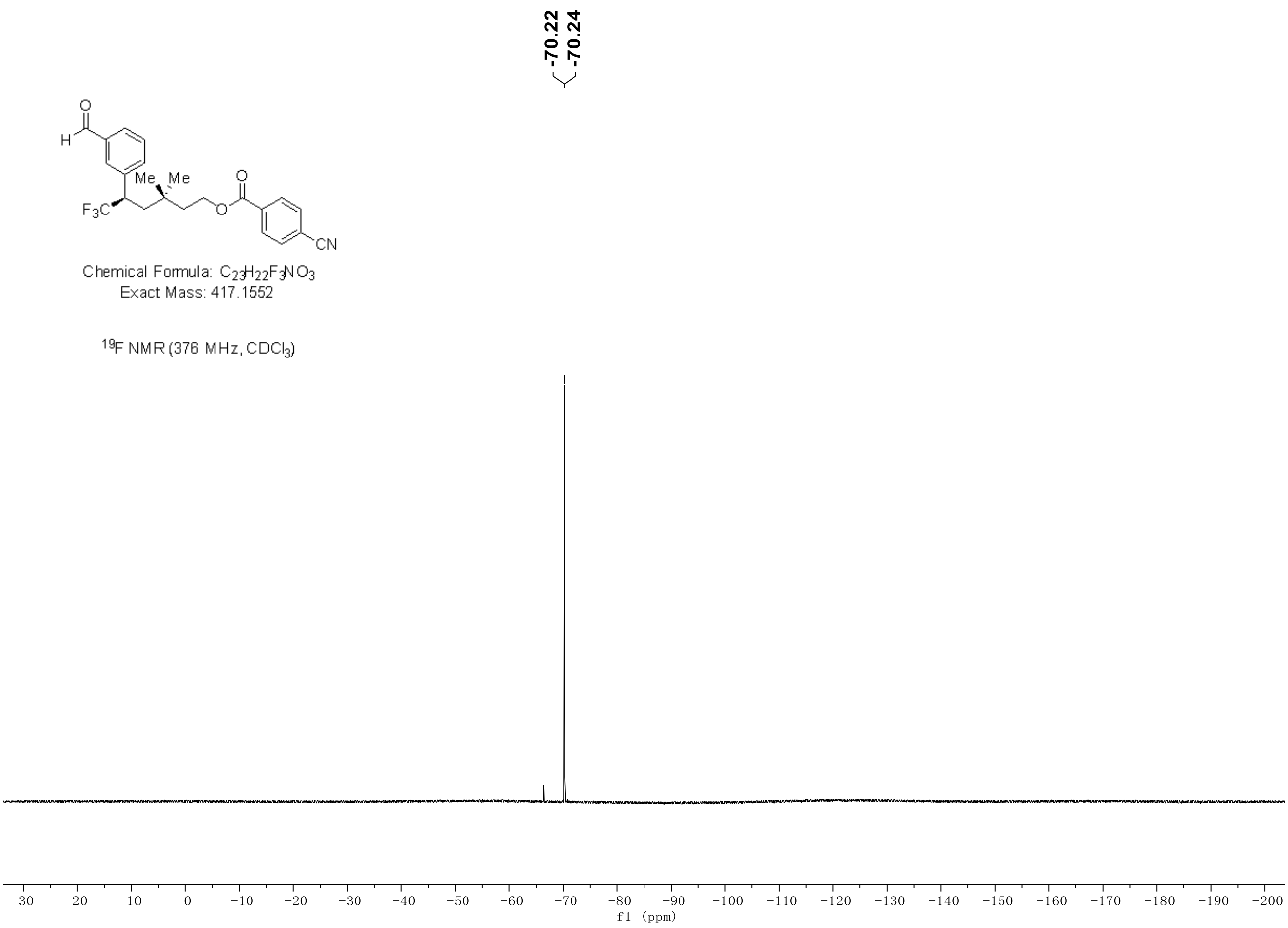

Supplementary Figure 151. ^{19}F NMR spectrum of compound 5k

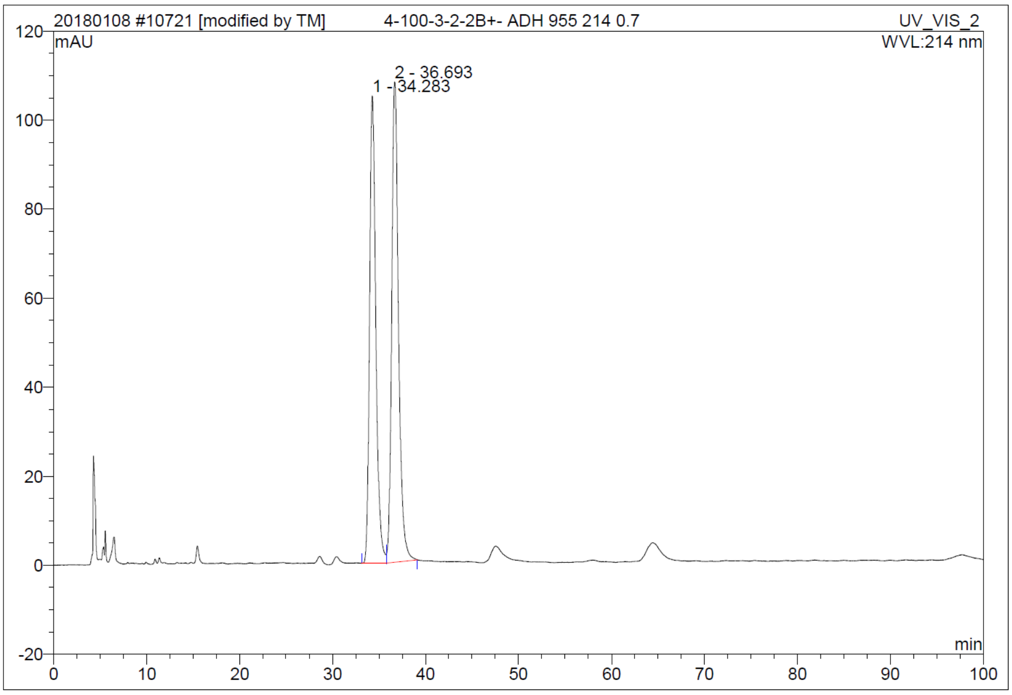

No.	Ret.Time min	Peak Name	Height mAU	Area mAU*min	Rel.Area %	Amount	Type
1	34.28	n.a.	105.027	81.994	47.24	n.a.	BM *
2	36.69	n.a.	107.973	91.560	52.76	n.a.	MB*
Total:			213.001	173.554	100.00	0.000	

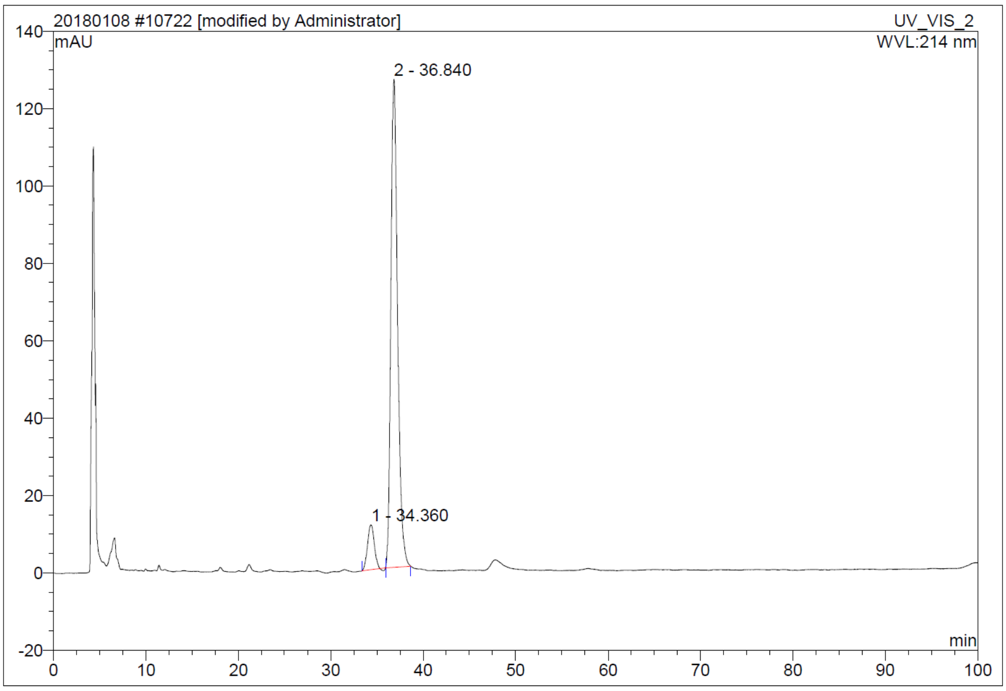

No.	Ret.Time min	Peak Name	Height mAU	Area mAU*min	Rel.Area %	Amount	Type
1	34.36	n.a.	11.585	9.568	8.25	n.a.	BMb*
2	36.84	n.a.	126.081	106.349	91.75	n.a.	bMB*
Total:			137.666	115.917	100.00	0.000	

Supplementary Figure 152. Chiral HPLC analysis of compound 5k

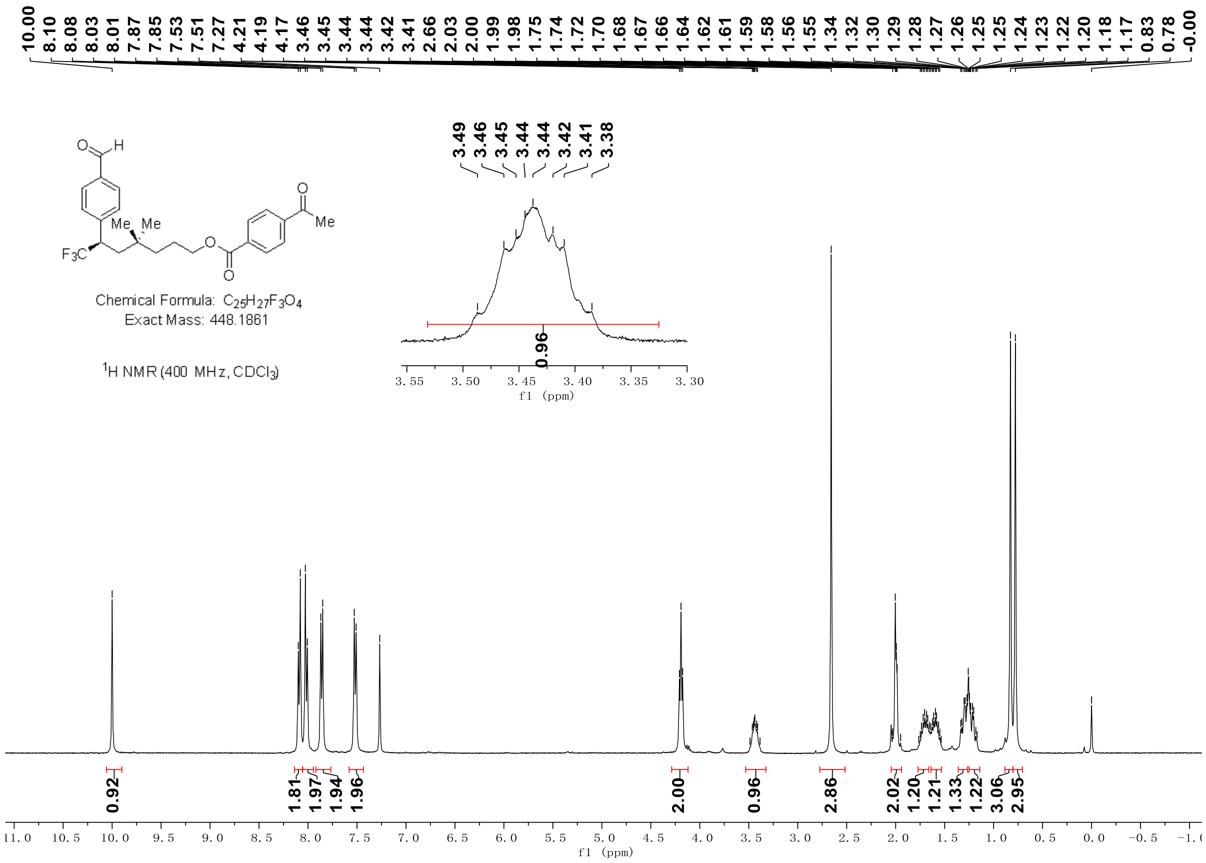

Supplementary Figure 153. ^{1}H NMR spectrum of compound 5l

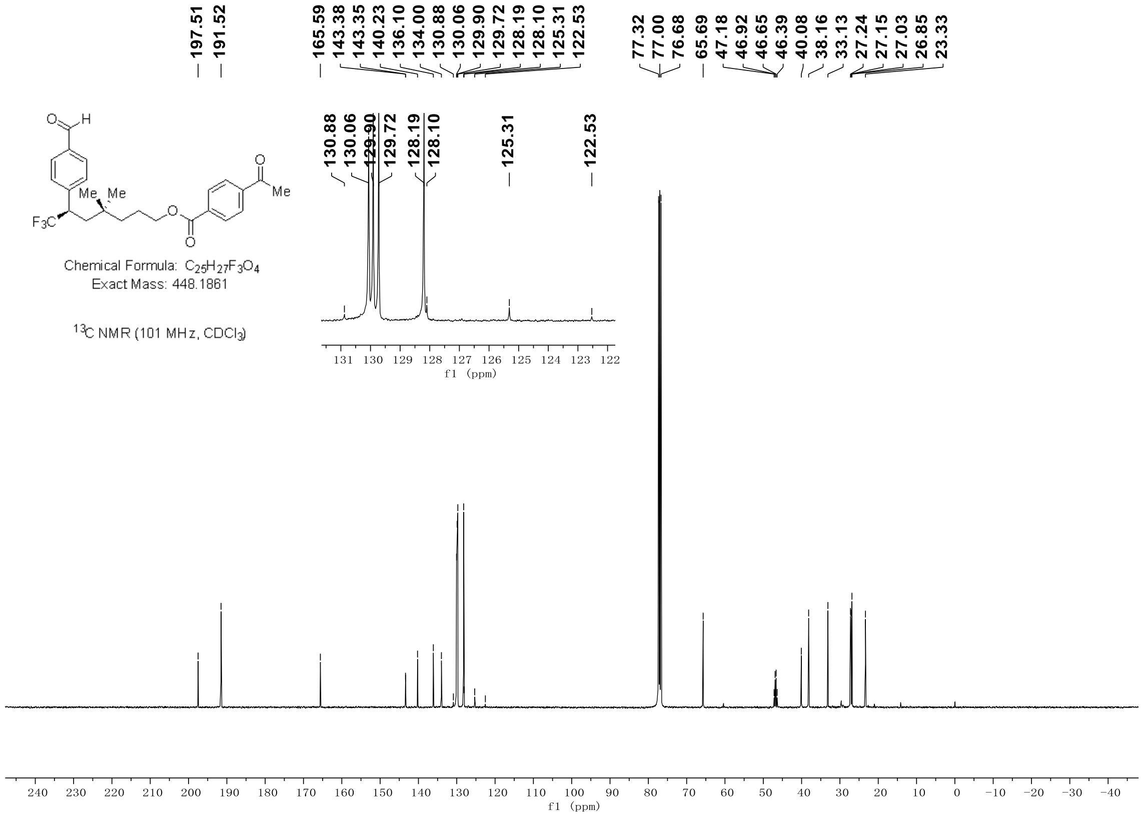

Supplementary Figure 154. ^{13}C NMR spectrum of compound 5l

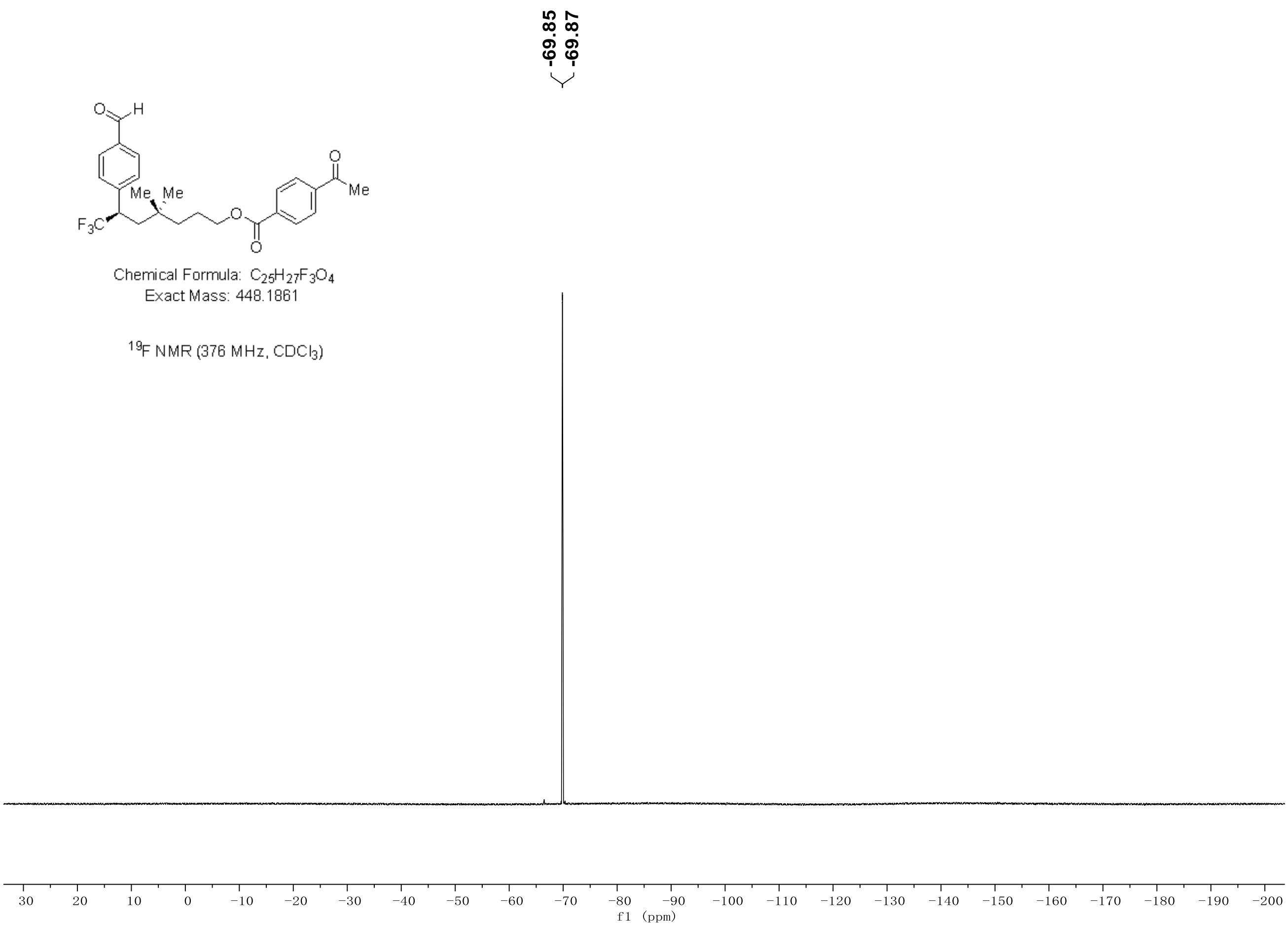

Supplementary Figure 155. ^{19}F NMR spectrum of compound 5l

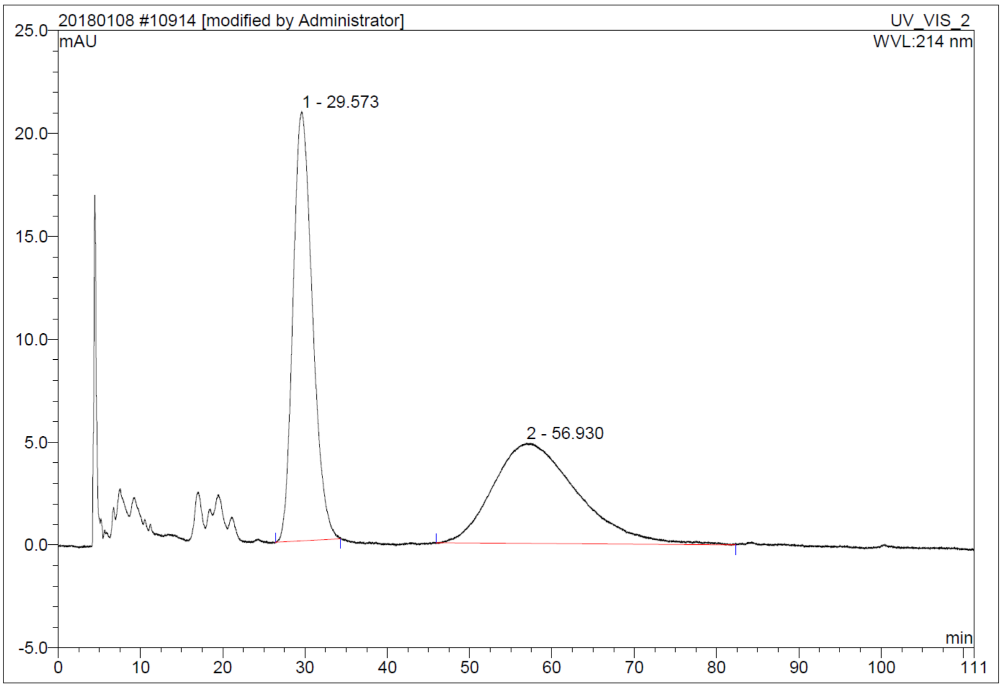

No.	Ret.Time min	Peak Name	Height mAU	Area mAU*min	Rel.Area %	Amount	Type
1	29.57	n.a.	20.894	57.073	49.63	n.a.	BMB*
2	56.93	n.a.	4.901	57.930	50.37	n.a.	BMB*
Total:			25.795	115.003	100.00	0.000	

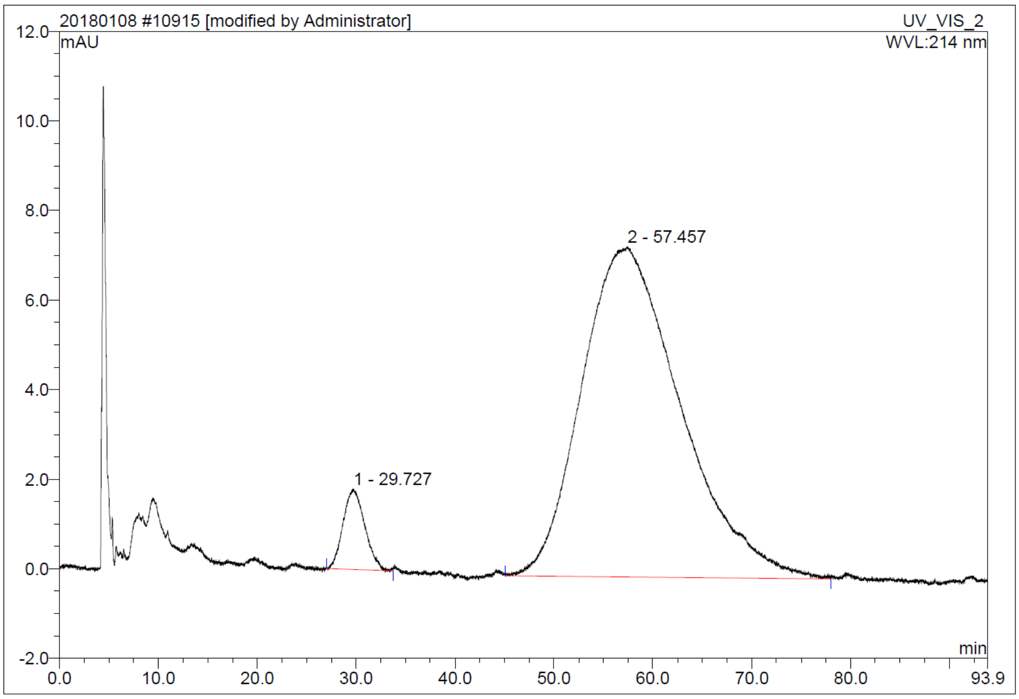

No.	Ret.Time min	Peak Name	Height mAU	Area mAU*min	Rel.Area %	Amount	Type
1	29.73	n.a.	1.808	4.570	5.08	n.a.	BMB*
2	57.46	n.a.	7.381	85.453	94.92	n.a.	BMB*
Total:			9.189	90.023	100.00	0.000	

Supplementary Figure 156. Chiral HPLC analysis of compound 5l

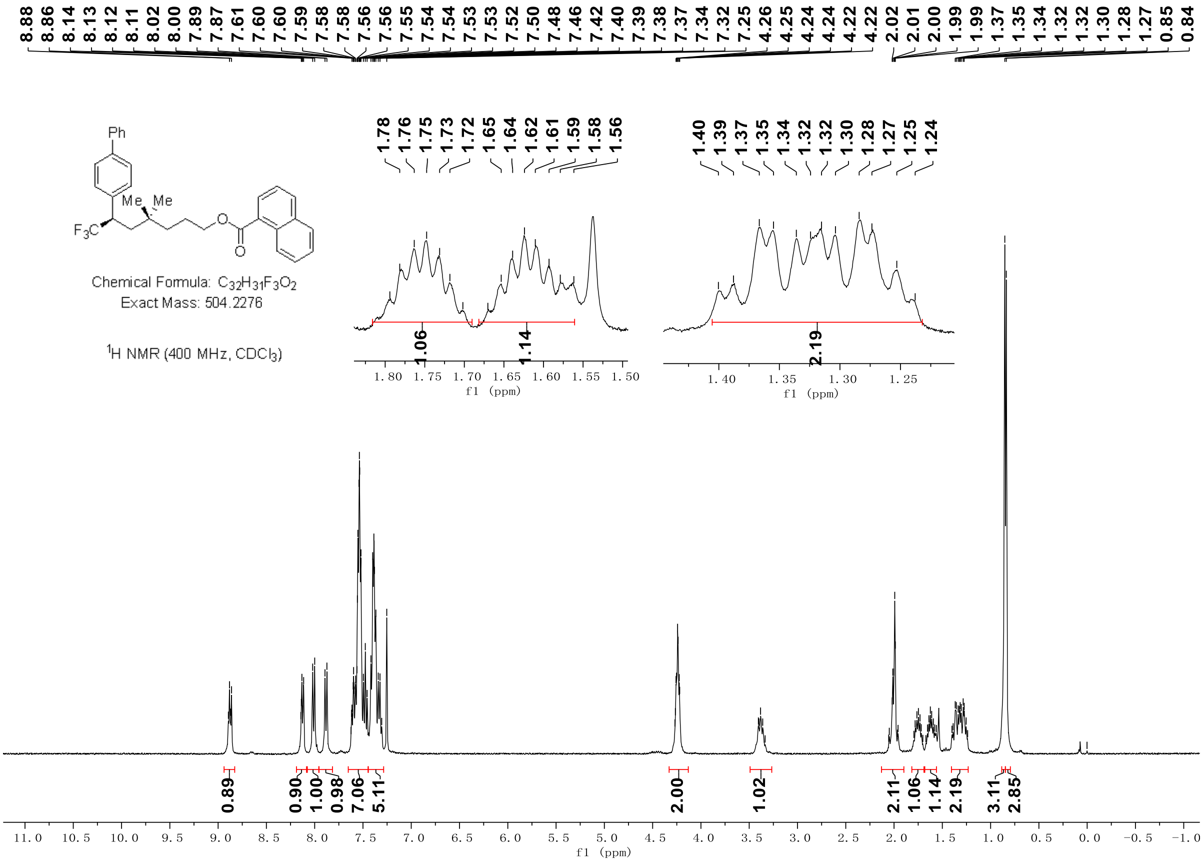

Supplementary Figure 157. ^{1}H NMR spectrum of compound 5m

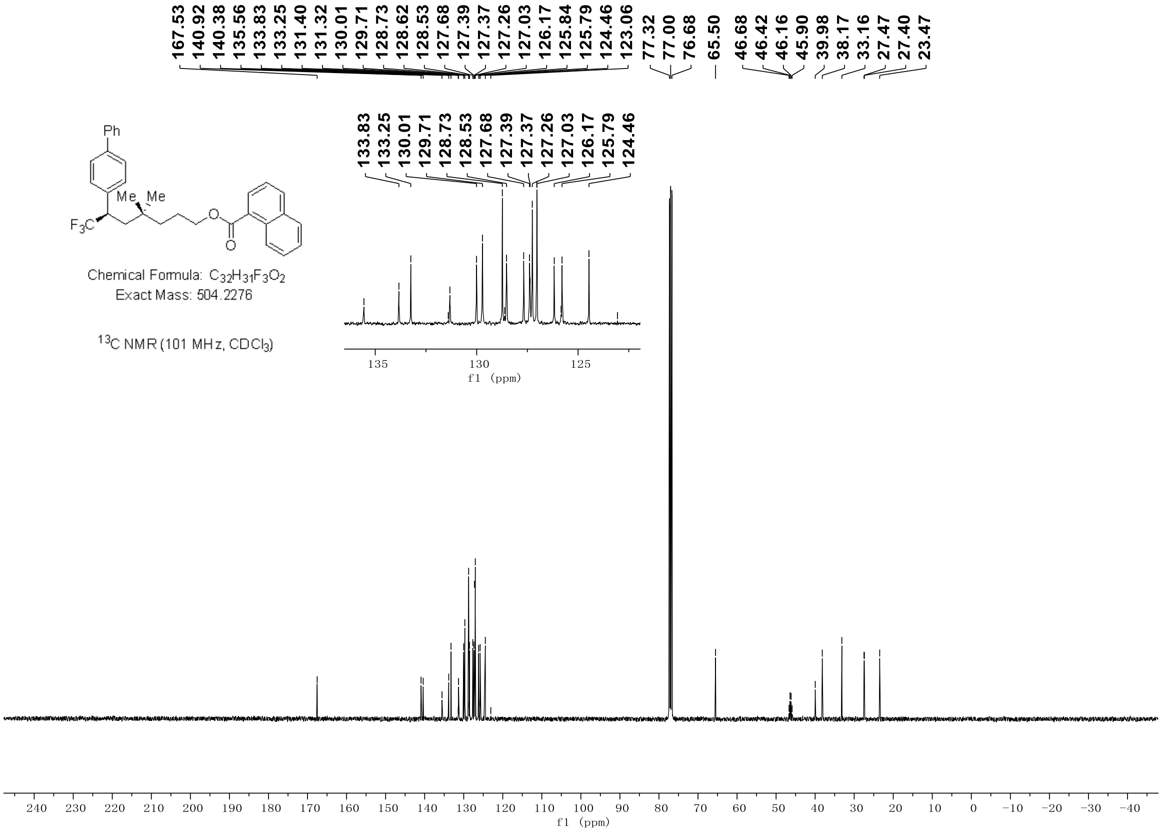

Supplementary Figure 158. ^{13}C NMR spectrum of compound 5m

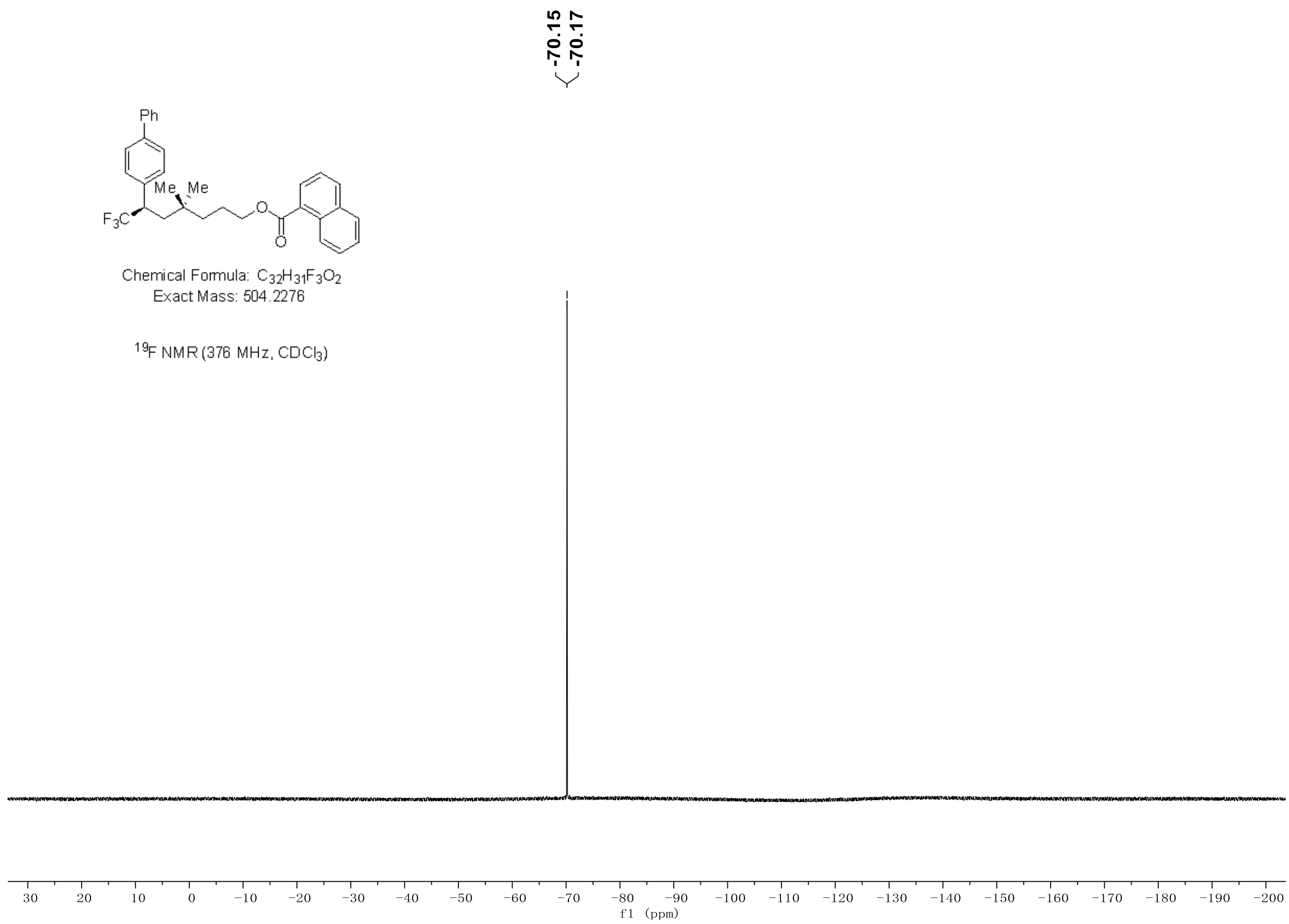

Supplementary Figure 159. ^{19}F **NMR spectrum of compound 5m**

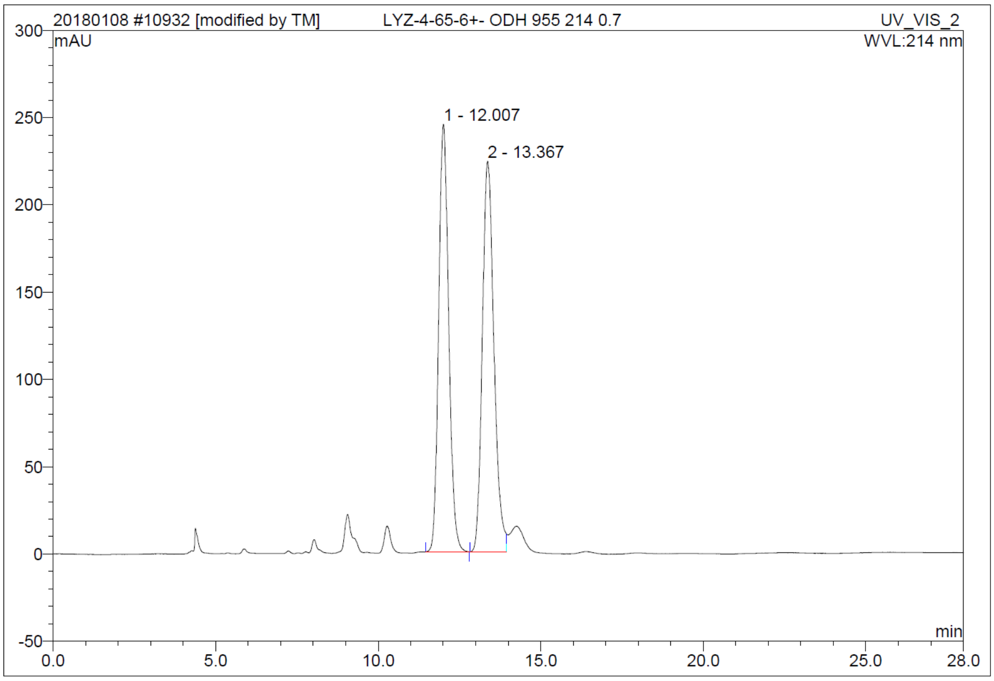

No.	Ret.Time min	Peak Name	Height mAU	Area mAU*min	Rel.Area %	Amount	Type
1	12.01	n.a.	245.029	87.708	48.70	n.a.	BM *
2	13.37	n.a.	223.896	92.378	51.30	n.a.	BM *
Total:			468.925	180.087	100.00	0.000	

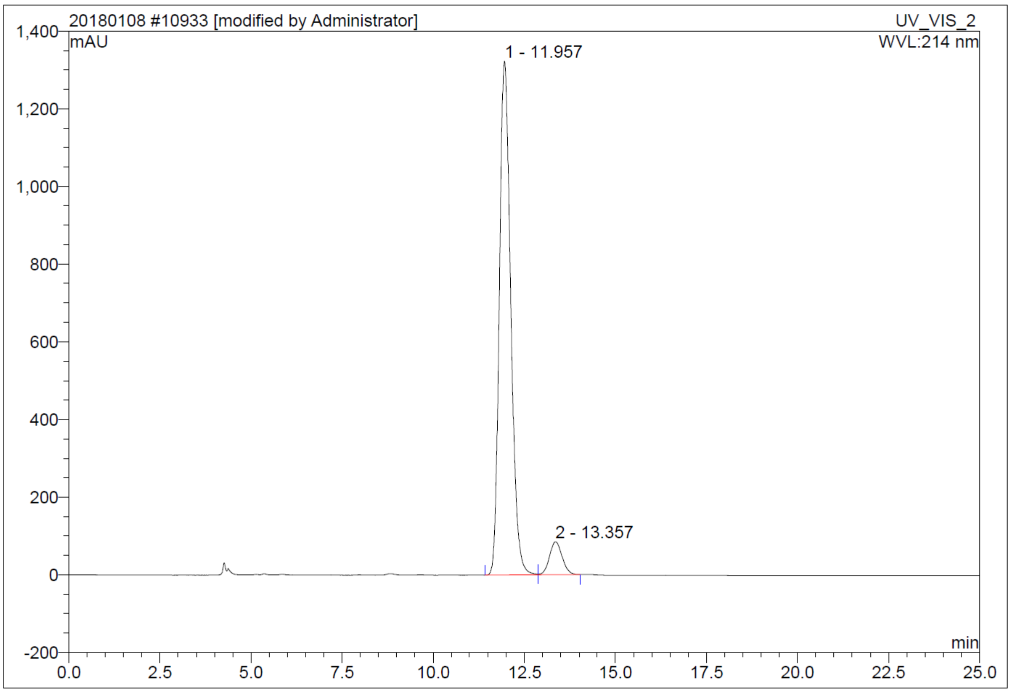

No.	Ret.Time min	Peak Name	Height mAU	Area mAU*min	Rel.Area %	Amount	Type
1	11.96	n.a.	1322.802	478.778	93.32	n.a.	BM
2	13.36	n.a.	84.867	34.283	6.68	n.a.	MB
Total:			1407.670	513.062	100.00	0.000	

Supplementary Figure 160. Chiral HPLC analysis of compound 5m

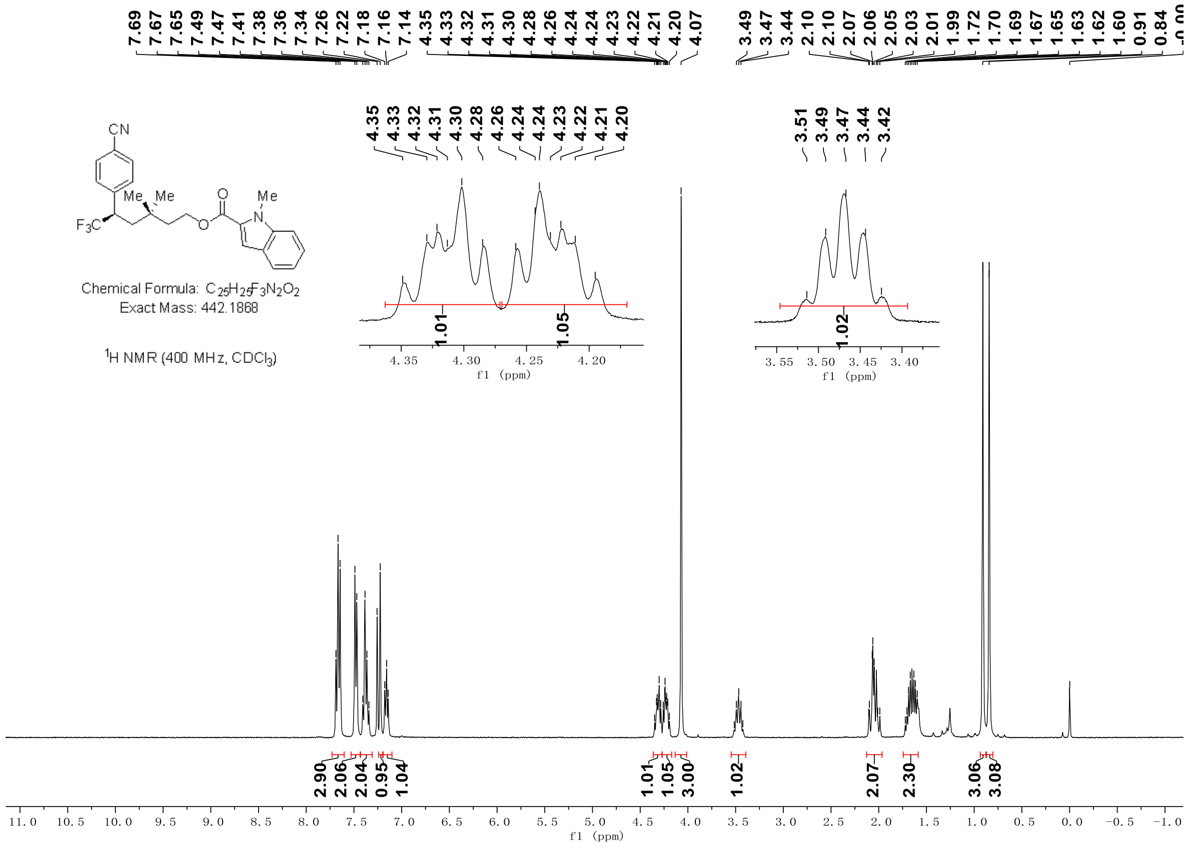

Supplementary Figure 161. ^{1}H NMR spectrum of compound 5n

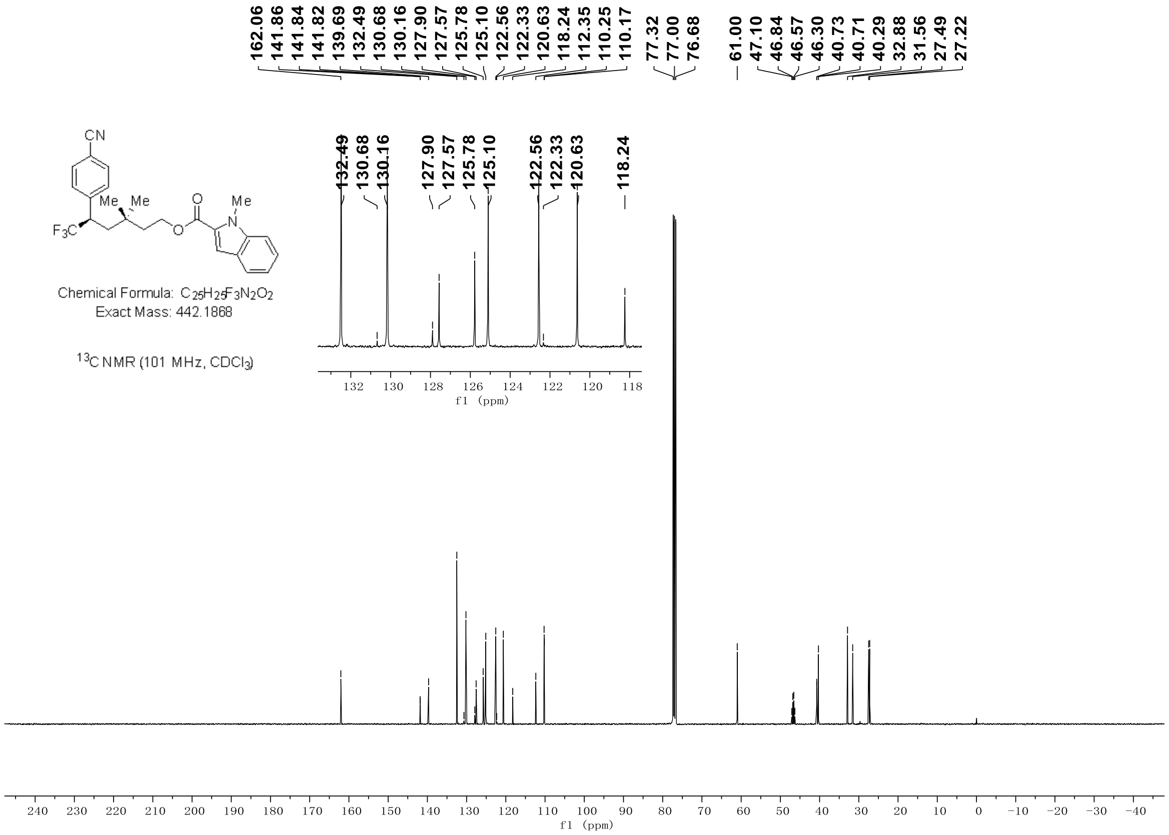

Supplementary Figure 162. ^{13}C NMR spectrum of compound 5n

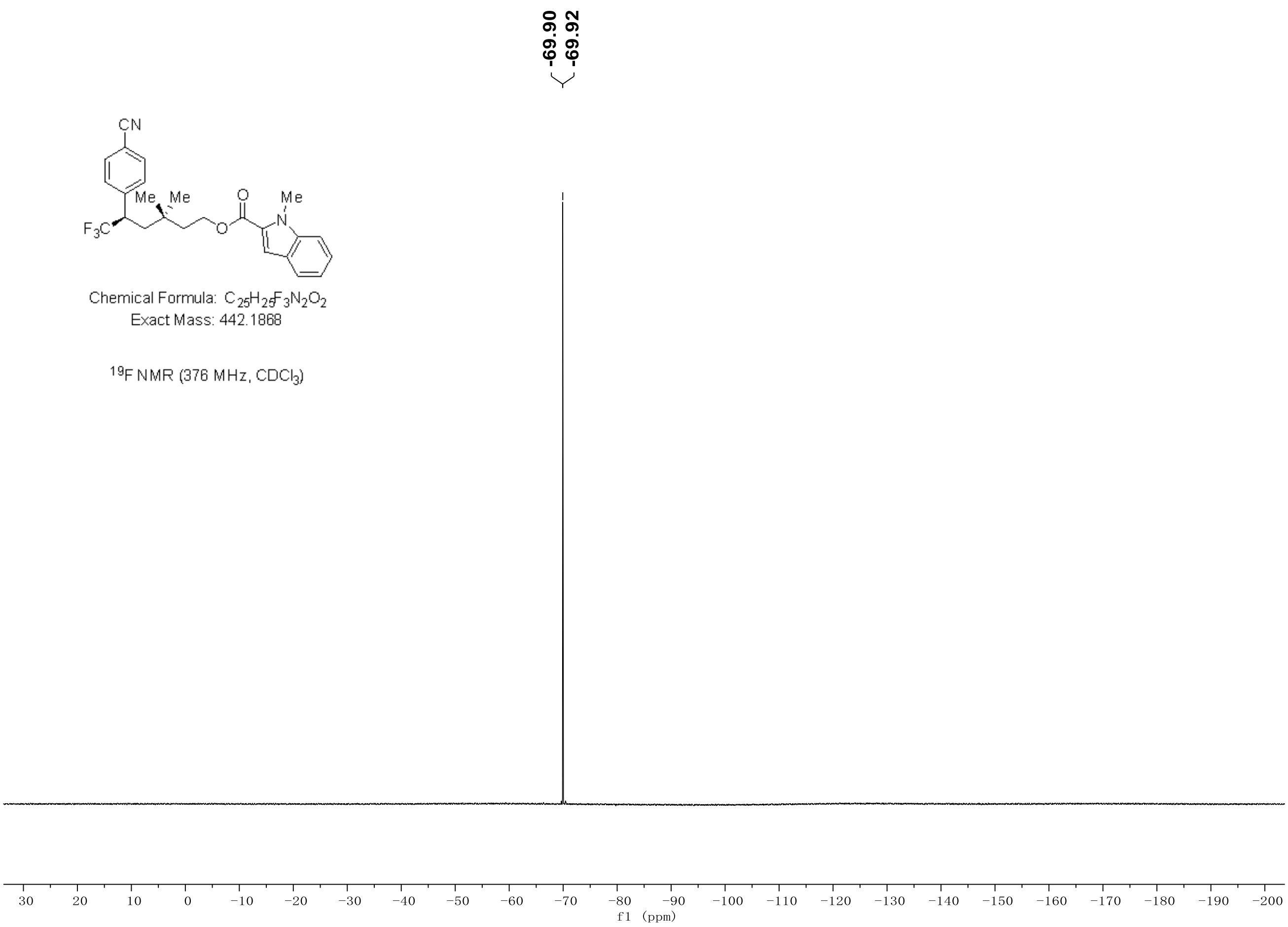

Supplementary Figure 163. ^{19}F NMR spectrum of compound 5n

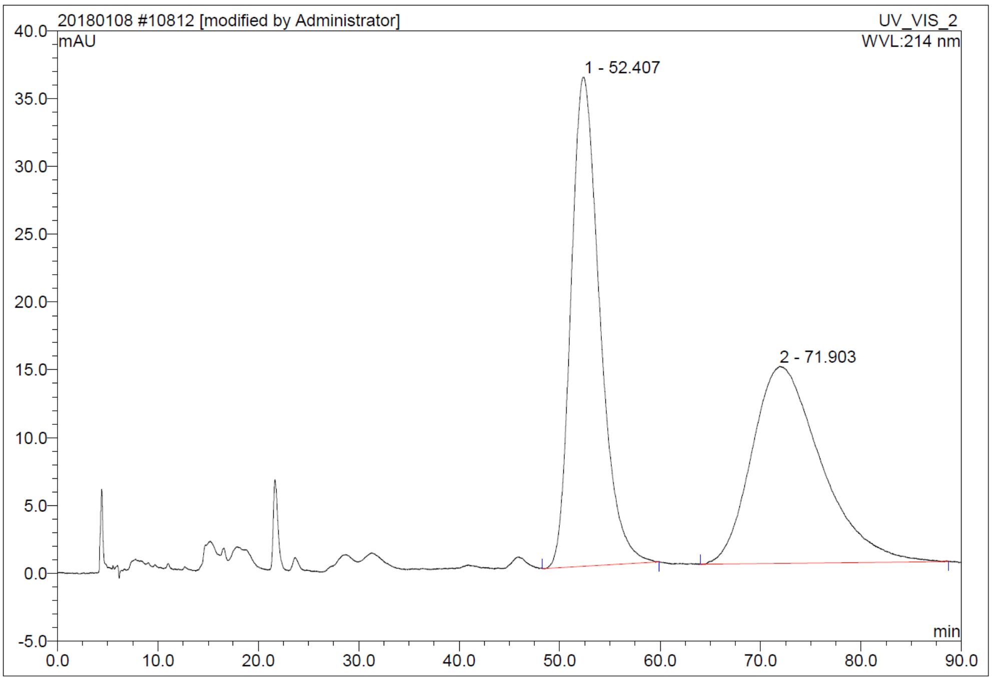

No.	Ret.Time min	Peak Name	Height mAU	Area mAU*min	Rel.Area %	Amount	Type
1	52.41	n.a.	36.053	119.911	50.83	n.a.	BMB*
2	71.90	n.a.	14.540	116.015	49.17	n.a.	BMB*
Total:			50.593	235.926	100.00	0.000	

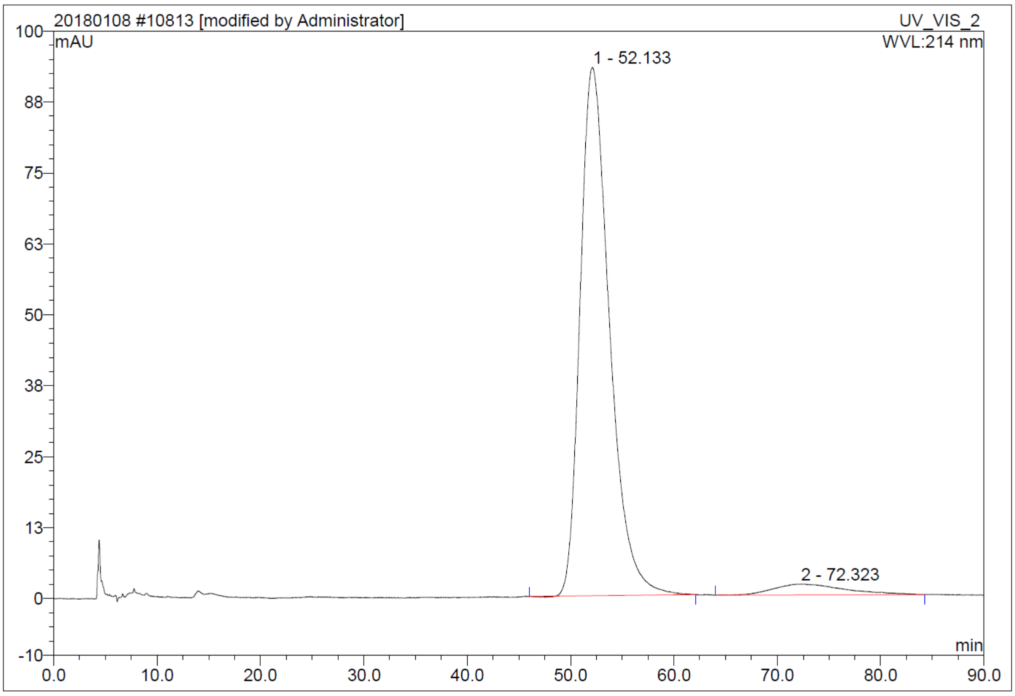

No.	Ret.Time min	Peak Name	Height mAU	Area mAU*min	Rel.Area %	Amount	Type
1	52.13	n.a.	93.098	306.476	95.18	n.a.	BMB*
2	72.32	n.a.	1.945	15.534	4.82	n.a.	BMB*
Total:			95.043	322.010	100.00	0.000	

Supplementary Figure 164. Chiral HPLC analysis of compound 5n

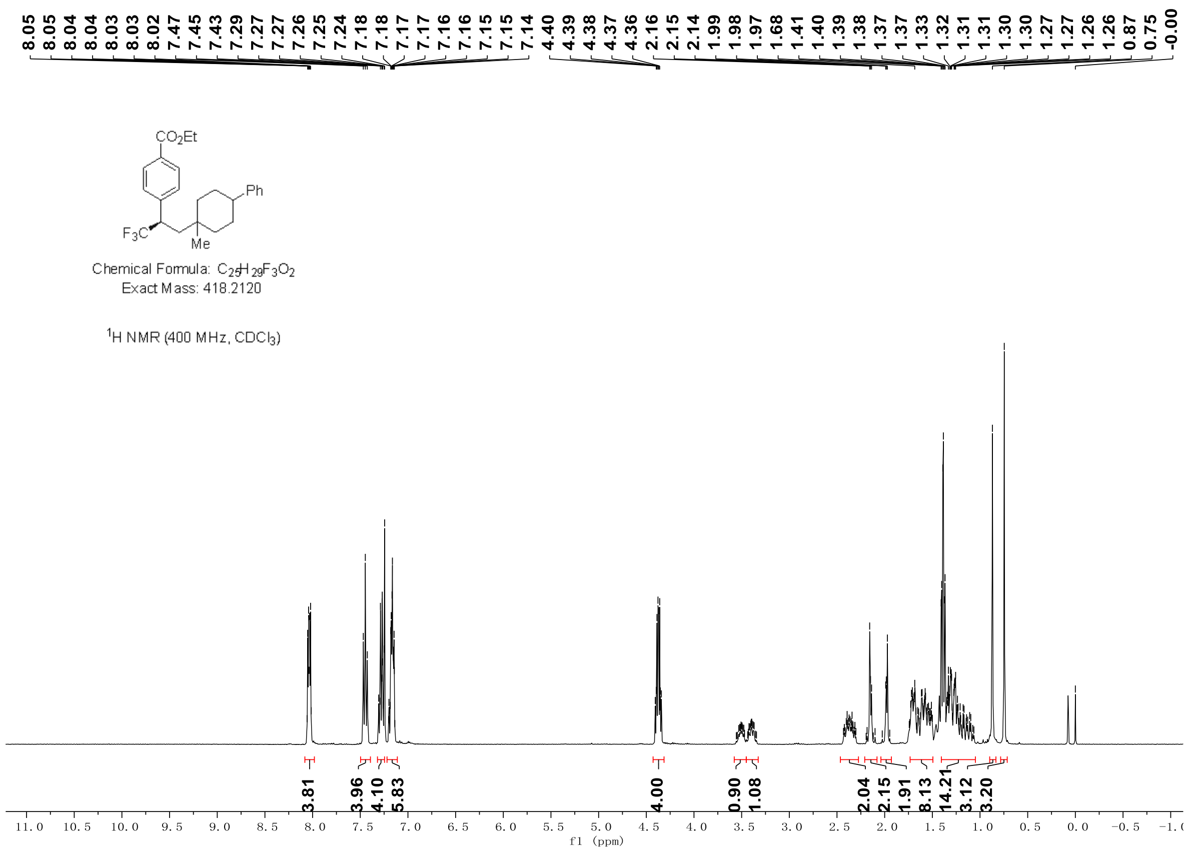

Supplementary Figure 165. ^{1}H NMR spectrum of compound 5o

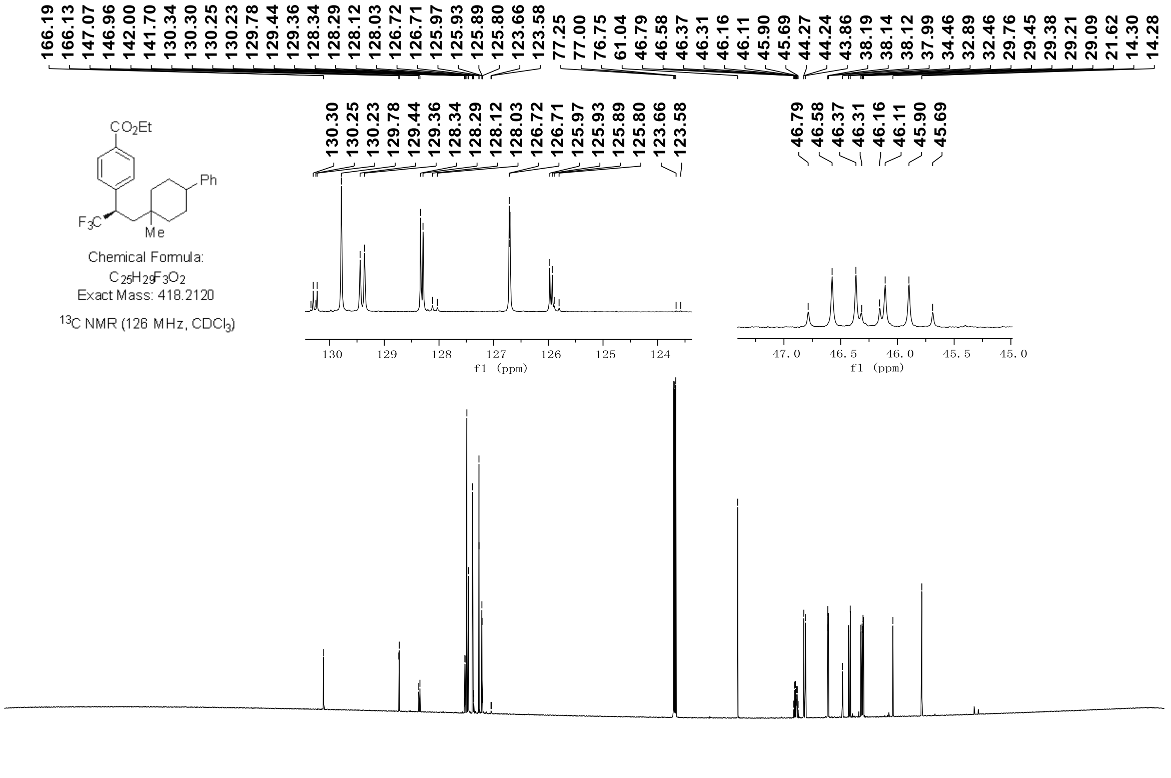

Supplementary Figure 166. ^{13}C NMR spectrum of compound 5o

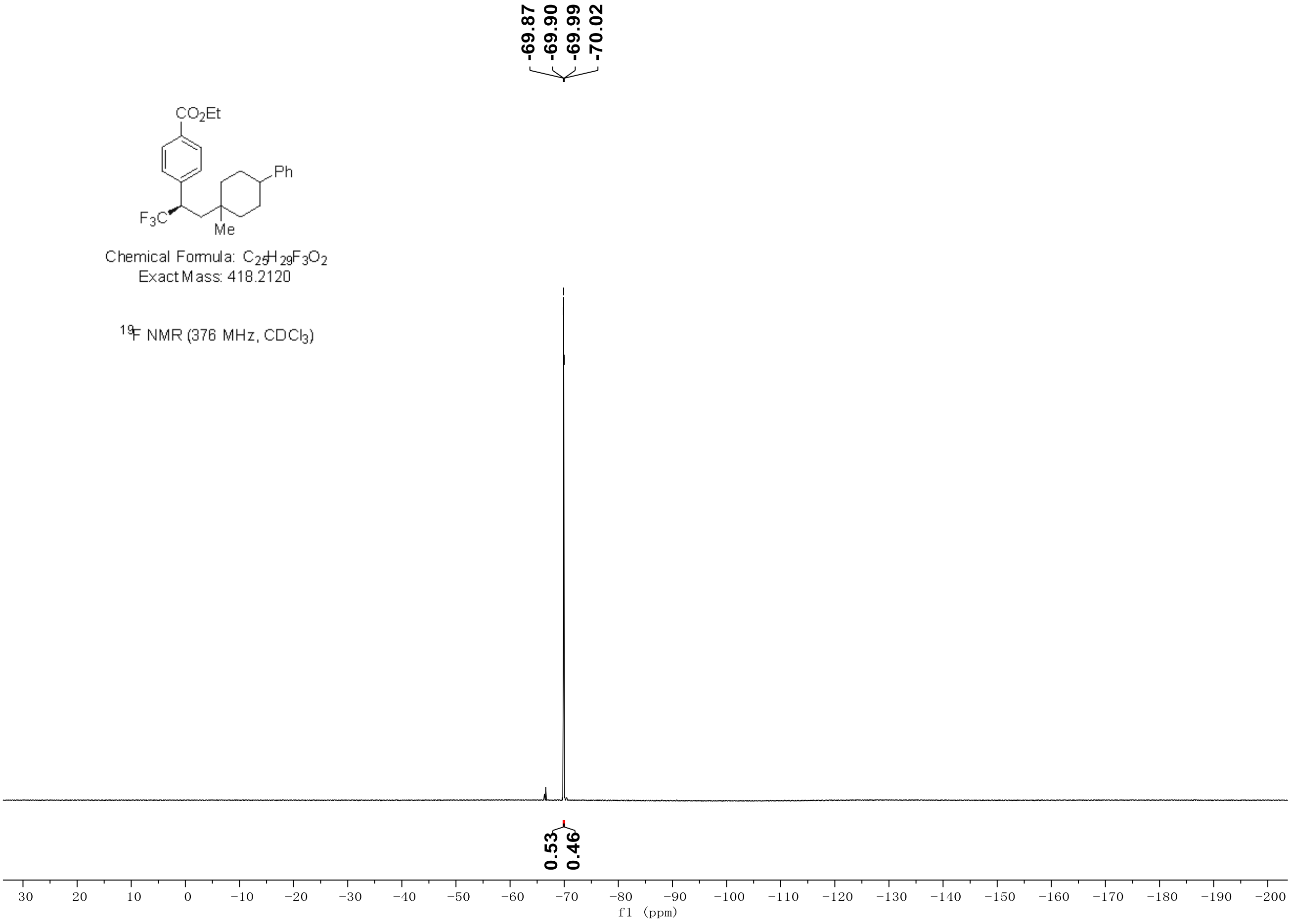

Supplementary Figure 167. ^{19}F NMR spectrum of compound 5o

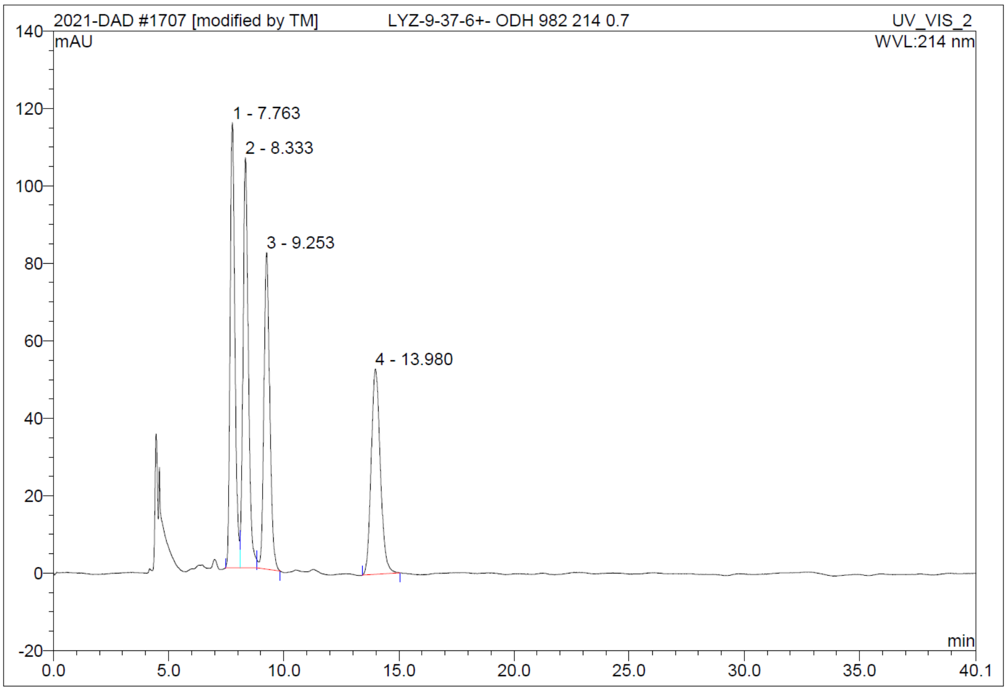

No.	Ret.Time min	Peak Name	Height mAU	Area mAU*min	Rel.Area %	Amount	Type
1	7.76	n.a.	114.939	27.627	26.43	n.a.	BM *
2	8.33	n.a.	105.911	28.443	27.21	n.a.	M *
3	9.25	n.a.	81.760	24.090	23.04	n.a.	MB*
4	13.98	n.a.	52.989	24.385	23.32	n.a.	BMB*
Total:			355.600	104.546	100.00	0.000	

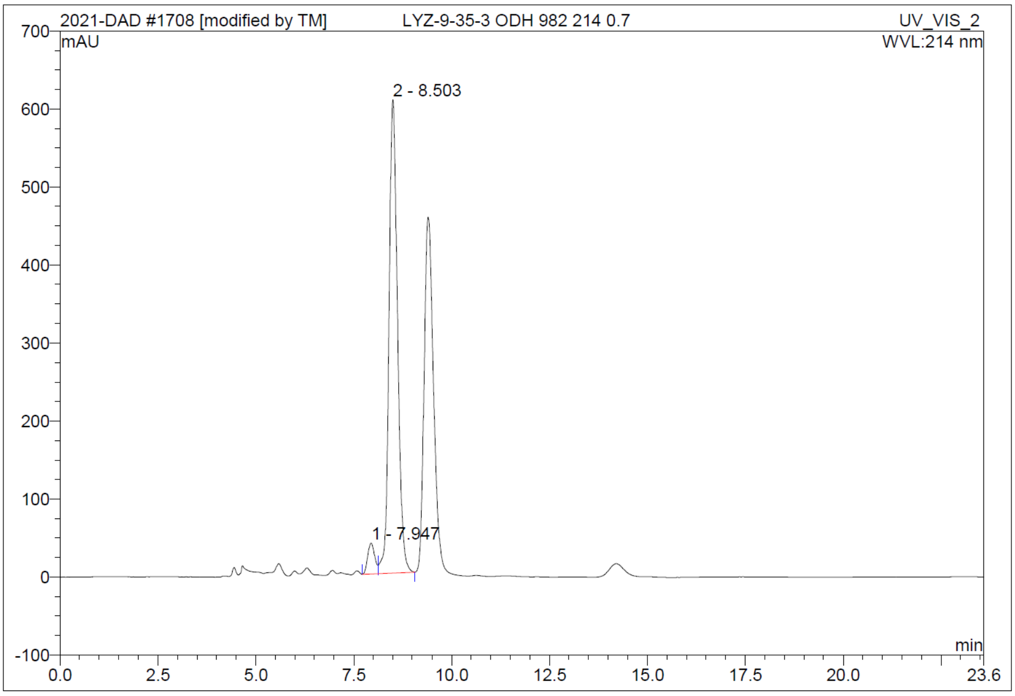

No.	Ret.Time min	Peak Name	Height mAU	Area mAU*min	Rel.Area %	Amount	Type
1	7.95	n.a.	39.534	8.197	5.08	n.a.	BM *
2	8.50	n.a.	606.556	153.271	94.92	n.a.	MB*
Total:			646.090	161.468	100.00	0.000	

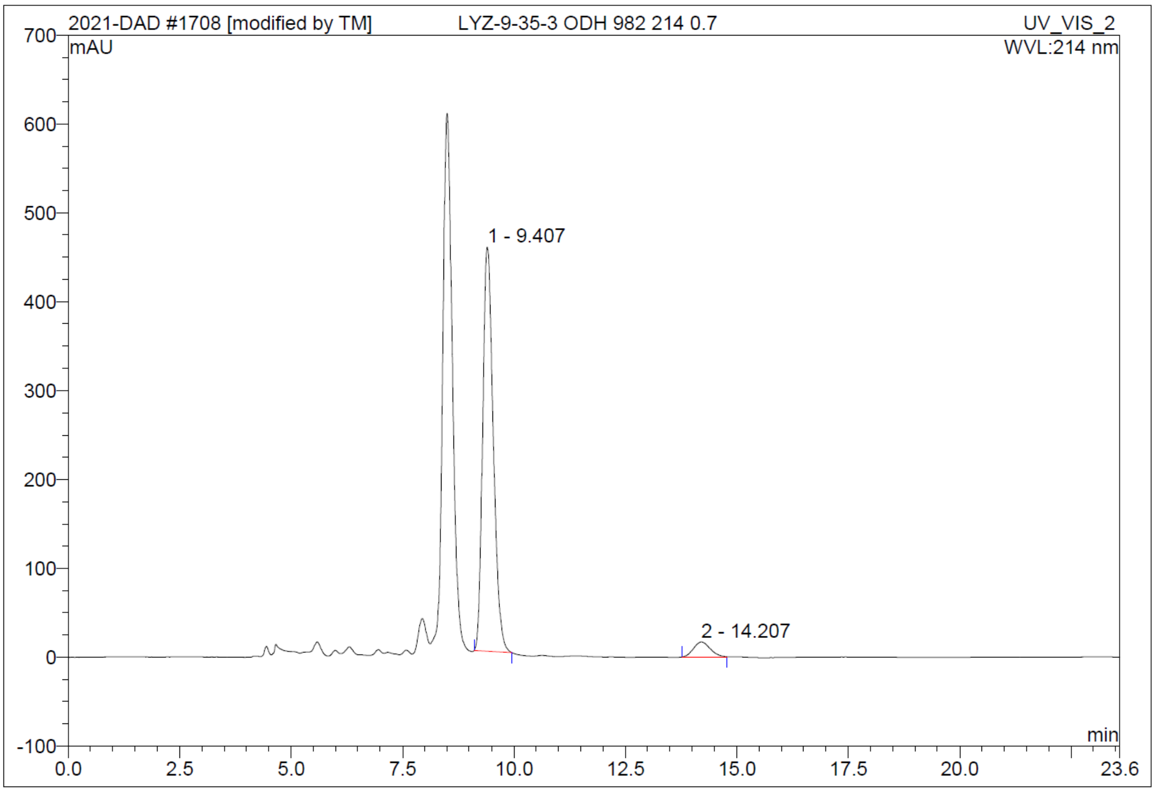

No.	Ret.Time min	Peak Name	Height mAU	Area mAU*min	Rel.Area %	Amount	Type
1	9.41	n.a.	454.684	124.940	94.57	n.a.	BMB*
2	14.21	n.a.	16.705	7.171	5.43	n.a.	BMB*
Total:			471.388	132.111	100.00	0.000	

Supplementary Figure 168. Chiral HPLC analysis of compound 5o

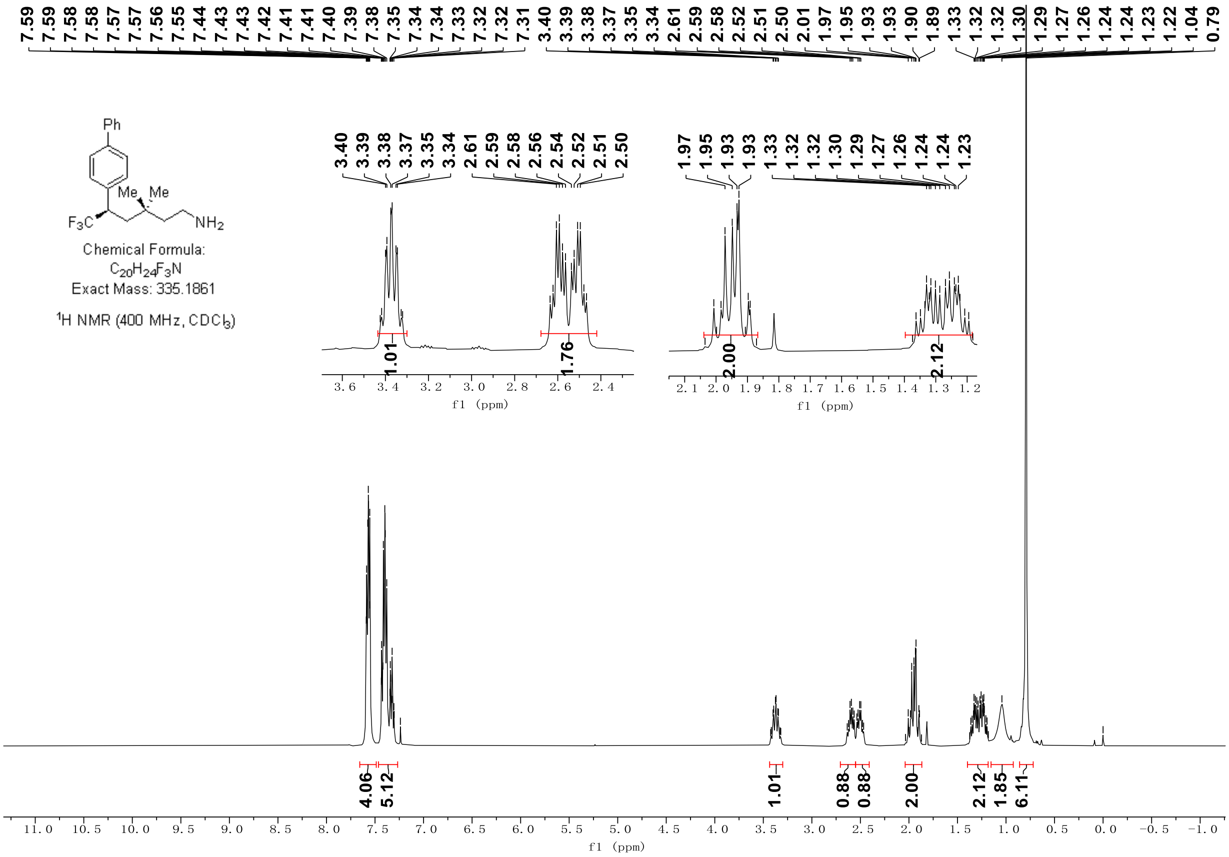

Supplementary Figure 169. ^{1}H NMR spectrum of compound 6

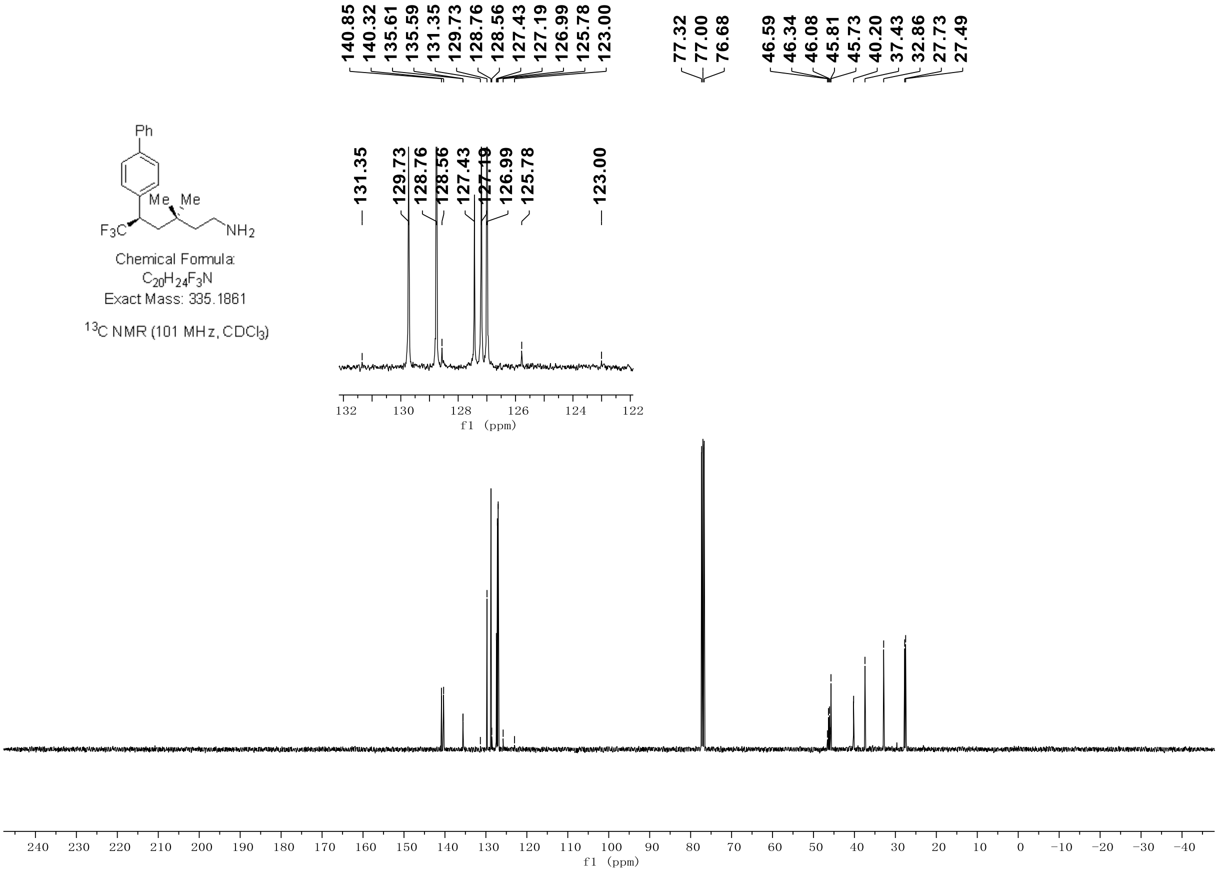

Supplementary Figure 170. ^{13}C NMR spectrum of compound 6

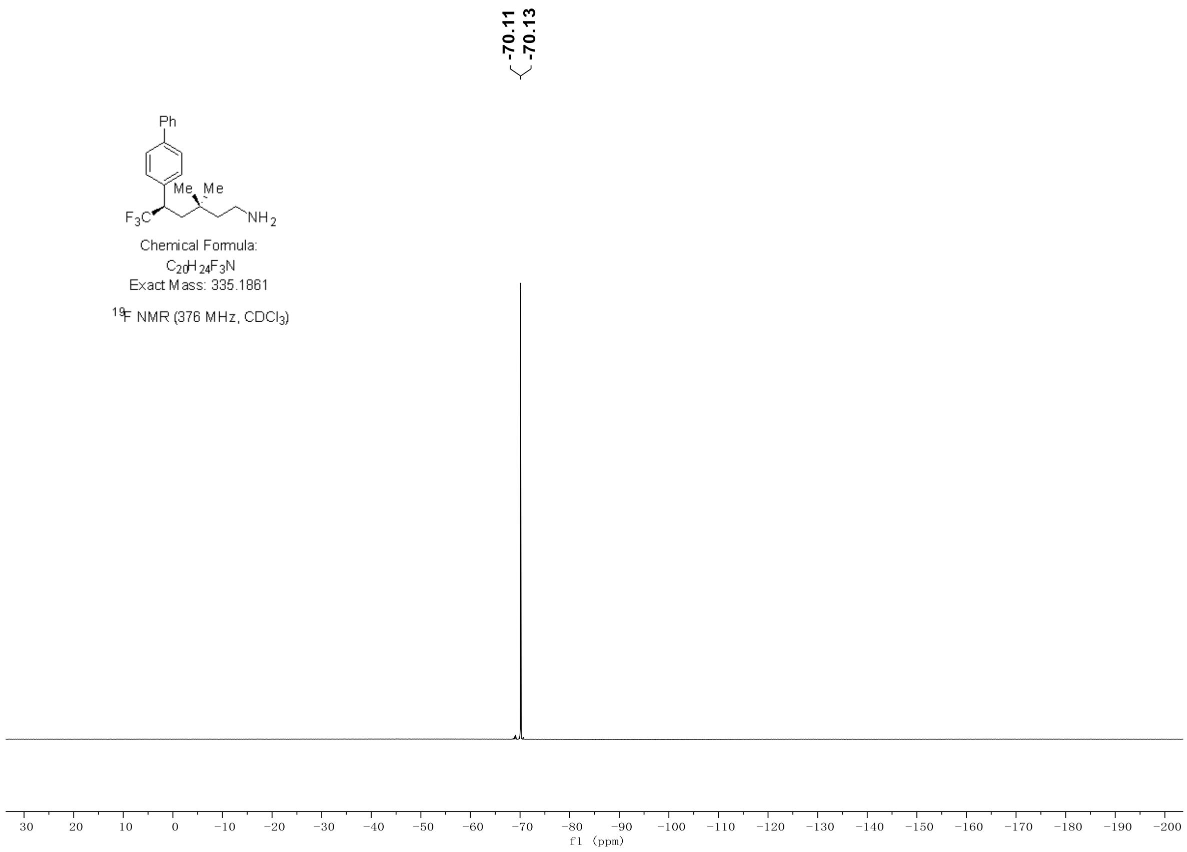

Supplementary Figure 171. ^{19}F NMR spectrum of compound 6

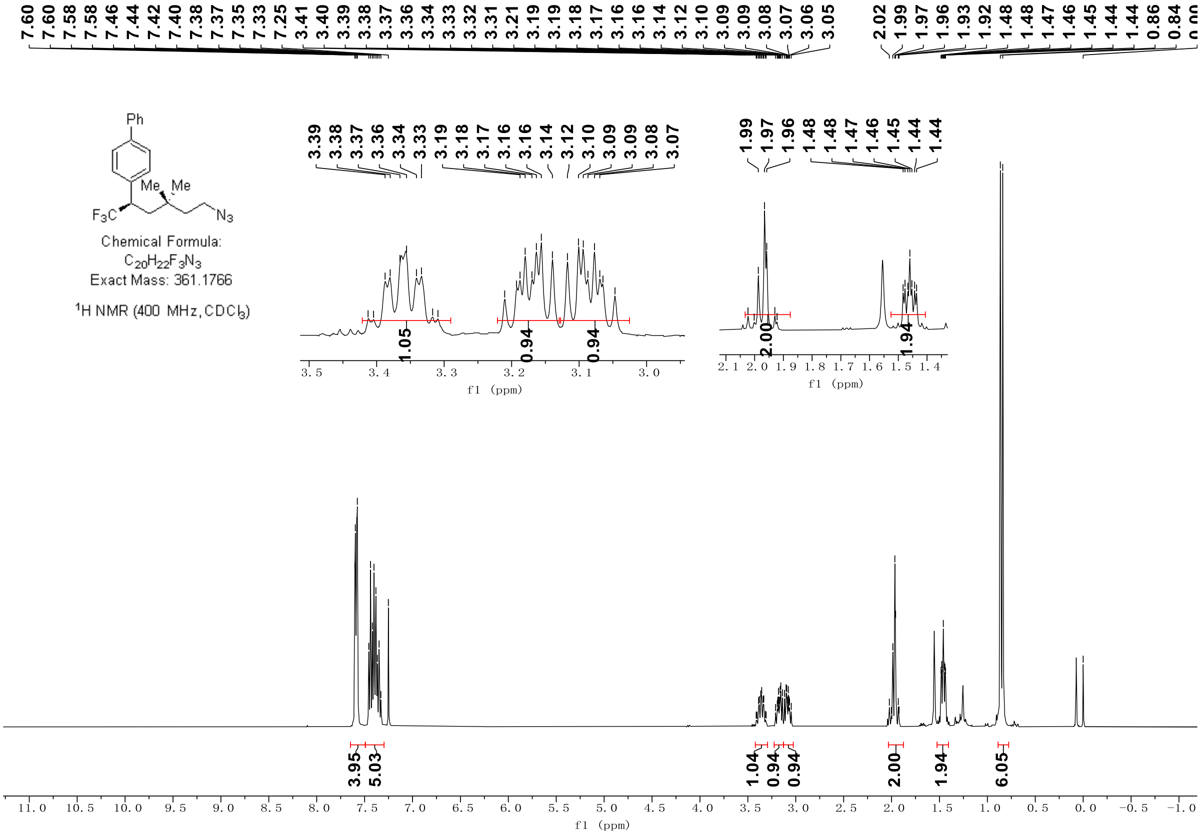

Supplementary Figure 172. ^{1}H NMR spectrum of compound 7a

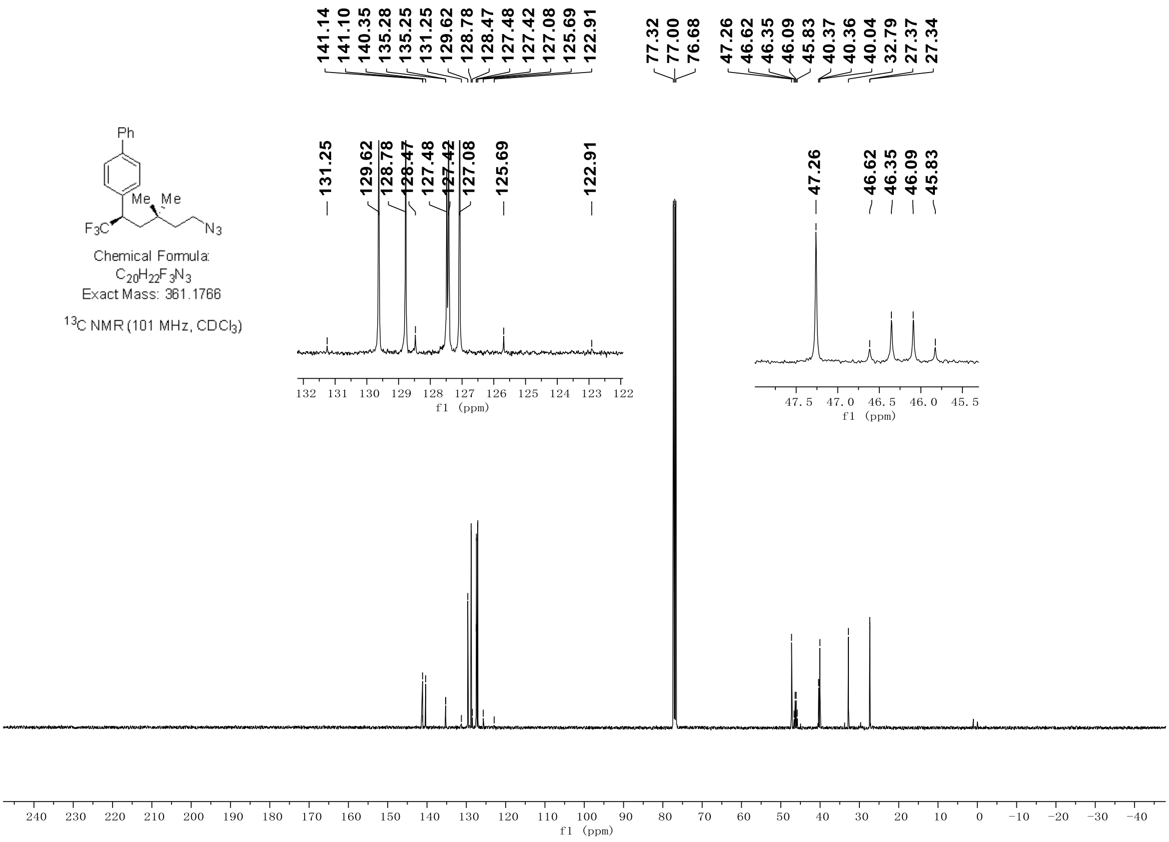

Supplementary Figure 173. ^{13}**C NMR spectrum of compound 7a**

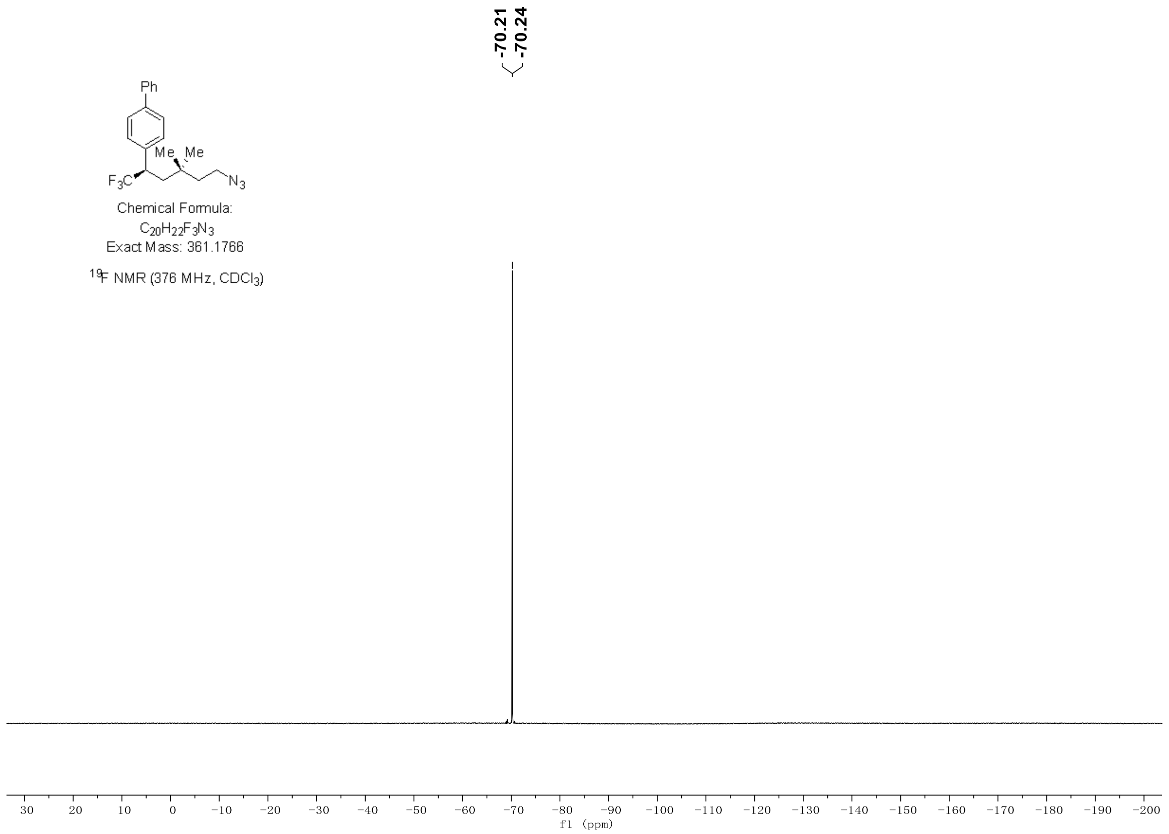

Supplementary Figure 174. ^{19}**F NMR spectrum of compound 7a**

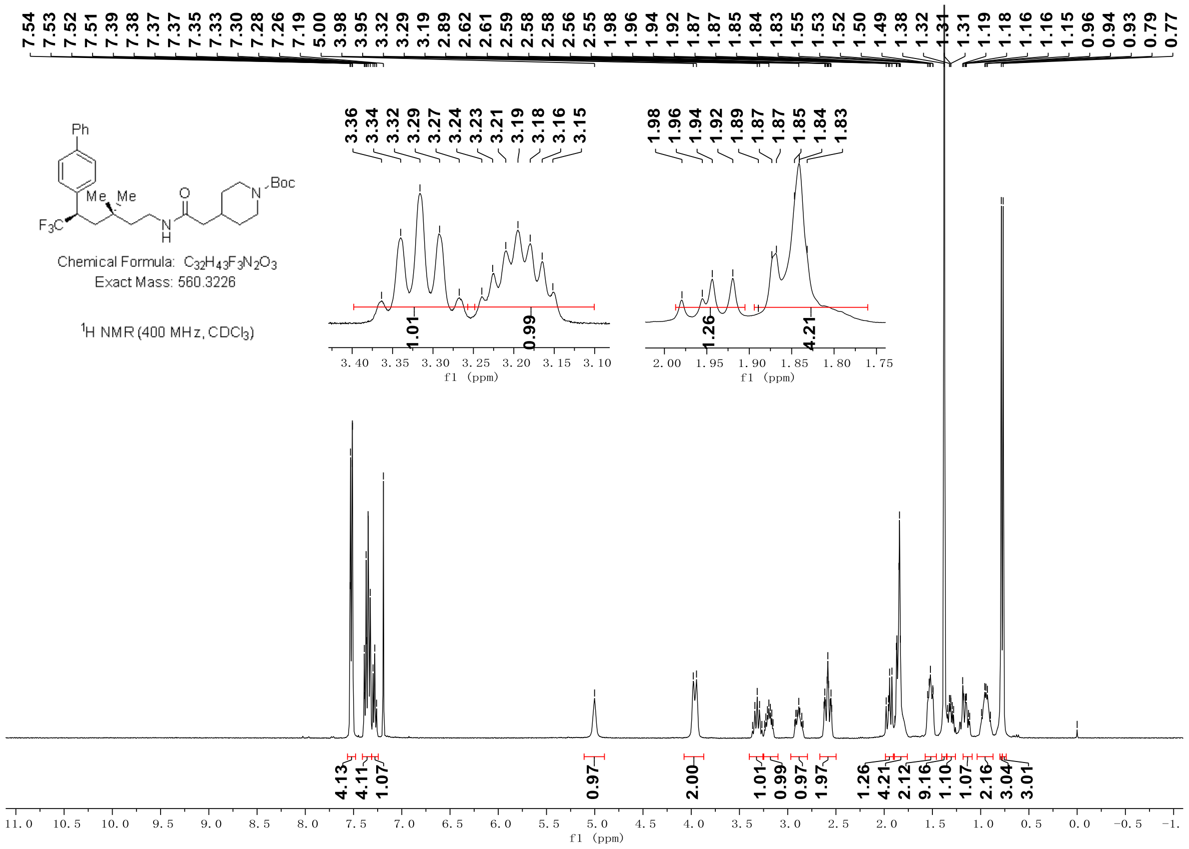

Supplementary Figure 175. ^{1}H NMR spectrum of compound 7b

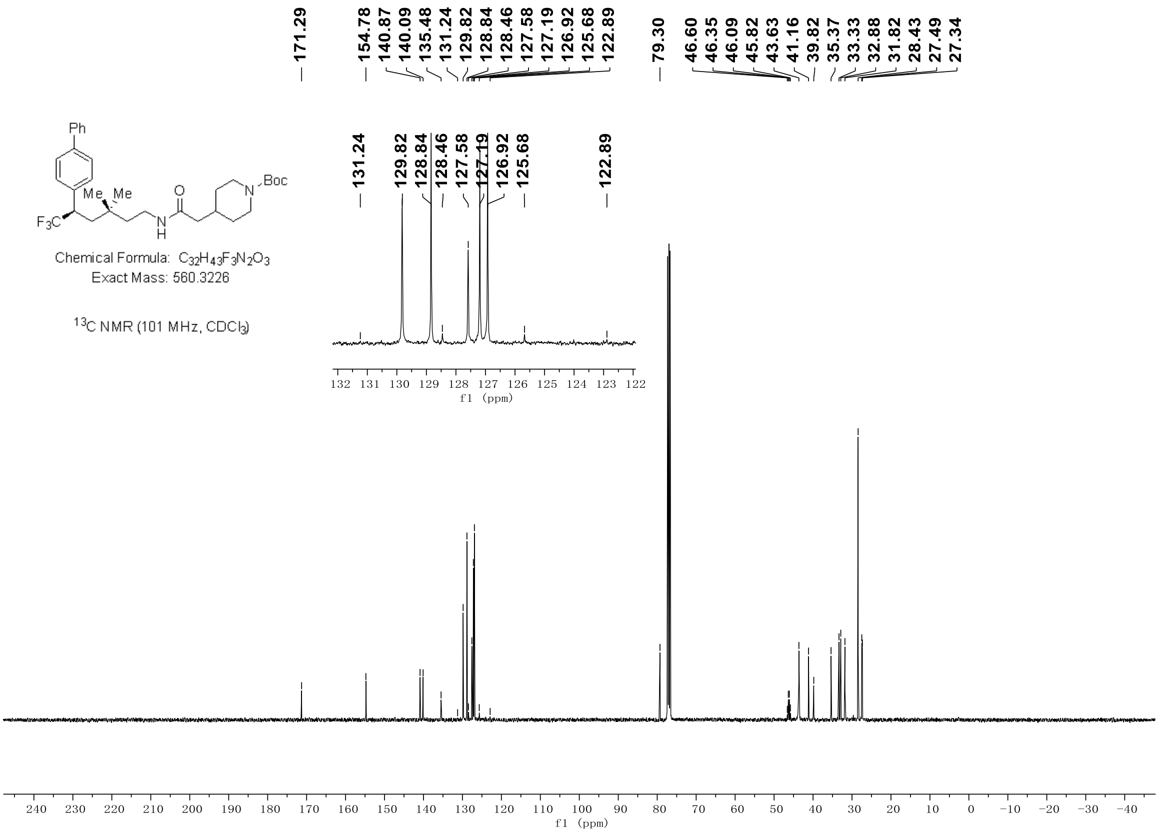

Supplementary Figure 176. ^{13}C NMR spectrum of compound 7b

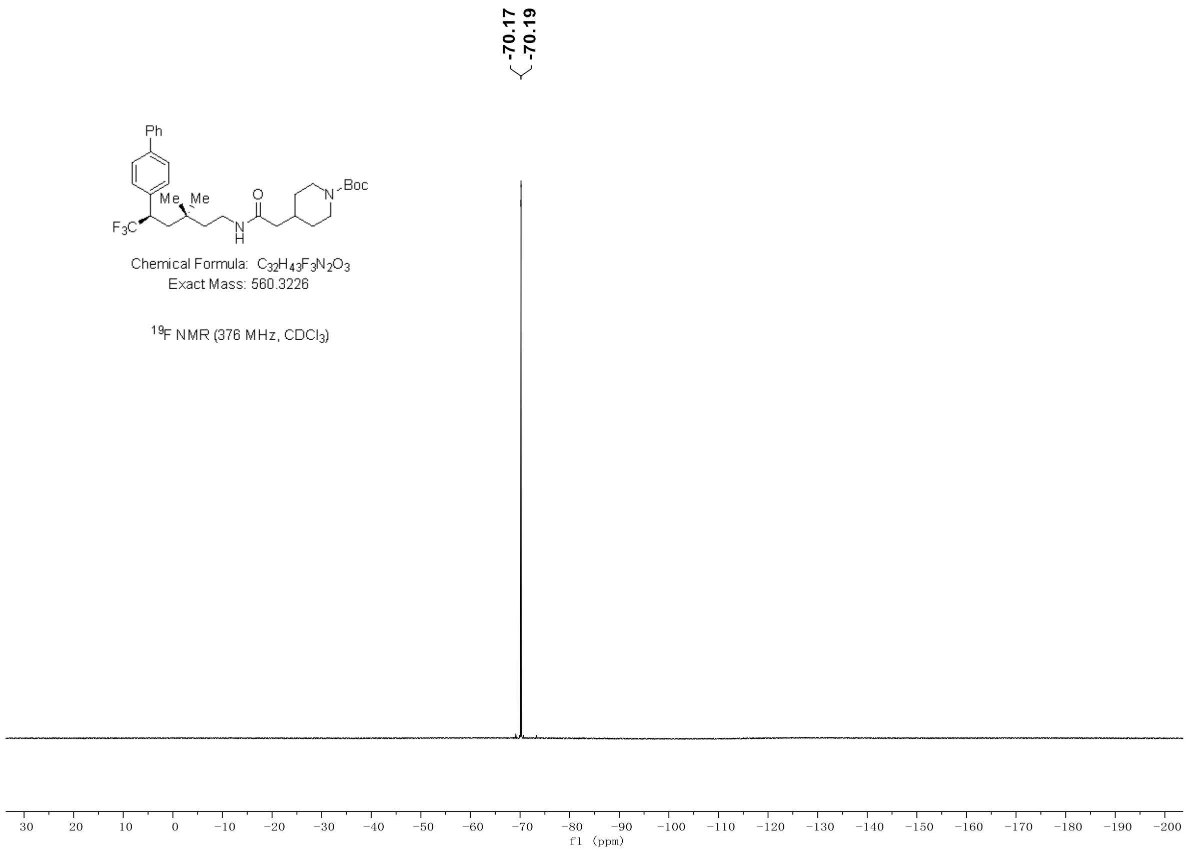

Supplementary Figure 177. ^{19}F NMR spectrum of compound 7b

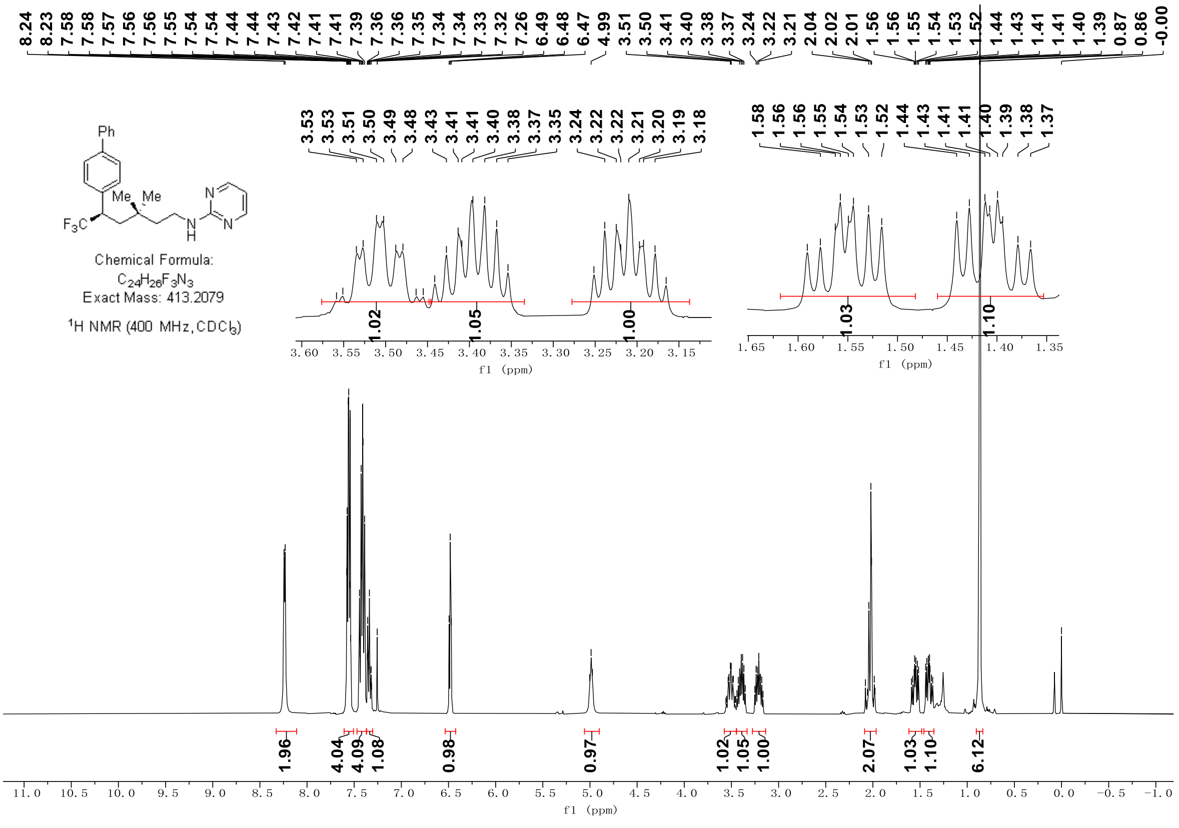

Supplementary Figure 178. ^{1}H NMR spectrum of compound 7c

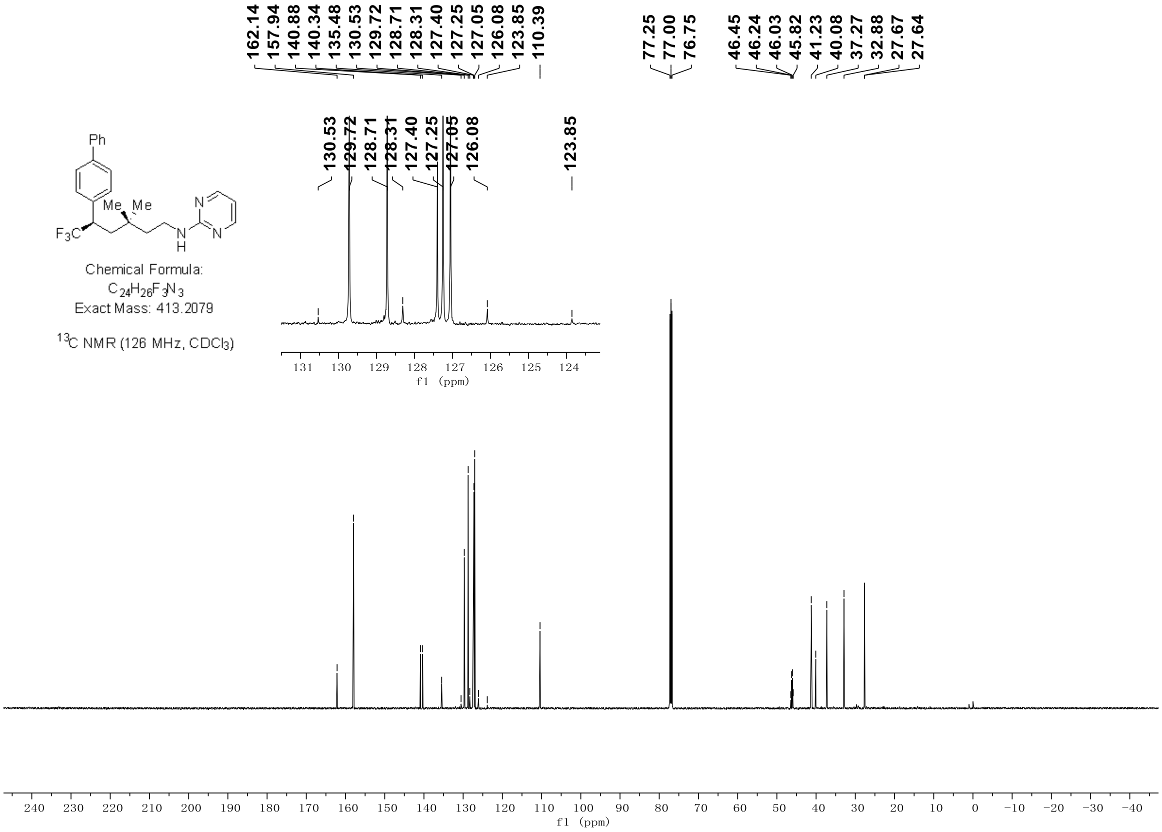

Supplementary Figure 179. ^{13}C NMR spectrum of compound 7c

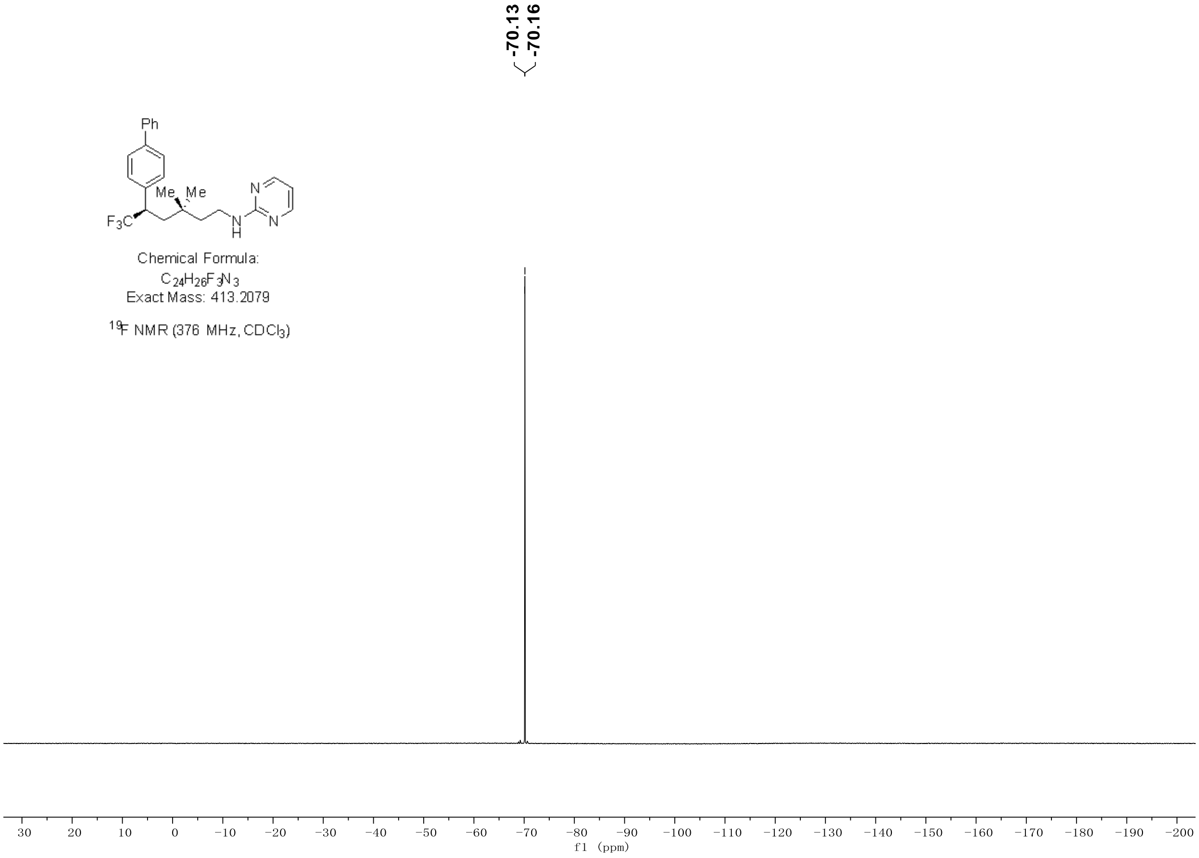

Supplementary Figure 180. ^{19}F NMR spectrum of compound 7c

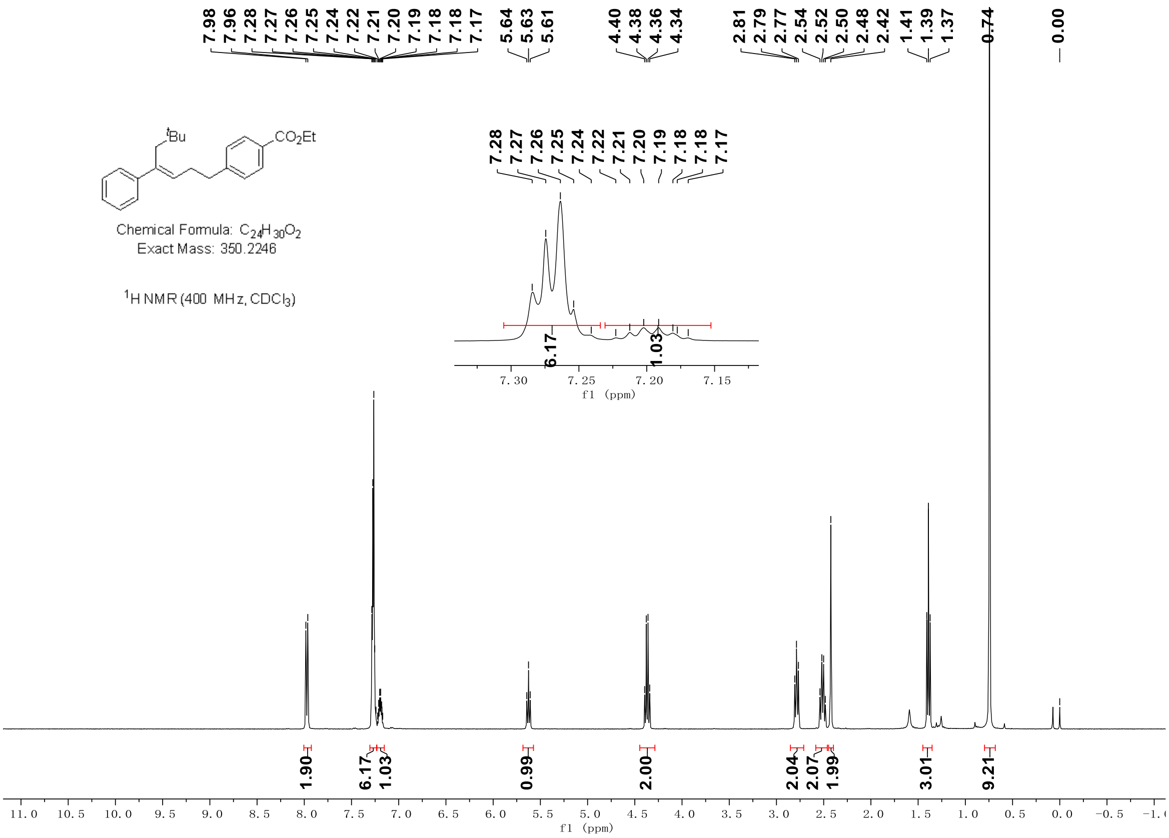

Supplementary Figure 181. ^{1}H NMR spectrum of compound 11a

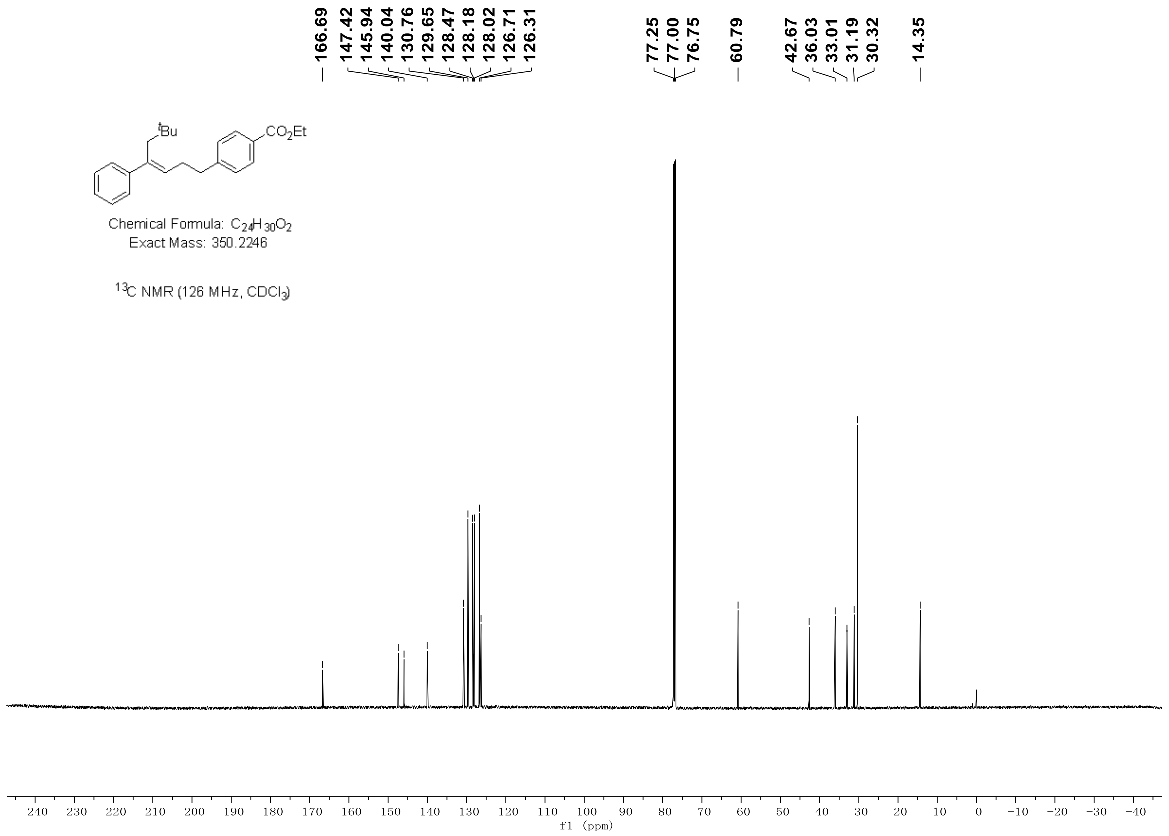

Supplementary Figure 182. ^{13}C NMR spectrum of compound 11b

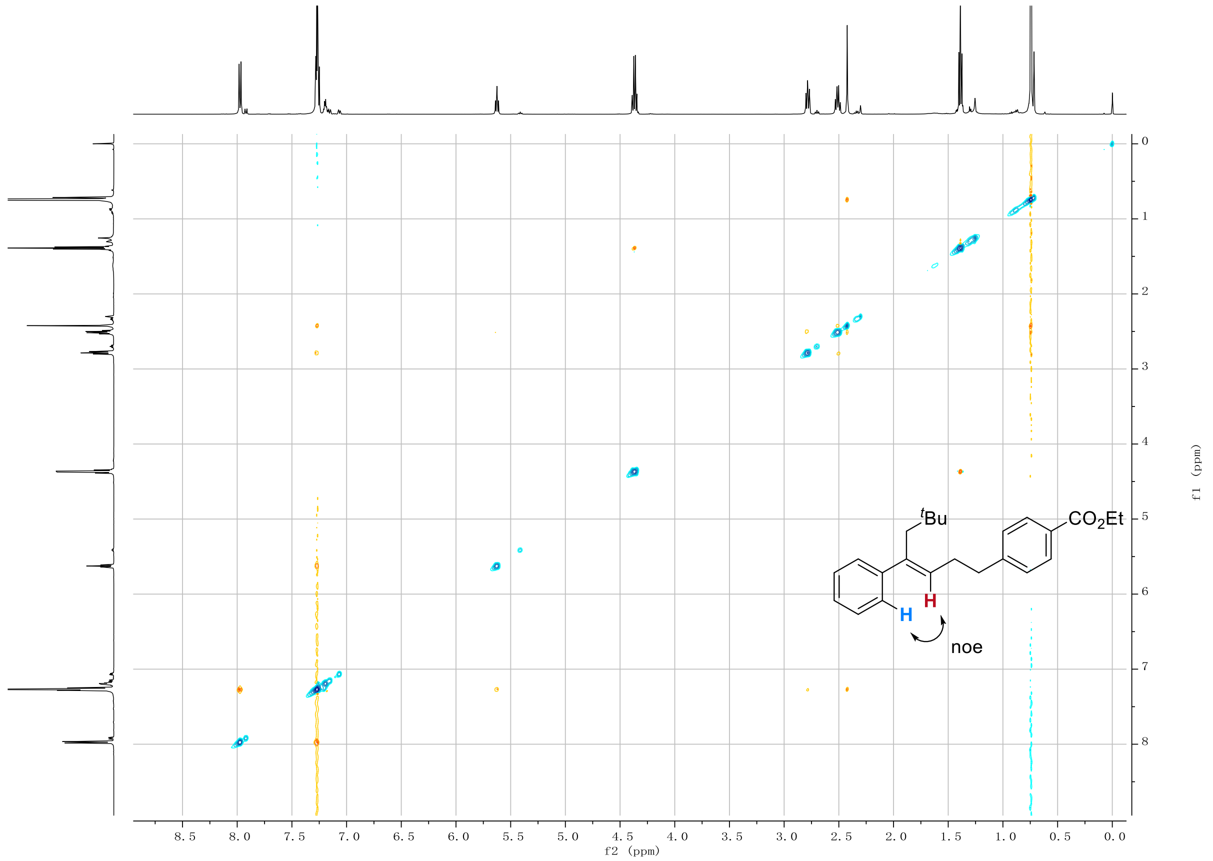

Supplementary Figure 183. NOE spectrum of compound 11a

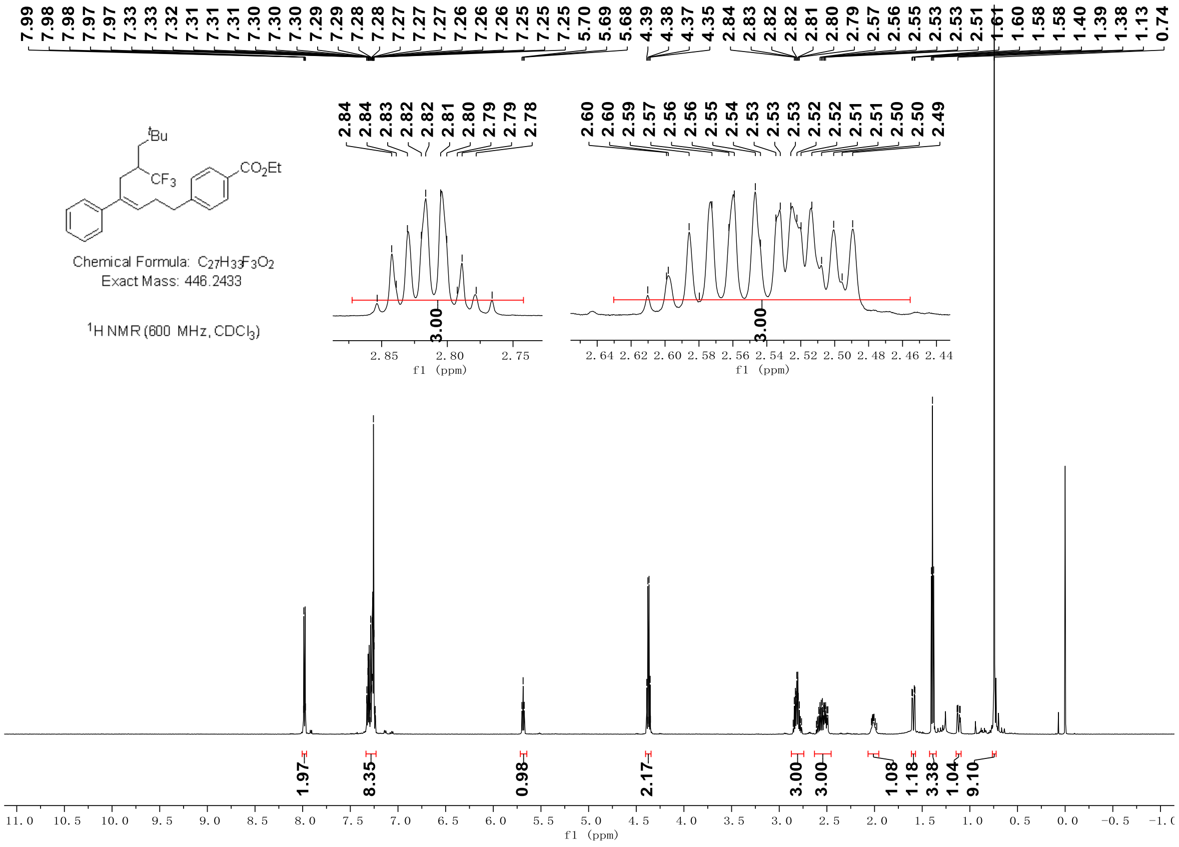

Supplementary Figure 184. ^{1}H NMR spectrum of compound 11b

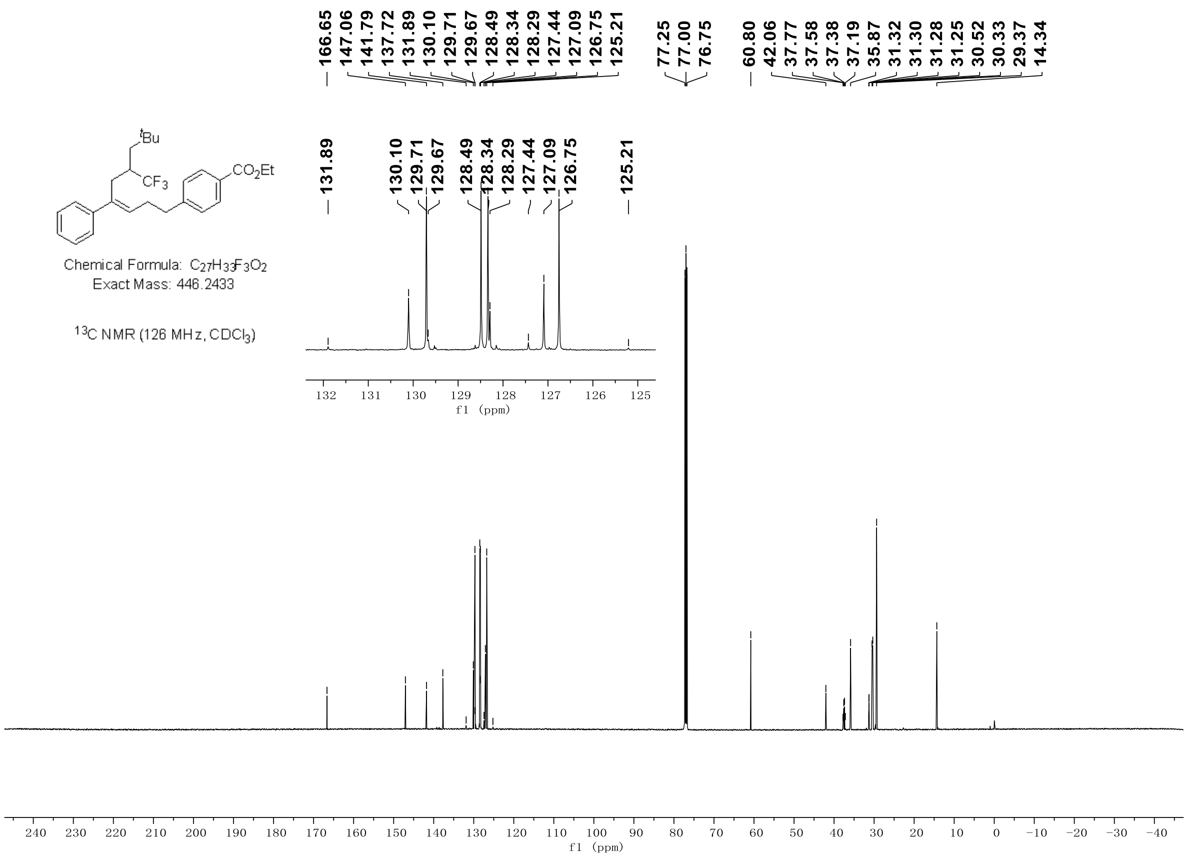

Supplementary Figure 185. ^{13}C NMR spectrum of compound 11b

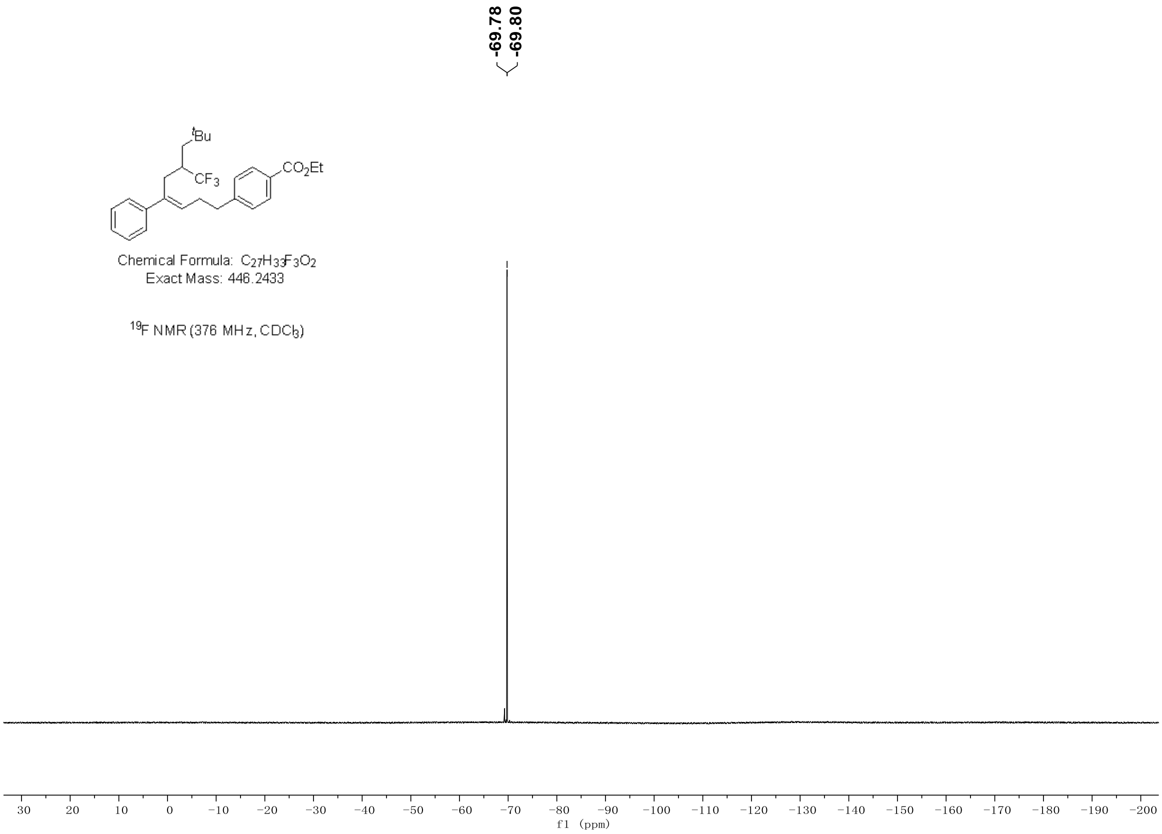

Supplementary Figure 186. ^{19}F NMR spectrum of compound 11b

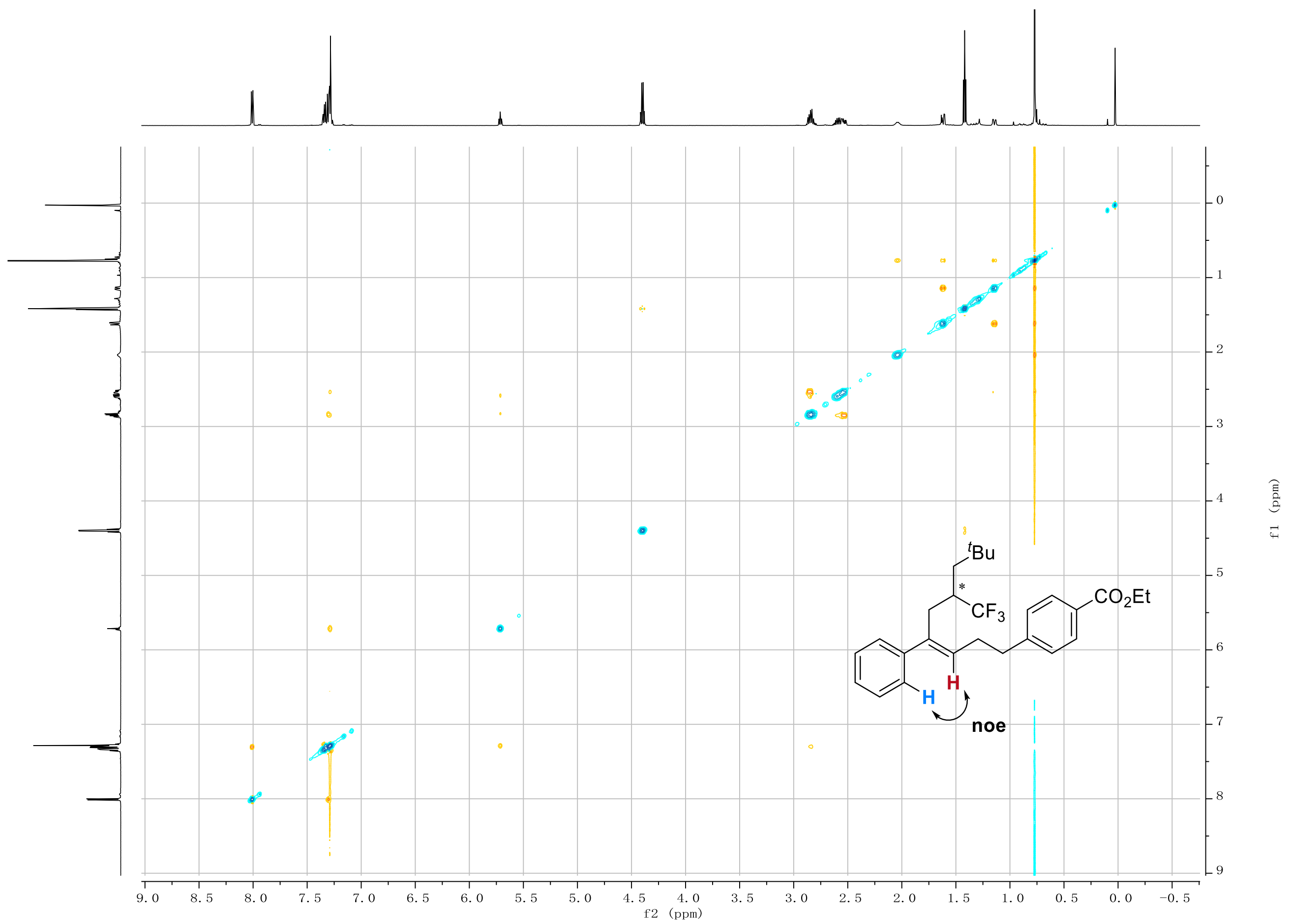

Supplementary Figure 187. NOE spectrum of compound 11b

V. Supplementary References

1. Chen, Y., Su, L. & Gong, H. Copper-catalyzed and indium-mediated methoxycarbonylation of unactivated alkyl iodides with balloon CO. *Org. Lett.* **21,** 4689-4693 (2019).
2. Chen, H., Liu, Z., Lv, Y., Tan, X., Shen, H., Yu, H. Z. & Li, C. Selective radical fluorination of tertiary alkyl halides at room temperature. *Angew. Chem., Int. Ed.* **56**, 15411-15415 (2017).
3. Shah, R., Farmer, L. A., Zilka, O., Van Kessel, A. T. M. & Pratt, D. A. Beyond DPPH: Use of fluorescence-enabled inhibited autoxidation to predict oxidative cell death rescue. *Cell. Chem. Biol.* **26**, 1594-1607 (2019).
4. Fleming, F. F., Shook, B. C., Jiang, T. & Steward, O. W. β-Siloxy unsaturated nitriles: Stereodivergent cyclizations to *cis*- and *trans*-decalins. *Tetrahedron* **59**, 737-745 (2003).
5. Salome, C., Salome-Grosjean, E., Park, K. D., Morieux, P., Swendiman, R., DeMarco, E., Stables, J. P. & Kohn, H. Synthesis and anticonvulsant activities of (*R*)-*N*-(4'-substituted)benzyl 2-acetamido-3-methoxypropionamides. *J. Med. Chem.* **53**, 1288-1305 (2010).
